# Genome size evolution in the diverse insect order Trichoptera

**DOI:** 10.1093/gigascience/giac011

**Published:** 2022-02-25

**Authors:** Jacqueline Heckenhauer, Paul B Frandsen, John S Sproul, Zheng Li, Juraj Paule, Amanda M Larracuente, Peter J Maughan, Michael S Barker, Julio V Schneider, Russell J Stewart, Steffen U Pauls

**Affiliations:** LOEWE Centre for Translational Biodiversity Genomics (LOEWE-TBG), Frankfurt 60325, Germany; Department of Terrestrial Zoology, Senckenberg Research Institute and Natural History Museum Frankfurt, Frankfurt 60325, Germany; LOEWE Centre for Translational Biodiversity Genomics (LOEWE-TBG), Frankfurt 60325, Germany; Department of Plant & Wildlife Sciences, Brigham Young University, Provo, UT 84602, USA; Data Science Lab, Smithsonian Institution, Washington, DC 20560, USA; Department of Biology, University of Rochester, Rochester, NY 14620, USA; Department of Biology, University of Nebraska Omaha, Omaha, NE 68182, USA; Department of Ecology and Evolutionary Biology, University of Arizona, Tucson, AZ 85721, USA; Department of Botany and Molecular Evolution, Senckenberg Research Institute and Natural History Museum Frankfurt, Frankfurt 60325, Germany; Department of Biology, University of Rochester, Rochester, NY 14620, USA; Department of Plant & Wildlife Sciences, Brigham Young University, Provo, UT 84602, USA; Department of Ecology and Evolutionary Biology, University of Arizona, Tucson, AZ 85721, USA; Department of Terrestrial Zoology, Senckenberg Research Institute and Natural History Museum Frankfurt, Frankfurt 60325, Germany; Department of Biomedical Engineering, University of Utah, Salt Lake City, UT 84112, USA; LOEWE Centre for Translational Biodiversity Genomics (LOEWE-TBG), Frankfurt 60325, Germany; Department of Terrestrial Zoology, Senckenberg Research Institute and Natural History Museum Frankfurt, Frankfurt 60325, Germany; Institute for Insect Biotechnology, Justus-Liebig-University, Gießen 35390, Germany

**Keywords:** biodiversity; *de novo* genome assembly, genomics, genomic diversity, genome duplication, genome size evolution, insects, repetitive elements, transposable elements, Trichoptera

## Abstract

**Background:**

Genome size is implicated in the form, function, and ecological success of a species. Two principally different mechanisms are proposed as major drivers of eukaryotic genome evolution and diversity: polyploidy (i.e., whole-genome duplication) or smaller duplication events and bursts in the activity of repetitive elements. Here, we generated *de novo* genome assemblies of 17 caddisflies covering all major lineages of Trichoptera. Using these and previously sequenced genomes, we use caddisflies as a model for understanding genome size evolution in diverse insect lineages.

**Results:**

We detect a ∼14-fold variation in genome size across the order Trichoptera. We find strong evidence that repetitive element expansions, particularly those of transposable elements (TEs), are important drivers of large caddisfly genome sizes. Using an innovative method to examine TEs associated with universal single-copy orthologs (i.e., BUSCO genes), we find that TE expansions have a major impact on protein-coding gene regions, with TE-gene associations showing a linear relationship with increasing genome size. Intriguingly, we find that expanded genomes preferentially evolved in caddisfly clades with a higher ecological diversity (i.e., various feeding modes, diversification in variable, less stable environments).

**Conclusion:**

Our findings provide a platform to test hypotheses about the potential evolutionary roles of TE activity and TE-gene associations, particularly in groups with high species, ecological, and functional diversities.

## Background

Genome size is a fundamental biological character. Studying its evolution may potentially lead to a better understanding of the origin and underlying processes of the myriad forms and functions of plants and animals. These diversification processes remain at the core of much biological research. Given their high species, ecological, and functional diversities, insects are excellent models for such research. To date 1,345 insect genome size estimates have been published [1], ranging 240-fold from 69 Mb in chironomid midges [2] to 16.5 Gb in the mountain grasshopper *Podisma pedestris* [3]. Genome size variation relates poorly to the number of coding genes or the complexity of the organism (C-value enigma [4–7]), and evolutionary drivers of genome size variation remain a topic of ongoing debate (e.g., [8–11]). Two principally different mechanisms are proposed as primary drivers of eukaryotic genome size evolution: whole-genome duplication (WGD, i.e., polyploidy) or smaller duplication events and expansion of repetitive elements (REs [6]). While WGD is ubiquitous in plant evolution, it has been regarded as the exception in animals [12, 13]. However, ancient WGD has been hypothesized to be an important driver of evolution of mollusks (e.g., [14]), amphibians (e.g., [15, 16]), fish (e.g., [17–19]), and arthropods (e.g., [20–22]), including multiple putative ancient large-scale gene duplications within Trichoptera [23].

RE expansion is an important driver of genome size variation in many eukaryotic genomes [24, 25]. The two major categories of REs are tandem repeats (e.g., satellite DNA) and mobile transposable elements (TEs). TEs are classified into Class I (retrotransposons: endogenous retroviruses, related long terminal repeat [LTR], and non-LTR retrotransposons: SINEs [short interspersed nuclear elements], LINEs [long interspersed nuclear elements]) and Class II elements (DNA transposons [26]). In insects, the known genomic proportion of TEs ranges from 1% in the Antarctic midge *Belgica antarctica* [27] to 65% in the migratory locust *Locusta migratoria* [28]. Broad-scale analysis of TE abundance in insects suggests that some order-specific signatures are present; however, major shifts in TE abundance are also common at shallow taxonomic levels [29, 30], including in Trichoptera [31]. The movement and proliferation of REs can have deleterious consequences on gene function and genome stability [32–36]. Moreover, repeat content and abundance can turn over rapidly even over short evolutionary time scales (reviewed in [37]). This rapid evolution has consequences for genome evolution and speciation; e.g., repeat divergence causes genetic incompatibilities between even closely related species [38]. However, TEs can also be sources of genomic innovation with selective advantages for the host [39–44] and they can contribute to global changes in gene regulatory networks [45–47]. Investigating RE dynamics in diverse clades provides a powerful lens for understanding their roles in genome function and evolution. Broad study of RE dynamics in species-rich groups with wide variation in RE activity is an important step towards efficiently identifying study systems at finer taxonomical scales (natural populations, species complexes, or recently diverged species) that are ideally suited to advance our understanding of molecular and evolutionary mechanisms underlying genome evolution. In addition, by taking this biodiversity genomics approach, we can develop new model systems and eventually better understand links between environmental factors, genome size evolution, adaptation, and speciation (see [48]).

With >16,500 species, caddisflies (Trichoptera) are among the most diverse of all aquatic insects [49]. Their species richness is reflective of their ecological diversity, including, e.g., microhabitat specialization, a full array of feeding modes, and diverse use of underwater silk secretions [50, 51]. An initial comparison of 6 caddisfly species found wide genome size variation in Trichoptera (ranging from 230 Mb to 1.4 Gb). In that study, we hypothesized that the observed variation was correlated with caddisfly phylogeny and that TEs contributed to a suborder-specific increase of genome size [31].

Here, we present a multi-faceted analysis to investigate genome size evolution in the order Trichoptera, as an example for highly diversified non-model organisms. Specifically, we (i) estimated genome size for species across the order to explore phylogenetic patterns in the distribution of genome size variation in Trichoptera and (ii) generated 17 new Trichoptera genomes to analyze, in conjunction with 9 existing genomes, the causes (WGD, TE expansions) of genome size variation in the evolution of caddisflies. Studying the genomic diversity of this highly diversified insect order adds new insights into drivers of genome size evolution with potential to shed light on how genome size is linked to form, function, and ecology.

## Data Description

### Genomic resources

Here, we combined long- and short-read sequencing technologies to generate 17 new *de novo* genome assemblies across a wide taxonomic range, covering all major lineages of Trichoptera. Details on sequencing coverage and assembly strategies are given in Supplementary Data File S1.2, Supplementary Data File S1.3 and Supplementary Note 3. To assess quality, we calculated assembly statistics with QUAST v5.0.2 [52], examined gene completeness with BUSCO v5 [53, 54], and screened for potential contamination with taxon-annotated GC-coverage (TAGC) plots using BlobTools v1.0 ([55], Supplementary Figs S31–S47). The new genomes are of comparable or better quality than other Trichoptera genomes previously reported in terms of BUSCO completeness and contiguity (Table 1). This study increases the number of assemblies in this order from 9 to 26, nearly tripling the number of available caddisfly genomes and thus providing a valuable resource for studying genomic diversity across this ecologically diverse insect order. The annotation of these genomes predicted 6,413–12,927 proteins (Supplementary Data File S1.2). Most of the annotated proteins (94.4–98.8%) showed significant sequence similarity to entries in the NCBI nr database. GO Distributions were similar to previously annotated caddisfly genomes, i.e., the major biological processes were cellular and metabolic processes. Catalytic activity was the largest subcategory in molecular function, and the cell membrane subcategories were the largest cellular component (Supplementary Figs S1–S30). This project has been deposited at NCBI under BioProject ID: PRJNA558902. For accession numbers of individual assemblies see Table 1.

**Table 1:** Comparison of assembly and annotation statistics of all available Trichoptera genomes

Species	Abbre-viation	Accession No.	Length (bp)	N50 (kb)	No. of contigs/scaffolds	BUSCOs**
*Agapetus fuscipes* ^c^	AF	JAGTXP000000000	552,637,417	2.8	296,752/291,536	C: 43.8% [S: 43.0%, D: 0.8%], F: 35.2%, M: 21.0%
*Agraylea sexmaculata* *	AS	JAGTTH000000000	196,044,125	86	7,077/7,050	C: 94.2% [S: 88.6%, D: 5.6%], F: 1.9%, M: 3.9%
*Agrypnia vestita* [30]	AV	GCA_016648135.1	1,352,945,503	111.8	25,541/25,153	C: 87.5% [S: 77.1%, D: 10.4%], F: 6.1%, M: 6.4%
*Drusus annulatus* *	DA	JAGWCC000000000	727,941,535	1,043.7	2,401	C: 90.3% [S: 89.6%, D: 0.7%], F: 6.5%, M: 3.2%
*Glossosoma conforme* *	GC1	JAGTXR000000000	568,249,599	2,212.1	653	C: 90.1% [S: 89.1%, D: 1.0%], F: 2.7%, M: 7.2%
*Glossosoma conforme* [56]	GC2	GCA_003347265.1	604,293,666	17.1	132,934/119,821	C: 78.4% [S: 77.3%, D: 1.1%], F: 15.0%, M: 6.6%
*Glyphotaelius pellucidula* [57]	GP	Glyphotaelius_pellucidus_k51_scaffolds	623,431,006	1.6	461,749	C: 20.3% [S: 19.7%, D: 0.6%], F: 39.9%, M: 39.8%
*Halesus radiatus* *	HR	JAHDVE000000000	973,356,502	125.2	12,636/12,484	C: 85.0% [S: 82.6%, D: 2.4%], F: 8.5%, M: 6.5%
*Himalopsyche phryganea* *	HP	JAGVSL000000000	633,785,554	4,634	710	C: 96.0% [S: 95.3%, D: 0.7%], F: 2.5%, M: 1.5%
*Hesperophylax magnus* [30]	HM	GCA_016648045.1	1,275,967,528	768.2	6,877	C: 88.4% [S: 80.7%, D: 7.7%], F: 6.4%, M: 5.2%
*Hydropsyche tenuis* [58]	HT	GCA_009617725.1	229,663,394	2,190.1	403	C: 95.7% [S: 94.9%, D: 0.8%], F: 2.4%, M: 1.9%
*Lepidostoma basale* *	LB	JAGTTH000000000	769,208,668	1,052	1,712/1,621	C: 94.4% [S: 93.1%, D: 1.3%], F: 3.5%, M: 2.1%
*Limnephilus lunatus* [59]	LL	GCA_000648945.2	1,369,180,260	69.1	69,049/58,718	70.4% [S: 66.2%, D: 4.2%], F: 20.8%, M: 8.8%
*Micrasema longulum* *	ML2	JAGXCS000000000	668,600,304	2.5	374,883/368,330	C: 45.0% [S: 44.1%, D: 0.9%], F: 34.5%, M: 20.5%
*Micrasema longulum* *	ML1	JAGVSM000000000	585,245,295	170.5	5,470/5,451	C: 78.6% [S: 77.6%, D: 1.0%], F: 5.4%, M: 16.0%
*Micrasema minimum* *	MM	JAGVSQ000000000	329,257,313	69.5	7,561	C: 59.1% [S: 58.6%, D: 0.5%], F: 10.4%, M: 30.5%
*Micropterna sequax* *	MS	JAGUCF000000000	778,692,278	7.9	144,300/144,286	C: 44.4% [S: 41.7%, D: 2.7%], F: 29.6%, M: 26.0%
*Odontocerum albicorne* *	OA	JAGTXQ000000000	1,305,984,461	266.4	9,583/9,303	C: 92.2% [S: 90.6%, D: 1.6%], F: 5.4%, M: 2.4%
*Parapysche elsis* *	PE	JAGVSN000000000	282,185,525	5,591.7	159	C: 95.1% [S: 94.3%, D: 0.8%], F: 1.8%, M: 3.1%
*Philopotamus ludificatus* *	PL	JAGXCT000000000	360,300,449	67.5	44,049/37,274	C: 92.0% [S: 90.0%, D: 2.0%], F: 4.7%, M: 3.3%
*Plectrocnemia conspersa* [58]	PC	GCA_009,617,715.1	396,695,105	869	1,614	C: 94.7% [S: 93.8%, D: 0.9%], F: 2.5%, M: 2.8%
*Rhyacophila brunnea* *	RB	JAGYXB000000000	1,086,872,538	1,030.6	2,227/2,125	C: 95.4% [S: 92.0%, D: 3.4%], F: 2.5%, M: 2.1%
*Rhyacophila evoluta* *	RE2	JAGVSQ000000000	565,830,460	9.9	118,140/114,057	C: 75.1% [S: 74.3%, D: 0.8%], F: 17.9%, M: 7.0%
*Rhyacophila evoluta* *	RE1	JAGVSO000000000	562,550,625	9.7	115,243/111,706	C: 74.1% [S: 73.4%, D: 0.7%], F: 18.7%, M: 7.2%
*Sericostoma sp*.[60]	SS	GCA_003003475.1	1,015,727,762	3.2	561,698	C: 30.9% [S: 30.3%, D: 0.6%], F: 40.2%, M: 28.9%
*Stenopsyche tienhuanesis* [61]	ST	GCA_008973525.1	451,494,475	1,296.7	552	C: 95.3% [S: 92.4%, D: 2.9%], F: 2.3%, M: 2.4%

* Assemblies produced in this study.

** N_Arthropoda_ = 2,124. C: complete; S: single; D: duplicated; F: fragmented; M: missing.

We downloaded existing Trichoptera genomes from GenBank [62] or Lepbase [58] and used these in conjunction with our newly generated genomes to analyze genome size evolution as explained in the following sections of this manuscript.

### Flow cytometry

In addition to genomic sequence data, we used flow cytometry (FCM) to detect genome size variation across the order. Our study increased the number of species with available FCM-based genome size estimates from 4 [63] to 31. Estimates were submitted to the Animal Genome Size Database [1].

## Analysis

### Genome size evolution in Trichoptera

**Figure 1:**
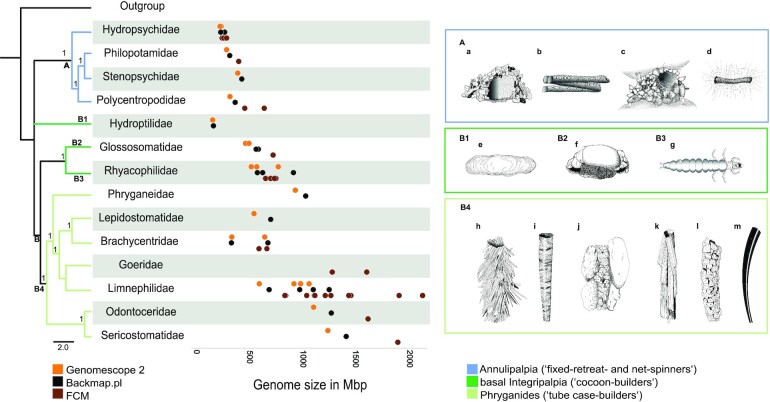
Ecological diversity (right) and genome size (left) in caddisflies. Phylogenetic relationships derived from ASTRAL-III analyses using single BUSCO genes. Goeridae, which was not included in the BUSCO gene set, was placed according to [64]. ASTRAL support values (local posterior probabilities) >0.9 are given for each node. The placement of Hydroptilidae (clade B1) was ambiguous. Because its placement was poorly supported in our analyses, we placed it according to Thomas et al. [64]. Taxa were collapsed to family level. Trichoptera are divided into two suborders: Annulipalpia (“fixed retreat– and net-spinners,” clade A: blue) and Intergripalpia (clade B: green), which includes basal Integripalpia (“cocoon-builders,” clades B1–B3, dark green) and Phryganides or “tube case–builders” (clade B4: light green). “Cocoon-builders” are divided into “purse case-building” (clade B1), “tortoise case-building” (clade B2), and “free-living” (clade B3) families. Genome size estimates based on different methods (Genomescope2: orange, Backmap.pl: black, flow cytometry [FCM]: brown) are given for various caddisfly families. Each dot corresponds to a mean estimate of a species. For detailed information on the species and number of individuals used in each method see Supplementary Data File S1.7. Colors and clade numbers in the phylogenetic tree refer to colored boxes with illustrations. The following species are illustrated by Ralph Holzenthal: a: *Hydropsyche sp*. (Hydropsychidae); b: *Chimarra sp*. (Philopotamidae); C: *Stenopsyche sp*. (Stenopsychidae); d: *Polycentropus* sp. (Polycentropodidae); e: *Agraylea sp*. (Hydroptilidae); f: *Glossosoma sp*. (Glossosomatidae); g: *Rhyacophila sp*. (Rhyacophilidae); h: *Fabria inornata* (Phryganeidae); i: *Micrasema* sp. (Brachycentridae); j:*Goera fuscula* (Goeridae); k: *Sphagnophylax meiops* (Limnephilidae); l: *Psilotreta sp*. (Odontoceridae), m: *Grumicha grumicha* (Sericostomatidae).

**Figure 2:**
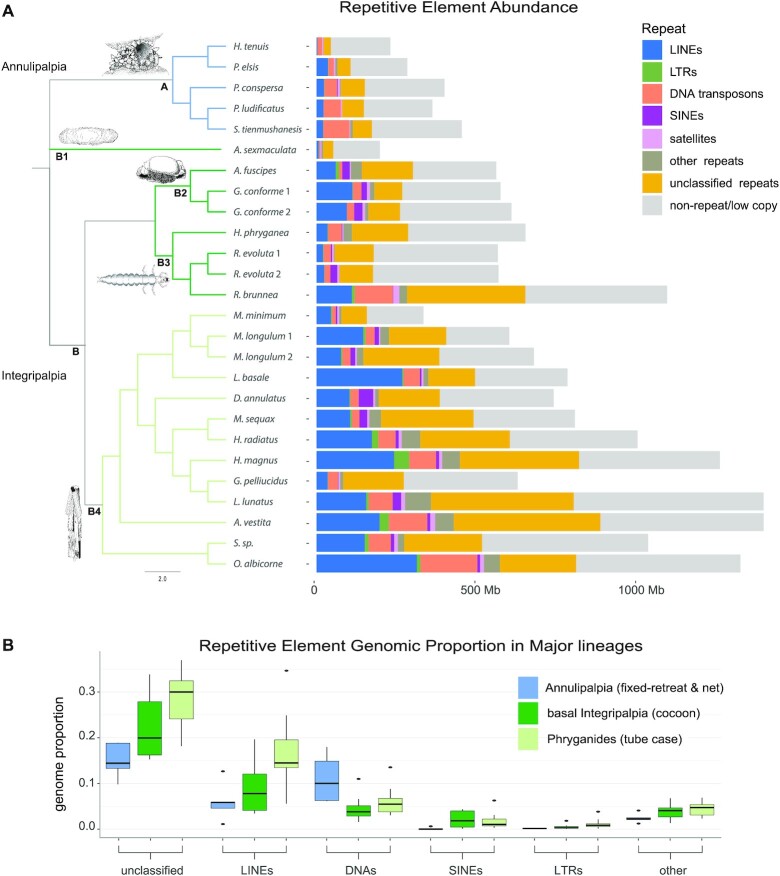
Repeat abundance and classification in 26 caddisfly genomes. Number of bp for each repeat type is given for each caddisfly genome. A: Repeat abundance and classification. Phylogenetic tree was reconstructed with ASTRAL-III using single BUSCO genes from the genome assemblies. The placement of Hydroptilidae (clade B1) was ambiguous. Because its placement was poorly supported in our analyses, we placed the single hydroptilid taxon (*Agraylea sexmaculata*) according to Thomas et al. [64]. Species names corresponding to the abbreviations in the tree can be found in Table 1. Trichoptera are divided into two suborders: Annulipalpia (“fixed retreat– and net-spinners,” clade A: blue) and Intergripalpia (clade B: green), which includes basal Integripalpia (“cocoon-builders,” clades B1–B3, dark green) and Phryganides or “tube case–builders” (clade B4: light green). “Cocoon-builders” are divided into “purse case-building” (clade B1), “tortoise case-building” (clade B2), and “free-living” (clade B3) families. An illustration of a representative of each clade is given. The “other repeats” category includes rolling circles, *Penelope*, low-complexity, simple repeats, and small RNAs. B: Box plots summarizing shifts in the genomic proportion of RE categories in major Trichoptera lineages. Colored rectangles in the boxplots show the first and third quartiles plotted around the median genomic proportion with outlier values shown as black dots.

On the basis of the genomes of 6 trichopteran species, Olsen et al. [31] found a 3-fold suborder-specific increase of genome size and hypothesized that genome size variation is correlated with their phylogeny. To test this hypothesis, we first reconstructed phylogenetic relationships by analyzing ∼2,000 single-copy BUSCO genes from the 26 study species (Figs 1 and 2, Supplementary Fig. S48). We obtained a molecular phylogeny that was in agreement with recent phylogenetic hypotheses ([64], see Supplementary Note 6) and that showed that Trichoptera is divided into two suborders: Annulipalpia (Figs 1 and 2: Clade A, blue) and Integripalpia (consisting of basal Integripalpia [Fig. 1: Clade B1–3, light green] and infraorder Phryganides [Fig. 1: clade B4, dark green]). Trichopterans use silk to build diverse underwater structures (see Fig. 1; Supplementary Note 6, Supplementary Fig. S48). Thus, we refer to Annulipalpia as “fixed retreat– and net-spinners,” to Phryganides (Integripalpia) as “tube case–builders,” and to basal Integripalpia as “cocoon-builders.”

We used 3 approaches for estimating genome size across Trichoptera: *k-*mer distribution estimates, back-mapping of sequence data to available draft genomes (as described in [65, see also 66]), and FCM (Supplementary Note 7, Figs S49–S72, Supplementary Data File S1.7). FCM estimates can be affected by chromatin condensation, the proportion of cells in G0–G1 phases [67, 68], and endoreplication in insect cells and tissues [69]. Sequence-based estimates can be affected by REs in the genome, resulting in smaller genome size estimates (e.g., [63, 70, 71]), as well as by GC-content because sequence library preparation including PCR amplification steps is associated with underrepresentation of GC- and AT-rich regions [72]. Bland-Altman plots (Supplementary Note 8, Supplementary Fig. S73) revealed general agreement of all 3 methods in our study. However, the FCM estimates were generally higher compared to the sequence-based estimates (Fig. 1, Supplementary Data File S1.7), and, among all 3 approaches, this measure is expected to be the most accurate [9]. We observe that variation among the methods increased with genome size, indicating issues potentially caused by repeat content (see section Repeat Dynamics).

We observed large variation in genome size across the order. Genome size tends to be lower in fixed retreat– and net-spinners and cocoon-builders compared to tube case–builders (Fig. 1). Specifically, we observe that genome size varies ∼14-fold, ranging from 1C = 154 Mb in cocoon-builders (Fig. 1, B1: Hydroptilidae) to 1C = 2,129 Mb in tube case–builders (Fig. 1, clade B4: Limnephilidae). Of the 29 species analyzed by FCM, *Halesus digitatus* (Fig. 1, clade B4: Limnephilidae, Intergripalpia) possessed the largest genome (1C = 2,129 Mb), while the genome of *Hydropsyche saxonica* (Fig. 1, clade A: Hydropsychidae, fixed retreat– and net-spinners) was the smallest (1C = 242 Mb). Genome size estimates based on sequence-based methods (*k-*mer–based and back-mapping) range from 1C = 154–160 Mb in *Agraylea sexmaculata* (Fig. 1, clade B1: Hydroptilidae, cocoon-builders) to 1C = 1,238–1,400 Mb in *Sericostoma* sp. (Fig. 1, clade B4: Sericostomatidae, tube case–builders).

## Repeat Dynamics

### Repetitive element abundance and classification

To understand the structural basis of genome size variation across the order Trichoptera we explored RE content. We found that major expansions of transposable elements (TEs) contribute to larger genomes in tube case–builders and some cocoon-builders, but particularly in tube case–builders with a average of ∼600 Mb of REs compared to ∼138 Mb in fixed retreat– and net-spinners (Fig. 2, Supplementary Data File S2.1). LINEs are the most abundant classified TEs in cocoon- and tube case–builders and comprise >154 Mb on average in tube case–builders, or a mean genome proportion of 16.9% (range = 5.6–34.7%). This represents a 1.8- and 2.8-fold increase in genome proportion relative to cocoon-builders and fixed retreat– and net-spinners, respectively. The LINE abundance of >312 Mb in *Odontocerum albicorne* exceeds the entire assembly lengths (152–282 Mb) of the 3 smallest genome assemblies (*Hydropsyche tenuis, Parapsyche elsis*, and *A. sexmaculata*) (Fig. 2). DNA transposons also comprise large genomic fractions in both cocoon- and tube case–builders (mean of 54.4 and 32.8 Mb, respectively). However, despite containing a large number of base pairs, they make up a smaller fraction of total base pairs in the genomes of cocoon- and tube case–builders than in fixed retreat– and net-spin, ners (mean genome proportion = 5.9%, 4.5%, and 11.1% in tube case–builders, cocoon-builders, and fixed retreat– and net-spinners, respectively) (Fig. 2B) and thus cannot, by themselves, explain the larger genome sizes. SINEs, LTRs, *Penelope* (grouped with “other” repeats in Fig. 2), and satellite DNAs show a disproportionate increase in cocoon- and tube case–builders; however, all categories combined make up a relatively small proportion of their genomes (all <3% on average in Integripalpia) (Fig. 2B). Unclassified repeats are the most abundant repeat category across all Trichoptera, and they also show disproportionate expansions in both cocoon- and case-builders relative to fixed retreat– and net-spinners (Fig. 2). The general trends noted in our assembly-based analysis of REs were corroborated by our reference-free analysis of repeat abundance (Supplementary Data File S2.2, Supplementary Data File S2.3, Supplementary Figs S146 and S147, Supplementary Note 10).

#### TE age distribution analysis

**Figure 3:**
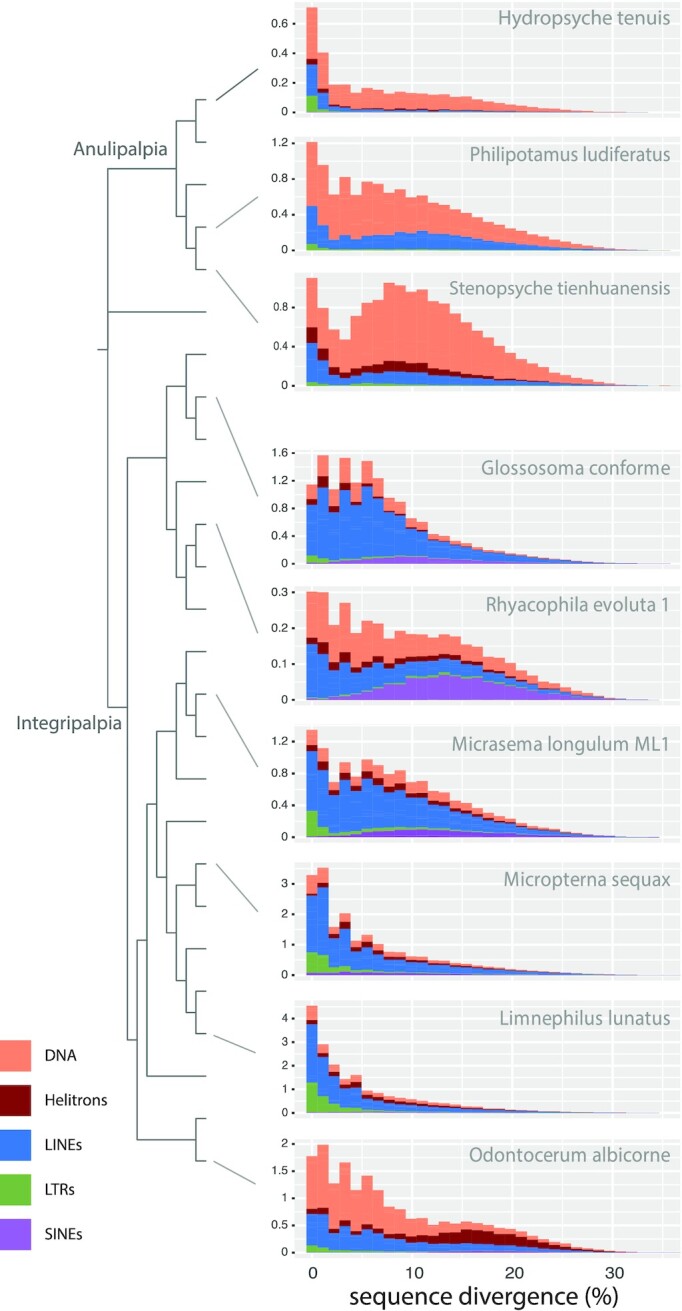
Transposable element age distribution landscapes. Representative examples are chosen from major Trichoptera lineages. The y-axis shows TE abundance as a proportion of the genome (e.g., 1.0 = 1% of the genome). The x-axis shows sequence divergence relative to TE consensus sequences for major TE classes. TE classes with abundance skewed toward the left (i.e., low sequence divergence) are inferred to have a recent history of diversification relative to TE classes with right-skewed abundance. Plots were generated in dnaPipeTE. Plots for all species are shown in Supplementary Fig. S148. For tip labels of the phylogenetic tree see Fig. 2.

To test whether the observed abundance patterns of specific TEs are driven by shared ancient proliferation events or more recent/ongoing activity of the respective TEs, we analyzed TE age distribution plots. These plots allow us to visualize specific RE classes/superfamilies that account for shifts in RE composition and abundance and infer the relative timing of those shifts based on the distribution of sequence divergence within each RE category. TE age distributions showed a high abundance of recently diverged TE sequences in cocoon- and tube case–builders, particularly in LINEs, DNA transposons, and LTRs in which the majority of TEs for a given class show 0–10% sequence divergence within copies of a given repeat (Fig. 3). This trend was particularly pronounced among tube case–builders, with several species showing high abundance of LINEs and DNA transposons with 0–5% sequence divergence (Fig. 3). This pattern suggests that the observed TE expansion is due primarily to ongoing TE activity within lineages rather than a few shared bursts of activity in ancestral lineages. This is further supported by our analysis of repeat subclasses with age distribution plots (Supplementary Fig. S148). For example, in our study, LINE abundance is often due to the expansion of different LINE subclasses even between species in the same sub-clade (e.g., compare *Lepidostoma* with *Micrasema, Himalopsyche* with *Glossosoma*; Supplementary Fig. S148). We also find evidence of shared ancient bursts of SINE activity in cocoon- and tube case–builders, although SINEs are not an abundant repeat class in any species (mean genomic proportion = 1.9% [SD 1.7%]) (Supplementary Fig. S148).

#### Associations between TE sequences and protein-coding genes

**Figure 4:**
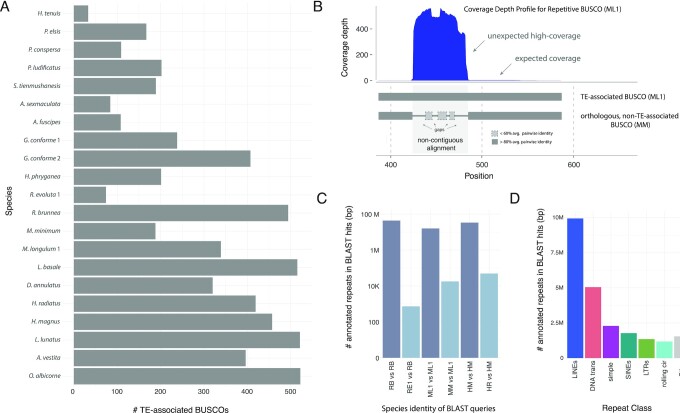
TE-BUSCO-gene associations in Trichoptera species. (A) Raw abundance of TE-associated BUSCO sequences present in the assembly of 2,442 BUSCOs in the OrthoDB 9 Endopterygota dataset. (B) *Top:* An example of a coverage depth profile of a TE-associated BUSCO gene (BUSCO EOG090R02Q9 from ML1 [“inflated species”]) that shows unexpected high coverage in the second exon putatively due to the presence of an RE-derived sequence fragment. *Bottom:* A typifying alignment between a TE-associated BUSCO and its orthologous BUSCO from a closely related species (“reference species”) that lacks TE-association. The non-TE-associated orthologous BUSCO shows non-contiguous alignment in regions of inflated coverage in the TE-associated BUSCO, consistent with the presence of an RE-derived sequence fragment in the TE-associated BUSCO that is absent in the reference species. (C) Summary of total bases annotated as REs obtained from each of the two BLAST searches. First, when we used BLAST to compare any TE-associated BUSCOs against an assembly for the same species, BLAST hits included megabases of annotated repeats (dark bars). Second, when non-TE-associated orthologs of the TE-associated BUSCOs in the first search are taken from a close relative and compared against the inflated species using BLAST, there is a dramatic decreae in BLAST hits annotated as REs. Note log scale on the y-axis. (D) Summary of annotations for BLAST hits for classified REs when TE-associated BUSCOs are compared against an assembly of the same species using BLAST.

During early exploration of our sequence data, we made an unexpected discovery that in some lineages, universal single-copy orthologs, or “BUSCO genes,” showed higher than expected coverage depth of mapped reads in 1 or more of their sequence fragments. Further analysis showed that these high-coverage BUSCO sequence regions are typically RE sequences (primarily TEs) that are either embedded within or located immediately adjacent to BUSCO genes, such that the BUSCO algorithm includes them in its annotation of a given gene. We refer to BUSCO genes containing these putative RE fragments as “TE-associated BUSCOs” (Supplementary Fig. S149, Supplementary Note 11). By estimating how many times they occur, we can quantitatively measure how TE-gene interactions change with changing genome size. In fact, we detected a positive linear relationship between TE-gene interactions and increasing genome size when measured with this accidently discovered metric. We found major expansions of TE-associated BUSCOs in cocoon- and tube case–builders (Fig. 4A) that are significantly correlated with total repeat abundance, as well as the genomic proportion of LINEs and DNA transposons (Supplementary Fig. S150). TE-associated BUSCOs comprise a relatively large fraction of total BUSCO genes in these lineages (mean of 11.2% and 21.4% of total BUSCOs in cocoon- and tube case–builders, respectively), compared to annulipalpian lineages (mean = 6.2%). This finding highlights the major impact of REs on the composition of protein-coding genes in species with repeat-rich genomes. The BUSCO-associated sequences may represent TEs recently inserted into BUSCO genes, the remnants left behind following historical TE transposition events, or TE sequences that are immediately adjacent to and inadvertently classified as BUSCO sequences.

To confirm that unexpectedly high-coverage sequence regions in TE-associated BUSCOs were in fact TE-derived sequences, we compared patterns of BUSCO gene structure (through pairwise alignment) across species pairs in which high-coverage regions (i.e., putative TE sequences) were present in the BUSCO gene of 1 species (i.e., the “inflated” species) but absent in the homologous BUSCO of the other (i.e., the “reference” species). This analysis showed that in 73 of 75 randomly sampled alignments, reference species showed gaps or highly non-contiguous alignments in high-coverage regions of the inflated species (Fig. 4B), suggesting that sequence insertions are typically present in high-coverage sequence regions of TE-associated BUSCOs. Our subsequent BLAST analysis showed that comparing a TE-associated BUSCO against its own assembly produced thousands to millions of BLAST hits from many contigs (Fig. 4C). This confirmed that the indel sequence present in high-coverage regions of inflated species shows high sequence similarity to REs elsewhere in the genome. We then used an intersect analysis on the BLAST results to confirm that the large majority of the excess BLAST hits overlap with RE annotations throughout the genome, most of which are TEs, with LINEs and DNA transposons being most abundant (Fig. 4D, Supplementary Data File S2.5). Finally, we found that if we replaced the TE-associated BLAST query sequence with the homologous but non-TE-associated BUSCO from its counterpart reference species, the number of BLAST hits was fewer (Fig. 4C, Supplementary Data File S2.6), offering further evidence that the TE sequence insertions driving the pattern of high coverage in read mapping excess BLAST hits are absent in reference species and thus carriable across relatively short time scales within Trichoptera. Taken together, these findings provide strong evidence that TE sequences (especially LINEs and DNA transposons) inadvertently annotated by BUSCO can account for the high-coverage regions that we observe in BUSCO genes (Fig. 4D).

Our accidental discovery that quantifying the frequency of TE-associated BUSCOs can serve as an estimate of TE-gene associations may prove useful in other systems given the wide use of BUSCO analysis in genomic studies. Finer details supporting the TE-gene association analysis are reported in Supplementary Note 11.

#### Gene and genome duplications

Recently, a transcriptome-based study found evidence for putative ancient gene and genome duplications in hexapods, including potential WGD events in caddisflies [23], suggesting that duplication events could be responsible for some genome size variation in Trichoptera. We investigated whether this pattern persists with whole-genome data and found that the age distribution of duplications in 18 genomes was significantly different compared to the background rate of gene duplication (Supplementary Figs S161 and S162). To identify whether any significant peak is consistent with a potential WGD, we used mixture modeling to identify peaks in these gene age distributions, which recovered no obvious peak consistent with an ancient WGD. To further investigate potential WGD, we used Smudgeplot [73] to visualize the haplotype structure and to estimate ploidy of the genomes.

While Smudgeplot predicted most of the genomes to be diploid, 4 genomes with rather small genome sizes (230–650 Mb) were predicted to be tetraploid (*H. tenuis, Rhyacophila evoluta* RSS1 and HR1, and *P. elsis*). However, the Genomescope2 results indicate that these are highly homozygous samples. Low heterozygosity is a known confounder of Smudgeplot analyses [73] because it inflates the signal of duplication when compared to the low level of heterozygosity. We therefore interpret these 4 putative polyploids as artifacts of low heterozygosity in the analysis. Moreover, in some cases Smudgeplot results remain unclear because the estimated coverage (1n) differs from the sequencing coverage, peak coverage from the backmap.pl approach, and Genomescope2 coverage "kcov" (Supplementary Data File S1.8.) when automatically estimated from the data. The adjustment of the expected haploid coverage based on Genomescope2 kcov when running Smudgeplot suggests that some species might not be diploid (*Hesperophylax magnus*: octoploid, Supplementary Figs S96–S98; *Micrasema longulum* ML1: tripolid, Supplementary Figs Figs S116–S118; and *O. albicorne*: tetraploid, Supplementary Figs Figs S127–S129. However, further sequencing as well as karyotyping including chromosome counting would need to be done to confirm polyploidy in these species.

## Discussion

The drivers and evolutionary consequences of genome size evolution are a topic of ongoing debate. Several models have been proposed [9]. Some hypothesize genome size to be a (mal)adaptive trait by affecting phenotypic traits such as developmental/life history, body size, and other cell size–related effects [59, 74–76] reviewed in [9]. On the other hand, neutral theories suggest that DNA accumulation occurs only by genetic drift without selective pressures playing a major role in the accumulation or loss of DNA (the mutational hazard hypothesis [MHH] [24] and the mutational equilibrium hypothesis [MEH] [25]). The MHH only allows for small deleterious effects for the accumulation of extra DNA, which is accompanied by higher mutation rates in larger genomes [24], while the MEH focuses on the balance between insertions and deletions. It suggests that genome expansions arise by means of “bursts” of duplication events or TE activity and that genome shrinkage may be caused by a more constant rate of small deletions [25].

In this study, we observe that genome size varies ∼14-fold across the order Trichoptera, with lower genome size estimates in fixed retreat– and net-spinners and cocoon-builders compared to tube case–builders, and explore potential drivers of genome size evolution. Although recent genomic studies have shown evidence of bursts of gene duplication and gene family expansion during the evolution of hexapods [23, 77], the presence of ancient genome duplication events is still a subject of debate [78–80]. When computing haplotype structure and ploidy estimation, Smudgeplot suggested polyploidy in 3 species. However, karyotypes including chromosome counts are missing for these species because only very few have been reported for caddisflies in general [81–83]. We found no evidence of ancient WGD in the gene age distribution in our Trichoptera genomes, although we recognize that some of our current genome assemblies might be too fragmented to infer synteny. This does not mean that we can rule out that duplication events played a role in genome size evolution in Trichoptera in the past. The emergence of Pacific Biosciences HiFi genomes of caddisflies (e.g., Darwin Tree of Life Project is currently planning to sequence several caddisfly genomes [84]) will allow a deeper exploration of putative ancient duplication events in Trichoptera.

We found evidence that TE expansions (especially LINEs) were important drivers of genome size evolution in Trichoptera (Fig. 2, Supplementary Figs S146 and S147), which is consistent with MEH. The TE age distribution analyses suggested that the high abundance of LINEs was due to ongoing/recent activity occurring independently across cocoon- and particularly tube case–builders (Fig. 3, Supplementary Fig. S148). Thus, the shift to large genomes in these lineages does not appear to be due to a single (or a few) shared ancient events; rather they maintained dynamic turnover in composition of their large genomes. Mutational bias affecting pathways tied to TE regulation may affect insertion/deletion ratios and subsequently lead to lineage-specific shifts in genome size equilibrium [85]. Such changes may be stochastic (e.g., due to drift) or linked to traits that evolve on independent trajectories as lineages diverge and are thereby constrained by phylogeny. Ecological factors, demographic history, and effective population size can further affect mutation rates. For example, environmental stress can trigger bursts of TE activity and elevated mutation rates [86–88], driving lineages that occupy niche space with frequent exposure to environmental stress toward increased TE loads and larger genomes. Similarly, lineages with small effective population sizes or that are prone to population bottlenecks may have higher mutation rates and/or reduced efficacy of natural selection, which would otherwise purge mildly deleterious TE load.

Although our study is not designed to pinpoint specific forces maintaining large genomes in some lineages, the pattern that we observe in the distribution of genome size (i.e., lower genome size estimates in fixed retreat– and net-spinners and cocoon-builders compared to tube case–builders) leads us to hypothesize that ecological factors may play a role in genome size evolution in the order. The 3 focal groups discussed here exhibit markedly different ecological strategies. Larvae of fixed retreat– and net-spinners generally occupy relatively narrow niche space in oxygen-rich flowing-water (mostly stream/river) environments where they rely on water currents to bring food materials to their filter nets. The evolutionary innovation of tube-case making is thought to have enabled tube case–builders to occupy a much greater diversity of ecological niche space by allowing them to obtain oxygen in lentic (e.g., pond, lake, marsh) environments, which are much more variable in temperature and oxygen availability than lotic environments [89, 90]. This environmental instability is greater over short (daily, seasonal) and long time scales (centuries, millennia) [91]. It is thus plausible that these tube case–building lineages experience greater environmental stress and less stable population demographics that could lead to both more frequent TE bursts and reduced efficacy of natural selection in purging deleterious effects of TE expansions as described above [24, 25].

We show that TE expansions (especially LINEs and DNA transposons) in cocoon- and tube case–builders have a major impact on protein-coding gene regions (Fig. 4). These TE-gene associations show a linear relationship with increasing genome size. This trend is particularly pronounced among tube case–builders, in which TE-associated BUSCOs comprise a mean of 21.4% of total BUSCO genes (compared with 6.2% in annulipalpians). This finding corroborates other studies highlighting the role of TEs as drivers of rapid genome evolution [92–95] and highlights their impact on genomic regions that have potential effects on phenotypes. Questions remain as to what evolutionary roles such changes in genic regions may play. In general, TE insertions are considered to have deleterious effects on their host's fitness activity [96, 97]. They are known to “interrupt” genes [34], pose a risk of ectopic recombination that can lead to genome rearrangements [32, 35, 98], and have epigenetic effects on neighboring sequences [55, 99]. Therefore, purifying selection keeps TEs at low frequencies [34]. However, there is growing evidence that TE activity can also be a critical source of new genetic variation driving diversification via chromosomal rearrangements and transposition events, which can result in mutations [100], including examples of co-option [101]; e.g., recent research in mammals has shown that DNA transposon fragments can be co-opted to form regulatory networks with genome-wide effects on gene expression [45].

Ecological correlates with genome size are widely discussed in other taxa [61, 102–105]. Caddisflies and other diverse insect lineages that feature various microhabitat specializations, feeding modes, and/or the use of silk represent evolutionary replicates with contrasting traits and dynamic genome size evolution. They thus have high potential as models for understanding links between ecology and the evolution of REs, genomes, and phenotypes. Our study lays a foundation for future work in caddisflies that investigates the potential impact of TE expansions on phenotypes and tests for evidence of co-option/adaptive impacts of TE-rich genomes against a null of neutral or slightly deleterious effects.

### Potential implications

Many open questions remain as to the causes and consequences of genome size evolution. As we move forward in an era where genome assemblies are attainable for historically intractable organisms (e.g., due to constraints given large genome sizes, tissue limitations, no close reference available) we can leverage new model systems spanning a greater diversity of life to understand how genomes evolve. Here, we provide genomic resources and new genome size estimates across lineages of an underrepresented insect order that spans major variation in genome size. These data allowed us to study genome size evolution in a phylogenetic framework to reveal lineage-specific patterns in which genome size correlates strongly with phylogeny and ecological characteristics within lineages. We find that large genomes dominate lineages with a wider range of ecological variation and that ongoing recent TE activity seems to maintain large genomes in these lineages. This leads us to hypothesize that ecological factors may be linked to genome size evolution in this group. The future directions spawned by our findings highlight the potential for using Trichoptera and other diverse insect groups to understand the link between ecological and genomic diversity, a link that has been challenging to study with past models [9].

We also show that TE expansions are associated with increasing genome size and have an effect on protein-coding regions. These effects have been greatest in the most species-rich and ecologically diverse caddisfly clades. While TEs are generally considered to have deleterious effects on their host's fitness activity, their roles can also be neutral or even adaptive. TE activity can be a critical source of new genetic variation and thus an important driver for diversification. Caddisflies and potentially other non-model insect groups are excellent models to test these contrasting hypotheses, as well as the potential impact of TEs on phenotypes. Using these models, especially with respect to the increasing emergence of high-quality insect genomes [106], will allow researchers to identify recurring patterns in TE dynamics and investigate their evolutionary implications across diverse clades.

## Methods

### DNA extraction, library preparation, sequencing, and sequence read processing

We extracted high molecular weight genomic DNA (gDNA) from 17 individuals (15 species) of caddisfly larvae (for sampling information, see Supplementary Data File S1.1) after removing the intestinal tracts using a salting-out protocol adapted from [107] as described in Supplementary Note 1. We generated gDNA libraries for a low-cost high-contiguity sequencing strategy, i.e., using a combination of short (Illumina) and long-read (Nanopore or Pacific Biosciences) technologies as described in Supplementary Note 2. For details on sequencing coverage for each specimen see Supplementary Data File S1.3.

### *De novo* genome assembly, annotation, and quality assessment

We applied different assembly strategies for different datasets. First, we applied a long-read assembly method using wtdbg2 v2.4 (WTDBG, RRID:SCR_017225) [108] with subsequent short-read polishing with Pilon v1.22 (Pilon, RRID:SCR_014731) [109] because this method revealed good results in previous *de novo* assemblies in caddisflies [63]. In cases where this pipeline did not meet the expected quality regarding contiguity and BUSCO completeness, we applied *de novo* hybrid assembly approaches of MaSuRCA v.3.1.1 (MaSuRCA, RRID:SCR_010691) [110] (Supplementary Note 3). Illumina-only data were assembled with SPAdes (SPAdes, RRID:SCR_000131) [111] (explained in Supplementary Note 3). Prior to annotating the individual genomes with MAKER2 v2.31.10 [112, 113] we used RepeatModeler v2.0 (RepeatModeler, RRID:SCR_015027) and RepeatMasker v4.1.0 (RepeatMasker, RRID:SCR_012954) to identify species-specific REs in each of the assemblies, relative to RepBase libraries v20181026 [114]. Transcriptome evidence for the annotation of the individual genomes included their species-specific or closely related *de novo* transcriptome provided by 1KITE [115, 116] (Suplementary Data File S1.9) or downloaded from Genbank as well as the complementary DNA and protein models from *Stenopsyche tienmushanensis* [117] and *Bombyx mori* (AR102, GenBank accession ID No. GCF_000151625.1). Additional protein evidence included the uniprot-sprot database (downloaded 25 September2018). We masked repeats on the basis of species-specific files produced by RepeatModeler. For *ab initio* gene prediction, species-specific AUGUSTUS gene prediction models, as well as *B. mori* SNAP gene models, were provided to MAKER. The EvidenceModeler (EVidenceModeler, RRID:SCR_014659) [118] and tRNAscan [119] options in MAKER were used to produce a weighted consensus gene structure and to identify transfer RNA genes. MAKER default options were used for BLASTN (BLASTN, RRID:SCR_001598), BLASTX (BLASTX, RRID:SCR_001653), and TBLASTX (TBLASTX, RRID:SCR_011823) searches. Two assemblies (*Agapetus fuscipens* GL3 and *M. longulum* ML1) were not annotated because of their low contiguity. All protein sequences were assigned putative names by BlastP Protein–Protein BLAST 2.2.30+ searches [120] and were functionally annotated using command line Blast2Go v1.3.3 (Blast2GO, RRID:SCR_005828) [121] (see Supplementary Note 4, Supplementary Figs S1–S30).

We calculated assembly statistics with QUAST v5.0.2 (QUAST, RRID:SCR_001228) [52] and examined completeness with BUSCO v5 (BUSCO, RRID:SCR_015008) [53, 54] using the Endopterygota odb10 dataset with the options "–long, –m = genome". A summary of the assembly statistics and BUSCO completeness is given in Table 1. The final genome assemblies and annotations were screened and filtered for potential contaminations with taxon-annotated GC-coverage (TAGC) plots using BlobTools v1.0 (Blobtools, RRID:SCR_017618) [122]. Details and blobplots are given in Supplementary Note 5 and Supplementary Figs S31–S47.

### Species tree reconstruction

We used the single-copy orthologs resulting from a BUSCOv3.0.2 analysis (with the Endopterygota odb9 dataset and options –long, –m = genome and –sp = fly) to generate a species tree. We first combined single-copy ortholog amino acid files from each species into a single FASTA for each ortholog. We then aligned them with the MAFFT L-INS-i algorithm [123]. We selected amino acid substitution models for each ortholog using ModelFinder (option -m mfp, [124] in IQtree v.2.0.6 [125] and estimated a maximum likelihood tree with 1,000 ultrafast bootstrap replicates [126] with the BNNI correction (option -bb 1000 -bnni). We combined the best maximum likelihood tree from each gene for species tree analysis in ASTRAL-III [127]. A locus tree was inferred using the alignment file (-s) and the partition file (-S) with the settings –prefix loci and -T AUTO in IQtree. Gene and site concordance factors were calculated with IQTree using the species tree (-t), the locus tree (–gcf), and the alignment file (-s) with 100 quartets for computing the site concordance factors (–scf 100) and –prefix concord for computing the gene concordance factors. We visualized the trees using FigTree v.1.4.4 (FigTree, RRID:SCR_008515).

### Genome size estimations and genome profiling

Genome size estimates of 27 species were conducted using FCM according to Otto [56] using *Lycopersicon esculentum* cv. Stupické polnítyčkové rané (2C  =  1.96 pg [57]) as internal standard and propidium iodine as stain (see Suplementary Data File S1.6). Additionally, we used trimmed, contamination-filtered short-read data (see Supplementary Note 2) to conduct genome profiling (estimation of major genome characteristics such as size, heterozygosity, and repetitiveness) using a *k-*mer distribution–based method (GenomeScope 2.0, RRID:SCR_017014) [73]. Genome scope profiles are available online (see links to Genomescope 2 in Suplementary Data File S1.4). In addition, we applied a second sequencing-based method for genome size estimates, which uses the back-mapping rate of sequenced reads to the assembly and coverage distribution (backmap.pl v0.1 [65], see Suplementary Data File S1.5). Details of all 3 methods are described in Supplementary Note 7. Coverage distribution per position and genome size estimate from backmap.pl are shown in Supplementary Figs S49–S72. We assessed the congruence among the 3 quantitative methods of measurement (Genomescope2, Backmap.pl, and FCM) with Bland-Altman-Plots using the function BlandAltmanLeh::bland.altman.plot in ggplot2 [60] in RStudio [128] (Supplementary Note 8, Supplementary Fig. S73).

### Repeat dynamics

#### Repeat abundance and classification

We identified and classified REs in the genome assemblies of each species using RepeatModeler2.0 [129]. We annotated repeats in the contamination-filtered assemblies with RepeatMasker 4.1.0 (RepeatMasker, RRID:SCR_012954 using the custom repeat libraries generated from RepeatModeler2 for each respective assembly with the search engine set to “ncbi” and using the -xsmall option. We converted the softmasked assembly resulting from the first RepeatMasker round into a hardmasked assembly using the lc2n.py script [130]. Finally, we reran RepeatMasker on the hard-masked genome with RepeatMasker's internal arthropod repeat library using -species “Arthropoda.” We then merged RepeatMasker output tables from both runs by parsing them with a custom-made script (RM_table_parser_families_.py [131]) and then combined the resulting data columns for the two runs in Excel.

We also estimated RE abundance and composition using RepeatExplorer2 [132, 133] and dnaPipeTE v.1.3.1 [134]. These reference-free approaches quantify repeats directly from unassembled short-read data. These analyses allowed us to test for general consistency of patterns with our assembly-based approach described above and to test for the presence of abundant repeat categories such as satellite DNAs, which can comprise large fractions of genomes yet can be prone to poor representation in the genome assembly. Prior to analysis, we normalized contamination-filtered (see Supplementary Note 2) input datasets to 0.5× coverage using RepeatProfiler [135] and seqtk [136] and then ran RepeatExplorer2 clustering with the Metazoa 3.0 database specified for annotation (Supplementary Fig. S146) and dnaPipeTE with the -RM_lib flag set to the Repbase v20170127 repeat library (Supplementary Fig. S147).

#### TE age distribution analysis

We further characterized RE dynamics in Trichoptera by analyzing TE landscapes, which show relative age differences among TE sequences and their genomic abundance. We used these analyses to test whether abundance patterns of specific TEs are driven by shared ancient proliferation events or more recent/ongoing activity of the respective TEs. For example, if shared ancient proliferation is driving abundance patterns of a given TE, the majority of its copies would show moderate to high sequence divergence (e.g., >10% pairwise divergence). In contrast, if abundance patterns are driven by recent/ongoing activity of a given TE, we would expect the majority of its sequences to show low sequence divergence (e.g., 0–10%). We generated TE age distribution plots using dnaPipeTE v1.3.1 [134] with genomic coverage for each species sampled to 0.5× prior to analysis and the -RM_lib flag set to the Repbase v20170127 repeat library (Supplementary Fig. S148).

#### TE sequence associations with protein-coding genes

We analyzed BUSCO genes (obtained from a BUSCOv3.0.2 analysis with the Endopterygota odb9 dataset and options –long, –m = genome and –sp = fly) for all species to quantify the abundance of TE-associated BUSCOs across samples and investigated associations between TEs and genic sequences in Trichoptera lineages by quantifying the abundance of TE-associated BUSCO genes (for presence and absence of TE-associated BUSCOs see Supplementary Fig. S149, Supplementary Data File S2.4). This analysis also allowed us to quantify shifts in associations between TEs and genic regions across Trichoptera lineages with varying repeat abundance. We identified BUSCO genes with high-coverage sequence regions based on coverage profiles and quantified their genomic abundance by using each TE-associated BUSCO as a query in a BLAST search against their respective genome assembly. We then conducted intersect analysis for all unique BUSCO hits from high-coverage sequences to determine whether these were annotated as TEs. We calculated the total number of bases in filtered BLAST after subtracting the number of bases at the locus belonging to all “complete” BUSCO genes and categorized high-coverage sequence regions in BUSCO genes based on their annotation status and repeat classification using custom scripts [131]. We plotted the number of the high-coverage BUSCO sequence regions belonging to RE categories (i.e., classes and subclasses) alongside plots of the relative genomic abundance of each respective category. In addition, we investigated BUSCO genes with regions of high coverage by pairwise alignments. Specifically, we visualized alignments of BUSCOs with high-coverage sequence regions (i.e., the “inflated species”) alongside orthologous BUSCOS that lack such regions taken from closely related species (i.e., the “reference” species). We further tested this prediction by taking the set of BUSCOs that only exhibited high-coverage regions in the inflated species and contrasted results of the two BLAST searches followed by an intersect analysis. A detailed description of this method is provided in Supplementary Note 11.

### Gene and genome duplications

#### Inference of WGDs from gene age distributions

To recover signal from potential WGDs, for each genome, we used the DupPipe pipeline to construct gene families and estimate the age distribution of gene duplications [137, 138]. We translated DNA sequences and identified open reading frames (ORFs) by comparing the Genewise [139] alignment to the best‐hit protein from a collection of proteins from 24 metazoan genomes from Metazome v3.0. For all DupPipe runs, we used protein‐guided DNA alignments to align our nucleic acid sequences while maintaining the ORFs. We estimated synonymous divergence (*K*_s_) using PAML with the F3×4 model [140] for each node in the gene family phylogenies (Supplementary Data File S1.10). We first identified taxa with potential WGDs by comparing their paralog ages to a simulated null distribution without ancient WGDs using a K-S goodness-of-fit test [141]. We then used mixture modeling to identify any significant peaks consistent with a potential WGD and to estimate their median paralog *K*_s_ values. Significant peaks were identified using a likelihood ratio test in the boot.comp function of the package mixtools in R [142].

#### Visualization of genome structure to estimate ploidy using Smudgeplots

We visualized the genome structure and estimated ploidy levels with Smudgeplot. For this purpose, we extracted genomic *k*-mers from *k*-mer counts produced with jellyfish (as described in “Genome size estimations and genome profiling”) using “jellyfish dump” with coverage thresholds previously estimated from *k*-mer histograms using the smudgeplot.py script. We computed the set of *k*-mer pairs with the Smudgeplot tool hetkmers. After generating the list of *k-*mer pair coverages, we generated smudgeplots using the coverage of the *k*-mer pairs and the “plot” tool within Smudgeplot. Ploidy, as well as the haploid *k*-mer coverage, was estimated directly from the data and compared to the estimated kcov reported by Genomescope2, sequencing coverage (and sequencing-based kcov), and peak coverage from the backmap.pl approach (see Supplementary Data File S1.8). When the haploid *k*-mer coverage estimated by Smudgeplot was inconsistent with the kcov observed by Genomescope2, it was manually adjusted using -n in smudgeplot.py plot. Details of the method and smudgeplots are given in Supplementary Note 9 and Supplementary Figs S74–145.

## Additional Files

**Supplementary Data File S1.1**. Sample information.

**Supplementary Data File S1.2**. Assembly statistics.

**Supplementary Data File S1.3**. Sequencing coverages.

**Supplementary Data File S1.4**. GenomeScope2 results.

**Supplementary Data File S1.5**. Backmap.pl results.

**Supplementary Data File S1.6**. Flow cytometry results.

**Supplementary Data File S1.7**. Genome size summary.

**Supplementary Data File S1.8**. Comparison coverage.

**Supplementary Data File S1.9**. List of transcriptomes.

**Supplementary Data File S1.10**. Paths to final Ks files.

**Supplementary Data File S2.1**. Assembly based repeat summary.

**Supplementary Data File S2.2**. RepeatExplorer summary.

**Supplementary Data File S2.3**. dnapipeTE_Results.

**Supplementary Data File S2.4**. TE-associated BUSCOs per Species.

**Supplementary Data File S2.5**. Summary of intersect analysis.

**Supplementary Data File S2.6**. Species pair tests.

**Supplementary Material.docx**

**Supplementary Note 1**. DNA extraction.

**Supplementary Note 2**. Sequencing strategies.

**Supplementary Note 3**. Assembly strategies.

**Supplementary Note 4**. Functional annotation of protein coding genes.

**Supplementary Note 5**. Contamination filtering.

**Supplementary Note 6:** Caddisfly silk usage.

**Supplementary Note 7:** Genome size estimations and genome profiling.

**Supplementary Note 8:** Bland-Altman-Plots.

**Supplementary Note 9:** Visualization of genome structure to estimate ploidy using smudgeplots.

**Supplementary Note 10:** Repeat abundance and classification based on reference-free analyses.

**Supplementary Note 11:** TE sequence association with protein-coding genes.

**Supplementary Note 12:** TE-associated BUSCOs

**Supplementary Figure S1**. Blast2GO Annotation Results of Drusus annulatus.

**Supplementary Figure S2**. Blast2GO Functional Annotation for Drusus annulatus.

**Supplementary Figure S3**. Blast2GO Annotation Results of Agraylea sexmaculata.

**Supplementary Figure S4**. Blast2GO Functional Annotation for Agraylea sexmaculata.

**Supplementary Figure S5**. Blast2GO Annotation Results of Glossosoma conforme.

**Supplementary Figure S6**. Blast2GO Functional Annotation for Glossosoma conforme.

**Supplementary Figure S7**. Blast2GO Annotation Results of Halesus radiatus.

**Supplementary Figure S8**. Blast2GO Functional Annotation for Halesus radiatus.

**Supplementary Figure S9**. Blast2GO Annotation Results of Himalopsyche phryganeae.

**Supplementary Figure S10**. Blast2GO Functional Annotation for Himalopsyche phryganeae.

**Supplementary Figure S11**. Blast2GO Annotation Results of Lepidostoma basale.

**Supplementary Figure S12**. Blast2GO Functional Annotation for Lepidostoma basale.

**Supplementary Figure S13**. Blast2GO Annotation Results of Micrasema longulum ML3.

**Supplementary Figure S14**. Blast2GO Functional Annotation for Micrasema longulum ML3.

**Supplementary Figure S15**. Blast2GO Annotation Results of Micrasema minimum.

**Supplementary Figure S16**. Blast2GO Functional Annotation for Micrasema minimum.

**Supplementary Figure S17**. Blast2GO Annotation Results of Micropterna sequax.

**Supplementary Figure S18**. Blast2GO Functional Annotation for Micropterna sequax.

**Supplementary Figure S19**. Blast2GO Annotation Results of Odontocerum albicorne.

**Supplementary Figure S20**. Blast2GO Functional Annotation for Odontocerum albicorne.

**Supplementary Figure S21**. Blast2GO Annotation Results of Parapsyche elsis.

**Supplementary Figure S22**. Blast2GO Functional Annotation for Parapsyche elsis.

**Supplementary Figure S23**. Blast2GO Annotation Results of Philopotamus ludiferatus.

**Supplementary Figure S24**. Blast2GO Functional Annotation for Philopotamus ludiferatus.

**Supplementary Figure S25**. Blast2GO Annotation Results of Rhyacophila brunneae.

**Supplementary Figure S26**. Blast2GO Functional Annotation for Rhyacophila brunneae.

**Supplementary Figure S27**. Blast2GO Annotation Results of Rhyacophila evoluta HR1.

**Supplementary Figure S28**. Blast2GO Functional Annotation for Rhyacophila evoluta HR1.

**Supplementary Figure S29**. Blast2GO Annotation Results of Rhyacophila evoluta RSS1.

**Supplementary Figure S30**. Blast2GO Functional Annotation for Rhyacophila evoluta RSS1.

**Supplementary Figure S31**. Taxon-annotated GC-coverage (TAGC) plots of Agapetus fuscipens GL3 genome assembly.

**Supplementary Figure S32**. Taxon-annotated GC-coverage (TAGC) plots of Agraylea sexmaculata AS19 genome assembly.

**Supplementary Figure S32**. Taxon-annotated GC-coverage (TAGC) plots of Drusus annulatus AC1 genome assembly.

**Supplementary Figure S34**. Taxon-annotated GC-coverage (TAGC) plots of Glossosoma conforme G1.

**Supplementary Figure S35**. Taxon-annotated GC-coverage (TAGC) plots of Halesus radiatus.

**Supplementary Figure S36**. Taxon-annotated GC-coverage (TAGC) plots of Himalopsyche phryganeae.

**Supplementary Figure S37**. Taxon-annotated GC-coverage (TAGC) plots of Lepidostoma basale.

**Supplementary Figure S38**. Taxon-annotated GC-coverage (TAGC) plots of Micrasema longulum ML1.

**Supplementary Figure S39**. Taxon-annotated GC-coverage (TAGC) plots of Micrasema longulum ML3.

**Supplementary Figure S40**. Taxon-annotated GC-coverage (TAGC) plots of Micrasema minimum.

**Supplementary Figure S41**. Taxon-annotated GC-coverage (TAGC) plots of Micropterna sequax.

**Supplementary Figure S42**. Taxon-annotated GC-coverage (TAGC) plots of Odontocerum albicorne.

**Supplementary Figure S43**. Taxon-annotated GC-coverage (TAGC) plots of Parapsyche elsis.

**Supplementary Figure S44**. Taxon-annotated GC-coverage (TAGC) plots of Philopotamus ludiferatus.

**Supplementary Figure S45**. Taxon-annotated GC-coverage (TAGC) plots of Rhyacophila brunneae.

**Supplementary Figure S46**. Taxon-annotated GC-coverage (TAGC) plots of Rhyacophila evoluta HR1.

**Supplementary Figure S47**. Taxon-annotated GC-coverage (TAGC) plots of Rhyacophila evoluta RSS1.

**Supplementary Figure S48**. Phylogenetic relationships derived from ASTRAL-III analyses using single BUSCO genes.

**Supplementary Figure S49**. Agapetus fuscipens: Coverage distribution per position and genome size estimate from backmap.pl.

**Supplementary Figure S50**. Agraylea sexmaculata: Coverage distribution per position and genome size estimate from backmap.pl.

**Supplementary Figure S51**. Agrypnia vestita. Coverage distribution per position and genome size estimate from backmap.pl.

**Supplementary Figure S52**. Drusus annulatus: Coverage distribution per position and genome size estimate from backmap.pl.

**Supplementary Figure S53**. Glossosoma conforme G1: Coverage distribution per position and genome size estimate from backmap.pl.

**Supplementary Figure S54**. Glossosoma conforme Glo: Coverage distribution per position and genome size estimate from backmap.pl.

**Supplementary Figure S56**. Halesus radiatus. Coverage distribution per position and genome size estimate from backmap.pl.

**Supplementary Figure S57**. Hesperophylax magnus: Coverage distribution per position and genome size estimate from backmap.pl.

**Supplementary Figure S58**. Himalopsyche phryganeae: Coverage distribution per position and genome size estimate from backmap.pl.

**Supplementary Figure S59**. Lepidostoma basale: Coverage distribution per position and genome size estimate from backmap.pl.

**Supplementary Figure S61**. Micrasema longulum ML1: Coverage distribution per position and genome size estimate from backmap.pl.

**Supplementary Figure S62**. Micrasema longulum ML3: Coverage distribution per position and genome size estimate from backmap.pl.

**Supplementary Figure S63**. Micrasema minimum: Coverage distribution per position and genome size estimate from backmap.pl.

**Supplementary Figure S64**. Micropterna sequax: Coverage distribution per position and genome size estimate from backmap.pl.

**Supplementary Figure S65**. Odontocerum albicorne: Coverage distribution per position and genome size estimate from backmap.pl.

**Supplementary Figure S66**. Parapsyche elsis: Coverage distribution per position and genome size estimate from backmap.pl.

**Supplementary Figure S67**. Philopotamus ludificatus: Coverage distribution per position and genome size estimate from backmap.pl.

**Supplementary Figure S68**. Rhyacophila brunnea: Coverage distribution per position and genome size estimate from backmap.pl.

**Supplementary Figure S69**. Rhyacophila evoluta HR1: Coverage distribution per position and genome size estimate from backmap.pl.

**Supplementary Figure S70**. Rhyacophila evoluta Rss1: Coverage distribution per position and genome size estimate from backmap.pl.

**Supplementary Figure S71**. Sericostoma sp.: Coverage distribution per position and genome size estimate from backmap.pl.

**Supplementary Figure S72**. Stenopsyche tienhuanesis: Coverage distribution per position and genome size estimate from backmap.pl.

**Supplementary Figure S73**. Bland-Altman-Plots to test the comparability of agreement between the three quantitative methods of genome size measurement (Genomescope2, Backmap.pl and FCM; supplementary Note 7).

**Supplementary Figure S74**. Smudgeplot for Agapetus fuscipens GL3 on the linear scale.

.**Supplementary Figure S75**. Smudgeplot for Agapetus fuscipens GL3 on the log scale.

**Supplementary Figure S76**. Smudgeplot for Agraylea sexmaculata AS19 on the linear scale.

**Supplementary Figure S77**. Smudgeplot for Agraylea sexmaculata AS19 on the log scale.

**Supplementary Figure S78**. Smudgeplot for Agrypnia vestiva on the linear scale.

**Supplementary Figure S79**. Smudgeplot for Agrypnia vestiva on the log scale.

**Supplementary Figure S80**. Smudgeplot for Drusus annulatus on the linear scale.

**Supplementary Figure S81**. Smudgeplot for Drusus annulatus AC1 on the log scale.

**Supplementary Figure S82**. Smudgeplot for Glossosma conforme G1 on the linear scale.

**Supplementary Figure S83**. Smudgeplot for Glossosma conforme G1 on the log scale.

**Supplementary Figure S84**. Smudgeplot for Glossosma conforme Glo on the linear scale.

**Supplementary Figure S85**. Smudgeplot for Glossosma conforme Glo on the log scale.

**Supplementary Figure S86**. Smudgeplot for Halesus radiatus L2 on the linear scale.

**Supplementary Figure S87**. Smudgeplot for Halesus radiatus L2 on the log scale .

**Supplementary Figure S88**. Smudgeplot for Himalopsyche phryganeae on the linear scale.

**Supplementary Figure S89**. Smudgeplot for Himalopsyche phryganeae on the log scale.

**Supplementary Figure S90**. Smudgeplot for Himalopsyche phryganeae on the linear scale, 1n was manually adjusted using -n based on the Genomescope2 kcov (-n=53).

**Supplementary Figure S91**. Smudgeplot for Himalopsyche phryganeae on the log scale, 1n was manually adjusted using -n based on the Genomescope2 kcov (-n=53).

**Supplementary Figure S92**. Smudgeplot for Himalopsyche phryganeae on the linear scale, 1n was manually adjusted using -n based on the kcov using the sequencing coverage (-n=61).

**Supplementary Figure S93**. Smudgeplot for Himalopsyche phryganeae on the log scale, 1n was manually adjusted using -n based on the kcov using the sequencing coverage (-n=61).

**Supplementary Figure S94**. Smudgeplot for Hesperophylax magnus on the linear scale.

**Supplementary Figure S95**. Smudgeplot for Hesperophylax magnus on the log scale.

**Supplementary Figure S96**. Smudgeplot for Hesperophylax magnus on the linear scale, 1n was manually adjusted using -n based on the Genomescope2 kcov (-n=21).

**Supplementary Figure S97**. Smudgeplot for Hesperophylax magnus on the log scale, 1n was manually adjusted using -n based on the Genomescope2 kcov (-n=21).

**Supplementary Figure S98**. Smudgeplot for Hesperophylax magnus on the linear scale, 1n was manually adjusted using -n based on the kcov using the sequencing coverage (-n=25).

**Supplementary Figure S99**. Smudgeplot for Hesperophylax magnus on the log scale, 1n was manually adjusted using -n based on the kcov using the sequencing coverage (-n=25).

**Supplementary Figure S100**. Smudgeplot for Hydropsyche tenuis on the linear scale.

**Supplementary Figure S101**. Smudgeplot for Hydropsyche tenuis on the log scale.

**Supplementary Figure S102**. Smudgeplot for Lepidostoma basale LB1 on the linear scale.

**Supplementary Figure S103**. Smudgeplot for Lepidostoma basale LB1 on the log scale.

**Supplementary Figure S104**. Smudgeplot for Lepidostoma basale on the linear scale, 1n was manually adjusted using -n based on the Genomescope2 kcov (-n=36).

**Supplementary Figure S105**. Smudgeplot for Lepidostoma basale on the log scale, 1n was manually adjusted using -n based on the Genomescope2 kcov (-n=36).

**Supplementary Figure S106**. Smudgeplot for Lepidostoma basale on the linear scale, 1n was manually adjusted using -n based on the kcov using the sequencing coverage (-n=48).

**Supplementary Figure S107**. Smudgeplot for Lepidostoma basale on the log scale, 1n was manually adjusted using -n based on the kcov using the sequencing coverage (-n=48).

**Supplementary Figure S108**. Smudgeplot for Micrasema longulum ML3 on the linear scale.

**Supplementary Figure S109**. Smudgeplot for Micrasema longulum ML3 on the log scale.

**Supplementary Figure S110**. Smudgeplot for Micrasema longulum ML3 on the linear scale, 1n was manually adjusted using -n based on the Genomescope2 kcov (-n=43).

**Supplementary Figure S111**. Smudgeplot for Micrasema longulum ML3 on the log scale, 1n was manually adjusted using -n based on the Genomescope2 kcov (-n=43).

**Supplementary Figure S112**. Smudgeplot for Micrasema longulum ML3 on the linear scale, 1n was manually adjusted using -n based on the kcov using the sequencing coverage (-n=48).

**Supplementary Figure S113**. Smudgeplot for Micrasema longulum ML3 on the log scale, 1n was manually adjusted using -n based on the kcov using the sequencing coverage (-n=48).

**Supplementary Figure S114**. Smudgeplot for Micrasema longulum ML1 on the linear scale.

**Supplementary Figure S115**. Smudgeplot for Micrasema longulum ML1 on the log scale.

**Supplementary Figure S116**. Smudgeplot for Micrasema longulum ML1 on the linear scale, 1n was manually adjusted using -n based on the Genomescope2 kcov (-n=39).

**Supplementary Figure S117**. Smudgeplot for Micrasema longulum ML1 on the log scale, 1n was manually adjusted using -n based on the Genomescope2 kcov (-n=39).

**Supplementary Figure S118**. Smudgeplot for Micrasema longulum ML1 on the linear scale, 1n was manually adjusted using -n based on the kcov using the sequencing coverage (-n=45).

**Supplementary Figure S119**. Smudgeplot for Micrasema longulum ML1 on the log scale, 1n was manually adjusted using -n based on the kcov using the sequencing coverage (-n=45).

**Supplementary Figure S120**. Smudgeplot for Mcirasema minimum K05 on the linear scale.

**Supplementary Figure S121**. Smudgeplot for Micrasema minimum K05 on the log scale.

**Supplementary Figure S122**. Smudgeplot for Micropterna sequax AB8 on the linear scale.

**Supplementary Figure S123**. Smudgeplot for Micropterna sequax AB8 on the log scale.

**Supplementary Figure S124**. Smudgeplot for Odontocerum albicorne OD1 on the linear scale.

**Supplementary Figure S125**. Smudgeplot for Odontocerum albicorne OD1 on the linear scale.

**Supplementary Figure S126**. Smudgeplot for Odontocerum albicorne OD1 on the linear scale, 1n was manually adjusted using -n based on the kcov using the sequencing coverage (-n=20).

**Supplementary Figure S127**. Smudgeplot for Odontocerum albicorne OD1 on the log scale, 1n was manually adjusted using -n based on the kcov using the sequencing coverage (-n=20).

**Supplementary Figure S128**. Smudgeplot for Odontocerum albicorne OD1 on the linear scale, 1n was manually adjusted using -n based on the Genomescope2 kcov (-n=25).

**Supplementary Figure S129**. Smudgeplot for Odontocerum albicorne OD1 on the log scale, 1n was manually adjusted using -n based on the Genomescope2 kcov (-n=25).

**Supplementary Figure S130**. Smudgeplot for Parapsyche elsis on the linear scale.

**Supplementary Figure S131**. Smudgeplot for Parapsyche elsis on the linear scale.

**Supplementary Figure S132**. Smudgeplot for Philopotamus ludificatus Ph2 on the linear scale.

**Supplementary Figure S133**. Smudgeplot for Philopotamus ludificatus Ph2 on the log scale.

**Supplementary Figure S134**. Smudgeplot for Plectrocnemia conspersa on the linear scale.

**Supplementary Figure S135**. Smudgeplot for Plectrocnemia conspersa on the log scale.

**Supplementary Figure S136**. Smudgeplot for Rhyacophila brunneae on the linear scale.

**Supplementary Figure S137**. Smudgeplot for Rhyacophila brunneae on the log scale.

**Supplementary Figure S138**. Smudgeplot for Rhyacophila evoluta HR1 on the linear scale.

**Supplementary Figure S139**. Smudgeplot for Rhyacophila evoluta HR1 on the log scale.

**Supplementary Figure S140**. Smudgeplot for Rhyacophila evoluta RSS1 on the linear scale.

**Supplementary Figure S141**. Smudgeplot for Rhyacophila evoluta RSS1 on the log scale.

**Supplementary Figure S142**. Smudgeplot for Sericostoma sp. on the linear scale.

**Supplementary Figure S143**. Smudgeplot for Sericostoma sp. on the log scale.

**Supplementary Figure S144**. Smudgeplot for Stenopsyche on the linear scale.

**Supplementary Figure S145**. Smudgeplot for Stenopsyche on the log scale.

S**upplementary Figure S146**. Repeat abundance summary from Repeat-Explorer2 .

**Supplementary Figure S147**. Repeat abundance summary from dnaPipeTE.

**Supplementary Figure S148**. Transposable element age distribution landscapes.

**Supplementary Figure S149**. Presence and absence of TE-associated BUSCOs.

**Supplementary Figure S150**. Correlations between bases in TE-associated BUSCO BLAST hits and genomic abundance of repeat categories.

**Supplementary Figure S151**. BUSCO EOG090R0A7C in IGV.

**Supplementary Figure S152**. BUSCO EOG090R0A26 in IGV.

**Supplementary Figure S153**. BUSCO EOG090R0AIP in IGV.

**Supplementary Figure S154**. BUSCO EOG090R0AIP in IGV.

**Supplementary Figure S155**. BUSCO EOG090R0BAL in IGV.

**Supplementary Figure S156**. BUSCO EOG090R0BV8 in IGV.

**Supplementary Figure S157**. BUSCO EOG090R0D3M in IGV.

**Supplementary Figure S158**. BUSCO EOG090R0D5K in IGV.

**Supplementary Figure S159**. BUSCO EOG090R0DJA in IGV.

**Supplementary Figure S160**. BUSCO EOG090R0DQF in IGV.

**Supplementary Figure S161**. Inference of WGDs from gene age distributions KS2.

**Supplementary Figure S162**. Inference of WGDs from gene age distributions KS5

**Table S1:**Ten BUSCOs of Hesperophylax magnus, their location in the genome and the start and end of the highly covered region.

giac011_GIGA-D-21-00216_Original_Submission

giac011_GIGA-D-21-00216_Revision_1

giac011_GIGA-D-21-00216_Revision_2

giac011_Response_to_Reviewer_Comments_Revision_1

giac011_Response_to_Reviewer_Comments_Revision_2

giac011_Reviewer_1_Report_Original_Submission Julie Blommaert -- 8/23/2021 Reviewed

giac011_Reviewer_1_Report_Revision_1 Julie Blommaert -- 11/30/2021 Reviewed

giac011_Reviewer_2_Report_Original_Submission Gregg Thomas -- 8/27/2021 Reviewed

giac011_Supplemental_Files

## Data Availability

This project has been deposited at NCBI and is available under BioProject ID: PRJNA558902.

The datasets supporting the results of this article are available in the supplementary data files S1 and S2. All supporting data and materials are available in the *GigaScience* GigaDB database [143]. All custom-made scripts used in this study are available at [131].

## Abbreviations

bp: base pairs; BLAST: Basic Local Alignment Search Tool; BUSCO: Benchmarking Universal Single-Copy Orthologs; FCM: flow cytometry; Gb: gigabase pair; GO: gene ontology; kb: kilobase pairs; LINE: long interspersed nuclear element; LTR: long terminal repeat; MAFFT: Multiple Alignment using Fast Fourier Transform; MaSuRCA: Maryland Super-Read Celera Assembler; Mb: megabase pairs; MEH: mutational equilibrium hypothesis; MHH: mutational hazard hypothesis; NCBI: National Center for Biotechnology Information; ORF: open reading frame; RE: repetitive element; SINE: short interspersed nuclear element; SPAdes: St. Petersburg genome Assembler; TE: transposable element; WGD: whole-genome duplication.

## Conflicts if interest

The authors declare that they have no conflicts of interest

## Competing Interests

The authors declare that they have no competing interests.

## Funding

This work is a result of the LOEWE-Centre for Translational Biodiversity Genomics funded by the Hessen State Ministry of Higher Education, Research and the Arts (HMWK) that supported J.H. and S.U.P., as well as internal funds of Senckenberg Research Institute provided to J.P. J.S.S. was supported by an NSF Postdoctoral Research Fellowship in Biology (DBI-1811930) and an NIH General Medical Sciences award (R35GM119515) to A.M.L. Sequencing was, in part, supported by BYU start-up funds to P.B.F. and funds from the Army Research Office, Life Science Division (Award No. W911NF-13–1-0319) to R.J.S.

## Authors' Contributions

Conceptualization—J.H., J.S.S., P.B.F., S.U.P.; Data curation—J.H.; Formal Analysis—J.H., P.J.M., J.P., J.S.S., P.B.F., Z.L.; Funding acquisition—A.M.L., P.B.F., S.U.P., R.J.S.; Investigation—J.H., J.P., J.S.S., P.B.F., Z.L.; Methodology—A.M.L., J.S.S., J.P., J.V.S., P.B.F.; Project administration—S.U.P.; Resources—J.P., M.B., P.B.F., S.U.P.; Visualization—J.H., J.S.S.; Writing—original draft—J.H., J.S.S., P.B.F., Z.L.; Writing—review & editing—A.M.L., J.H., P.J.M., J.P., J.S.S., J.V.S., M.S.B., P.B.F., R.J.S., S.U.P., Z.L.
