# Supplementary material for: Genome size evolution in the diverse insect order Trichoptera
Source: Gigascience. 2022 Feb 25;11:giac011. doi: 10.1093/gigascience/giac011 (PMC8881205; doi:10.1093/gigascience/giac011)
Supplement: giac011_GIGA-D-21-00216_Original_Submission [file giac011_giga-d-21-00216_original_submission.pdf]

GigaScience

Genome size evolution in the diverse insect order Trichoptera

--Manuscript Draft--

**Manuscript Number:**	GIGA-D-21-00216
**Full Title:**	Genome size evolution in the diverse insect order Trichoptera
**Article Type:**	Research
**Funding Information:**	national science foundation (DBI-1811930) — Dr. John S. Sproul<br>national institutes of health (R35GM119515) — Dr. Amanda M. Larracuente<br>brigham young university — Dr. Paul B. Frandsen<br>hessen state ministry of higher education, research and the arts — Dr. Jacqueline Heckenhauer<br>hessisches ministerium für wissenschaft und kunst — Dr. Jacqueline Heckenhauer, Dr. Steffen U. Pauls<br>army research office (W911NF-13-1-0319) — Dr. Russell J. Stewart
**Abstract:**	Background<br><br>Genome size is implicated in form, function, and ecological success of a species. Two principally different mechanisms are proposed as major drivers of eukaryotic genome evolution and diversity: Polyploidy (i.e. whole genome duplication: WGD) or smaller duplication events and bursts in the activity of repetitive elements (RE). Here, we generated de novo genome assemblies of 17 caddisflies covering all major lineages of Trichoptera. Using these and previously sequenced genomes, we use caddisflies as a model for understanding genome size evolution in diverse insect lineages.<br><br>Results<br><br>We detect a ~14-fold variation in genome size across the order Trichoptera. While gene age distribution analyses found no evidence that ancient WGD played a major role in genome size variation in this group, we find strong evidence that transposable element (TE) expansions are the primary drivers of large caddisfly genome sizes. Using an innovative method to examine TEs associated with BUSCO genes, we find that TE expansions have a major impact on protein-coding gene regions with TE-gene associations showing a linear relationship with increasing genome size. Intriguingly, we find that expanded genomes and TE-gene associations preferentially evolved in caddisfly clades with a higher ecological diversity (i.e. various feeding modes, diversification in variable, less stable environments).<br><br>Conclusion<br><br>Our findings provide a platform to test hypotheses about the potential evolutionary roles of TE activity and TE-gene associations, particularly in groups with high species, ecological, and functional diversities.
**Corresponding Author:**	Jacqueline Heckenhauer, Ph.D.<br>LOEWE-TBG: LOEWE-Zentrum fur Translationale Biodiversitatsgenomik<br>Frankfurt am Main, Hesse GERMANY
**Corresponding Author Secondary Information:**	
**Corresponding Author's Institution:**	LOEWE-TBG: LOEWE-Zentrum fur Translationale Biodiversitatsgenomik
**Corresponding Author's Secondary Institution:**	
**First Author:**	Jacqueline Heckenhauer, Ph.D.

Powered by Editorial Manager® and ProduXion Manager® from Aries Systems Corporation

First Author Secondary Information:	
Order of Authors:	Jacqueline Heckenhauer, Ph.D.
	Paul B. Frandsen
	John S. Sproul
	Zheng Li
	Juraj Paule
	Amanda M. Larracuente
	Peter J. Maughan
	Michael S. Barker
	Julio V. Schneider
	Russell J. Stewart
	Steffen U. Pauls
Order of Authors Secondary Information:	
Additional Information:	
Question	Response
Are you submitting this manuscript to a special series or article collection?	No
**Experimental design and statistics**<br><br>Full details of the experimental design and statistical methods used should be given in the Methods section, as detailed in our Minimum Standards Reporting Checklist. Information essential to interpreting the data presented should be made available in the figure legends.<br><br>Have you included all the information requested in your manuscript?	Yes
**Resources**<br><br>A description of all resources used, including antibodies, cell lines, animals and software tools, with enough information to allow them to be uniquely identified, should be included in the Methods section. Authors are strongly encouraged to cite Research Resource Identifiers (RRIDs) for antibodies, model organisms and tools, where possible.	Yes

Have you included the information requested as detailed in our Minimum Standards Reporting Checklist?	
**Availability of data and materials**<br><br>All datasets and code on which the conclusions of the paper rely must be either included in your submission or deposited in publicly available repositories (where available and ethically appropriate), referencing such data using a unique identifier in the references and in the “Availability of Data and Materials” section of your manuscript.<br><br>Have you have met the above requirement as detailed in our Minimum Standards Reporting Checklist?	No
If not, please give reasons for any omissions below.<br><br>as follow-up to "**Availability of data and materials**<br><br>All datasets and code on which the conclusions of the paper rely must be either included in your submission or deposited in publicly available repositories (where available and ethically appropriate), referencing such data using a unique identifier in the references and in the “Availability of Data and Materials” section of your manuscript.<br><br>Have you have met the above requirement as detailed in our Minimum Standards Reporting Checklist?<br><br>"	Raw data and assemblies are available at NCBI under BioProject: PRJNA558902. Data supporting the results will be submitted to GigaDB and can be accessed under this link: https://app.box.com/folder/136985262731?v=trich-genomes

Powered by Editorial Manager® and ProduXion Manager® from Aries Systems Corporation

Main Text Manuscript Click here to access/download;Manuscript;maintext.docx.docx

Genome size evolution in the diverse insect order Trichoptera

Authors

Jacqueline Heckenhauer (jacqueline.heckenhauer@senckenberg.de)[1,2*], Paul B. Frandsen (paul_frandsen@byu.edu)[1,3,4], John S. Sproul (johnssproul@gmail.com)[5], Zheng Li (zheng.li@austin.utexas.edu)[6], Juraj Paule (juraj.paule@senckenberg.de)[7], Amanda M. Larracuente (alarracu@ur.rochester.edu)[5], Peter J. Maughan (jeff_maughan@byu.edu)[3], Michael S. Barker (msbarker@arizona.edu)[6], Julio V. Schneider (julio.schneider@senckenberg.de)[2], Russell J. Stewart (russell.stewart@utah.edu)[8], Steffen U. Pauls (steffen.pauls@senckenberg.de)[1,2,9]

Affiliations

[*]corresponding author

[1]LOEWE Centre for Translational Biodiversity Genomics (LOEWE-TBG), Frankfurt, Germany,

[2]Department of Terrestrial Zoology, Senckenberg Research Institute and Natural History Museum Frankfurt, Frankfurt, Germany

[3]Department of Plant & Wildlife Sciences, Brigham Young University, Provo, UT

[4]Data Science Lab, Smithsonian Institution, Washington, DC

[5]Department of Biology, University of Rochester, Rochester, NY, USA

[6]Department of Ecology and Evolutionary Biology, University of Arizona, Tucson, USA

[7]Department of Botany and Molecular Evolution, Senckenberg Research Institute and Natural History Museum Frankfurt, Frankfurt, Germany

[8]Department of Biomedical Engineering, University of Utah, Salt Lake City, UT

[9]Institute for Insect Biotechnology, Justus-Liebig-University, Gießen, Germany

Abstract

Background: Genome size is implicated in form, function, and ecological success of a species. Two principally different mechanisms are proposed as major drivers of eukaryotic genome evolution and diversity: Polyploidy (i.e. whole genome duplication: WGD) or smaller duplication events and bursts in the activity of repetitive elements (RE). Here, we generated *de novo* genome assemblies of 17 caddisflies covering all major lineages of Trichoptera. Using these and previously sequenced genomes, we use caddisflies as a model for understanding genome size evolution in diverse insect lineages.

Results: We detect a ~14-fold variation in genome size across the order Trichoptera. While gene age distribution analyses found no evidence that ancient WGD played a major role in genome size variation in this group, we find strong evidence that transposable element (TE) expansions are the primary drivers of large caddisfly genome sizes. Using an innovative method to examine TEs associated with BUSCO genes, we find that TE expansions have a major impact on protein-coding gene regions with TE-gene associations showing a linear relationship with increasing genome size. Intriguingly, we find that expanded genomes and TE-gene associations preferentially evolved in caddisfly clades with a higher ecological diversity (i.e. various feeding modes, diversification in variable, less stable environments).

Conclusion: Our findings provide a platform to test hypotheses about the potential evolutionary roles of TE activity and TE-gene associations, particularly in groups with high species, ecological, and functional diversities.

Key words:

biodiversity, *de novo* genome assembly, genomics, genomic diversity, genome duplication, genome size evolution, insects, repetitive elements, transposable elements, Trichoptera

Background

Genome size can vary widely among closely and distantly related species, though our knowledge is still scarce for many non-model groups. This is especially true for insects, which account for much of the earth's species diversity. To date 1,345 insect genome size estimates have been published, representing less than 0.15% of all described insect species (Gregory, 2005: Animal Genome Size Database: http://www.genomesize.com, last accessed 2021-04-30). Genome size in insects ranges 240-fold from 69 Mb in chironomid midges [1] to 16.5 Gb in the mountain grasshopper *Podisma pedestris* [2].

Genome size variation relates poorly to the number of coding genes or the complexity of the organism (C-value enigma, [3],[4],[5],[6]) and evolutionary drivers of genome size variation remain a topic of ongoing debate (e.g. [7], [8], [9], [10]). Two principally different mechanisms are proposed as primary drivers of eukaryotic genome size evolution: Whole genome duplication (WGD, i.e. polyploidy) or smaller duplication events and expansion of repetitive elements (REs, [5]). While WGD is ubiquitous in plant evolution, it has been regarded as the exception in animals [11], [12]. However, ancient WGD has been hypothesized to be an important driver of evolution of mollusks (e.g. [13]) amphibians (e.g. [14], [15], fish (e.g. [16], [17], [18]) and arthropods (e.g. [19], [20], [21]), including multiple putative ancient large-scale gene duplications within Trichoptera [22].

REs are the primary driver of genome size variation in many eukaryotic genomes [23], [24]. The two major categories of REs are tandem repeats (satellite DNA) and mobile transposable elements (TEs). TEs are classified into class I [retrotransposons: endogenous retroviruses (ERVs), related long terminal repeat (LTR) and non-LTR retrotransposons: SINEs (Short Interspersed Nuclear Elements), LINEs (Long Interspersed Nuclear Elements)] and class II elements (DNA transposons, [25]). In insects, the genomic proportion of TEs ranges from 1% in the antarctic midge *Belgica antarctica* [26] to 65% in the migratory locust *Locusta migratoria* [27]. Broad-scale analysis of TE abundance in insects suggests that some order-

specific signatures are present, however, major shifts in TE abundance are also common at shallow taxonomic levels [28], [29], including in Trichoptera [30]. The movement and proliferation of REs can have deleterious consequences on gene function and genome stability [31], [32], [33], [34], [35]. Moreover, repeat content and abundance can turn over rapidly even over short evolutionary time scales (reviewed in [36]). This rapid evolution has consequences for genome evolution and speciation, e.g. repeat divergence causes genetic incompatibilities between even closely related species [37]. However, TEs can also be sources of genomic innovation with selective advantages for the host [38], [39], [40], [41], [42], [43] and they can contribute to global changes in gene regulatory networks [44], [45], [46]. Investigating RE dynamics in diverse clades provides a powerful lens for understanding their capacity for contributing to genome function and evolution. In addition, studying REs in species-rich groups may be critical for understanding how environmental factors are linked to genome evolution in natural populations (see [47]).

With more than 16,500 species, caddisflies (Trichoptera) are among the most diverse of all aquatic insects [48]. Their species richness is reflective of their ecological diversity, including, e.g. microhabitat specialization, full array of feeding modes, and diverse use of underwater silk secretions [49], [50]. An initial comparison of six caddisfly species found wide genome size variation in Trichoptera (ranging from 230 Mbp to 1.4 Gbp). In that study, we hypothesized that the observed variation was correlated with caddisfly phylogeny and that TEs contributed to a suborder-specific increase of genome size [30].

Here, we present a multi-faceted analysis to investigate genome size evolution in the order Trichoptera, as an example for highly diversified non-model organisms. Specifically, we (i) estimated genome size for species across the order to explore phylogenetic patterns in the distribution of genome size variation in Trichoptera and (ii) generated 17 new Trichoptera genomes to analyze, in conjunction with 9 existing genomes, the causes (WGD, TE

expansions) of genome size variation in the evolution of caddisflies. Studying the genomic diversity of this highly diversified insect order adds new insights into drivers of genome size evolution with potential to shed light on how genome size is linked to form, function, and ecology.

Data Description

Genomic resources

Non-model insect clades are largely underrepresented in genomic research, especially aquatic insects [51], [52]. This makes studying broad trends of genome evolution, such as the evolution of genome size and the links between ecological diversification and genome evolution, very difficult. Only nine genomes were available for the order Trichoptera prior to this study. With this in mind, we combined long- and short-read sequencing technologies to generate 17 new *de novo* genome assemblies across a wide taxonomic range, covering all major lineages of Trichoptera. Details on sequencing coverage and assembly strategies are given in Data S1 and supplementary notes 1-4. To assess quality, we calculated assembly statistics with QUAST v5.0.2 [53], examined completeness with BUSCO v3.0.2 [54], [55] and screened for potential contamination with taxon-annotated GC-coverage (TAGC) plots using BlobTools v1.0 (94, supplementary Figs. S31-S47). The new genomes are of comparable or better quality than other Trichoptera genomes previously reported in terms of BUSCO completeness and contiguity (Table 1, Data S1). This study increases the number of assemblies in this order from nine to 26, nearly tripling the number of available caddisfly genomes and thus providing a valuable resource for studying genomic diversity across this ecologically diverse insect order. The annotation of these genomes predicted 6,413 to 12,927 proteins (Table 1). Most of the annotated proteins (94.4% - 98.8%) showed significant sequence similarity to entries in the NCBI nr database. GO Distributions were similar to previously annotated caddisfly genomes, i.e. the major biological processes were cellular and

metabolic processes. Catalytic activity was the largest subcategory in molecular function and the cell membrane subcategories were the largest cellular component (supplementary Figs. S1-S30). This project has been deposited at NCBI under BioProject ID: PRJNA558902. For accession numbers of individual assemblies see Table 1.

We downloaded existing Trichoptera genomes from GenBank (https://www.ncbi.nlm.nih.gov/genome/) or Lepbase (http://download.lepbase.org/v4/) and used these in conjunction with our newly generated genomes to analyze genome size evolution as explained in the following sections of this manuscript.

Flow cytometry

In addition to genomic resources, we used flow cytometry to detect genome size variation across the order. Our study increased the number of species with available flow cytometry-based genome size estimates from 4 [57] to 31. Estimates were submitted to the Animal Genome Size Database (http://www.genomesize.com).

Analysis

Genome size evolution in Trichoptera

Based on the genomes of six trichopteran species, Olsen et al. [30] found a 3-fold suborder-specific increase of genome size and hypothesized that genome size variation is correlated with their phylogeny. To test this hypothesis, we first reconstructed phylogenetic relationships by analyzing ~2,000 single-copy BUSCO genes from the 26 study species (Fig. 1 & Fig. 2, Fig. S48). We obtained a robust molecular phylogeny that was in agreement with recent phylogenetic hypotheses ([58], see supplementary note 6) and which showed that Trichoptera is divided into two suborders: Annulipalpia (Figs. 1 & 2: Clade A, blue) and Integripalpia [consisting of basal Integripalpia (Fig. 1: Clade B1-3, light green) and infraorder Phryganides (Fig. 1: clade B4, dark green)]. Trichopterans use silk to build diverse underwater structures

(see illustrations Fig.1; supplementary note 6, supplementary Fig. S48). Thus, we refer to Annulipalpia as 'fixed retreat- and net-spinners', to Phryganides (Integripalpia) as 'tube case-builders', and to basal Integripalpia as 'cocoon-builders'.

We used three approaches for estimating genome size across Trichoptera: *k-mer* distribution-estimates, backmapping of sequence data to available draft genomes (as described in [59]), and flow cytometry (FCM, supplementary note 7, supplementary figures S49-S72, Data S1). FCM estimates can be affected by chromatin condensation, the proportion of cells in G0 to G1 phases [60], [61] and endoreplication in insect cells and tissues [62] while *kmer*-based estimates can be affected by repetitive elements in the genome resulting in smaller genome size estimates (e.g. [63], [57], [64]). Bland-Altman plots (supplementary note 8, Fig. S73) revealed general agreement of all three methods in our study. However, the FCM estimates were generally higher compared to the sequence-based estimates (Fig. 1, Data S1) and, among all three approaches, this measure is expected to be the most accurate [8]. We observe that variation among the methods increased with genome size, indicating issues potentially caused by repeat content (see Results *Repeat dynamics*).

We observed large variation in genome size across the order. Genome size tends to be lower in 'fixed retreat- and net-spinners' and 'cocoon-builders' compared to 'tube case-builders' (Fig. 1). Specifically, we observe that genome size varies ~14-fold ranging from 1C = 154 Mb in 'cocoon-builders' (Fig. 1, B1: Hydroptilidae) to 1C = 2129 Mb in 'tube case-builders' (Fig. 1, clade B4: Limnephilidae). Of the 29 species analyzed by FCM, *Halesus digitatus* (Fig. 1, clade B4: Limnephilidae, Intergripalpia) possessed the largest genome (1C = 2129 Mb), while the genome of *Hydropsyche saxonica* (Fig. 1, clade A: Hydropsychidae, 'fixed retreat- and net-spinners') was the smallest (1C = 242 Mb). Genome size estimates based on sequence-based methods (*kmer*-based and back-mapping) range from 1C = 154 - 160 Mb in *Agraylea sexmaculata* (Fig. 1, clade B1: Hydroptilidae, 'cocoon-builders') to 1C = 1238 - 1400 Mb in *Sericostoma* sp. (Fig. 1, clade B4: Sericostomatidae, 'tube case-builders').

Repeat Dynamics

Repetitive element abundance and classification

To understand the structural basis of genome size variation across the order Trichoptera we explored repetitive element (RE) content. We found that major expansions of transposable elements (TEs) contribute to larger genomes in 'tube case-' and some 'cocoon-builders', but particularly in 'tube case-builders' with an average of ~600 Mb of REs compared to ~138 Mb in 'fixed retreat- and net-spinners' (Fig. 2 A, B). LINEs are the most abundant classified TEs in 'cocoon-' and 'tube case-builders' and comprise >154 Mb on average in 'tube case-builders', or an average genome proportion of 16.9% (range = 5.6–34.7%). This represents a 1.8- and 2.8-fold increase in genome proportion relative to 'cocoon-builders' and 'fixed retreat- and net-spinners', respectively. The LINE abundance of >312 Mb in *Odontocerum albicorne* exceeds the entire assembly lengths (152–282 Mb) of the three smallest genome assemblies (*Hydropsyche tenuis, Parapsyche elsis,* and *Agraylea sexmaculata*) (Fig. 2 A, B). DNA transposons also comprise large genomic fractions in both 'cocoon-' and 'tube case-builders' (averages of 54.4 Mb and 32.8 Mb, respectively). However, despite containing a large number of bps, they make up a smaller fraction of total bps in the genomes of 'cocoon-' and 'tube case-builders' than in 'fixed retreat- and net-spinners' (average genome proportion = 5.9%, 4.5%, and 11.1% in 'tube case-builders', 'cocoon-builders', and 'fixed retreat- and net-spinners', respectively) (Fig. 2 B), and thus cannot explain the larger genome sizes. SINEs, LTRs, *Penelope* (grouped with "other" repeats in Fig. 2), and satDNAs show a disproportionate increase in 'cocoon-' and 'tube case-builders', however, all categories combined make up a relatively small proportion of their genomes (all less than 3% on average in Integripalpia) (Fig. 2, B). Unclassified repeats are the most abundant repeat category across all Trichoptera but also show disproportionate expansions in both 'cocoon-' and 'case-builders' relative to 'fixed retreat- and net-spinners' (Fig. 2 A, B). The general trends noted in

our assembly-based analysis of REs were corroborated by our reference-free analysis of repeat abundance (Fig. S74, supplementary note 9).

TE age distribution analysis

To test whether the observed abundance patterns of specific TEs are driven by shared ancient proliferation events or more recent/ongoing activity of the respective TEs, we analyzed TE age distributions. TE age distributions showed a high abundance of recently diverged TE sequences in 'cocoon-' and 'tube case-builders', particularly in LINEs, DNA transposons, and LTRs in which the majority of TEs for a given class show 0–10% sequence divergence within copies of a given repeat (Fig. 3). This trend was particularly pronounced among 'tube case-builders' with several species showing high abundance of LINEs and DNA transposons with 0–5% sequence divergence (Fig. 3). This pattern suggests that the observed TE expansion is due primarily to ongoing TE activity within lineages rather than a few shared bursts of activity in ancestral lineages. This is further supported by our analysis of repeat sub-classes with landscape plots (Fig. S75). For example, in our study, LINE abundance is often due to the expansion of different LINE subclasses even between species in the same sub-clade (e.g., compare *Lepidostoma* with *Micrasema*, *Himalopsyche* with *Glossosoma*; Fig. S75). We also find evidence of shared ancient bursts of SINE activity in 'cocoon-' and 'tube case-builders', although SINEs are not an abundant repeat class in any species (avg. genomic proportion=1.9% stdev=1.7%) (Fig. S75).

Associations between TE sequences and protein-coding genes

During early exploration of our sequence data, it became apparent that some lineages showed an amplification in the copy number of fragments of sequences classified by BUSCO. We refer to these putative gene fragments discovered by BUSCO as 'repetitive BUSCOs' (supplementary Fig. S76, supplementary note 10). We found major expansions of repetitive

BUSCO gene fragments in ‘cocoon-’ and ‘tube case-builders’ (Fig. 4A) that are significantly correlated with total repeat abundance, as well as the genomic proportion of LINEs and DNA transposons (supplementary Fig. S77). To test whether repetitive fragments were due to TE insertions near or in the BUSCO genes or, conversely, due to the proliferation of “true” BUSCO protein-coding gene fragments throughout the genome, we examined patterns of BUSCO gene structure in pairwise alignments of three species pairs in which one species has a repetitive fragment (i.e., the “inflated” species) and its counterpart does not (i.e., the “reference” species). 73 of 75 randomly sampled alignments were consistently missing or non-contiguous in inflated regions in the reference sequence (Fig. 4B). The number of BLAST hits in the genome assembly of an “inflated species” when using the BUSCO sequence from the “reference species” as a query was significantly fewer than when using the BUSCO sequence from the “inflated species” as a query (Fig. 4C, Data S2). Our intersect analysis aimed at (I) quantifying shifts in associations between TEs and genic regions across Trichoptera lineages with varying repeat abundance and (II) determining if repetitive fragments were annotated as TEs. It showed that large fractions of BLAST hits for repetitive BUSCOs (when BLASTed to their own assembly) are in fact annotated transposable element sequences, especially LINEs and DNA transposons (Fig. 4D). We further validated the insertion of TEs by mapping Nanopore reads to subsets of repetitive BUSCOs, thus ensuring that junctions between repetitive BUSCOs and non-repetitive regions were embedded in full reads and do not occur disproportionately at ends of reads as would be expected in chimeric reads (see supplementary note 11, supplementary Figs. S76-S87). Taken together, these findings provide strong evidence that the repetitive fragments we observe in BUSCO genes are derived from TE sequences. These sequences may represent remnants left behind following historical insertion events, or TE sequences with open reading frames that are immediately adjacent to protein-coding genes and inadvertently classified as BUSCO fragments. Given the large fraction of BUSCO genes shown to have TE fragments (averages

of 11.2% and 21.4% in ‘cocoon-’ and ‘tube case-builders’, respectively, compared to 0.062% in annulipalipans), this finding highlights the impact of TEs on the composition of protein-coding genes in species with repeat-rich genomes.

Gene and genome duplications

Recently, a transcriptome-based study found evidence for putative ancient gene and genome duplications in hexapods, including potential WGD events in caddisflies [22], suggesting that duplication events could be responsible for some genome size variation in Trichoptera. We investigated whether this pattern persists with whole genome data and found that the age distribution of duplications in 18 genomes were significantly different compared to the background rate of gene duplication (Figs. 88 & 89). To identify if any significant peak is consistent with a potential WGD, we used mixture modeling to identify peaks in these gene age distributions. We found no obvious peak consistent with an ancient WGD suggesting that WGD did not play a major role in genome size variation in Trichoptera.

Discussion

The drivers and evolutionary consequences of genome size evolution are a topic of ongoing debate. Several models have been proposed [8]. Some hypothesize genome size to be a (mal)adaptive trait by impacting phenotypic traits such as developmental/life history, body size and other cell-size related effects [65], [66], [67], [68] reviewed in [8]. On the other hand, neutral theories suggest that DNA accumulation occurs only by genetic drift without selective pressures playing a major role in the accumulation or loss of DNA [the mutational hazard hypothesis (MHH, [23] and the mutational equilibrium hypothesis (MEH, [24]]. The MHH only allows for small deleterious effects for the accumulation of extra DNA which is accompanied by higher mutation rates in larger genomes [23], while the MEH focuses on the balance between insertions and deletions. It suggests that genome expansions arise by ‘bursts’

of duplication events or TE activity and genome shrinkage may be caused by a more constant rate of small deletions [24].

In this study, we observe that genome size varies ~14-fold across the order Trichoptera, with lower genome size estimates in 'fixed retreat- and net-spinners' and 'cocoon-builders' compared to 'tube case-builders' and explore potential drivers of genome size evolution. Although, recent genomic studies have shown evidence of bursts of gene duplication and gene family expansion during the evolution of hexapods [22], [69] the presence of ancient genome duplication events are still in debate [70], [71], [72]. We found no evidence of ancient WGD in the gene age distribution in our Trichoptera genomes, although we recognize that our current genome assemblies might be too fragmented to infer synteny. Future studies using chromosome-level genome assemblies are needed to allow a deeper exploration of putative ancient duplications observed by Li *et al.* [22].

We find evidence that TE expansions (especially LINEs) are the primary driver of genome size evolution in Trichoptera (Fig. 2, Fig. S3), which is consistent with the MEH explained above. Our TE age distribution analysis suggests that the high abundance of LINEs is due to ongoing/recent activity occurring independently across 'cocoon-' and particularly 'tube case-builders' (Fig. 3, Fig. S4). Thus, the shift to large genomes in these lineages does not appear to be due to a single (or few) shared ancient events, they rather maintain dynamic turnover in composition in their large genomes. Mutational bias affecting pathways tied to TE-regulation may affect insertion/deletion ratios and subsequently lead to lineage-specific shifts in genome size equilibrium [73]. Such changes may be stochastic (e.g., due to drift), or linked to traits that evolve on independent trajectories as lineages diverge and are thereby constrained by phylogeny. Ecological factors, demographic history and effective population size can further impact mutation rates. For example, environmental stress can trigger bursts of TE activity and elevated mutation rates [74], [75], [76] driving lineages that occupy niche space with frequent exposure to environmental stress toward increased TE loads and larger genomes. Similarly,

lineages with small effective population sizes or which are prone to population bottlenecks may have higher mutation rates and/or reduced efficacy of natural selection which would otherwise purge mildly deleterious TE load.

Although our study is not designed to pinpoint specific forces maintaining large genomes in some lineages, the pattern we observe in the distribution of genome size (i.e. lower genome size estimates in 'fixed retreat- and net-spinners' and 'cocoon-builders' compared to 'tube case-builders') leads us to hypothesize that ecological factors may play a role in genome size evolution in the order. The three focal groups discussed here exhibit markedly different ecological strategies. Larvae of 'fixed retreat- and net-spinners' generally occupy relatively narrow niche space in oxygen-rich flowing-water (mostly stream/river) environments where they rely on water currents to bring food materials to their filter nets. The evolutionary innovation of tube-case making is thought to have enabled 'tube case-builders' to occupy a much greater diversity of ecological niche space by allowing them to obtain oxygen in lentic (e.g., pond, lake, marsh) environments which are much more variable in temperature and oxygen availability than lotic environments [77], [78]. This environmental instability is greater over short (daily, seasonal) and long time scales (centuries, millennia) [79]. It is thus plausible these tube case-building lineages experience greater environmental stress and less stable population demographics that could lead to both more frequent TE bursts and reduced efficacy of natural selection in purging deleterious effects of TE expansions as described above [23], [24].

We show that TE expansions (especially LINEs and DNA transposons) in 'cocoon-' and 'tube case-builders' have a major impact on protein-coding gene regions (Fig. 4). These TE-gene associations show a linear relationship with increasing genome size. This trend is particularly pronounced among 'tube case-builders' in which an average of 21.4% of BUSCO genes that have internal or adjacent repetitive sequences (compared to 6.2% in annulipalpians). This finding corroborates other studies highlighting the role of TEs as drivers of rapid genome

evolution [80], [81], [82], [83] and highlights their impact on genomic regions that have potential effects on phenotypes. Questions remain as to what evolutionary roles such changes in genic regions may play. In general, TE insertions are considered to have deleterious effects on their host's fitness activity [84], [85]. They are known to "interrupt" genes [33], pose a risk of ectopic recombination that can lead to genome rearrangements [34], [31], [86], and have epigenetic effects on neighboring sequences [87], [88]. Therefore, purifying selection keeps TEs at low frequencies [33]. However, there is growing evidence that TE activity can also be a critical source of new genetic variation driving diversification via chromosomal rearrangements and transposition events which can result in mutations [89], including examples, of co-option [90], e.g. recent research in mammals has shown that DNA transposon fragments can be co-opted to form regulatory networks with genome-wide effects on gene expression [44].

Ecological correlates with genome size are widely discussed in other taxa [91], [92], [93], [94], [95]. Caddisflies and other diverse insect lineages that feature various microhabitat specializations, feeding modes, and/or the use of silk represent evolutionary replicates with contrasting traits and dynamic genome size evolution. They thus have high potential as models for understanding links between ecology and the evolution of REs, genomes, and phenotypes. Our study lays a foundation for future work in caddisflies that investigates the potential impact of TE expansions on phenotypes and tests for evidence of co-option/adaptive impacts of TE-rich genomes against a null of neutral or slightly deleterious effects.

Potential implications

Many open questions remain as to the causes and consequences of genome size evolution. As we move forward in an era where genome assemblies are attainable for historically intractable organisms (e.g. due to constraints given large genome sizes, tissue limitations, no close reference available) we can leverage new model systems spanning a greater diversity of life to

understand how genomes evolve. Here, we provide genomic resources and new genome size estimates across lineages of an underrepresented insect order that spans major variation in genome size. These data allowed us to study genome size evolution in a phylogenetic framework to reveal lineage-specific patterns in which genome size correlates strongly with phylogeny and ecological characteristics within lineages. We find that large genomes dominate lineages that occupy unstable environments, and that ongoing recent TE activity appears to maintain large genomes in these lineages. This leads us to hypothesize that ecological factors may play a predictable role in genome size evolution in this group. The future directions spawned by our findings highlight the potential for using Trichoptera and other diverse insect groups to understand the link between ecological and genomic diversity, a link that has been challenging to study with past models [8].

We also show that TE expansions are associated with increasing genome size and have an impact on protein-coding regions. These impacts have been greatest in the most species-rich and ecologically diverse caddisfly clades. While TEs are generally considered to have deleterious effects on their host's fitness activity, their roles can also be neutral or even adaptive. TE activity can be a critical source of new genetic variation and thus an important driver for diversification. Caddisflies and potentially other non-model insect groups are excellent models to test these contrasting hypotheses, as well as the potential impact of TEs on phenotypes. Using these models, especially with respect to the increasing emergence of high-quality insect genomes [52], will allow researchers to identify recurring patterns in TE dynamics and investigate their evolutionary implications across diverse clades.

Methods

DNA extraction, library preparation, sequencing, and sequence read processing

We extracted high molecular weight genomic DNA (gDNA) from 17 individuals (15 species) of caddisfly larvae (for sampling information, see Data S1) after removing the intestinal tracts using a salting-out protocol adapted from [96] as described in supplementary note 3. We generated gDNA libraries for a low-cost high-contiguity sequencing strategy, i.e. employing a combination of short (Illumina) and long read (Nanopore or PacBio) technologies as described in supplementary notes 1-2. For details on sequencing coverage for each specimen see Data S1.

De novo genome assembly, annotaion and quality assessment

We applied different assembly strategies for different datasets. First, we applied a long-read assembly method with subsequent short-read polishing as this method revealed good results in previous *de novo* assemblies in caddisflies [57]. In cases where this pipeline did not meet the expected quality regarding contiguity and BUSCO completeness, we applied de novo hybrid assembly approaches supplementary note 5. Illumina-only data was assembled with SPAdes [97] explained in supplementary note3).

Prior to annotating the individual genomes with MAKER2 v2.31.10 [98], [99] we used RepeatModeler v1.0.11 and RepeatMasker v4.0.7, to identify species-specific repetitive elements in each of the assemblies, relative to RepBase libraries v20181026; www.girinst.org). Transcriptome evidence for the annotation of the individual genomes included their species-specific or closely related *de novo* transcriptome provided by 1KITE (http://www.1kite.org/; last accessed November 11, 2019, Data S1) or downloaded from Genbank as well as the cDNA and protein models from *Stenopsyche tienmushanensis* [100] and *Bombyx mori* (AR102, GenBank accession ID# GCF_000151625.1). Additional protein evidence included the uniprot-sprot database (downloaded September 25, 2018). We masked repeats based on species-specific files produced by RepeatModeler. For *ab initio* gene prediction, species specific AUGUSTUS gene prediction models as well as *Bombyx mori*

SNAP gene models were provided to MAKER. The EvidenceModeler [101] and tRNAscan [102] options in MAKER were employed to produce a weighted consensus gene structure and to identify tRNAs genes. MAKER default options were utilized for BLASTN, BLASTX, TBLASTX searches. Two assemblies (*Agapetus fuscipens* GL3 and *Micrasema longulum* ML1) were not annotated because of their low contiguity. All protein sequences were assigned putative names by BlastP Protein–Protein BLAST 2.2.30+ searches [103] and were functionally annotated using command line Blast2Go v1.3.3 [104], see supplementary note 4, Figs. S1-S31).

We calculated assembly statistics with QUAST v5.0.2 [53] and examined completeness with BUSCO v3.0.2 [54], [55] using the Endopterygota odb9 dataset with the options *--long, –m = genome* and *–sp= fly*. A summary of the assembly statistics and BUSCO completeness is given in Data S1. The final genome assemblies and annotations were screened and filtered for potential contaminations with taxon-annotated GC-coverage (TAGC) plots using BlobTools v1.0 [56]. Details and blobplots are given in supplementary note 5 & supplementary Figs. S31-S47.

Species tree reconstruction

We used the single-copy orthologs resulting from the BUSCO analyses to generate a species tree. We first combined single-copy ortholog amino acid files from each species into a single FASTA for each ortholog. We then aligned them with the MAFFT L-INS-i algorithm [105]. We selected amino acid substitution models for each ortholog using ModelFinder (option -m mfp,[106] in IQtree v.2.0.6 [107] and estimated a maximum likelihood tree with 1000 ultrafast bootstrap replicates [108] with the BNNI correction (option -bb 1000 -bnni). We combined the best maximum likelihood tree from each gene for species tree analysis in ASTRAL-III [109]. We visualized the trees using FigTree v.1.4.4 (http://tree.bio.ed.ac.uk/software/figtree/).

Genome size estimations and genome profiling

Genome size estimates of 27 species were conducted using flow cytometry (FCM) according to Otto [110] using *Lycopersicon esculentum* cv. Stupické polnítyčkové rané (2C = 1.96 pg;[111]) as internal standard and propidium iodine as stain. Additionally, we used the short-read data sequenced in this study, to conduct genome profiling (estimation of major genome characteristics such as size, heterozygosity, and repetitiveness) using a *k-mer* distribution-based method (GenomeScope 2.0, [112]. Genome scope profiles are available online (see links to Genomescope 2 in Data S1). In addition, we applied a second sequencing-based method for genome size estimates, which uses the backmapping rate of sequenced reads to the assembly and coverage distribution (backmap.pl v0.1, [59]. Details of all three methods are described in supplementary note 7**.** Coverage distribution per position and genome size estimate from backmap.pl are shown in Figs. S49-72). We assessed the congruence among the three quantitative methods of measurement (Genomescope2, Backmap.pl and FCM) with Bland-Altman-Plots using the function BlandAltmanLeh::bland.altman.plot in ggplot2 [113] in RStudio (RStudio Team (2020). RStudio: Integrated Development for R. RStudio, PBC, Boston, MA URL http://www.rstudio.com/; supplementary note 8, Fig. S73).

Repeat dynamics

Repeat abundance and classification

We identified and classified repetitive elements in the genome assemblies of each species using RepeatModeler2.0 [114]. We annotated repeats in the contamination filtered assemblies with RepeatMasker 4.1.0 (http://www.repeatmasker.org) using the custom repeat libraries generated from RepeatModeler2 for each respective assembly with the search engine set to "ncbi" and using the -xsmall option. We converted the softmasked assembly resulting from the first RepeatMasker round into a hardmasked assembly using the lc2n.py script (https://github.com/PdomGenomeProject/repeat-masking). Finally, we re-ran RepeatMasker

on the hard-masked genome with RepeatMasker's internal arthropod repeat library using -species "Arthropoda". We then merged RepeatMasker output tables from both runs to summarize the abundance of repeat categories.

We also estimated repetitive element abundance and composition using RepeatExplorer2 [115], [116]. This reference-free approach quantifies repeats directly from unassembled short-read data using clustering-based methods. This analysis allowed us to test for general consistency of patterns with our assembly-based approach described above, and to test for the presence of abundant repeat categories such as satellite DNAs which can comprise large fractions of genomes yet can be prone to poor representation in the genome assembly. Prior to analysis in RepeatExplorer2 we normalized input data sets to 0.5x coverage using RepeatProfiler [117] and seqtk (https://github.com/lh3/seqtk), and then ran RepeatExplorer2 clustering with the Metazoa 3.0 database specified for annotation (supplementary Fig. S74).

TE age distribution analysis

We further characterized repetitive element dynamics in Trichoptera by analyzing TE landscapes, which show relative age differences among TE sequences and their genomic abundance. We used these analyses to test whether abundance patterns of specific TEs are driven by shared ancient proliferation events or more recent/ongoing activity of the respective TEs. For example, if shared ancient proliferation is driving abundance patterns of a given TE, the majority of its copies would show moderate to high sequence divergence (e.g., >10% pairwise divergence). In contrast, if abundance patterns are driven by recent/ongoing activity of a given TE, we would expect the majority of its sequences to show low sequence divergence (e.g., 0–10%). We generated TE landscape plots using dnaPipeTE v1.3.1 [118] with genomic coverage for each species sampled to 0.5X prior to analysis and the -RM_lib flag set to the Repbase v20170127 repeat library (supplementary Fig. S75).

TE sequence associations with protein-coding genes

During the early exploration of our sequence data we noted that copy number profiles of some BUSCO genes showed regions of unexpected high copy number with the frequency of such genes varying widely across species. Following this observation, we analyzed all BUSCO genes for all species to quantify the abundance of repetitive BUSCOs across samples and test two hypotheses that could account for the observed patterns: (I) inflated copy-number of BUSCO gene fragments occur when sequences of repetitive elements (e.g. TEs) are inserted into (or immediately adjacent to genes; (II) inflated regions are BUSCO gene fragments that have proliferated throughout the genome (e.g., by hitch-hiking with TEs). We investigated associations between TEs and genic sequences in Trichoptera lineages by quantifying the abundance of repetitive sequences found within or adjacent to BUSCO genes (for presence and absence of repetitive BUSCOs see Fig. S5, Data S2). This analysis also allowed us to quantify shifts in associations between TEs and genic regions across Trichoptera lineages with varying repeat abundance. We used copy number profiles to identify BUSCO genes with repetitive sequences based on coverage profiles and quantified their genomic abundance by using each repetitive BUSCO as a query in a BLAST search against their respective genome assembly. We then conducted intersect analysis for all unique BUSCO hits from repetitive sequences to determine if repetitive fragments were annotated as TEs. We calculated the total number of bases in filtered BLAST after subtracting the number of bases at the locus belonging to all 'complete' BUSCO genes and categorize BUSCO repeats based on their annotation status and repeat classification using custom scripts (available at https://app.box.com/folder/136985262731?v=trich-genomes). We plotted the number of BUSCO repeats belonging to repetitive element categories (i.e., classes and subclasses) alongside plots of the relative genomic abundance of each respective category. For details of this method readers are directed to supplementary note 10. To validate that the insertion of TEs are not an artefact of poor assembly quality, we mapped Nanopore reads back to

519 repetitive BUSCO sequences and showed that junctions between repetitive BUSCOs and non-
520 repetitive regions are embedded in full reads (see supplementary note 11, supplementary
521 Table S1, supplementary Figs.78-87). In addition, we investigated BUSCO genes with
522 repetitive sequences by pairwise alignments. Specifically, we visualized alignments of
523 BUSCOs with repetitive fragments alongside orthologous BUSCOS that lack repetitive
524 fragments taken from closely related species. To test whether repetitive fragments are due to
525 TE insertions or to proliferation of true BUSCO gene fragments we examined patterns of
526 BUSCO gene structure in pairwise alignments of species in which one species has a repetitive
527 fragment (i.e., the "inflated" species) and its counterpart does not (i.e., the "reference"
528 species). To reduce the likelihood that indels due to sequence divergence (i.e., not due to REs)
529 would complicate the analysis we conducted this test with three pairs of closely related
530 species: *Micrasema longulum* and *Micrasema minimum*; *Rhyacophila brunnea* and
531 *Rhyacophila evoluta*; and *Hesperophylax magnus* and *Halesus radiatus*. We predicted that if
532 repetitive regions were due to RE sequence insertion (i.e., Hyp1 above) that inflated species
533 would show indels at coordinates of repetitive fragments relative to reference species. If
534 repetitive regions were due to proliferation of true BUSCO sequence (i.g., Hyp2 above) we
535 predicted that alignments would be contiguous over the repetitive region. We further tested
536 this prediction by taking the set of BUSCOs that are only repetitive in the inflated species and
537 contrasted results of two BLAST searches followed by an intersect analysis. A detailed
538 description of this method is provided in supplementary note 9.
539
540 *Gene and Genome duplications*
541 *Inference of WGDs from gene age distributions*
542 To recover signal from potential WGDs, for each genome, we used the DupPipe pipeline to
543 construct gene families and estimate the age distribution of gene duplications [119],
544 https://bitbucket.org/barkerlab/evopipes/src/master/). We translated DNA sequences and

identified ORFs by comparing the Genewise [120] alignment to the best- hit protein from a collection of proteins from 24 metazoan genomes from Metazome v3.0. For all DupPipe runs, we used protein- guided DNA alignments to align our nucleic acid sequences while maintaining the ORFs. We estimated synonymous divergence (K_s) using PAML with the F3X4 model [121] for each node in the gene family phylogenies. We first identified taxa with potential WGDs by comparing their paralog ages to a simulated null distribution without ancient WGDs using a K-S goodness-of-fit test [122]. We then used mixture modeling to identify if any significant peaks consistent with a potential WGD and to estimate their median paralog K_s values. Significant peaks were identified using a likelihood ratio test in the boot.comp function of the package mixtools in R [123].

Figures Legends:

Fig. 1: Ecological diversity (right) and genome size (left) in caddisflies. Phylogenetic relationships derived from ASTRAL-III analyses using single BUSCO genes. Goeridae, which was not included in the BUSCO gene set, was placed according to [58]. ASTRAL support values (local posterior probabilities) higher than 0.9 are given for each node. Nodes with less than 0.9 support were collapsed. Taxa were collapsed to family level. Trichoptera are divided into two suborders: Annulipalpia ('fixed retreat- and net-spinners', clade A: blue) and Intergripalpia (clade B: green) which includes basal Integripalpia ('cocoon-builders', clades B1-B3, dark green) and Phryganides or 'tube case-builders' (clade B4: light green). 'Cocoon-builders' are divided into 'purse case'- (clade B1), 'tortoise case-building' (clade B2) and 'free-living' (clade B3) families. Genome size estimates based on different methods (Genomescope2: orange, Backmap.pl: black, Flow Cytometry (FCM): brown) are given for various caddisfly families. Each dot corresponds to a mean estimate of a species. For detailed information on the species and number of individuals used in each method see Data S1.

Colors and clade numbers in the phylogenetic tree refer to colored boxes with illustrations. The following species are illustrated by Ralph Holzenthal: a: *Hydropsyche sp.* (Hydropsychidae); b: *Chimarra sp.* (Philopotamidae); c: *Stenopsyche sp.* (Stenopsychidae); d: *Polycentropus* sp. (Polycentropodidae); e: *Agraylea sp.* (Hydroptilidae); f: *Glossosoma sp.* (Glossosomatidae); g: *Rhyacophila sp.* (Rhyacophilidae); h: *Fabria inornata* (Phryganeidae); i: *Micrasema* sp. (Brachycentridae); j:*Goera fuscula* (Goeridae); k: *Sphagnophylax meiops* (Limnephilidae); l: *Psilotreta sp.* (Odontoceridae), m: *Grumicha grumicha* (Sericostomatidae).

Fig. 2: Repeat abundance and classification in 26 caddisfly genomes. Number of bp for each repeat type is given for each caddisfly genome. A: Repeat abundance and classification. Phylogenetic tree was reconstructed with ASTRAL-III using single BUSCO genes from the genome assemblies. Nodes with ASTRAL support below 0.9 were collapsed. Species names corresponding to the abbreviations in the tree can be found in Table 1. Trichoptera are divided into two suborders: Annulipalpia ('fixed retreat- and net-spinners', clade A: blue) and Intergripalpia (clade B: green) which includes basal Integripalpia ('cocoon-builders', clades B1-B3, dark green) and Phryganides or 'tube case-builders' (clade B4: light green). 'Cocoon-builders' are divided into 'purse case'- (clade B1), 'tortoise case-building' (clade B2) and 'free-living' (clade B3) families. An illustration of a representative of each clade is given. The "other_repeats" category includes: rolling-circles, *Penelope*, low-complexity, simple repeats, and small RNAs. B: Boxplots summarizing shifts in the genomic proportion of RE categories in major Trichoptera lineages.

Fig. 3: Transposable element age distribution landscapes. Representative examples are chosen from major Trichoptera lineages. The y-axis shows TE abundance as a proportion of the genome (e.g., 1.0 = 1% of the genome). The x-axis shows sequence divergence relative to

TE consensus sequences for major TE classes. TE classes with abundance skewed toward the left (i.e., low sequence divergence) are inferred to have a recent history of diversification relative to TE classes with right-skewed abundance. Plots were generated in dnaPipeTE. Plots for all species are shown in supplementary Fig. 4. For tip labels of the phylogenetic tree see Fig. 2.

Fig. 4: TE-BUSCO-gene associations in Trichoptera species. (A) Raw abundance of BUSCO with repetitive fragments present in the assembly out of a total of 2442BUSCOs studied in the OrthoDB 9 Endopterygota dataset. (B) Upper plot: An example of a copy number profile of a repetitive BUSCO gene (BUSCO EOG090R02Q9 from ML1 ('inflated species')) which shows unexpected high coverage in the second exon putatively due to the presence of a TE-derived sequence fragment. Lower plot: alignments of repetitive BUSCOs to non-repetitive orthologs taken from a closely related species ('reference species') are consistently non-contiguous in regions of inflated coverage in the repetitive BUSCO, consistent with the presence of a TE-derived sequence fragment in the repetitive BUSCO which is absent in the reference species. (C) Summary of total bases annotated as REs obtained from each of two BLAST searches. First, when repetitive BUSCOs are BLASTed against an assembly for the same species BLAST hits included megabases of annotated repeats (dark plots). Second, when non-repetitive orthologs of the repetitive BUSCOs in the first search are taken from a close relative and BLASTed against the inflated species, there is a dramatic drop in BLAST hits annotated as REs. Note log scale on the y-axis. (D) Summary of annotations for BLAST hits for classified REs when repetitive BUSCOs are BLASTed against an assembly of the same species.

Table 1. Comparison of assembly and annotation statistics of all available Trichoptera Genomes. *Assemblies produced in this study. **$N_{Arthropoda}$=2442, % of present BUSCOS = complete + fragmented.

Species	Abbre-viation	Accession number	Length (bp)	N50 (kbp)	No. of contigs	BUSCOS %**
*Agapetus fuscipes**	AF	JAGTXP000000000	552,637,417	2.8	291,536	75.5
*Agraylea sexmaculata**	AS	JAGTTH000000000	196,044,125	86	7,050	96.5
Agrypnia vestita [30]	AV	JADDOH000000000	1,352,945,503	111.8	25,541	92.8
*Drusus annulatus**	DA	JAGWCC000000000	727,941,535	1,043.7	2,401	96.8
*Glossosoma conforme**	GC1	JAGTXR000000000	568,249,599	2,212.1	653	91.8
Glossosoma conforme [124]	GC2	GCA_003347265.1	604,293,666	17.1	119,821	92.1
Glyphotaelius pellucidula [125]	GP	Glyphotaelius_pellucidus_k51_scaffolds	623,431,006	1.6	461,749	68.6
*Halesus radiatus**	HR	JAHDVE000000000	973,356,502	125.2	12,484	90.6
*Himalopsyche phryganea**	HP	JAGVSL000000000	633,785,554	4,634	710	97.8
Hesperophylax magnus [30]	HM	JADDOG000000000	1,275,967,528	768.2	6,877	95.2
Hydropsyche tenuis [57]	HT	GCA_009617725.1	229,663,394	2,190.1	403	97.6
*Lepidostoma basale**	LB	JAGTTH000000000	769,208,668	1,052	1,621	97.0
Limnephilus lunatus [69]	LL	GCA_000648945.2	1,369,180,260	69.1	58,718	91
*Micrasema longulum**	ML2	JAGXCS000000000	668,600,304	2.5	368,330	69.8
*Micrasema longulum**	ML1	JAGVSM000000000	585,245,295	170.5	5,451	84.5
*Micrasema minimum**	MM	JAGVSQ000000000	329,257,313	69.5	7,561	67.1
*Micropterna sequax**	MS	JAGUCF000000000	778,692,278	7.9	144,286	68.9
*Odontocerum albicorne**	OA	JAGTXQ000000000	1,305,984,461	266.4	9,303	95.9
*Parapsyche elsis**	PE	JAGVSN000000000	282,185,525	5,591.7	159	97.4
*Philopotamus ludificatus**	PL	JAGXCT000000000	360,300,449	67.5	37,274	92.6
Plectrocnemia conspersa [57]	PC	GCA_009617715.1	396,695,105	869	1,614	97.8
*Rhyacophila brunnea**	RB	JAGYXB000000000	1,086,872,538	1,030.6	2,125	97.3
*Rhyacophila evoluta**	RE2	JAGVSQ000000000	565,830,460	9.9	114,057	91.7

*Rhyacophila evoluta**	RE1	JAGVSO000000000	562,550,625	9.7	111,706	91.7
Sericostoma sp.[126]	SS	GCA_003003475.1	1,015,727,762	3.2	561,698	60.3
Stenopsyche tienhuanesis [100]	ST	GCA_008973525.1	451,494,475	1,296.7	552	97.6

Data and materials availability: This project has been deposited at NCBI under BioProject ID: PRJNA558902. The data sets supporting the results of this article are available in the Supplementary, Data files S1 and S2 and at https://app.box.com/folder/136985262731?v=trich-genomes. *The data available at the link will be uploaded to GigaDB when the paper is sent out for review.*

Declarations

Consent for publication: Not applicable

Competing interests: Authors declare that they have no competing interests.

Funding

This work is a result of the LOEWE-Centre for Translational Biodiversity Genomics funded by the Hessen State Ministry of Higher Education, Research and the Arts (HMWK) that supported JH and SUP, as well as internal funds of Senckenberg Research Institute provided to JP. JSS was supported by an NSF Postdoctoral Research Fellowship in Biology (DBI-1811930) and an NIH General Medical Sciences award (R35GM119515) to AML. Sequencing was, in part, supported by BYU start-up funds to PBF and funds from the Army Research Office, Life Science Division (Award no. W911NF-13-1-0319) to RJS.

Author's contributions:

Conceptualization –JH, JSS, PBF, SUP

Data curation – JH

Formal Analysis - JH, JM, JP, JSS, PBF, ZL

Funding acquisition – AML, PBF, SUP, RJS, RJS

Investigation – JH, JP, JSS, PBF, ZL

Methodology – AML, JSS, JP, JVS, PBF

Project administration – SUP

Resources – JP, MB, PBF, SUP

Visualization - JH, JSS

Writing – original draft – JH, JSS, PBF, ZL

Writing – review & editing – AML, JH, JM, JP, JSS, JVS, MB, PBF, RJS, ,RJS, SUP, ZL

Acknowledgments

The authors thank Ralph Holzenthal for providing illustrations of larval Trichoptera and the structures they build. We thank Bob Wisseman for collecting *Himalopsyche phryganeae.*

References

1. Cornette R, Gusev O, Nakahara Y, Shimura S, Kikawada T, Okuda T. Chironomid midges (Diptera, chironomidae) show extremely small genome sizes. *Zoolog Sci*. 2015; doi: 10.2108/zs140166.

2. Westerman M, Barton NH, Hewitt GM. Differences in DNA content between two chromosomal races of the grasshopper Podisma pedestris. *Heredity*. Nature Publishing Group; 1987; doi: 10.1038/hdy.1987.36.

3. Thomas CA. The genetic organization of chromosomes. *Annu Rev Genet*. Annual Reviews; 1971; doi: 10.1146/annurev.ge.05.120171.001321.

4. Bernard J. The Eukaryote Genome in Development and Evolution. Springer Netherlands;

5. Gregory TR. The C-value Enigma in Plants and Animals: A Review of Parallels and an Appeal for Partnership. *Ann Bot*. 2005; doi: 10.1093/aob/mci009.

6. Elliott TA, Gregory TR. What's in a genome? The C-value enigma and the evolution of eukaryotic genome content. *Philos Trans R Soc B Biol Sci*. Royal Society; 2015; doi: 10.1098/rstb.2014.0331.

7. Abdel-Haleem H. The Origins of Genome Architecture. *J Hered*. 2007; doi: 10.1093/jhered/esm073.

8. Blommaert J. Genome size evolution: towards new model systems for old questions. *Proc R Soc B Biol Sci*. Royal Society; 2020; doi: 10.1098/rspb.2020.1441.

9. Canapa A, Barucca M, Biscotti MA, Forconi M, Olmo E. Transposons, Genome Size, and Evolutionary Insights in Animals. *Cytogenet Genome Res*. 2015; doi: 10.1159/000444429.

10. Petrov DA. Evolution of genome size: new approaches to an old problem. *Trends Genet TIG*. 2001; doi: 10.1016/s0168-9525(00)02157-0.

11. Orr HA. "Why Polyploidy is Rarer in Animals Than in Plants" Revisited. *Am Nat*. The University of Chicago Press; 1990; doi: 10.1086/285130.

12. Otto SP, Whitton J. Polyploid Incidence and Evolution. *Annu Rev Genet*. 2000; doi: 10.1146/annurev.genet.34.1.401.

13. Hallinan NM, Lindberg DR. Comparative analysis of chromosome counts infers three paleopolyploidies in the mollusca. *Genome Biol Evol*. 2011; doi: 10.1093/gbe/evr087.

14. Alexandrou MA, Swartz BA, Matzke NJ, Oakley TH. Genome duplication and multiple evolutionary origins of complex migratory behavior in Salmonidae. *Mol Phylogenet Evol*. 2013; doi: 10.1016/j.ympev.2013.07.026.

15. Campbell MA, Hale MC, McKinney GJ, Nichols KM, Pearse DE. Long-Term Conservation of Ohnologs Through Partial Tetrasomy Following Whole-Genome Duplication in Salmonidae. *G3 GenesGenomesGenetics*. 2019; doi: 10.1534/g3.119.400070.

16. Woods IG, Wilson C, Friedlander B, Chang P, Reyes DK, Nix R, et al.. The zebrafish gene map defines ancestral vertebrate chromosomes. *Genome Res*. 2005; doi: 10.1101/gr.4134305.

17. Berthelot C, Brunet F, Chalopin D, Juanchich A, Bernard M, Noël B, et al.. The rainbow trout genome provides novel insights into evolution after whole-genome duplication in vertebrates. *Nat Commun*. Nature Publishing Group; 2014; doi: 10.1038/ncomms4657.

18. Glasauer SMK, Neuhauss SCF. Whole-genome duplication in teleost fishes and its evolutionary consequences. *Mol Genet Genomics MGG*. 2014; doi: 10.1007/s00438-014-0889-2.

19. Clarke TH, Garb JE, Hayashi CY, Arensburger P, Ayoub NA. Spider Transcriptomes Identify Ancient Large-Scale Gene Duplication Event Potentially Important in Silk Gland Evolution. *Genome Biol Evol*. 2015; doi: 10.1093/gbe/evv110.

20. Kenny NJ, Chan KW, Nong W, Qu Z, Maeso I, Yip HY, et al.. Ancestral whole-genome duplication in the marine chelicerate horseshoe crabs. *Heredity*. Nature Publishing Group; 2016; doi: 10.1038/hdy.2015.89.

21. Schwager EE, Sharma PP, Clarke T, Leite DJ, Wierschin T, Pechmann M, et al.. The house spider genome reveals an ancient whole-genome duplication during arachnid evolution. *BMC Biol*. 2017; doi: 10.1186/s12915-017-0399-x.

22. Li Z, Tiley GP, Galuska SR, Reardon CR, Kidder TI, Rundell RJ, et al.. Multiple large-scale gene and genome duplications during the evolution of hexapods. *Proc Natl Acad Sci*. National Academy of Sciences; 2018; doi: 10.1073/pnas.1710791115.

23. Lynch M, Conery JS. The Origins of Genome Complexity. *Science*. American Association for the Advancement of Science; 2003; doi: 10.1126/science.1089370.

24. Petrov DA. Mutational Equilibrium Model of Genome Size Evolution. *Theor Popul Biol*. 2002; doi: 10.1006/tpbi.2002.1605.

25. Wicker T, Sabot F, Hua-Van A, Bennetzen JL, Capy P, Chalhoub B, et al.. A unified classification system for eukaryotic transposable elements. *Nat Rev Genet*. Nature Publishing Group; 2007; doi: 10.1038/nrg2165.

26. Kelley JL, Peyton JT, Fiston-Lavier A-S, Teets NM, Yee M-C, Johnston JS, et al.. Compact genome of the Antarctic midge is likely an adaptation to an extreme environment. *Nat Commun*. Nature Publishing Group; 2014; doi: 10.1038/ncomms5611.

27. Wang X, Fang X, Yang P, Jiang X, Jiang F, Zhao D, et al.. The locust genome provides insight into swarm formation and long-distance flight. *Nat Commun*. Nature Publishing Group; 2014; doi: 10.1038/ncomms3957.

28. Petersen M, Armisén D, Gibbs RA, Hering L, Khila A, Mayer G, et al.. Diversity and evolution of the transposable element repertoire in arthropods with particular reference to insects. *BMC Ecol Evol*. 2019; doi: 10.1186/s12862-018-1324-9.

29. Gilbert C, Peccoud J, Cordaux R. Transposable Elements and the Evolution of Insects. *Annu Rev Entomol*. Annual Reviews; 2021; doi: 10.1146/annurev-ento-070720-074650.

30. Olsen LK, Heckenhauer J, Sproul JS, Dikow RB, Gonzalesz VL, Kweskin MP, et al.. De novo whole genome assemblies of Agrypnia vestita Walker, and Hesperophlax magnus Banks reveal substantial repetitive element expansion in tube case-making caddisflies (Insecta: Trichoptera). *Genome Biol Evol*. 2021; doi: 10.1093/gbe/evab013.

31. Montgomery E, Charlesworth B, Langley CH. A test for the role of natural selection in the stabilization of transposable element copy number in a population of Drosophila melanogaster. *Genet Res*. Cambridge University Press; 1987; doi: 10.1017/S0016672300026707.

32. Charlesworth B, Langley CH. Transposition of copia elements in Drosophila. *Nature*. Nature Publishing Group; 1988; doi: 10.1038/332021b0.

33. Charlesworth B, Langley CH. The Population Genetics of Drosophila Transposable Elements. *Annu Rev Genet*. 1989; doi: 10.1146/annurev.ge.23.120189.001343.

34. Montgomery EA, Huang SM, Langley CH, Judd BH. Chromosome rearrangement by ectopic recombination in Drosophila melanogaster: genome structure and evolution. *Genetics*. 1991; doi: 10.1093/genetics/129.4.1085.

35. Mieczkowski PA, Lemoine FJ, Petes TD. Recombination between retrotransposons as a source of chromosome rearrangements in the yeast Saccharomyces cerevisiae. *DNA Repair*. 2006; doi: 10.1016/j.dnarep.2006.05.027.

36. Ugarković Đ, Plohl M. Variation in satellite DNA profiles—causes and effects. *EMBO J*. John Wiley & Sons, Ltd; 2002; doi: 10.1093/emboj/cdf612.

37. Ferree PM, Barbash DA. Species-Specific Heterochromatin Prevents Mitotic Chromosome Segregation to Cause Hybrid Lethality in Drosophila. *PLOS Biol*. Public Library of Science; 2009; doi: 10.1371/journal.pbio.1000234.

38. Gahan LJ, Gould F, Heckel DG. Identification of a Gene Associated with Bt Resistance in Heliothis virescens. *Science*. American Association for the Advancement of Science; 2001; doi: 10.1126/science.1060949.

39. Chen S, Li X. Transposable elements are enriched within or in close proximity to xenobiotic-metabolizing cytochrome P450 genes. *BMC Evol Biol*. 2007; doi: 10.1186/1471-2148-7-46.

40. González J, Lenkov K, Lipatov M, Macpherson JM, Petrov DA. High Rate of Recent Transposable Element–Induced Adaptation in Drosophila melanogaster. *PLOS Biol*. Public Library of Science; 2008; doi: 10.1371/journal.pbio.0060251.

41. González J, Karasov TL, Messer PW, Petrov DA. Genome-Wide Patterns of Adaptation to Temperate Environments Associated with Transposable Elements in Drosophila. *PLOS Genet*. Public Library of Science; 2010; doi: 10.1371/journal.pgen.1000905.

42. Itokawa K, Komagata O, Kasai S, Okamura Y, Masada M, Tomita T. Genomic structures of Cyp9m10 in pyrethroid resistant and susceptible strains of Culex quinquefasciatus. *Insect Biochem Mol Biol*. 2010; doi: 10.1016/j.ibmb.2010.06.001.

43. Hof AE van't, Campagne P, Rigden DJ, Yung CJ, Lingley J, Quail MA, et al.. The industrial melanism mutation in British peppered moths is a transposable element. *Nature*. Nature Publishing Group; 2016; doi: 10.1038/nature17951.

44. Feschotte C. Transposable elements and the evolution of regulatory networks. *Nat Rev Genet*. Nature Publishing Group; 2008; doi: 10.1038/nrg2337.

45. Ellison CE, Bachtrog D. Dosage Compensation via Transposable Element Mediated Rewiring of a Regulatory Network. *Science*. American Association for the Advancement of Science; 2013; doi: 10.1126/science.1239552.

46. Santos ME, Braasch I, Boileau N, Meyer BS, Sauteur L, Böhne A, et al.. The evolution of cichlid fish egg-spots is linked with a cis -regulatory change. *Nat Commun*. Nature Publishing Group; 2014; doi: 10.1038/ncomms6149.

47. Alfsnes K, Leinaas HP, Hessen DO. Genome size in arthropods; different roles of phylogeny, habitat and life history in insects and crustaceans. *Ecol Evol*. 2017; doi: https://doi.org/10.1002/ece3.3163.

48. : Trichoptera World Checklist. https://entweb.sites.clemson.edu/database/trichopt/ Accessed 2021 May 8.

49. Wiggins GB, Mackay RJ. Some Relationships between Systematics and Trophic Ecology in Nearctic Aquatic Insects, with Special Reference to Trichoptera. *Ecology*. 1978; doi: https://doi.org/10.2307/1938234.

50. Mackay RJ, Wiggins GB. Ecological Diversity in Trichoptera. *Annu Rev Entomol*. 1979; doi: 10.1146/annurev.en.24.010179.001153.

51. Hotaling S, Kelley JL, Frandsen PB. Aquatic Insects Are Dramatically Underrepresented in Genomic Research. *Insects*. Multidisciplinary Digital Publishing Institute; 2020; doi: 10.3390/insects11090601.

52. Hotaling S, Sproul JS, Heckenhauer J, Powell A, Larracuente AM, Pauls SU, et al.. Long-reads are revolutionizing 20 years of insect genome sequencing. *Genome Biol Evol*. 2021; doi: 10.1093/gbe/evab138.

53. Gurevich A, Saveliev V, Vyahhi N, Tesler G. QUAST: Quality assessment tool for genome assemblies. *Bioinforma Oxf Engl*. 2013; doi: 10.1093/bioinformatics/btt086.

54. Simão F, Waterhouse R, Ioannidis P, Zdobnov E. BUSCO: Assessing genome assembly and annotation completeness with single-copy orthologs. *Bioinforma Oxf Engl*. 2015; doi: 10.1093/bioinformatics/btv351.

55. Waterhouse RM, Seppey M, Simão FA, Manni M, Ioannidis P, Klioutchnikov G, et al.. BUSCO Applications from Quality Assessments to Gene Prediction and Phylogenomics. *Mol Biol Evol*. 2018; doi: 10.1093/molbev/msx319.

56. Laetsch DR, Blaxter ML. BlobTools: Interrogation of genome assemblies. *F1000Research*. 2017; doi: 10.12688/f1000research.12232.1.

57. Heckenhauer J, Frandsen PB, Gupta DK, Paule J, Prost S, Schell T, et al.. Annotated Draft Genomes of Two Caddisfly Species Plectrocnemia conspersa CURTIS and Hydropsyche tenuis NAVAS (Insecta: Trichoptera). *Genome Biol Evol*. 2019; doi: 10.1093/gbe/evz264.

58. Thomas JA, Frandsen PB, Prendini E, Zhou X, Holzenthal RW. A multigene phylogeny and timeline for Trichoptera (Insecta). *Syst Entomol*. 2020; doi: https://doi.org/10.1111/syen.12422.

59. Schell T, Feldmeyer B, Schmidt H, Greshake B, Tills O, Truebano M, et al.. An Annotated Draft Genome for Radix auricularia (Gastropoda, Mollusca). *Genome Biol Evol*. 2017; doi: 10.1093/gbe/evx032.

60. Hanrahan SJ, Johnston JS. New genome size estimates of 134 species of arthropods. *Chromosome Res Int J Mol Supramol Evol Asp Chromosome Biol*. 2011; doi: 10.1007/s10577-011-9231-6.

61. Yu Y-S, Jin S, Cho N, Lim J, Kim C-H, Lee S-G, et al.. Genome Size Estimation of Callipogon relictus Semenov (Coleoptera: Cerambycidae), an Endangered Species and a Korea Natural Monument. *Insects*. Multidisciplinary Digital Publishing Institute; 2021; doi: 10.3390/insects12020111.

62. Hare EE, Johnston JS. Genome size determination using flow cytometry of propidium iodide-stained nuclei. *Methods Mol Biol Clifton NJ*. 2011; doi: 10.1007/978-1-61779-228-1_1.

831 63. Austin CM, Tan MH, Harrisson KA, Lee YP, Croft LJ, Sunnucks P, et al.. De novo
832 genome assembly and annotation of Australia's largest freshwater fish, the Murray cod
833 (Maccullochella peelii), from Illumina and Nanopore sequencing read. *GigaScience*. 2017;
834 doi: 10.1093/gigascience/gix063.

835 64. Pflug JM, Holmes VR, Burrus C, Johnston JS, Maddison DR. Measuring Genome Sizes
836 Using Read-Depth, k-mers, and Flow Cytometry: Methodological Comparisons in Beetles
837 (Coleoptera). *G3 GenesGenomesGenetics*. 2020; doi: 10.1534/g3.120.401028.

838 65. Bennett MD, Riley R. The duration of meiosis. *Proc R Soc Lond B Biol Sci*. Royal
839 Society; 1971; doi: 10.1098/rspb.1971.0066.

840 66. Cavalier-Smith T. Nuclear volume control by nucleoskeletal DNA, selection for cell
841 volume and cell growth rate, and the solution of the DNA C-value paradox. *J Cell Sci*. 1978;
842 doi: 10.1242/jcs.34.1.247.

843 67. Gregory TR, Hebert PDN. The Modulation of DNA Content: Proximate Causes and
844 Ultimate Consequences. *Genome Res*. 1999; doi: 10.1101/gr.9.4.317.

845 68. CAVALIER-SMITH T. Economy, Speed and Size Matter: Evolutionary Forces Driving
846 Nuclear Genome Miniaturization and Expansion. *Ann Bot*. 2005; doi: 10.1093/aob/mci010.

847 69. Thomas GWC, Dohmen E, Hughes DST, Murali SC, Poelchau M, Glastad K, et al.. Gene
848 content evolution in the arthropods. *Genome Biol*. 2020; doi: 10.1186/s13059-019-1925-7.

849 70. Nakatani Y, McLysaght A. Macrosynteny analysis shows the absence of ancient whole-
850 genome duplication in lepidopteran insects. *Proc Natl Acad Sci U S A*. 2019; doi:
851 10.1073/pnas.1817937116.

852 71. Li Z, Tiley GP, Rundell RJ, Barker MS. Reply to Nakatani and McLysaght: Analyzing
853 deep duplication events. *Proc Natl Acad Sci*. National Academy of Sciences; 2019; doi:
854 10.1073/pnas.1819227116.

855 72. Roelofs D, Zwaenepoel A, Sistermans T, Nap J, Kampfraath AA, Van de Peer Y, et al..
856 Multi-faceted analysis provides little evidence for recurrent whole-genome duplications
857 during hexapod evolution. *BMC Biol*. 2020; doi: 10.1186/s12915-020-00789-1.

858 73. Lamichhaney S, Catullo R, Keogh JS, Clulow S, Edwards SV, Ezaz T. A bird-like
859 genome from a frog: Mechanisms of genome size reduction in the ornate burrowing frog,
860 Platyplectrum ornatum. *Proc Natl Acad Sci*. National Academy of Sciences; 2021; doi:
861 10.1073/pnas.2011649118.

862 74. Capy P, Gasperi G, Biémont C, Bazin C. Stress and transposable elements: co-evolution
863 or useful parasites? *Heredity*. 2000; doi: 10.1046/j.1365-2540.2000.00751.x.

864 75. Pecinka A, Dinh HQ, Baubec T, Rosa M, Lettner N, Scheid OM. Epigenetic Regulation of
865 Repetitive Elements Is Attenuated by Prolonged Heat Stress in Arabidopsis. *Plant Cell*. 2010;
866 doi: 10.1105/tpc.110.078493.

867 76. Tittel-Elmer M, Bucher E, Broger L, Mathieu O, Paszkowski J, Vaillant I. Stress-Induced
868 Activation of Heterochromatic Transcription. *PLOS Genet*. Public Library of Science; 2010;
869 doi: 10.1371/journal.pgen.1001175.

77. Wiggins G. Larvae of the North American Caddisfly Genera (Trichoptera). Larvae North Am. Caddisfly Genera Trichoptera. University of Toronto Press;

78. Wiggins GB, Information CI for S and T, Museum RO. Caddisflies: The Underwater Architects. University of Toronto Press;

79. Dijkstra K-DB, Monaghan MT, Pauls SU. Freshwater biodiversity and aquatic insect diversification. *Annu Rev Entomol*. 2014; doi: 10.1146/annurev-ento-011613-161958.

80. Kordiš D, Lovšin N, Gubenšek F. Phylogenomic Analysis of the L1 Retrotransposons in Deuterostomia. *Syst Biol*. 2006; doi: 10.1080/10635150601052637.

81. Warren IA, Naville M, Chalopin D, Levin P, Berger CS, Galiana D, et al.. Evolutionary impact of transposable elements on genomic diversity and lineage-specific innovation in vertebrates. *Chromosome Res*. 2015; doi: 10.1007/s10577-015-9493-5.

82. Suh A, Churakov G, Ramakodi MP, Platt RN II, Jurka J, Kojima KK, et al.. Multiple Lineages of Ancient CR1 Retroposons Shaped the Early Genome Evolution of Amniotes. *Genome Biol Evol*. 2015; doi: 10.1093/gbe/evu256.

83. Grandi FC, An W. Non-LTR retrotransposons and microsatellites. *Mob Genet Elem*. Taylor & Francis; 2013; doi: 10.4161/mge.25674.

84. Mackay TFC. Transposable elements and fitness in Drosophila melanogaster. *Genome*. NRC Research Press Ottawa, Canada; 2011; doi: 10.1139/g89-046.

85. Pasyukova EG, Nuzhdin SV, Morozova TV, Mackay TFC. Accumulation of Transposable Elements in the Genome of Drosophila melanogaster is Associated with a Decrease in Fitness. *J Hered*. 2004; doi: 10.1093/jhered/esh050.

86. Langley CH, Montgomery E, Hudson R, Kaplan N, Charlesworth B. On the role of unequal exchange in the containment of transposable element copy number. *Genet Res*. Cambridge University Press; 1988; doi: 10.1017/S0016672300027695.

87. Hollister JD, Gaut BS. Epigenetic silencing of transposable elements: A trade-off between reduced transposition and deleterious effects on neighboring gene expression. *Genome Res*. 2009; doi: 10.1101/gr.091678.109.

88. Lee YCG, Karpen GH. Pervasive epigenetic effects of Drosophila euchromatic transposable elements impact their evolution. Nordborg M, editor. *eLife*. eLife Sciences Publications, Ltd; 2017; doi: 10.7554/eLife.25762.

89. Kraaijeveld K. Genome Size and Species Diversification. *Evol Biol*. 2010; doi: 10.1007/s11692-010-9093-4.

90. Cosby RL, Chang N-C, Feschotte C. Host–transposon interactions: conflict, cooperation, and cooption. *Genes Dev*. 2019; doi: 10.1101/gad.327312.119.

91. Hardie DC, Hebert PD. Genome-size evolution in fishes. *Can J Fish Aquat Sci*. NRC Research Press Ottawa, Canada; 2011; doi: 10.1139/f04-106.

92. Rees DJRJ, Dufresne FD, Glémet HG, Belzile CB. Amphipod genome sizes: first estimates for Arctic species reveal genomic giants. *Genome*. 2007; doi: 10.1139/G06-155.

93. Dufresne F, Jeffery N. A guided tour of large genome size in animals: what we know and where we are heading. *Chromosome Res*. 2011; doi: 10.1007/s10577-011-9248-x.

94. Lertzman-Lepofsky G, Mooers AØ, Greenberg DA. Ecological constraints associated with genome size across salamander lineages. *Proc R Soc B Biol Sci*. Royal Society; 2019; doi: 10.1098/rspb.2019.1780.

95. Vinogradov AE. Larger genomes for molluskan land pioneers. *Genome*. 2000; doi: 10.1139/g99-063.

96. Miller SA, Dykes DD, Polesky HF. A simple salting out procedure for extracting DNA from human nucleated cells. *Nucleic Acids Res*. 16:12151988;

97. Bankevich A, Nurk S, Antipov D, Gurevich AA, Dvorkin M, Kulikov AS, et al.. SPAdes: A New Genome Assembly Algorithm and Its Applications to Single-Cell Sequencing. *J Comput Biol*. 2012; doi: 10.1089/cmb.2012.0021.

98. Cantarel BL, Korf I, Robb SMC, Parra G, Ross E, Moore B, et al.. MAKER: an easy-to-use annotation pipeline designed for emerging model organism genomes. *Genome Res*. 2008; doi: 10.1101/gr.6743907.

99. Holt C, Yandell M. MAKER2: an annotation pipeline and genome-database management tool for second-generation genome projects. *BMC Bioinformatics*. 2011; doi: 10.1186/1471-2105-12-491.

100. Luo S, Tang M, Frandsen PB, Stewart RJ, Zhou X. The genome of an underwater architect, the caddisfly Stenopsyche tienmushanensis Hwang (Insecta: Trichoptera). *GigaScience*. 2018; doi: 10.1093/gigascience/giy143.

101. Haas BJ, Salzberg SL, Zhu W, Pertea M, Allen JE, Orvis J, et al.. Automated eukaryotic gene structure annotation using EVidenceModeler and the Program to Assemble Spliced Alignments. *Genome Biol*. 2008; doi: 10.1186/gb-2008-9-1-r7.

102. Lowe TM, Chan PP. tRNAscan-SE On-line: integrating search and context for analysis of transfer RNA genes. *Nucleic Acids Res*. 2016; doi: 10.1093/nar/gkw413.

103. Camacho C, Coulouris G, Avagyan V, Ma N, Papadopoulos J, Bealer K, et al.. BLAST+: architecture and applications. *BMC Bioinformatics*. 2009; doi: 10.1186/1471-2105-10-421.

104. Götz S, García-Gómez JM, Terol J, Williams TD, Nagaraj SH, Nueda MJ, et al.. High-throughput functional annotation and data mining with the Blast2GO suite. *Nucleic Acids Res*. 2008; doi: 10.1093/nar/gkn176.

105. Katoh K, Kuma K, Toh H, Miyata T. MAFFT version 5: improvement in accuracy of multiple sequence alignment. *Nucleic Acids Res*. 2005; doi: 10.1093/nar/gki198.

106. Kalyaanamoorthy S, Minh BQ, Wong TKF, von Haeseler A, Jermiin LS. ModelFinder: fast model selection for accurate phylogenetic estimates. *Nat Methods*. Nature Publishing Group; 2017; doi: 10.1038/nmeth.4285.

107. Minh BQ, Schmidt HA, Chernomor O, Schrempf D, Woodhams MD, von Haeseler A, et al.. IQ-TREE 2: New Models and Efficient Methods for Phylogenetic Inference in the Genomic Era. *Mol Biol Evol*. 2020; doi: 10.1093/molbev/msaa015.

947 108. Hoang DT, Chernomor O, von Haeseler A, Minh BQ, Vinh LS. UFBoot2: Improving the
948 Ultrafast Bootstrap Approximation. *Mol Biol Evol*. 2018; doi: 10.1093/molbev/msx281.

949 109. Zhang C, Rabiee M, Sayyari E, Mirarab S. ASTRAL-III: polynomial time species tree
950 reconstruction from partially resolved gene trees. *BMC Bioinformatics*. 2018; doi:
951 10.1186/s12859-018-2129-y.

952 110. Otto F. DAPI staining of fixed cells for high-resolution flow cytometry of nuclear DNA.
953 *Methods Cell Biol*. 1990; doi: 10.1016/s0091-679x(08)60516-6.

954 111. Dolezel J, Binarova P, Lucretti S. Analysis of Nuclear DNA content in plant cells by
955 Flow cytometry. *Biol Plant*. 1989; doi: 10.1007/BF02907241.

956 112. Ranallo-Benavidez TR, Jaron KS, Schatz MC. GenomeScope 2.0 and Smudgeplot for
957 reference-free profiling of polyploid genomes. *Nat Commun*. 2020; doi: 10.1038/s41467-020-
958 14998-3.

959 113. Wickham H. ggplot2: Elegant Graphics for Data Analysis. 2nd ed. Springer International
960 Publishing;

961 114. Flynn JM, Hubley R, Goubert C, Rosen J, Clark AG, Feschotte C, et al..
962 RepeatModeler2 for automated genomic discovery of transposable element families. *Proc*
963 *Natl Acad Sci*. National Academy of Sciences; 2020; doi: 10.1073/pnas.1921046117.

964 115. Novák P, Neumann P, Pech J, Steinhaisl J, Macas J. RepeatExplorer: a Galaxy-based
965 web server for genome-wide characterization of eukaryotic repetitive elements from next-
966 generation sequence reads. *Bioinformatics*. 2013; doi: 10.1093/bioinformatics/btt054.

967 116. Novák P, Ávila Robledillo L, Koblížková A, Vrbová I, Neumann P, Macas J. TAREAN:
968 a computational tool for identification and characterization of satellite DNA from
969 unassembled short reads. *Nucleic Acids Res*. 2017; doi: 10.1093/nar/gkx257.

970 117. Negm S, Greenberg A, Larracuente AM, Sproul JS. RepeatProfiler: a pipeline for
971 visualization and comparative analysis of repetitive DNA profiles. *bioRxiv*. Cold Spring
972 Harbor Laboratory; 2020; doi: 10.1101/2020.05.22.111252.

973 118. Goubert C, Modolo L, Vieira C, ValienteMoro C, Mavingui P, Boulesteix M. De Novo
974 Assembly and Annotation of the Asian Tiger Mosquito (Aedes albopictus) Repeatome with
975 dnaPipeTE from Raw Genomic Reads and Comparative Analysis with the Yellow Fever
976 Mosquito (Aedes aegypti). *Genome Biol Evol*. 2015; doi: 10.1093/gbe/evv050.

977 119. Barker MS, Dlugosch KM, Dinh L, Challa RS, Kane NC, King MG, et al.. EvoPipes.net:
978 Bioinformatic Tools for Ecological and Evolutionary Genomics. *Evol Bioinforma*. SAGE
979 Publications Ltd STM; 2010; doi: 10.4137/EBO.S5861.

980 120. Birney E, Clamp M, Durbin R. GeneWise and Genomewise. *Genome Res*. 2004; doi:
981 10.1101/gr.1865504.

982 121. Yang Z. PAML 4: Phylogenetic Analysis by Maximum Likelihood. *Mol Biol Evol*. 2007;
983 doi: 10.1093/molbev/msm088.

122. Cui L, Wall PK, Leebens-Mack JH, Lindsay BG, Soltis DE, Doyle JJ, et al.. Widespread genome duplications throughout the history of flowering plants. *Genome Res*. 2006; doi: 10.1101/gr.4825606.

123. Benaglia T, Chauveau D, Hunter DR, Young DS. mixtools: An R Package for Analyzing Mixture Models. *J Stat Softw*. 2009; doi: 10.18637/jss.v032.i06.

124. Weigand H, Weiss M, Cai H, Li Y, Yu L, Zhang C, et al.. Fishing in troubled waters: Revealing genomic signatures of local adaptation in response to freshwater pollutants in two macroinvertebrates. *Sci Total Environ*. 2018; doi: 10.1016/j.scitotenv.2018.03.109.

125. Ferguson L, Marlétaz F, Carter J-M, Taylor WR, Gibbs M, Breuker CJ, et al.. Ancient Expansion of the Hox Cluster in Lepidoptera Generated Four Homeobox Genes Implicated in Extra-Embryonic Tissue Formation. *PLOS Genet*. Public Library of Science; 2014; doi: 10.1371/journal.pgen.1004698.

126. Weigand H, Weiss M, Cai H, Li Y, Yu L, Zhang C, et al.. Deciphering the origin of mito-nuclear discordance in two sibling caddisfly species. *Mol Ecol*. 2017; doi: 10.1111/mec.14292.

Table 1. Comparison of assembly and annotation statistics of all available Trichoptera Genomes. *Assemblies produced in this study. **$N_{Arthropoda}$=2442, % of present BUSCOS = complete + fragmented.

**Species**	**Abbre-viation**	**Accession number**	**Length (bp)**	**N50 (kbp)**	**No. of contigs**	**BUSCOS %****
*Agapetus fuscipes**	AF	JAGTXP000000000	552,637,417	2.8	291,536	75.5
*Agraylea sexmaculata**	AS	JAGTTH000000000	196,044,125	86	7,050	96.5
Agrypnia vestita [30]	AV	JADDOH000000000	1,352,945,503	111.8	25,541	92.8
*Drusus annulatus**	DA	JAGWCC000000000	727,941,535	1,043.7	2,401	96.8
*Glossosoma conforme**	GC1	JAGTXR000000000	568,249,599	2,212.1	653	91.8
Glossosoma conforme [124]	GC2	GCA_003347265.1	604,293,666	17.1	119,821	92.1
Glyphotaelius pellucidula [125]	GP	Glyphotaelius_pellucidus_k51_scaffolds	623,431,006	1.6	461,749	68.6
*Halesus radiatus**	HR	JAHDVE000000000	973,356,502	125.2	12,484	90.6
*Himalopsyche phryganea**	HP	JAGVSL000000000	633,785,554	4,634	710	97.8
Hesperophylax magnus [30]	HM	JADDOG000000000	1,275,967,528	768.2	6,877	95.2
Hydropsyche tenuis [57]	HT	GCA_009617725.1	229,663,394	2,190.1	403	97.6
*Lepidostoma basale**	LB	JAGTTH000000000	769,208,668	1,052	1,621	97.0
Limnephilus lunatus [69]	LL	GCA_000648945.2	1,369,180,260	69.1	58,718	91
*Micrasema longulum**	ML2	JAGXCS000000000	668,600,304	2.5	368,330	69.8
*Micrasema longulum**	ML1	JAGVSM000000000	585,245,295	170.5	5,451	84.5
*Micrasema minimum**	MM	JAGVSQ000000000	329,257,313	69.5	7,561	67.1
*Micropterna sequax**	MS	JAGUCF000000000	778,692,278	7.9	144,286	68.9
*Odontocerum albicorne**	OA	JAGTXQ000000000	1,305,984,461	266.4	9,303	95.9
*Parapsyche elsis**	PE	JAGVSN000000000	282,185,525	5,591.7	159	97.4
*Philopotamus ludificatus**	PL	JAGXCT000000000	360,300,449	67.5	37,274	92.6
Plectrocnemia conspersa [57]	PC	GCA_009617715.1	396,695,105	869	1,614	97.8
*Rhyacophila brunnea**	RB	JAGYXB000000000	1,086,872,538	1,030.6	2,125	97.3
*Rhyacophila evoluta**	RE2	JAGVSQ000000000	565,830,460	9.9	114,057	91.7

*Rhyacophila evoluta**	RE1	JAGVSO000000000	562,550,625	9.7	111,706	91.7
Sericostoma sp.[126]	SS	GCA_003003475.1	1,015,727,762	3.2	561,698	60.3
Stenopsyche tienhuanesis [100]	ST	GCA_008973525.1	451,494,475	1,296.7	552	97.6
