# Supplementary material for: Genome size evolution in the diverse insect order Trichoptera
Source: Gigascience. 2022 Feb 25;11:giac011. doi: 10.1093/gigascience/giac011 (PMC8881205; doi:10.1093/gigascience/giac011)
Supplement: giac011_GIGA-D-21-00216_Revision_1 [file giac011_giga-d-21-00216_revision_1.pdf]

GigaScience

Genome size evolution in the diverse insect order Trichoptera

--Manuscript Draft--

**Manuscript Number:**	GIGA-D-21-00216R1
**Full Title:**	Genome size evolution in the diverse insect order Trichoptera
**Article Type:**	Research
**Funding Information:**	national science foundation (DBI-1811930) — Dr. John S. Sproul<br>national institutes of health (R35GM119515) — Dr. Amanda M. Larracuente<br>brigham young university — Dr. Paul B. Frandsen<br>hessen state ministry of higher education, research and the arts — Dr. Jacqueline Heckenhauer<br>hessisches ministerium für wissenschaft und kunst — Dr. Jacqueline Heckenhauer<br>Dr. Steffen U. Pauls<br>army research office (W911NF-13-1-0319) — Dr. Russell J. Stewart
**Abstract:**	Background<br><br>Genome size is implicated in form, function, and ecological success of a species. Two principally different mechanisms are proposed as major drivers of eukaryotic genome evolution and diversity: Polyploidy (i.e., whole genome duplication: WGD) or smaller duplication events and bursts in the activity of repetitive elements (RE). Here, we generated de novo genome assemblies of 17 caddisflies covering all major lineages of Trichoptera. Using these and previously sequenced genomes, we use caddisflies as a model for understanding genome size evolution in diverse insect lineages.<br><br>Results<br><br>We detect a ~14-fold variation in genome size across the order Trichoptera. We find strong evidence that repetitive element (RE) expansions, particularly those of transposable elements (TEs), are important drivers of large caddisfly genome sizes. Using an innovative method to examine TEs associated with universal single copy orthologs (i.e., BUSCO genes), we find that TE expansions have a major impact on protein-coding gene regions, with TE-gene associations showing a linear relationship with increasing genome size. Intriguingly, we find that expanded genomes preferentially evolved in caddisfly clades with a higher ecological diversity (i.e., various feeding modes, diversification in variable, less stable environments).<br><br>Conclusion<br><br>Our findings provide a platform to test hypotheses about the potential evolutionary roles of TE activity and TE-gene associations, particularly in groups with high species, ecological, and functional diversities.
**Corresponding Author:**	Jacqueline Heckenhauer, Ph.D.<br>LOEWE-TBG: LOEWE-Zentrum fur Translationale Biodiversitatsgenomik<br>Frankfurt am Main, Hesse GERMANY
**Corresponding Author Secondary Information:**	
**Corresponding Author's Institution:**	LOEWE-TBG: LOEWE-Zentrum fur Translationale Biodiversitatsgenomik
**Corresponding Author's Secondary Institution:**	
**First Author:**	Jacqueline Heckenhauer, Ph.D.
**First Author Secondary Information:**	

Powered by Editorial Manager® and ProduXion Manager® from Aries Systems Corporation

**Order of Authors:**	Jacqueline Heckenhauer, Ph.D.
	Paul B. Frandsen
	John S. Sproul
	Zheng Li
	Juraj Paule
	Amanda M. Larracuente
	Peter J. Maughan
	Michael S. Barker
	Julio V. Schneider
	Russell J. Stewart
	Steffen U. Pauls
**Order of Authors Secondary Information:**	
**Response to Reviewers:**	Dear Hongling Zhou, and, dear Editorial Board,<br>We would like to thank you for the opportunity to revise our manuscript and respond to the important points raised by qualified reviewers.<br>We would like to re-submit our revised manuscript entitled “Genome size evolution in the diverse insect order Trichoptera (caddisflies)” for review after incorporating feedback from both reviewers.<br>We thank both reviewers for their insightful critiques and interest in our manuscript, which nearly triples the number of available caddisfly genomes, explores the role of transposable element expansions in genome size evolution of caddisflies and provides a new method for detection of TE sequence associations with protein-coding genes. Since both reviewers stated that they found the description of this new method difficult to understand, we undertook a substantial revision to the relevant sections in the manuscript.<br>Another suggestion of one reviewer was to conduct further analyses to investigate the role of WGD events in the evolution of genome size in caddisflies. As recommended by the reviewer, we ran Smudgeplot and did not detect evidence for polyploidy or WGD events. While this, of course, does not mean that we can completely rule out that WGD plays a role in genome size evolution in Trichoptera, the bulk of the evidence from our study suggests that repetitive elements played the most important role and we focus primarily on their role in this study. We reformulated the discussion to emphasize this.<br><br>The revised manuscript addresses the concerns raised and was submitted via the submission portal (with and without using Track Changes). A point-by-point response to the reviewers’ suggestions can be found below.<br>Again, we thank you for the opportunity to revise our manuscript. Please let us know if there is anything else you require from us.<br><br>Sincerely,<br><br>Jacqueline Heckenhauer on behalf of all authors<br><br>Point-by-point 'Response to Reviewers':<br><br>Reviewer reports:<br><br>Reviewer #1: Summary of the paper and overall impression<br>In their paper, "Genome size evolution in the diverse insect order Trichoptera", Heckenhauer et al report a 14-fold variation in genome size in caddis-flies. The authors find evidence for increases in transposable elements associated with larger genomes, and report that in caddis-flies living in less stable environments, some genes are replicated in association with transposable elements. Overall, this paper represents a comprehensive collection of data, however, I have some concerns about some of the reporting of methods, some analyses and conclusions. To support some of the conclusions, namely that WGD or large-scale duplications do not play a role in caddis fly genomes, I believe the authors could perform additional analyses. Further, I was left

confused by the descriptions of the methods, especially around the replicated BUSCO gene analyses. Please see my comments below.

Response: We thank the reviewer for their valuable comments and suggestions. We address these below.

Main comments:
1. The authors report that their gene-age distribution analyses do not support the hypothesis of a WGD, but given previous suggestions that WGD are important in these species, the authors should conduct additional analyses (e.g smudgeplot, minor allele frequency distributions in single-copy genes) to rule out this possibility. While it can indeed be difficult to find a balance between the evidence of absence and an absence of evidence, more effort should go into resolving the matter of WGD in caddis-flies. Some of the genomescope peaks, and some of the coverage peaks from the backmap approach seem to at least hint at large-scale duplications or variations in copy number. Further analyses should also consider if assembled gene copies may be collapsed duplicates.

Response: We appreciate this critique and conducted additional analyses to detect WGD events. As suggested by the reviewer, we ran Smudgeplot on all genomes. This is described in detail in the main text (lines 294-304, 323-326, 577-587, supplementary note 9) and smudgeplots are given in the supplementary material (Figs. S74-121). The new, additional analyses, did not uncover evidence for WGD. This does, of course, not mean that we can entirely rule out that WGD played a role in genome size evolution in Trichoptera. However, we do find strong evidence that repetitive elements played a key role in genome size expansion in this study. We reformulated this in the discussion accordingly (lines 323-331). We hope that further studies using chromosome-length assemblies might help to resolve the matter of WGD in caddisflies. However, generating these is out of the scope of this present study. The emergence of PacBio HiFi genomes of caddisflies (e.g., Darwin Tree of Life is currently planning to sequence 28 caddisfly genomes) might be useful to further test the role of WGD events in the evolution of Trichoptera. We have emphasized this in the revised version of the manuscript (lines lines 323-331).

2. I admit I am confused by the terminology around the TE-associated BUSCO genes. Are these cases where BUSCO has reported a high number of duplicates? Or where BUSCO annotated regions have a high coverage? Two things need to be clarified here; what made them stick out in the first place (coverage? Duplications?), and what are they really (TEs that BUSCO mistook for BUSCOs? fragments of real BUSCOs attached to TEs?).

Response: We thank the reviewer for expressing this confusion and suggesting points that need clarification. To address both reviewers' comments (see also reviewer 2, main comment 1), we thoroughly reworked our presentation of the TE-associated BUSCO analysis and results to help readers to understand the concept of this new discovery (lines 234–281, 536-559, 639-656, supplementary note 11).

To answer Reviewer 1's questions here: (1) These BUSCOs first stuck out because we had mapped reads to all BUSCOs as part of our early data exploration and noted that a subset of BUSCO genes had sequence regions with unexpectedly high coverage when visualizing read depth profiles, and this was especially pronounced in species with large genome sizes. (2) The high coverage regions turned out to be fragments of repetitive elements (especially transposable elements) inserted within or immediately adjacent to BUSCOs, and incorrectly annotated by the BUSCO algorithm. Most of our methods/results, which caused some confusion to both reviewers were describing methods used to validate that the high coverage regions do, in fact, consist of TE-derived sequences.

We improved our presentation of this topic in three ways. First, in our presentation of this topic (lines 234–244), we now lead off by answering both questions raised by Reviewer 1 above up front. Only then do we go into more detail (see pasted text below). Second, we use more effective and standardized terminology throughout. Instead of using the somewhat vague/misleading term, "repetitive BUSCOs", we use the more correct and descriptive term "TE-associated BUSCOs" throughout the text

	and in Fig.4. Third, in supplementary note 11, we integrated results alongside methods so that the flow of methodological logic and the results from each intermediate analysis are integrated and all in one place. In addition to these major edits, we include minor adjustments to the text in lines noted above. We feel the manuscript is much improved through addressing both reviewers' feedback on this point.<br><br>(**pasted from lines 234–244) "During early exploration of our sequence data, we made an unexpected discovery that in some lineages, universal single copy orthologs, or "BUSCO genes," showed higher than expected coverage depth of mapped reads in one or more of their sequence fragments. Further analysis showed that these high-coverage BUSCO sequence regions are typically RE sequences (primarily TEs) that are either embedded within or located immediately adjacent to BUSCO genes, such that the BUSCO algorithm includes them in its annotation of a given gene. We refer to BUSCO genes containing these putative RE fragments as 'TE-associated BUSCOs' (supplementary Fig. S125, supplementary note 11). By estimating how many times they occur, we can quantitatively measure how TE-gene interactions change with changing genome size. In fact, we detected a positive linear relationship between TE-gene interactions and increasing genome size when measured with this metric."<br><br>Minor comments:<br>1. Lines 53-57: "Genome size can vary widely among closely and distantly related species, though our knowledge is still scarce for many non-model groups. This is especially true for insects, which account for much of the earth's species diversity. To date 1,345 insect genome size estimates have been published, representing less than 0.15% of all described insect species." While I appreciate the authors' point that there is a relatively little data available about genome size and only a small proportion of non-model insects in the Animal Genome Size database, this is the case for all groups, and insects actually represent the largest group of invertebrates in the AGSDb. However, this does not mean insects, or chironomid are a poor system to study this in, so authors could reframe this first sentence to justify the study system with something more than highlighting how understudied this is in insects.<br><br>Response: This is an important point. We reformulated the beginning of the background section (lines 54-61) as follows: "Genome size is a fundamental biological character. Studying its evolution may potentially lead to a better understanding of the origin and underlying processes of the myriad forms and functions of plants and animals, subjects which remain at the core of much biological research. Given their high species, ecological and functional diversities, insects are excellent models for such research. To date 1,345 insect genome size estimates have been published (Gregory, 2005: Animal Genome Size Database: http://www.genomesize.com, last accessed 2021-04-30) ranging 240-fold from 69 Mbp in chironomid midges [1] to 16.5p Gbp in the mountain grasshopper Podisma pedestris [2].<br><br>2. Line 76: correct to "In insects, the KNOWN ranges of genomic repeat proportion are…"<br><br>Response: We agree and corrected this sentence (lines 76-78): "In insects, the known genomic proportion of TEs ranges from 1% in the antarctic midge Belgica antarctica [26] to 65% in the migratory locust Locusta migratoria [27]."<br><br>3. Lines 89-91: Why are species rich groups a better system to study RE evolution and environmental interactions than e.g. populations, species complexes, recently diverged species, or groups in the process of speciation?<br><br>Response: Thank you for raising this point. As the reviewer points out we didn't present the most effective logical argument here. We updated this section (lines 87-95) to better represent our logic as follows: "Investigating RE dynamics in diverse clades provides a powerful lens for understanding their roles in genome function and evolution. Broadly studying of RE dynamics in species-rich groups with wide variation in RE activity is an important step towards efficiently identifying study systems at finer taxonomical scales (natural populations, species complexes, or recently diverged species) that are ideally suited to advance our understanding of molecular and evolutionary mechanisms underlying genome evolution. In addition, by taking this biodiversity genomics approach, we can develop new model systems and eventually

better understand links between environmental factors, genome size evolution, adaptation, and speciation (see [47]).”

4. Lines 113-115: The data description does not, in my opinion, need to justify the species selection since this is done in the intro

Response: Thanks for this suggestion. We deleted the first two sentences of the data description (line 114).

5. Genome size estimates- sequencing based estimates can also be impacted by GC-content, especially in libraries which were produced using PCR, this may be a useful point regarding the differences between FCM and sequencing-based estimates

Response: We added this point in the main text (lines 164-166).

6. RepMod versions inconsistent Line 463 says v2, earlier says v1

Response: We used RepeatModeler2.0 and corrected the text in line 439.

7. Line 468-469- What did you use to merge repmask out files?

Response: To merge the repeatmasker out files, we parsed the output tables from each of the two repeatmasker runs with a script (RM_table_parser_families_.py) and then combined the resulting data columns for the two runs in Excel. We added this information in lines 507-510. The script has been uploaded to https://github.com/jhcaddisfly/TE-gene_intersect_analysis.

8. All read-based analyses: were they run on decontaminated read libraries? If so, please briefly clarify this in the main manuscript. Genome size with GenomeScope: 444-448; RepeatExplorer: Lines 471-479

Response: Yes, all read-based analyses were run on decontaminated read libraries. We clarified this in lines 483 and 517.

9. Why only use dnaPipeTE for repeat divergences and not also abundances? Does dnaPipeTE agree with RepeatExplorer?

Response: As suggested, we now include dnaPipeTE-based abundances, as well. The results were overall congruent to those obtained by RepeatMasker and RepeatExplorer2 and the primary message of our manuscript is consistent among all three methods. Compared to RepeatExplorer, dnaPipeTE resulted in more thorough annotation of DNA transposons (which were very uncommon in the RepeatExplorer output, but commonly annotated in our primary approach with RepeatModeler/RepeatMasker), simple repeats, Helitrons, and some other categories. This results in a smaller proportion of unclassified repeats compared to RepeatExplorer. We note that there is a stylistic difference in output between the methods. The dnaPipeTE plot (supplementary note 10, Figure S123) has more repeat categories shown, and the categories are in a different order. However, for shared categories, we use the same color scheme between both plots. We adjusted the main text to include dnapipeTE (lines 208-210, 511-512, 520) and added the results to supplementary note 10, and supplementary figure S123.

10. Line 495: What is meant by "BUSCO genes showed regions of unexpected high copy number…"? Are these genes reported by BUSCO as duplicated or is this referring to increased coverage?

Response: Here, “copy number” referred to coverage depths. We updated the language to clarify. The updated language is pasted in our previous response above (see response above, reviewer 1, main comment 2).

11. Lines 506-507: "We used copy number profiles to identify BUSCO genes with repetitive sequences based on coverage profiles" The meaning of this is unclear. The reported copy number from BUSCO? Coverage of mapped reads?

Powered by Editorial Manager® and ProduXion Manager® from Aries Systems Corporation

Response: The copy number profiles were generated through mapping reads back to BUSCO sequences and are not reported in BUSCO output. We clarified this text in response to an earlier comment from the reviewer (see response above to reviewer 1, main comment 2).

12. Table 1- please report the full BUSCO summary (e.g. C:39.7%[S:39.2%,D:0.5%],F:35.8%,M:24.5%,n:2442) for each species, lumping complete and fragmented together is unneccesary, and readers are usually interested enough in the full complement of BUSCOs that it should not be in the supplements, but in the main paper

Response: We adjusted table 1 according to the reviewer's suggestion.

13. Coverages from backmap method can and should be compared to genomescope kcov estimates (while correcting for kmer size; see here for a brief explanation https://www.biostars.org/p/221672/), this will validate both approaches and offer further evidence when considering polploidy.

Response: We hope that we understand the reviewer's suggestion correctly. We did not compare mapping coverage of the backmap method with the Genomescope 2 kcov estimates. We think that it is important to make a fundamental distinction between these values since these are derived from two different methods/algorithms. However, we agree that it is interesting to compare kcov estimates between Genomescope2 and smudgeplots. We did this and the results are given in table "DataS1_Sup.8-Comparison_GenomeScope2_Smudgeplots".

14. In the supplementary note about TAGC plots, Figures S31, S36, S38, S44, S45, S46, S47 don't list contaminant exclusion criteria- if contaminants weren't removed this needs to be stated, and in some cases, especially those where there are different "blobs", (e.g. S47) justified

Response: We only classified contigs with taxonomic assignment to phyla other than Arthropoda as contaminated, when these also showed different GC content and coverage. The taxonomic assignment may be not as expected because a) some contigs are just too small to be recognized correctly and b) there might be conflict in the assignment and therefore Blobtools just declares these contigs as no-hit (see "taxrule" in Blobtools manual). A valid reason for not excluding any contigs of assemblies shown in figures S31, S36, S38, S44, S45, S46, S47 is that in every "fraction" of the blobs there are contigs assigned to arthropods. Moreover, after uploading the genomes to NCBI, the NCBI genome assembly processing pipeline served as a second quality control step. In a few cases (e.g., S38, S44, S45) NCBI's contamination screen identified additional contamination. These were filtered out manually or by NCBI. We added this information to supplementary note 5.

15. Supplementary note 9: Figure reference is wrong?

Response: We have corrected the Figure reference in supplementary note 10 (before supplementary note 9).

16. Supplementary note 10: Can coverage comparisons using average BUSCO coverage be re-run using corrected kcov estimates? This would validate the BUSCO coverage approach.

Response: Our understanding from this comment is that there is a lack of clarity in describing what we mean by repetitive BUSCOs / and average BUSCO coverage. We reformulated these definitions in the main text (see respond to reviewer 1, main comment 2).

17. Supp Data 1: Coverage estimates would be more accurate if based on FCM measurements and total sequenced bp (before and after decontamination) and can also be compared to corrected kcov estimates

Response: We recalculated sequencing coverage based on total sequenced base

pairs before and after decontamination as well as FCM measurements if these were available. For sequences without FCM, we used the Genomescope2 genome size estimates as a basis for coverage calculation (“DataS1_Sup.2-Assembly_statistics”, “DataS1_Sup.3_Sequencing_coverages”). These results, as well as the results of the corrected kcov estimates obtained by Genomescope2 and smudgeplots are given in “DataS1_Sup.8-Comparison_GenomeScope2_Smudgeplots”.

18. Limnephilus lunatus has too low coverage to get reliable genomescope

Response: We agree and exclude the results from Limnephilus lunatus from Genomescope2 as well as from the backmap approach. We also realized that the coverage of Glyphothaelius was too low for backmap.pl. In addition, we realized that for previously published genomes (Sericostoma, Stenopsyche, Glososssoma), we used all short-read used in the assembly to estimate genome sizes backmap.pl. However, since these belong to multiple individuals, we reran the contamination filtering, backmap.pl on only reads from one individual (see “DataS1_Sup.3_Sequencing_coverage” for accession number of reads used) belonged to multiple individuals. However, the resulting genome size estimates did not change much. We updated the results in the supplementary files, rerun the Bland-Altman-analysis and modified figure 1.

Reviewer #2: This paper presents 17 new insect genomes from the order of caddisflies (Trichoptera). The authors combine these genomes with 9 previously sequenced genomes to analyze genome size evolution across the order. They find that genome size tends to correlate with evolution of repeat elements, specifically expansion of transposable elements (TEs). Interestingly, the authors also notice that TE expansions also correlate with gene copy-number (or gene fragment copy-number), even of highly conserved genes used to assess genome completeness. Overall, I find this paper very well written and easy to follow. The genomic resources and analyses presented provide novel new resources and findings for insects in the order Trichoptera, with potential implications beyond. I have only minor suggestions before publication, outlined below.

Response: We thank the reviewer for the kind comments, we are pleased to hear that our manuscript was easy to follow and our new resources and findings are appreciated.

1. Regarding the TE and BUSCO gene fragment associations, while I think this is a really interesting analysis, I found the underlying models a bit difficult to understand. Line 236 reads, "To test whether repetitive fragments were due to TE insertions near or in the BUSCO genes or, conversely, due to the proliferation of 'true' BUSCO protein-coding gene fragments…"
Is the idea that a BUSCO gene has been duplicated itself and then one copy is either fragmented by a TE insertion or hitch-hikes with a TE (as mentioned on line 501)? Or are these fragments only of BUSCO genes that didn't match a full BUSCO gene at all, but the fragments that did match had unexpectedly high coverage? I guess I'm just confused as to whether a gene duplication needs to precede the TE insertions/hitch-hiking, which is subsequently pseudogenized either prior to or because of the TE activity, or if these are gene losses. I understand how the TE could inflate the coverage of these fragments, but I guess I'm still not clear on how these fragments arise in the first place. Any clarification would be helpful! Also, if the case is that these are fragments of BUSCO genes that have no full matches in the genome, how might assembly contiguity or quality be affecting these matches?

Response: Thank you for raising this point, which was also raised by reviewer 1. As described in our response to Reviewer 1 (main comment 2), we have thoroughly revised our presentation of the TE-associated BUSCO gene analysis/results to clarify points raised by both reviewers. The revised text we pasted in our response is pasted again below with additional text from the manuscript and addresses the reviewer’s question about how these fragments arise in the first place.
Regarding the additional point on assembly quality raised by the reviewer here, we considered potential impact of the assembly quality on our conclusion on the TE-gene association study. One line of evidence that suggests assembly artefacts are not driving the pattern of increasing TE-associated BUSCOs with increasing assembly size

is the fact that in less contiguous assemblies we found notably fewer TE-associated BUSCOs (including in replicate conspecifics with varying assembly quality). If assembly artefacts in low-quality assemblies were driving the abundance of TE-associated BUSCOs we would have expected to find an increase of these with decreasing assembly quality. Moreover, when mapping Nanopore reads back to an exemplary subset of TE-associated BUSCOs, we observed that junctions between TE-associated and non- TE-associated regions are embedded in full reads and do not occur disproportionately at ends of reads. For this reason, we conclude that our analysis detected valid TE-gene associations and not simply assembly artefacts (this test is explained in detail in supplementary note 12).

(**pasted from lines 234–244) “During early exploration of our sequence data, we made an unexpected discovery that in some lineages, universal single copy orthologs, or “BUSCO genes,” showed higher than expected coverage depth of mapped reads in one or more of their sequence fragments. Further analysis showed that these high-coverage BUSCO sequence regions are typically RE sequences (primarily TEs) that are either embedded within or located immediately adjacent to BUSCO genes, such that the BUSCO algorithm includes them in its annotation of a given gene. We refer to BUSCO genes containing these putative RE fragments as ‘TE-associated BUSCOs’ (supplementary Fig. S125, supplementary note 11). By estimating how many times they occur, we can quantitatively measure how TE-gene interactions change with changing genome size. In fact, we detected a positive linear relationship between TE-gene interactions and increasing genome size when measured with this metric.” And from lines 251-254: “The BUSCO-associated sequences may represent TEs recently inserted into BUSCO genes, the remnants left behind following historical TE transposition events, or TE sequences that are immediately adjacent to and inadvertently classified as BUSCO sequences.”

2. One thing that I noticed throughout the figures is that branch B1, leading to A. sexmaculata, the branch leading to clade A, and the branch leading to clade B (as labeled in Figures 1 and 2) appear to form a polytomy. I don't find this mentioned in the text and am wondering why this relationship remains unresolved with these data. I don't think this has any bearing on the results, since all analyses are done on the tips of the tree, but I think readers looking at these trees will want to know what is going on at that node.

Response: As shown in supplementary S48, the position of the branch B1 leading to A. sexmaculata is not supported in this study. Both the local posterior probability (48) and the site/ gene concordance values (31, 35) show that there is conflicting signal in the single locus trees. Moreover, the internode length is very short. For this reason, we collapsed the tree in figures 1 and 2 in the main text. As written in the figure legends, we collapsed nodes with less than 0.9 local posterior probability. We adjusted this unresolved node according to Thomas et al. (2020). While this incongruence is interesting from a phylogenetic standpoint, more data is necessary to assess the phylogenetic position of Hydroptilidae (Figs. 1 & 2: Clade B1, supplementary Fig. S48: Agraylea sexmaculata) which remains underrepresented in genomic research.

3. The authors use custom scripts for their BUSCO-TE correlation analysis and provide a link to a Box folder on line 514. I would request that these scripts be put somewhere more stable and accessible (e.g., github). Not only was I asked to login when clicking the link, but after I had done so that link didn't seem to exist.

Response: We are sorry that the reviewer was unable to access the data. We created a repository for the TE-BUSCO gene intersect analysis including all custom scripts and a description of the whole pipeline: https://github.com/jhcaddisfly/TE-gene_intersect_analysis
Data supporting the results will be submitted to GigaDB and can be accessed under this link without logging in: https://byu.box.com/v/trich-genomes
Minor/editorial points
1. Would the authors be able to report concordance factors for the species tree? I think this should be easy enough with IQ-tree and is something I ask everyone to do. This may also help answer my question about the polytomy.

Response: We computed the concordance factors as described in lines 473-476 and

	included these in the supplementary figure S48. The position of the branch B1 is not supported (Astral support value: 48). The low site concordance (31) and gene concordance (35) values also suggest that is disconcordant signal in the single locus trees. For this reason, the position of A. sexmaculata remains unresolved.<br><br>2. The authors do a good job of mentioning and citing programs used throughout the manuscript but seem to skip this in the Assembly section (starting on Line 398). "First, we applied a long-read assembly method…" Which one? Same for "de novo hybrid assembly approaches." I see that assembly is covered in detail in the Supplement, but I think naming the main programs used (wbtdbg2 and Masurca) should be in the main text.<br><br>Response: We added the main programs used to the main text (lines 433, 436).<br><br>3. Line 281-282: I think some of the brackets and parentheses here are mismatched or un-closed.<br><br>Response: We corrected this (line 312).
**Additional Information:**	
**Question**	**Response**
Are you submitting this manuscript to a special series or article collection?	No
**Experimental design and statistics**<br><br>Full details of the experimental design and statistical methods used should be given in the Methods section, as detailed in our Minimum Standards Reporting Checklist. Information essential to interpreting the data presented should be made available in the figure legends.<br><br>Have you included all the information requested in your manuscript?	Yes
**Resources**<br><br>A description of all resources used, including antibodies, cell lines, animals and software tools, with enough information to allow them to be uniquely identified, should be included in the Methods section. Authors are strongly encouraged to cite Research Resource Identifiers (RRIDs) for antibodies, model organisms and tools, where possible.<br><br>Have you included the information requested as detailed in our Minimum	Yes

Standards Reporting Checklist?	
**Availability of data and materials**<br><br>All datasets and code on which the conclusions of the paper rely must be either included in your submission or deposited in publicly available repositories (where available and ethically appropriate), referencing such data using a unique identifier in the references and in the “Availability of Data and Materials” section of your manuscript.<br><br>Have you have met the above requirement as detailed in our Minimum Standards Reporting Checklist?	No
If not, please give reasons for any omissions below.<br><br>as follow-up to "**Availability of data and materials**<br><br>All datasets and code on which the conclusions of the paper rely must be either included in your submission or deposited in publicly available repositories (where available and ethically appropriate), referencing such data using a unique identifier in the references and in the “Availability of Data and Materials” section of your manuscript.<br><br>Have you have met the above requirement as detailed in our Minimum Standards Reporting Checklist?<br><br>"	Raw data and assemblies are available at NCBI under BioProject: PRJNA558902. Data supporting the results will be submitted to GigaDB and can be accessed under this link: https://app.box.com/folder/136985262731?v=trich-genomes

Powered by Editorial Manager® and ProduXion Manager® from Aries Systems Corporation

Manuscript - Main text Click here to access/download;Manuscript;Maintext.docx

Genome size evolution in the diverse insect order Trichoptera

Authors

Jacqueline Heckenhauer (jacqueline.heckenhauer@senckenberg.de)[1,2*], Paul B. Frandsen (paul_frandsen@byu.edu)[1,3,4], John S. Sproul (johnssproul@gmail.com)[5,6], Zheng Li (zheng.li@austin.utexas.edu)[7], Juraj Paule (juraj.paule@senckenberg.de)[8], Amanda M. Larracuente (alarracu@ur.rochester.edu)[5], Peter J. Maughan (jeff_maughan@byu.edu)[3], Michael S. Barker (msbarker@arizona.edu)[7], Julio V. Schneider (julio.schneider@senckenberg.de)[2], Russell J. Stewart (russell.stewart@utah.edu)[9], Steffen U. Pauls (steffen.pauls@senckenberg.de)[1,2,10]

Affiliations

[*]corresponding author

[1]LOEWE Centre for Translational Biodiversity Genomics (LOEWE-TBG), Frankfurt, Germany,

[2]Department of Terrestrial Zoology, Senckenberg Research Institute and Natural History Museum Frankfurt, Frankfurt, Germany

[3]Department of Plant & Wildlife Sciences, Brigham Young University, Provo, UT

[4]Data Science Lab, Smithsonian Institution, Washington, DC

[5]Department of Biology, University of Rochester, Rochester, NY, USA

[6]Department of Biology, University of Nebraska Omaha, Omaha, USA

[7]Department of Ecology and Evolutionary Biology, University of Arizona, Tucson, USA

[8]Department of Botany and Molecular Evolution, Senckenberg Research Institute and Natural History Museum Frankfurt, Frankfurt, Germany

[9]Department of Biomedical Engineering, University of Utah, Salt Lake City, UT

[10]Institute for Insect Biotechnology, Justus-Liebig-University, Gießen, Germany

Abstract

Background: Genome size is implicated in form, function, and ecological success of a species. Two principally different mechanisms are proposed as major drivers of eukaryotic genome evolution and diversity: Polyploidy (i.e., whole genome duplication: WGD) or smaller duplication events and bursts in the activity of repetitive elements (RE). Here, we generated *de novo* genome assemblies of 17 caddisflies covering all major lineages of Trichoptera. Using these and previously sequenced genomes, we use caddisflies as a model for understanding genome size evolution in diverse insect lineages.

Results: We detect a ~14-fold variation in genome size across the order Trichoptera. We find strong evidence that repetitive element (RE) expansions, particularly those of transposable elements (TEs), are important drivers of large caddisfly genome sizes. Using an innovative method to examine TEs associated with universal single copy orthologs (i.e., BUSCO genes), we find that TE expansions have a major impact on protein-coding gene regions, with TE-gene associations showing a linear relationship with increasing genome size. Intriguingly, we find that expanded genomes preferentially evolved in caddisfly clades with a higher ecological diversity (i.e., various feeding modes, diversification in variable, less stable environments).

Conclusion: Our findings provide a platform to test hypotheses about the potential evolutionary roles of TE activity and TE-gene associations, particularly in groups with high species, ecological, and functional diversities.

Key words:

biodiversity, *de novo* genome assembly, genomics, genomic diversity, genome duplication, genome size evolution, insects, repetitive elements, transposable elements, Trichoptera

Background

Genome size is a fundamental biological character. Studying its evolution may potentially lead to a better understanding of the origin and underlying processes of the myriad forms and functions of plants and animals. This diversification process remain at the core of much biological research. Given their high species, ecological and functional diversities, insects are excellent models for such research. To date 1,345 insect genome size estimates have been published (Gregory, 2005: Animal Genome Size Database: http://www.genomesize.com, last accessed 2021-04-30) ranging 240-fold from 69 Mbp in chironomid midges [1] to 16.5p Gbp in the mountain grasshopper *Podisma pedestris* [2]. Genome size variation relates poorly to the number of coding genes or the complexity of the organism (C-value enigma, [3],[4],[5],[6]) and evolutionary drivers of genome size variation remain a topic of ongoing debate (e.g. [7], [8], [9], [10]). Two principally different mechanisms are proposed as primary drivers of eukaryotic genome size evolution: Whole genome duplication (WGD, i.e., polyploidy) or smaller duplication events and expansion of repetitive elements (REs, [5]). While WGD is ubiquitous in plant evolution, it has been regarded as the exception in animals [11], [12]. However, ancient WGD has been hypothesized to be an important driver of evolution of mollusks (e.g. [13]) amphibians (e.g. [14], [15], fish (e.g. [16], [17], [18]) and arthropods (e.g. [19], [20], [21]), including multiple putative ancient large-scale gene duplications within Trichoptera [22].

RE expansion is an important driver of genome size variation in many eukaryotic genomes [23], [24]. The two major categories of REs are tandem repeats (e.g., satellite DNA) and mobile transposable elements (TEs). TEs are classified into class I [retrotransposons: endogenous retroviruses (ERVs), related long terminal repeat (LTR) and non-LTR retrotransposons: SINEs (Short Interspersed Nuclear Elements), LINEs (Long Interspersed Nuclear Elements)] and class II elements (DNA transposons, [25]). In insects, the known genomic proportion of TEs ranges from 1% in the antarctic midge *Belgica antarctica* [26] to 65% in the migratory locust *Locusta migratoria* [27]. Broad-scale analysis of TE abundance in insects suggests that some order-

specific signatures are present, however, major shifts in TE abundance are also common at shallow taxonomic levels [28], [29], including in Trichoptera [30]. The movement and proliferation of REs can have deleterious consequences on gene function and genome stability [31], [32], [33], [34], [35]. Moreover, repeat content and abundance can turn over rapidly even over short evolutionary time scales (reviewed in [36]). This rapid evolution has consequences for genome evolution and speciation, e.g., repeat divergence causes genetic incompatibilities between even closely related species [37]. However, TEs can also be sources of genomic innovation with selective advantages for the host [38], [39], [40], [41], [42], [43] and they can contribute to global changes in gene regulatory networks [44], [45], [46]. Investigating RE dynamics in diverse clades provides a powerful lens for understanding their roles in genome function and evolution. Broadly studying of RE dynamics in species-rich groups with wide variation in RE activity is an important step towards efficiently identifying study systems at finer taxonomical scales (natural populations, species complexes, or recently diverged species) that are ideally suited to advance our understanding of molecular and evolutionary mechanisms underlying genome evolution. In addition, by taking this biodiversity genomics approach, we can develop new model systems and eventually better understand links between environmental factors, genome size evolution, adaptation, and speciation (see [47]).

With more than 16,500 species, caddisflies (Trichoptera) are among the most diverse of all aquatic insects [48]. Their species richness is reflective of their ecological diversity, including, e.g. microhabitat specialization, a full array of feeding modes, and diverse use of underwater silk secretions [49], [50]. An initial comparison of six caddisfly species found wide genome size variation in Trichoptera (ranging from 230 Mbp to 1.4 Gbp). In that study, we hypothesized that the observed variation was correlated with caddisfly phylogeny and that TEs contributed to a suborder-specific increase of genome size [30].

Here, we present a multi-faceted analysis to investigate genome size evolution in the order Trichoptera, as an example for highly diversified non-model organisms. Specifically, we (i)

estimated genome size for species across the order to explore phylogenetic patterns in the distribution of genome size variation in Trichoptera and (ii) generated 17 new Trichoptera genomes to analyze, in conjunction with 9 existing genomes, the causes (WGD, TE expansions) of genome size variation in the evolution of caddisflies. Studying the genomic diversity of this highly diversified insect order adds new insights into drivers of genome size evolution with potential to shed light on how genome size is linked to form, function, and ecology.

Data Description

Genomic resources

Here, we combined long- and short-read sequencing technologies to generate 17 new *de novo* genome assemblies across a wide taxonomic range, covering all major lineages of Trichoptera. Details on sequencing coverage and assembly strategies are given in DataS1_Sup.2, DataS1_Sup.3, and supplementary note 3. To assess quality, we calculated assembly statistics with QUAST v5.0.2 [51], examined gene completeness with BUSCO v3.0.2 [52], [53] and screened for potential contamination with taxon-annotated GC-coverage (TAGC) plots using BlobTools v1.0 ([94], supplementary Figs. S31-S47). The new genomes are of comparable or better quality than other Trichoptera genomes previously reported in terms of BUSCO completeness and contiguity (Table 1). This study increases the number of assemblies in this order from nine to 26, nearly tripling the number of available caddisfly genomes and thus providing a valuable resource for studying genomic diversity across this ecologically diverse insect order. The annotation of these genomes predicted 6,413 to 12,927 proteins (Datas1_Sup.2). Most of the annotated proteins (94.4% - 98.8%) showed significant sequence similarity to entries in the NCBI nr database. GO Distributions were similar to previously annotated caddisfly genomes, i.e. the major biological processes were cellular and metabolic processes. Catalytic activity was the largest subcategory in molecular function and the cell membrane subcategories were the largest cellular component (supplementary Figs. S1-S30).

This project has been deposited at NCBI under BioProject ID: PRJNA558902. For accession numbers of individual assemblies see Table 1.

We downloaded existing Trichoptera genomes from GenBank (https://www.ncbi.nlm.nih.gov/genome/) or Lepbase (http://download.lepbase.org/v4/) and used these in conjunction with our newly generated genomes to analyze genome size evolution as explained in the following sections of this manuscript.

Flow cytometry

In addition to genomic sequence data, we used flow cytometry to detect genome size variation across the order. Our study increased the number of species with available flow cytometry-based genome size estimates from 4 [55] to 31. Estimates were submitted to the Animal Genome Size Database (http://www.genomesize.com).

Analysis

Genome size evolution in Trichoptera

Based on the genomes of six trichopteran species, Olsen et al. [30] found a 3-fold suborder-specific increase of genome size and hypothesized that genome size variation is correlated with their phylogeny. To test this hypothesis, we first reconstructed phylogenetic relationships by analyzing ~2,000 single-copy BUSCO genes from the 26 study species (Figs. 1 & 2, Fig. S48). We obtained a molecular phylogeny that was in agreement with recent phylogenetic hypotheses ([56], see supplementary note 6) and which showed that Trichoptera is divided into two suborders: Annulipalpia (Figs. 1 & 2: Clade A, blue) and Integripalpia [consisting of basal Integripalpia (Fig. 1: Clade B1-3, light green) and infraorder Phryganides (Fig. 1: clade B4, dark green)]. Trichopterans use silk to build diverse underwater structures (see illustrations Fig. 1; supplementary note 6, supplementary Fig. S48). Thus, we refer to Annulipalpia as 'fixed

retreat- and net-spinners', to Phryganides (Integripalpia) as 'tube case-builders', and to basal Integripalpia as 'cocoon-builders'.

We used three approaches for estimating genome size across Trichoptera: *k-mer* distribution-estimates, backmapping of sequence data to available draft genomes (as described in [57]), and flow cytometry (FCM, supplementary note 7, supplementary figures S49-S72, DataS1_Sup.7). FCM estimates can be affected by chromatin condensation, the proportion of cells in G0 to G1 phases [58], [59] and endoreplication in insect cells and tissues [60]. Sequence-based estimates can be affected by repetitive elements in the genome resulting in smaller genome size estimates (e.g. [61], [55], [62]), as well as by GC-content because sequence library preparation including PCR amplification steps are associated with underrepresentation of GC and AT rich regions [63]. Bland-Altman plots (supplementary note 8, Fig. S73) revealed general agreement of all three methods in our study. However, the FCM estimates were generally higher compared to the sequence-based estimates (Fig. 1, DataS1_Sup.7) and, among all three approaches, this measure is expected to be the most accurate [8]. We observe that variation among the methods increased with genome size, indicating issues potentially caused by repeat content (see Results *Repeat dynamics*).

We observed large variation in genome size across the order. Genome size tends to be lower in 'fixed retreat- and net-spinners' and 'cocoon-builders' compared to 'tube case-builders' (Fig. 1). Specifically, we observe that genome size varies ~14-fold ranging from 1C = 154 Mbp in 'cocoon-builders' (Fig. 1, B1: Hydroptilidae) to 1C = 2129 Mbp in 'tube case-builders' (Fig. 1, clade B4: Limnephilidae). Of the 29 species analyzed by FCM, *Halesus digitatus* (Fig. 1, clade B4: Limnephilidae, Intergripalpia) possessed the largest genome (1C = 2129 Mbp), while the genome of *Hydropsyche saxonica* (Fig. 1, clade A: Hydropsychidae, 'fixed retreat- and net-spinners') was the smallest (1C = 242 Mbp). Genome size estimates based on sequence-based methods (*kmer*-based and back-mapping) range from 1C = 154 - 160 Mb in *Agraylea*

sexmaculata (Fig. 1, clade B1: Hydroptilidae, 'cocoon-builders') to 1C = 1238 - 1400 Mbp in *Sericostoma* sp. (Fig. 1, clade B4: Sericostomatidae, 'tube case-builders').

Repeat Dynamics

Repetitive element abundance and classification

To understand the structural basis of genome size variation across the order Trichoptera we explored repetitive element (RE) content. We found that major expansions of transposable elements (TEs) contribute to larger genomes in 'tube case-' and some 'cocoon-builders', but particularly in 'tube case-builders' with an average of ~600 Mbp of REs compared to ~138 Mbp in 'fixed retreat- and net-spinners' (Fig. 2 A, B). LINEs are the most abundant classified TEs in 'cocoon-' and 'tube case-builders' and comprise >154 Mb on average in 'tube case-builders', or an average genome proportion of 16.9% (range = 5.6–34.7%). This represents a 1.8- and 2.8-fold increase in genome proportion relative to 'cocoon-builders' and 'fixed retreat- and net-spinners', respectively. The LINE abundance of >312 Mbp in *Odontocerum albicorne* exceeds the entire assembly lengths (152–282 Mbp) of the three smallest genome assemblies (*Hydropsyche tenuis*, *Parapsyche elsis*, and *Agraylea sexmaculata*) (Fig. 2 A, B). DNA transposons also comprise large genomic fractions in both 'cocoon-' and 'tube case-builders' (averages of 54.4 Mbp and 32.8 Mbp, respectively). However, despite containing a large number of bps, they make up a smaller fraction of total bps in the genomes of 'cocoon-' and 'tube case-builders' than in 'fixed retreat- and net-spinners' (average genome proportion = 5.9%, 4.5%, and 11.1% in 'tube case-builders', 'cocoon-builders', and 'fixed retreat- and net-spinners', respectively) (Fig. 2 B), and thus cannot, by themselves, explain the larger genome sizes. SINEs, LTRs, *Penelope* (grouped with "other" repeats in Fig. 2), and satDNAs show a disproportionate increase in 'cocoon-' and 'tube case-builders', however, all categories combined make up a relatively small proportion of their genomes (all less than 3% on average in Integripalpia) (Fig. 2, B). Unclassified repeats are the most abundant repeat category across

all Trichoptera, and they also show disproportionate expansions in both ‘cocoon-’ and ‘case-builders’ relative to ‘fixed retreat- and net-spinners’ (Fig. 2 A, B). The general trends noted in our assembly-based analysis of REs were corroborated by our reference-free analysis of repeat abundance (Figs. S122, S123 supplementary note 10).

TE age distribution analysis

To test whether the observed abundance patterns of specific TEs are driven by shared ancient proliferation events or more recent/ongoing activity of the respective TEs, we analyzed TE age distribution plots. These plots allow us to visualize specific RE classes/superfamilies that account for shifts in RE composition and abundance and infer the relative timing of those shifts based on the distribution of sequence divergence within each RE category. TE age distributions showed a high abundance of recently diverged TE sequences in ‘cocoon-’ and ‘tube case-builders’, particularly in LINEs, DNA transposons, and LTRs in which the majority of TEs for a given class show 0–10% sequence divergence within copies of a given repeat (Fig. 3). This trend was particularly pronounced among ‘tube case-builders’ with several species showing high abundance of LINEs and DNA transposons with 0–5% sequence divergence (Fig. 3). This pattern suggests that the observed TE expansion is due primarily to ongoing TE activity within lineages rather than a few shared bursts of activity in ancestral lineages. This is further supported by our analysis of repeat sub-classes with age distribution plots (Fig. S124). For example, in our study, LINE abundance is often due to the expansion of different LINE subclasses even between species in the same sub-clade (e.g., compare *Lepidostoma* with *Micrasema*, *Himalopsyche* with *Glossosoma*; Fig. S124). We also find evidence of shared ancient bursts of SINE activity in ‘cocoon-’ and ‘tube case-builders’, although SINEs are not an abundant repeat class in any species (avg. genomic proportion=1.9% stdev=1.7%) (Fig. S124).

Associations between TE sequences and protein-coding genes

During early exploration of our sequence data, we made an unexpected discovery that in some lineages, universal single copy orthologs or "BUSCO genes", showed higher than expected coverage depth of mapped reads in one or more of their sequence fragments. Further analysis showed that these high coverage BUSCO sequence regions are typically RE sequences (primarily TEs) that are either embedded within or located immediately adjacent to BUSCO genes, such that the BUSCO algorithm includes them in its annotation of a given gene. We refer to BUSCO genes containing these putative RE fragments as 'TE-associated BUSCOs' (supplementary Fig. S125, supplementary note 11). By estimating how many times they occur, we can quantitatively measure how TE-gene interactions change with changing genome size. In fact, we detected a positive linear relationship between TE-gene interactions and increasing genome size when measured with this accidently discovered metric. We found major expansions of TE-associated BUSCOs in 'cocoon-' and 'tube case-builders' (Fig. 4A) that are significantly correlated with total repeat abundance, as well as the genomic proportion of LINEs and DNA transposons (supplementary Fig. S126). TE-associated BUSCOs comprise a relatively large fraction of total BUSCO genes in these lineages (averages of 11.2% and 21.4% of total BUSCOs in 'cocoon-' and 'tube case-builders', respectively), compared to annulipalpian lineages (avg = 6.2%). This finding highlights the major impact of REs on the composition of protein-coding genes in species with repeat-rich genomes. The BUSCO-associated sequences may represent TEs recently inserted into BUSCO genes, the remnants left behind following historical TE transposition events, or TE sequences that are immediately adjacent to and inadvertently classified as BUSCO sequences.

To confirm that unexpectedly high-coverage sequence regions in TE-associated BUSCOs were in fact TE-derived sequences, we compared patterns of BUSCO gene structure (though pairwise alignment) across species pairs in which high-coverage regions (i.e., putative TE sequences) were present in the BUSCO gene of one species (i.e., the "inflated" species), but absent in the

homologous BUSCO of the other (i.e., the "reference" species). This analysis showed that in 73 of 75 randomly sampled alignments, reference species showed gaps or highly non-contiguous alignments in high-coverage regions of the inflated species (Fig. 4B), suggesting that sequence insertions are typically present in high-coverage sequence regions of TE-associated BUSCOs. Our subsequent BLAST analysis showed that comparing a TE-associated BUSCO against its own assembly produced thousands-millions of BLAST hits from many contigs (Fig. 4C). This confirmed that the indel sequence present in high-coverage regions of "inflated" species show high sequence similarity to repetitive elements elsewhere in the genome. We then used an intersect analysis on the BLAST results to confirm that the large majority of the excessive BLAST hits overlap with RE annotations throughout the genome, most of which are TEs with LINEs and DNA transposons being most abundant (Fig. 4D, DataS2_Sup.5). Finally, we found that if we replaced the TE-associated BLAST query sequence with the homologous, but non-TE associated BUSCO from its counterpart reference species, the number of BLAST hits was fewer (Fig. 4C, DataS2_Sup.6), offering further evidence that the TE sequence insertions driving the pattern of high-coverage in read mapping excessive BLAST hits are absent in reference species and thus carriable across relatively short time scales within Trichoptera. Taken together, these findings provide strong evidence that TE sequences (especially LINEs and DNA transposons) inadvertently annotated by BUSCO can account for the high-coverage regions we observe in BUSCO genes (Fig. 4D).

Our accidental discovery that quantifying the frequency of TE-associated BUSCOs can serve as an estimate of TE-gene associations may prove useful in other systems given the wide use of BUSCO analysis in genomic studies. Finer details supporting the TE-gene association analysis are reported in supplementary note 11.

Gene and genome duplications

Recently, a transcriptome-based study found evidence for putative ancient gene and genome duplications in hexapods, including potential WGD events in caddisflies [22], suggesting that duplication events could be responsible for some genome size variation in Trichoptera. We investigated whether this pattern persists with whole genome data and found that the age distribution of duplications in 18 genomes were significantly different compared to the background rate of gene duplication (Figs. S137 & S138). To identify if any significant peak is consistent with a potential WGD, we used mixture modeling to identify peaks in these gene age distributions, which recovered no obvious peak consistent with an ancient WGD. To further investigate potential WGD, we used Smudgeplot [64] to visualize the haplotype structure and to estimate ploidy of the genomes.

While Smudgeplot predicted most of the genomes to be diploid, four genomes with rather small genome sizes (230 Mb – 650 Mbp) were predicted to be tetraploid (*Hydropsyche tenuis*, *Rhyacophila evoluta* RSS1 and HR1, *Parapsyche elsis*). However, the Genomescope 2 results indicate that these are highly homozygous samples. Low heterozygosity is a known confounder of smudgeplot analyses (see https://github.com/KamilSJaron/smudgeplot/wiki/tutorial-strawberry) because it inflates the signal of duplication when compared to the low level of heterozygosity. We therefore interpret these four putative polyploids as artifacts of low heterozygosity in the analysis.

Discussion

The drivers and evolutionary consequences of genome size evolution are a topic of ongoing debate. Several models have been proposed [8]. Some hypothesize genome size to be a (mal)adaptive trait by impacting phenotypic traits such as developmental/life history, body size and other cell-size related effects [65], [66], [67], [68] reviewed in [8]. On the other hand, neutral theories suggest that DNA accumulation occurs only by genetic drift without selective

pressures playing a major role in the accumulation or loss of DNA [the mutational hazard hypothesis (MHH, [23]) and the mutational equilibrium hypothesis (MEH, [24])]. The MHH only allows for small deleterious effects for the accumulation of extra DNA which is accompanied by higher mutation rates in larger genomes [23], while the MEH focuses on the balance between insertions and deletions. It suggests that genome expansions arise by 'bursts' of duplication events or TE activity and genome shrinkage may be caused by a more constant rate of small deletions [24].

In this study, we observe that genome size varies ~14-fold across the order Trichoptera, with lower genome size estimates in 'fixed retreat- and net-spinners' and 'cocoon-builders' compared to 'tube case-builders' and explore potential drivers of genome size evolution. Although, recent genomic studies have shown evidence of bursts of gene duplication and gene family expansion during the evolution of hexapods [22], [69] the presence of ancient genome duplication events are still a subject of debate [70], [71], [72]. We found neither evidence for whole duplication events when computing haplotype structure and ploidy with Smudgeplot, nor evidence of ancient WGD in the gene age distribution in our Trichoptera genomes although we recognize that some of our current genome assemblies might be too fragmented to infer synteny. This does not mean that we can rule out that duplication events played a role in genome size evolution in Trichoptera in the past. The emergence of PacBio HiFi genomes of caddisflies (e.g., Darwin Tree of Life is currently planning to sequence 28 caddisfly genomes; https://www.darwintreeoflife.org/) will allow a deeper exploration of putative ancient duplication events in Trichoptera.

We found evidence that TE expansions (especially LINEs) were important drivers of genome size evolution in Trichoptera (Fig. 2, Figs. S122 & S123), which is consistent with the mutational equilibrium hypothesis (MEH). The TE age distribution analyses suggested that the high abundance of LINEs was due to ongoing/recent activity occurring independently across 'cocoon-' and particularly 'tube case-builders' (Fig. 3, Fig. S124). Thus, the shift to large

genomes in these lineages does not appear to be due to a single (or few) shared ancient events, rather they maintained dynamic turnover in composition of their large genomes. Mutational bias affecting pathways tied to TE-regulation may affect insertion/deletion ratios and subsequently lead to lineage-specific shifts in genome size equilibrium [73]. Such changes may be stochastic (e.g., due to drift), or linked to traits that evolve on independent trajectories as lineages diverge and are thereby constrained by phylogeny. Ecological factors, demographic history, and effective population size can further impact mutation rates. For example, environmental stress can trigger bursts of TE activity and elevated mutation rates [74], [75], [76] driving lineages that occupy niche space with frequent exposure to environmental stress toward increased TE loads and larger genomes. Similarly, lineages with small effective population sizes or which are prone to population bottlenecks may have higher mutation rates and/or reduced efficacy of natural selection which would otherwise purge mildly deleterious TE load.

Although our study is not designed to pinpoint specific forces maintaining large genomes in some lineages, the pattern we observe in the distribution of genome size (i.e. lower genome size estimates in 'fixed retreat- and net-spinners' and 'cocoon-builders' compared to 'tube case-builders') leads us to hypothesize that ecological factors may play a role in genome size evolution in the order. The three focal groups discussed here exhibit markedly different ecological strategies. Larvae of 'fixed retreat- and net-spinners' generally occupy relatively narrow niche space in oxygen-rich flowing-water (mostly stream/river) environments where they rely on water currents to bring food materials to their filter nets. The evolutionary innovation of tube-case making is thought to have enabled 'tube case-builders' to occupy a much greater diversity of ecological niche space by allowing them to obtain oxygen in lentic (e.g., pond, lake, marsh) environments which are much more variable in temperature and oxygen availability than lotic environments [77], [78]. This environmental instability is greater over short (daily, seasonal) and long-time scales (centuries, millennia) [79]. It is thus plausible

these tube case-building lineages experience greater environmental stress and less stable population demographics that could lead to both more frequent TE bursts and reduced efficacy of natural selection in purging deleterious effects of TE expansions as described above [23], [24].

We show that TE expansions (especially LINEs and DNA transposons) in 'cocoon-' and 'tube case-builders' have a major impact on protein-coding gene regions (Fig. 4). These TE-gene associations show a linear relationship with increasing genome size. This trend is particularly pronounced among 'tube case-builders' in which TE-associated BUSCOs comprise an average of 21.4% of total BUSCO genes (compared to 6.2% in annulipalpians). This finding corroborates other studies highlighting the role of TEs as drivers of rapid genome evolution [80], [81], [82], [83] and highlights their impact on genomic regions that have potential effects on phenotypes. Questions remain as to what evolutionary roles such changes in genic regions may play. In general, TE insertions are considered to have deleterious effects on their host's fitness activity [84], [85]. They are known to "interrupt" genes [33], pose a risk of ectopic recombination that can lead to genome rearrangements [34], [31], [86], and have epigenetic effects on neighboring sequences [87], [88]. Therefore, purifying selection keeps TEs at low frequencies [33]. However, there is growing evidence that TE activity can also be a critical source of new genetic variation driving diversification via chromosomal rearrangements and transposition events which can result in mutations [89], including examples, of co-option [90], e.g. recent research in mammals has shown that DNA transposon fragments can be co-opted to form regulatory networks with genome-wide effects on gene expression [44].

Ecological correlates with genome size are widely discussed in other taxa [91], [92], [93], [94], [95]. Caddisflies and other diverse insect lineages that feature various microhabitat specializations, feeding modes, and/or the use of silk represent evolutionary replicates with contrasting traits and dynamic genome size evolution. They thus have high potential as models for understanding links between ecology and the evolution of REs, genomes, and phenotypes.

Our study lays a foundation for future work in caddisflies that investigates the potential impact of TE expansions on phenotypes and tests for evidence of co-option/adaptive impacts of TE-rich genomes against a null of neutral or slightly deleterious effects.

Potential implications

Many open questions remain as to the causes and consequences of genome size evolution. As we move forward in an era where genome assemblies are attainable for historically intractable organisms (e.g. due to constraints given large genome sizes, tissue limitations, no close reference available) we can leverage new model systems spanning a greater diversity of life to understand how genomes evolve. Here, we provide genomic resources and new genome size estimates across lineages of an underrepresented insect order that spans major variation in genome size. These data allowed us to study genome size evolution in a phylogenetic framework to reveal lineage-specific patterns in which genome size correlates strongly with phylogeny and ecological characteristics within lineages. We find that large genomes dominate lineages with a wider range of ecological variation, and that ongoing recent TE activity appears to maintain large genomes in these lineages. This leads us to hypothesize that ecological factors may be linked to genome size evolution in this group. The future directions spawned by our findings highlight the potential for using Trichoptera and other diverse insect groups to understand the link between ecological and genomic diversity, a link that has been challenging to study with past models [8].

We also show that TE expansions are associated with increasing genome size and have an impact on protein-coding regions. These impacts have been greatest in the most species-rich and ecologically diverse caddisfly clades. While TEs are generally considered to have deleterious effects on their host's fitness activity, their roles can also be neutral or even adaptive. TE activity can be a critical source of new genetic variation and thus an important driver for diversification. Caddisflies and potentially other non-model insect groups are excellent models

to test these contrasting hypotheses, as well as the potential impact of TEs on phenotypes. Using these models, especially with respect to the increasing emergence of high-quality insect genomes [96], will allow researchers to identify recurring patterns in TE dynamics and investigate their evolutionary implications across diverse clades.

Methods

DNA extraction, library preparation, sequencing, and sequence read processing

We extracted high molecular weight genomic DNA (gDNA) from 17 individuals (15 species) of caddisfly larvae (for sampling information, see DataS1_Sup.1) after removing the intestinal tracts using a salting-out protocol adapted from [97] as described in supplementary note 1. We generated gDNA libraries for a low-cost high-contiguity sequencing strategy, i.e. employing a combination of short (Illumina) and long read (Nanopore or PacBio) technologies as described in supplementary notes 2. For details on sequencing coverage for each specimen see DataS1_Sup.3.

De novo genome assembly, annotation and quality assessment

We applied different assembly strategies for different datasets. First, we applied a long-read assembly method using wtdbg2 v2.4 [98] with subsequent short-read polishing with Pilon v1.22 [99] as this method revealed good results in previous *de novo* assemblies in caddisflies [55]. In cases where this pipeline did not meet the expected quality regarding contiguity and BUSCO completeness, we applied *de novo* hybrid assembly approaches of MaSuRCA v.3.1.1 [100] (supplementary note 3). Illumina-only data was assembled with SPAdes [101] explained in supplementary note 3. Prior to annotating the individual genomes with MAKER2 v2.31.10 [102], [103] we used RepeatModeler v2.0 and RepeatMasker v4.1.0, to identify species-specific repetitive elements in each of the assemblies, relative to RepBase libraries v20181026;

www.girinst.org). Transcriptome evidence for the annotation of the individual genomes included their species-specific or closely related *de novo* transcriptome provided by 1KITE (http://www.1kite.org/; last accessed November 11, 2019, DataS1_Sup.9) or downloaded from Genbank as well as the cDNA and protein models from *Stenopsyche tienmushanensis* [104] and *Bombyx mori* (AR102, GenBank accession ID# GCF_000151625.1). Additional protein evidence included the uniprot-sprot database (downloaded September 25, 2018). We masked repeats based on species-specific files produced by RepeatModeler. For *ab initio* gene prediction, species specific AUGUSTUS gene prediction models as well as *Bombyx mori* SNAP gene models were provided to MAKER. The EvidenceModeler [105] and tRNAscan [106] options in MAKER were employed to produce a weighted consensus gene structure and to identify tRNAs genes. MAKER default options were utilized for BLASTN, BLASTX, TBLASTX searches. Two assemblies (*Agapetus fuscipens* GL3 and *Micrasema longulum* ML1) were not annotated because of their low contiguity. All protein sequences were assigned putative names by BlastP Protein–Protein BLAST 2.2.30+ searches [107] and were functionally annotated using command line Blast2Go v1.3.3 [108], see supplementary note 4, Figs. S1-S30). We calculated assembly statistics with QUAST v5.0.2 [51] and examined completeness with BUSCO v3.0.2 [52], [53] using the Endopterygota odb9 dataset with the options *--long*, *–m = genome* and *–sp= fly*. A summary of the assembly statistics and BUSCO completeness is given in Table 1. The final genome assemblies and annotations were screened and filtered for potential contaminations with taxon-annotated GC-coverage (TAGC) plots using BlobTools v1.0 [54]. Details and blobplots are given in supplementary note 5 & supplementary Figs. S31-S47.

Species tree reconstruction

We used the single-copy orthologs resulting from the BUSCO analyses to generate a species tree. We first combined single-copy ortholog amino acid files from each species into a single

FASTA for each ortholog. We then aligned them with the MAFFT L-INS-i algorithm [109]. We selected amino acid substitution models for each ortholog using ModelFinder (option -m mfp,[110] in IQtree v.2.0.6 [111] and estimated a maximum likelihood tree with 1000 ultrafast bootstrap replicates [112] with the BNNI correction (option -bb 1000 -bnni). We combined the best maximum likelihood tree from each gene for species tree analysis in ASTRAL-III [113]. A locus tree was inferred using the alignment file (-s) and the partition file (-S) with the settings –prefix loci and -T AUTO in IQtree. Gene and site concordance factors were calculated with IQTree using the species tree (-t), the locus tree (--gcf) and the alignment file (-s) with 100 quartets for computing the site concordance factors (--scf 100) and --prefix concord for computing the gene concordance factors. We visualized the trees using FigTree v.1.4.4 (http://tree.bio.ed.ac.uk/software/figtree/).

Genome size estimations and genome profiling

Genome size estimates of 27 species were conducted using flow cytometry (FCM) according to Otto [114] using *Lycopersicon esculentum* cv. Stupické polnítyčkové rané (2C = 1.96 pg;[115]) as internal standard and propidium iodine as stain. Additionally, we used trimmed, contamination filtered short-read data (see supplementary note 2) to conduct genome profiling (estimation of major genome characteristics such as size, heterozygosity, and repetitiveness) using a *k-mer* distribution-based method (GenomeScope 2.0, [64]. Genome scope profiles are available online (see links to Genomescope 2 in DataS1_Sup.4). In addition, we applied a second sequencing-based method for genome size estimates, which uses the backmapping rate of sequenced reads to the assembly and coverage distribution (backmap.pl v0.1, [57]. Details of all three methods are described in supplementary note 7**.** Coverage distribution per position and genome size estimate from backmap.pl are shown in Figs. S49-72). We assessed the congruence among the three quantitative methods of measurement (Genomescope2, Backmap.pl and FCM) with Bland-Altman-Plots using the function

493 BlandAltmanLeh::bland.altman.plot in ggplot2 [116] in RStudio (RStudio Team (2020).
494 RStudio: Integrated Development for R. RStudio, PBC, Boston, MA URL
495 http://www.rstudio.com/; supplementary note 8, Fig. S73).
496
497 **Repeat dynamics**
498 *Repeat abundance and classification*
499 We identified and classified repetitive elements in the genome assemblies of each species using
500 RepeatModeler2.0 [117]. We annotated repeats in the contamination filtered assemblies with
501 RepeatMasker 4.1.0 (http://www.repeatmasker.org) using the custom repeat libraries generated
502 from RepeatModeler2 for each respective assembly with the search engine set to "ncbi" and
503 using the -xsmall option. We converted the softmasked assembly resulting from the first
504 RepeatMasker round into a hardmasked assembly using the lc2n.py script
505 (https://github.com/PdomGenomeProject/repeat-masking). Finally, we re-ran RepeatMasker
506 on the hard-masked genome with RepeatMasker's internal arthropod repeat library using -
507 species "Arthropoda". We then merged RepeatMasker output tables from both runs by parsing
508 them with a script (RM_table_parser_families_.py, available at
509 https://github.com/jhcaddisfly/TE-gene_intersect_analysis) and then combined the resulting
510 data columns for the two runs in Excel.
511 We also estimated repetitive element abundance and composition using RepeatExplorer2[118],
512 [119] and dnaPipeTE v.1.3.1 [120]. These reference-free approaches quantifies repeats directly
513 from unassembled short-read data. These analyses allowed us to test for general consistency of
514 patterns with our assembly-based approach described above, and to test for the presence of
515 abundant repeat categories such as satellite DNAs which can comprise large fractions of
516 genomes yet can be prone to poor representation in the genome assembly. Prior to analysis, we
517 normalized contamination filtered (see supplementary note 2) input data sets to 0.5x coverage
518 using RepeatProfiler [121] and seqtk (https://github.com/lh3/seqtk), and then ran

RepeatExplorer2 clustering with the Metazoa 3.0 database specified for annotation (supplementary Fig. S122) and dnaPipeTE with the -RM_lib flag set to the Repbase v20170127 repeat library (supplementary Fig. S123).

TE age distribution analysis

We further characterized repetitive element dynamics in Trichoptera by analyzing TE landscapes, which show relative age differences among TE sequences and their genomic abundance. We used these analyses to test whether abundance patterns of specific TEs are driven by shared ancient proliferation events or more recent/ongoing activity of the respective TEs. For example, if shared ancient proliferation is driving abundance patterns of a given TE, the majority of its copies would show moderate to high sequence divergence (e.g., >10% pairwise divergence). In contrast, if abundance patterns are driven by recent/ongoing activity of a given TE, we would expect the majority of its sequences to show low sequence divergence (e.g., 0–10%). We generated TE age distribution plots using dnaPipeTE v1.3.1 [120] with genomic coverage for each species sampled to 0.5X prior to analysis and the -RM_lib flag set to the Repbase v20170127 repeat library (supplementary Fig. S124).

TE sequence associations with protein-coding genes

We analyzed BUSCO genes for all species to quantify the abundance of TE-associated BUSCOs across samples and investigated associations between TEs and genic sequences in Trichoptera lineages by quantifying the abundance of TE-associated BUSCO genes (for presence and absence of TE-associated BUSCOs see Fig. S125, DataS2_Sup.3). This analysis also allowed us to quantify shifts in associations between TEs and genic regions across Trichoptera lineages with varying repeat abundance. We identified BUSCO genes with high-coverage sequence regions based on coverage profiles and quantified their genomic abundance by using each TE-associated BUSCO as a query in a BLAST search against their respective

genome assembly. We then conducted intersect analysis for all unique BUSCO hits from high coverage sequences to determine if these were annotated as TEs. We calculated the total number of bases in filtered BLAST after subtracting the number of bases at the locus belonging to all 'complete' BUSCO genes and categorized high coverage sequence regions in BUSCO genes based on their annotation status and repeat classification using custom scripts (available at https:// https://github.com/jhcaddisfly/TE-gene_intersect_analysis). We plotted the number of the high coverage BUSCO sequence regions belonging to repetitive element categories (i.e., classes and subclasses) alongside plots of the relative genomic abundance of each respective category. In addition, we investigated BUSCO genes with regions of high coverage by pairwise alignments. Specifically, we visualized alignments of BUSCOs with high coverage sequence regions (i.e., the "inflated species") alongside orthologous BUSCOS that lack such regions taken from closely related species (i.e., the "reference" species). We further tested this prediction by taking the set of BUSCOs that only exhibited high coverage regions in the inflated species and contrasted results of two BLAST searches followed by an intersect analysis. A detailed description of this method is provided in supplementary note 11.

Gene and Genome duplications

Inference of WGDs from gene age distributions

To recover signal from potential WGDs, for each genome, we used the DupPipe pipeline to construct gene families and estimate the age distribution of gene duplications [122], https://bitbucket.org/barkerlab/evopipes/src/master/). We translated DNA sequences and identified ORFs by comparing the Genewise [123] alignment to the best- hit protein from a collection of proteins from 24 metazoan genomes from Metazome v3.0. For all DupPipe runs, we used protein- guided DNA alignments to align our nucleic acid sequences while maintaining the ORFs. We estimated synonymous divergence (K_s) using PAML with the F3X4 model [124] for each node in the gene family phylogenies. We first identified taxa with potential

WGDs by comparing their paralog ages to a simulated null distribution without ancient WGDs using a K-S goodness-of-fit test [125]. We then used mixture modeling to identify if any significant peaks consistent with a potential WGD and to estimate their median paralog K_s values. Significant peaks were identified using a likelihood ratio test in the boot.comp function of the package mixtools in R [126].

Visualization of genome structure to estimate ploidy using smudgeplots

We visualized the genome structure and estimated ploidy levels with smudgeplot. For this purpose, we extracted genomic kmers from kmer counts produced with jellyfish (as described above in "Genome size estimation and genome profiling") using "jellyfish dump" with coverage thresholds previously estimated from kmer histograms using the smudgeplot.py script. We computed the set of kmer pairs with the Smudgeplot tool hetkmers. After generating the list of kmer pair coverages, we generated smudgeplots using the coverage of the kmer pairs and the "plot" tool within Smudgeplot. Ploidy as well as the haploid kmer coverage was estimated directly from the data and compared to the estimates reported by Genomescope2 (see DataS1-Sup.4). Details of the method and smudgeplots are given in supplementary figures S74-121.

Figures Legends:

Fig. 1: Ecological diversity (right) and genome size (left) in caddisflies. Phylogenetic relationships derived from ASTRAL-III analyses using single BUSCO genes. Goeridae, which was not included in the BUSCO gene set, was placed according to [56]. ASTRAL support values (local posterior probabilities) higher than 0.9 are given for each node. The placement of Hydroptilidae (clade B1) was ambiguous. Since its placement was poorly supported in our analyses, we placed it according to Thomas et al. [56]. Taxa were collapsed to family level.

Trichoptera are divided into two suborders: Annulipalpia ('fixed retreat- and net-spinners', clade A: blue) and Intergripalpia (clade B: green) which includes basal Integripalpia ('cocoon-builders', clades B1-B3, dark green) and Phryganides or 'tube case-builders' (clade B4: light green). 'Cocoon-builders' are divided into 'purse case'- (clade B1), 'tortoise case-building' (clade B2) and 'free-living' (clade B3) families. Genome size estimates based on different methods (Genomescope2: orange, Backmap.pl: black, Flow Cytometry (FCM): brown) are given for various caddisfly families. Each dot corresponds to a mean estimate of a species. For detailed information on the species and number of individuals used in each method see Data S1_Sup.7 -Genome size - Summary. Colors and clade numbers in the phylogenetic tree refer to colored boxes with illustrations. The following species are illustrated by Ralph Holzenthal: a: *Hydropsyche sp.* (Hydropsychidae); b: *Chimarra sp.* (Philopotamidae); c: *Stenopsyche sp.* (Stenopsychidae); d: *Polycentropus* sp. (Polycentropodidae); e: *Agraylea sp.* (Hydroptilidae); f: *Glossosoma sp.* (Glossosomatidae); g: *Rhyacophila sp.* (Rhyacophilidae); h: *Fabria inornata* (Phryganeidae); i: *Micrasema* sp. (Brachycentridae); j:*Goera fuscula* (Goeridae); k: *Sphagnophylax meiops* (Limnephilidae); l: *Psilotreta sp.* (Odontoceridae), m: *Grumicha grumicha* (Sericostomatidae).

Fig. 2: Repeat abundance and classification in 26 caddisfly genomes. Number of bp for each repeat type is given for each caddisfly genome. A: Repeat abundance and classification. Phylogenetic tree was reconstructed with ASTRAL-III using single BUSCO genes from the genome assemblies. The placement of Hydroptilidae (clade B1) was ambiguous. Since its placement was poorly supported in our analyses, we placed the single hydroptilid taxon (*Agraylea sexmaculata*) according to Thomas et al. [56]. Species names corresponding to the abbreviations in the tree can be found in Table 1. Trichoptera are divided into two suborders: Annulipalpia ('fixed retreat- and net-spinners', clade A: blue) and Intergripalpia (clade B: green) which includes basal Integripalpia ('cocoon-builders', clades B1-B3, dark green) and

Phryganides or ‘tube case-builders’ (clade B4: light green). ‘Cocoon-builders’ are divided into ‘purse case’- (clade B1), ‘tortoise case-building’ (clade B2) and ‘free-living’ (clade B3) families. An illustration of a representative of each clade is given. The “other_repeats” category includes: rolling-circles, *Penelope*, low-complexity, simple repeats, and small RNAs. B: Boxplots summarizing shifts in the genomic proportion of RE categories in major Trichoptera lineages.

Fig. 3: Transposable element age distribution landscapes. Representative examples are chosen from major Trichoptera lineages. The y-axis shows TE abundance as a proportion of the genome (e.g., 1.0 = 1% of the genome). The x-axis shows sequence divergence relative to TE consensus sequences for major TE classes. TE classes with abundance skewed toward the left (i.e., low sequence divergence) are inferred to have a recent history of diversification relative to TE classes with right-skewed abundance. Plots were generated in dnaPipeTE. Plots for all species are shown in supplementary Fig. S123. For tip labels of the phylogenetic tree see Fig. 2.

Fig. 4: TE-BUSCO-gene associations in Trichoptera species. (A) Raw abundance of TE-associated BUSCO sequences present in the assembly of 2442BUSCOs in the OrthoDB 9 Endopterygota dataset. (B) Upper plot: An example of a coverage depth profile of a TE-associated BUSCO gene [BUSCO EOG090R02Q9 from ML1 (‘inflated species’)] which shows unexpected high coverage in the second exon putatively due to the presence of a RE-derived sequence fragment. Lower plot: A typifying alignment between a TE-associated BUSCO and its orthologous BUSCO from a closely related species (‘reference species’) that lack TE-association. The non-TE-associated orthologous BUSCO shows non-contiguous alignment in regions of inflated coverage in the TE-associated BUSCO, consistent with the presence of a RE-derived sequence fragment in the TE-associated BUSCO that is absent in the

reference species. (C) Summary of total bases annotated as REs obtained from each of two BLAST searches. First, when we used BLAST to compare an TE-associated BUSCOs against an assembly for the same species BLAST hits included megabases of annotated repeats (dark plots). Second, when non-TE-associated orthologs of the TE-associated BUSCOs in the first search are taken from a close relative and compared against the inflated species using BLAST, there is a dramatic drop in BLAST hits annotated as REs. Note log scale on the y-axis. (D) Summary of annotations for BLAST hits for classified REs when TE-associated BUSCOs are compared against an assembly of the same species using BLAST,

Data and materials availability:

This project has been deposited at NCBI under BioProject ID: PRJNA558902.

The data sets supporting the results of this article are available in the supplementary, data files S1 and S2 and at https://byu.box.com/v/trich-genomes. The data available at the link will be uploaded to GigaDB when the paper is accepted.

Declarations

Consent for publication: Not applicable

Competing interests: Authors declare that they have no competing interests.

Funding

This work is a result of the LOEWE-Centre for Translational Biodiversity Genomics funded by the Hessen State Ministry of Higher Education, Research and the Arts (HMWK) that supported JH and SUP, as well as internal funds of Senckenberg Research Institute provided to JP. JSS was supported by an NSF Postdoctoral Research Fellowship in Biology (DBI-1811930) and an NIH General Medical Sciences award (R35GM119515) to AML. Sequencing was, in part,

supported by BYU start-up funds to PBF and funds from the Army Research Office, Life Science Division (Award no. W911NF-13-1-0319) to RJS.

Author's contributions:

Conceptualization –JH, JSS, PBF, SUP

Data curation – JH

Formal Analysis - JH, JM, JP, JSS, PBF, ZL

Funding acquisition – AML, PBF, SUP, RJS, RJS

Investigation – JH, JP, JSS, PBF, ZL

Methodology – AML, JSS, JP, JVS, PBF

Project administration – SUP

Resources – JP, MB, PBF, SUP

Visualization - JH, JSS

Writing – original draft – JH, JSS, PBF, ZL

Writing – review & editing – AML, JH, JM, JP, JSS, JVS, MB, PBF, RJS, RJS, SUP, ZL

Acknowledgments

The authors thank Ralph Holzenthal for providing illustrations of larval Trichoptera and the structures they build. We thank Bob Wisseman for collecting *Himalopsyche phryganeae.* We thank both reviewers for their insightful critiques and interest in our manuscript.

References

1. Cornette R, Gusev O, Nakahara Y, Shimura S, Kikawada T, Okuda T. Chironomid midges (Diptera, chironomidae) show extremely small genome sizes. *Zoolog Sci*. 2015; doi: 10.2108/zs140166.

2. Westerman M, Barton NH, Hewitt GM. Differences in DNA content between two chromosomal races of the grasshopper Podisma pedestris. *Heredity*. Nature Publishing Group; 1987; doi: 10.1038/hdy.1987.36.

3. Thomas CA. The genetic organization of chromosomes. *Annu Rev Genet*. Annual Reviews; 1971; doi: 10.1146/annurev.ge.05.120171.001321.

4. Bernard J. The Eukaryote Genome in Development and Evolution. Springer Netherlands;

5. GREGORY TR. The C-value Enigma in Plants and Animals: A Review of Parallels and an Appeal for Partnership. *Ann Bot*. 2005; doi: 10.1093/aob/mci009.

6. Elliott TA, Gregory TR. What's in a genome? The C-value enigma and the evolution of eukaryotic genome content. *Philos Trans R Soc B Biol Sci*. Royal Society; 2015; doi: 10.1098/rstb.2014.0331.

7. Abdel-Haleem H. The Origins of Genome Architecture. *J Hered*. 2007; doi: 10.1093/jhered/esm073.

8. Blommaert J. Genome size evolution: towards new model systems for old questions. *Proc R Soc B Biol Sci*. Royal Society; 2020; doi: 10.1098/rspb.2020.1441.

9. Canapa A, Barucca M, Biscotti MA, Forconi M, Olmo E. Transposons, Genome Size, and Evolutionary Insights in Animals. *Cytogenet Genome Res*. 2015; doi: 10.1159/000444429.

10. Petrov DA. Evolution of genome size: new approaches to an old problem. *Trends Genet TIG*. 2001; doi: 10.1016/s0168-9525(00)02157-0.

11. Orr HA. "Why Polyploidy is Rarer in Animals Than in Plants" Revisited. *Am Nat*. The University of Chicago Press; 1990; doi: 10.1086/285130.

12. Otto SP, Whitton J. Polyploid Incidence and Evolution. *Annu Rev Genet*. 2000; doi: 10.1146/annurev.genet.34.1.401.

13. Hallinan NM, Lindberg DR. Comparative analysis of chromosome counts infers three paleopolyploidies in the mollusca. *Genome Biol Evol*. 2011; doi: 10.1093/gbe/evr087.

14. Alexandrou MA, Swartz BA, Matzke NJ, Oakley TH. Genome duplication and multiple evolutionary origins of complex migratory behavior in Salmonidae. *Mol Phylogenet Evol*. 2013; doi: 10.1016/j.ympev.2013.07.026.

15. Campbell MA, Hale MC, McKinney GJ, Nichols KM, Pearse DE. Long-Term Conservation of Ohnologs Through Partial Tetrasomy Following Whole-Genome Duplication in Salmonidae. *G3 GenesGenomesGenetics*. 2019; doi: 10.1534/g3.119.400070.

16. Woods IG, Wilson C, Friedlander B, Chang P, Reyes DK, Nix R, et al.. The zebrafish gene map defines ancestral vertebrate chromosomes. *Genome Res*. 2005; doi: 10.1101/gr.4134305.

17. Berthelot C, Brunet F, Chalopin D, Juanchich A, Bernard M, Noël B, et al.. The rainbow trout genome provides novel insights into evolution after whole-genome duplication in vertebrates. *Nat Commun*. Nature Publishing Group; 2014; doi: 10.1038/ncomms4657.

18. Glasauer SMK, Neuhauss SCF. Whole-genome duplication in teleost fishes and its evolutionary consequences. *Mol Genet Genomics MGG*. 2014; doi: 10.1007/s00438-014-0889-2.

19. Clarke TH, Garb JE, Hayashi CY, Arensburger P, Ayoub NA. Spider Transcriptomes Identify Ancient Large-Scale Gene Duplication Event Potentially Important in Silk Gland Evolution. *Genome Biol Evol*. 2015; doi: 10.1093/gbe/evv110.

20. Kenny NJ, Chan KW, Nong W, Qu Z, Maeso I, Yip HY, et al.. Ancestral whole-genome duplication in the marine chelicerate horseshoe crabs. *Heredity*. Nature Publishing Group; 2016; doi: 10.1038/hdy.2015.89.

21. Schwager EE, Sharma PP, Clarke T, Leite DJ, Wierschin T, Pechmann M, et al.. The house spider genome reveals an ancient whole-genome duplication during arachnid evolution. *BMC Biol*. 2017; doi: 10.1186/s12915-017-0399-x.

22. Li Z, Tiley GP, Galuska SR, Reardon CR, Kidder TI, Rundell RJ, et al.. Multiple large-scale gene and genome duplications during the evolution of hexapods. *Proc Natl Acad Sci*. National Academy of Sciences; 2018; doi: 10.1073/pnas.1710791115.

23. Lynch M, Conery JS. The Origins of Genome Complexity. *Science*. American Association for the Advancement of Science; 2003; doi: 10.1126/science.1089370.

24. Petrov DA. Mutational Equilibrium Model of Genome Size Evolution. *Theor Popul Biol*. 2002; doi: 10.1006/tpbi.2002.1605.

25. Wicker T, Sabot F, Hua-Van A, Bennetzen JL, Capy P, Chalhoub B, et al.. A unified classification system for eukaryotic transposable elements. *Nat Rev Genet*. Nature Publishing Group; 2007; doi: 10.1038/nrg2165.

26. Kelley JL, Peyton JT, Fiston-Lavier A-S, Teets NM, Yee M-C, Johnston JS, et al.. Compact genome of the Antarctic midge is likely an adaptation to an extreme environment. *Nat Commun*. Nature Publishing Group; 2014; doi: 10.1038/ncomms5611.

27. Wang X, Fang X, Yang P, Jiang X, Jiang F, Zhao D, et al.. The locust genome provides insight into swarm formation and long-distance flight. *Nat Commun*. Nature Publishing Group; 2014; doi: 10.1038/ncomms3957.

28. Petersen M, Armisén D, Gibbs RA, Hering L, Khila A, Mayer G, et al.. Diversity and evolution of the transposable element repertoire in arthropods with particular reference to insects. *BMC Ecol Evol*. 2019; doi: 10.1186/s12862-018-1324-9.

29. Gilbert C, Peccoud J, Cordaux R. Transposable Elements and the Evolution of Insects. *Annu Rev Entomol*. Annual Reviews; 2021; doi: 10.1146/annurev-ento-070720-074650.

30. Olsen LK, Heckenhauer J, Sproul JS, Dikow RB, Gonzalesz VL, Kweskin MP, et al.. De novo whole genome assemblies of Agrypnia vestita Walker, and Hesperophlax magnus Banks reveal substantial repetitive element expansion in tube case-making caddisflies (Insecta: Trichoptera). *Genome Biol Evol*. 2021; doi: 10.1093/gbe/evab013.

31. Montgomery E, Charlesworth B, Langley CH. A test for the role of natural selection in the stabilization of transposable element copy number in a population of Drosophila melanogaster. *Genet Res*. Cambridge University Press; 1987; doi: 10.1017/S0016672300026707.

32. Charlesworth B, Langley CH. Transposition of copia elements in Drosophila. *Nature*. Nature Publishing Group; 1988; doi: 10.1038/332021b0.

33. Charlesworth B, Langley CH. The Population Genetics of Drosophila Transposable Elements. *Annu Rev Genet*. 1989; doi: 10.1146/annurev.ge.23.120189.001343.

776 34. Montgomery EA, Huang SM, Langley CH, Judd BH. Chromosome rearrangement by ectopic
777 recombination in Drosophila melanogaster: genome structure and evolution. *Genetics*. 1991; doi:
778 10.1093/genetics/129.4.1085.

779 35. Mieczkowski PA, Lemoine FJ, Petes TD. Recombination between retrotransposons as a source of
780 chromosome rearrangements in the yeast Saccharomyces cerevisiae. *DNA Repair*. 2006; doi:
781 10.1016/j.dnarep.2006.05.027.

782 36. Ugarković Đ, Plohl M. Variation in satellite DNA profiles—causes and effects. *EMBO J*. John Wiley
783 & Sons, Ltd; 2002; doi: 10.1093/emboj/cdf612.

784 37. Ferree PM, Barbash DA. Species-Specific Heterochromatin Prevents Mitotic Chromosome
785 Segregation to Cause Hybrid Lethality in Drosophila. *PLOS Biol*. Public Library of Science; 2009; doi:
786 10.1371/journal.pbio.1000234.

787 38. Gahan LJ, Gould F, Heckel DG. Identification of a Gene Associated with Bt Resistance in Heliothis
788 virescens. *Science*. American Association for the Advancement of Science; 2001; doi:
789 10.1126/science.1060949.

790 39. Chen S, Li X. Transposable elements are enriched within or in close proximity to xenobiotic-
791 metabolizing cytochrome P450 genes. *BMC Evol Biol*. 2007; doi: 10.1186/1471-2148-7-46.

792 40. González J, Lenkov K, Lipatov M, Macpherson JM, Petrov DA. High Rate of Recent Transposable
793 Element–Induced Adaptation in Drosophila melanogaster. *PLOS Biol*. Public Library of Science; 2008;
794 doi: 10.1371/journal.pbio.0060251.

795 41. González J, Karasov TL, Messer PW, Petrov DA. Genome-Wide Patterns of Adaptation to
796 Temperate Environments Associated with Transposable Elements in Drosophila. *PLOS Genet*. Public
797 Library of Science; 2010; doi: 10.1371/journal.pgen.1000905.

798 42. Itokawa K, Komagata O, Kasai S, Okamura Y, Masada M, Tomita T. Genomic structures of
799 Cyp9m10 in pyrethroid resistant and susceptible strains of Culex quinquefasciatus. *Insect Biochem*
800 *Mol Biol*. 2010; doi: 10.1016/j.ibmb.2010.06.001.

801 43. Hof AE van't, Campagne P, Rigden DJ, Yung CJ, Lingley J, Quail MA, et al.. The industrial melanism
802 mutation in British peppered moths is a transposable element. *Nature*. Nature Publishing Group;
803 2016; doi: 10.1038/nature17951.

804 44. Feschotte C. Transposable elements and the evolution of regulatory networks. *Nat Rev Genet*.
805 Nature Publishing Group; 2008; doi: 10.1038/nrg2337.

806 45. Ellison CE, Bachtrog D. Dosage Compensation via Transposable Element Mediated Rewiring of a
807 Regulatory Network. *Science*. American Association for the Advancement of Science; 2013; doi:
808 10.1126/science.1239552.

809 46. Santos ME, Braasch I, Boileau N, Meyer BS, Sauteur L, Böhne A, et al.. The evolution of cichlid fish
810 egg-spots is linked with a cis -regulatory change. *Nat Commun*. Nature Publishing Group; 2014; doi:
811 10.1038/ncomms6149.

812 47. Alfsnes K, Leinaas HP, Hessen DO. Genome size in arthropods; different roles of phylogeny,
813 habitat and life history in insects and crustaceans. *Ecol Evol*. 2017; doi:
814 https://doi.org/10.1002/ece3.3163.

48. : Trichoptera World Checklist. https://entweb.sites.clemson.edu/database/trichopt/ Accessed 2021 May 8.

49. Wiggins GB, Mackay RJ. Some Relationships between Systematics and Trophic Ecology in Nearctic Aquatic Insects, with Special Reference to Trichoptera. *Ecology*. 1978; doi: https://doi.org/10.2307/1938234.

50. Mackay RJ, Wiggins GB. Ecological Diversity in Trichoptera. *Annu Rev Entomol*. 1979; doi: 10.1146/annurev.en.24.010179.001153.

51. Gurevich A, Saveliev V, Vyahhi N, Tesler G. QUAST: Quality assessment tool for genome assemblies. *Bioinforma Oxf Engl*. 2013; doi: 10.1093/bioinformatics/btt086.

52. Simão F, Waterhouse R, Ioannidis P, Zdobnov E. BUSCO: Assessing genome assembly and annotation completeness with single-copy orthologs. *Bioinforma Oxf Engl*. 2015; doi: 10.1093/bioinformatics/btv351.

53. Waterhouse RM, Seppey M, Simão FA, Manni M, Ioannidis P, Klioutchnikov G, et al.. BUSCO Applications from Quality Assessments to Gene Prediction and Phylogenomics. *Mol Biol Evol*. 2018; doi: 10.1093/molbev/msx319.

54. Laetsch DR, Blaxter ML. BlobTools: Interrogation of genome assemblies. *F1000Research*. 2017; doi: 10.12688/f1000research.12232.1.

55. Heckenhauer J, Frandsen PB, Gupta DK, Paule J, Prost S, Schell T, et al.. Annotated Draft Genomes of Two Caddisfly Species Plectrocnemia conspersa CURTIS and Hydropsyche tenuis NAVAS (Insecta: Trichoptera). *Genome Biol Evol*. 2019; doi: 10.1093/gbe/evz264.

56. Thomas JA, Frandsen PB, Prendini E, Zhou X, Holzenthal RW. A multigene phylogeny and timeline for Trichoptera (Insecta). *Syst Entomol*. 2020; doi: https://doi.org/10.1111/syen.12422.

57. Schell T, Feldmeyer B, Schmidt H, Greshake B, Tills O, Truebano M, et al.. An Annotated Draft Genome for Radix auricularia (Gastropoda, Mollusca). *Genome Biol Evol*. 2017; doi: 10.1093/gbe/evx032.

58. Hanrahan SJ, Johnston JS. New genome size estimates of 134 species of arthropods. *Chromosome Res Int J Mol Supramol Evol Asp Chromosome Biol*. 2011; doi: 10.1007/s10577-011-9231-6.

59. Yu Y-S, Jin S, Cho N, Lim J, Kim C-H, Lee S-G, et al.. Genome Size Estimation of Callipogon relictus Semenov (Coleoptera: Cerambycidae), an Endangered Species and a Korea Natural Monument. *Insects*. Multidisciplinary Digital Publishing Institute; 2021; doi: 10.3390/insects12020111.

60. Hare EE, Johnston JS. Genome size determination using flow cytometry of propidium iodide-stained nuclei. *Methods Mol Biol Clifton NJ*. 2011; doi: 10.1007/978-1-61779-228-1_1.

61. Austin CM, Tan MH, Harrisson KA, Lee YP, Croft LJ, Sunnucks P, et al.. De novo genome assembly and annotation of Australia's largest freshwater fish, the Murray cod (Maccullochella peelii), from Illumina and Nanopore sequencing read. *GigaScience*. 2017; doi: 10.1093/gigascience/gix063.

62. Pflug JM, Holmes VR, Burrus C, Johnston JS, Maddison DR. Measuring Genome Sizes Using Read-Depth, k-mers, and Flow Cytometry: Methodological Comparisons in Beetles (Coleoptera). *G3 GenesGenomesGenetics*. 2020; doi: 10.1534/g3.120.401028.

853 63. Benjamini Y, Speed TP. Summarizing and correcting the GC content bias in high-throughput
854 sequencing. *Nucleic Acids Res*. 2012; doi: 10.1093/nar/gks001.

855 64. Ranallo-Benavidez TR, Jaron KS, Schatz MC. GenomeScope 2.0 and Smudgeplot for reference-free
856 profiling of polyploid genomes. *Nat Commun*. 2020; doi: 10.1038/s41467-020-14998-3.

857 65. Bennett MD, Riley R. The duration of meiosis. *Proc R Soc Lond B Biol Sci*. Royal Society; 1971; doi:
858 10.1098/rspb.1971.0066.

859 66. Cavalier-Smith T. Nuclear volume control by nucleoskeletal DNA, selection for cell volume and cell
860 growth rate, and the solution of the DNA C-value paradox. *J Cell Sci*. 1978; doi: 10.1242/jcs.34.1.247.

861 67. Gregory TR, Hebert PDN. The Modulation of DNA Content: Proximate Causes and Ultimate
862 Consequences. *Genome Res*. 1999; doi: 10.1101/gr.9.4.317.

863 68. CAVALIER-SMITH T. Economy, Speed and Size Matter: Evolutionary Forces Driving Nuclear
864 Genome Miniaturization and Expansion. *Ann Bot*. 2005; doi: 10.1093/aob/mci010.

865 69. Thomas GWC, Dohmen E, Hughes DST, Murali SC, Poelchau M, Glastad K, et al.. Gene content
866 evolution in the arthropods. *Genome Biol*. 2020; doi: 10.1186/s13059-019-1925-7.

867 70. Nakatani Y, McLysaght A. Macrosynteny analysis shows the absence of ancient whole-genome
868 duplication in lepidopteran insects. *Proc Natl Acad Sci U S A*. 2019; doi: 10.1073/pnas.1817937116.

869 71. Li Z, Tiley GP, Rundell RJ, Barker MS. Reply to Nakatani and McLysaght: Analyzing deep
870 duplication events. *Proc Natl Acad Sci*. National Academy of Sciences; 2019; doi:
871 10.1073/pnas.1819227116.

872 72. Roelofs D, Zwaenepoel A, Sistermans T, Nap J, Kampfraath AA, Van de Peer Y, et al.. Multi-faceted
873 analysis provides little evidence for recurrent whole-genome duplications during hexapod evolution.
874 *BMC Biol*. 2020; doi: 10.1186/s12915-020-00789-1.

875 73. Lamichhaney S, Catullo R, Keogh JS, Clulow S, Edwards SV, Ezaz T. A bird-like genome from a frog:
876 Mechanisms of genome size reduction in the ornate burrowing frog, Platyplectrum ornatum. *Proc*
877 *Natl Acad Sci*. National Academy of Sciences; 2021; doi: 10.1073/pnas.2011649118.

878 74. Capy P, Gasperi G, Biémont C, Bazin C. Stress and transposable elements: co-evolution or useful
879 parasites? *Heredity*. 2000; doi: 10.1046/j.1365-2540.2000.00751.x.

880 75. Pecinka A, Dinh HQ, Baubec T, Rosa M, Lettner N, Scheid OM. Epigenetic Regulation of Repetitive
881 Elements Is Attenuated by Prolonged Heat Stress in Arabidopsis. *Plant Cell*. 2010; doi:
882 10.1105/tpc.110.078493.

883 76. Tittel-Elmer M, Bucher E, Broger L, Mathieu O, Paszkowski J, Vaillant I. Stress-Induced Activation
884 of Heterochromatic Transcription. *PLOS Genet*. Public Library of Science; 2010; doi:
885 10.1371/journal.pgen.1001175.

886 77. Wiggins G. Larvae of the North American Caddisfly Genera (Trichoptera). Larvae North Am.
887 Caddisfly Genera Trichoptera. University of Toronto Press;

888 78. Wiggins GB, Information CI for S and T, Museum RO. Caddisflies: The Underwater Architects.
889 University of Toronto Press;

79. Dijkstra K-DB, Monaghan MT, Pauls SU. Freshwater biodiversity and aquatic insect diversification. *Annu Rev Entomol*. 2014; doi: 10.1146/annurev-ento-011613-161958.

80. Kordiš D, Lovšin N, Gubenšek F. Phylogenomic Analysis of the L1 Retrotransposons in Deuterostomia. *Syst Biol*. 2006; doi: 10.1080/10635150601052637.

81. Warren IA, Naville M, Chalopin D, Levin P, Berger CS, Galiana D, et al.. Evolutionary impact of transposable elements on genomic diversity and lineage-specific innovation in vertebrates. *Chromosome Res*. 2015; doi: 10.1007/s10577-015-9493-5.

82. Suh A, Churakov G, Ramakodi MP, Platt RN II, Jurka J, Kojima KK, et al.. Multiple Lineages of Ancient CR1 Retroposons Shaped the Early Genome Evolution of Amniotes. *Genome Biol Evol*. 2015; doi: 10.1093/gbe/evu256.

83. Grandi FC, An W. Non-LTR retrotransposons and microsatellites. *Mob Genet Elem*. Taylor & Francis; 2013; doi: 10.4161/mge.25674.

84. Mackay TFC. Transposable elements and fitness in Drosophila melanogaster. *Genome*. NRC Research Press Ottawa, Canada; 2011; doi: 10.1139/g89-046.

85. Pasyukova EG, Nuzhdin SV, Morozova TV, Mackay TFC. Accumulation of Transposable Elements in the Genome of Drosophila melanogaster is Associated with a Decrease in Fitness. *J Hered*. 2004; doi: 10.1093/jhered/esh050.

86. Langley CH, Montgomery E, Hudson R, Kaplan N, Charlesworth B. On the role of unequal exchange in the containment of transposable element copy number. *Genet Res*. Cambridge University Press; 1988; doi: 10.1017/S0016672300027695.

87. Hollister JD, Gaut BS. Epigenetic silencing of transposable elements: A trade-off between reduced transposition and deleterious effects on neighboring gene expression. *Genome Res*. 2009; doi: 10.1101/gr.091678.109.

88. Lee YCG, Karpen GH. Pervasive epigenetic effects of Drosophila euchromatic transposable elements impact their evolution. Nordborg M, editor. *eLife*. eLife Sciences Publications, Ltd; 2017; doi: 10.7554/eLife.25762.

89. Kraaijeveld K. Genome Size and Species Diversification. *Evol Biol*. 2010; doi: 10.1007/s11692-010-9093-4.

90. Cosby RL, Chang N-C, Feschotte C. Host–transposon interactions: conflict, cooperation, and cooption. *Genes Dev*. 2019; doi: 10.1101/gad.327312.119.

91. Hardie DC, Hebert PD. Genome-size evolution in fishes. *Can J Fish Aquat Sci*. NRC Research Press Ottawa, Canada; 2011; doi: 10.1139/f04-106.

92. Rees DJRJ, Dufresne FD, Glémet HG, Belzile CB. Amphipod genome sizes: first estimates for Arctic species reveal genomic giants. *Genome*. 2007; doi: 10.1139/G06-155.

93. Dufresne F, Jeffery N. A guided tour of large genome size in animals: what we know and where we are heading. *Chromosome Res*. 2011; doi: 10.1007/s10577-011-9248-x.

94. Lertzman-Lepofsky G, Mooers AØ, Greenberg DA. Ecological constraints associated with genome size across salamander lineages. *Proc R Soc B Biol Sci*. Royal Society; 2019; doi: 10.1098/rspb.2019.1780.

95. Vinogradov AE. Larger genomes for molluskan land pioneers. *Genome*. 2000; doi: 10.1139/g99-063.

96. Hotaling S, Sproul JS, Heckenhauer J, Powell A, Larracuente AM, Pauls SU, et al.. Long-reads are revolutionizing 20 years of insect genome sequencing. *Genome Biol Evol*. 2021; doi: 10.1093/gbe/evab138.

97. Miller SA, Dykes DD, Polesky HF. A simple salting out procedure for extracting DNA from human nucleated cells. *Nucleic Acids Res*. 16:12151988;

98. Ruan J, Li H. Fast and accurate long-read assembly with wtdbg2. *Nat Methods*. Nature Publishing Group; 2020; doi: 10.1038/s41592-019-0669-3.

99. Walker BJ, Abeel T, Shea T, Priest M, Abouelliel A, Sakthikumar S, et al.. Pilon: An Integrated Tool for Comprehensive Microbial Variant Detection and Genome Assembly Improvement. *PLOS ONE*. Public Library of Science; 2014; doi: 10.1371/journal.pone.0112963.

100. Zimin AV, Puiu D, Luo M-C, Zhu T, Koren S, Marçais G, et al.. Hybrid assembly of the large and highly repetitive genome of Aegilops tauschii, a progenitor of bread wheat, with the MaSuRCA mega-reads algorithm. *Genome Res*. 2017; doi: 10.1101/gr.213405.116.

101. Bankevich A, Nurk S, Antipov D, Gurevich AA, Dvorkin M, Kulikov AS, et al.. SPAdes: A New Genome Assembly Algorithm and Its Applications to Single-Cell Sequencing. *J Comput Biol*. 2012; doi: 10.1089/cmb.2012.0021.

102. Cantarel BL, Korf I, Robb SMC, Parra G, Ross E, Moore B, et al.. MAKER: an easy-to-use annotation pipeline designed for emerging model organism genomes. *Genome Res*. 2008; doi: 10.1101/gr.6743907.

103. Holt C, Yandell M. MAKER2: an annotation pipeline and genome-database management tool for second-generation genome projects. *BMC Bioinformatics*. 2011; doi: 10.1186/1471-2105-12-491.

104. Luo S, Tang M, Frandsen PB, Stewart RJ, Zhou X. The genome of an underwater architect, the caddisfly Stenopsyche tienmushanensis Hwang (Insecta: Trichoptera). *GigaScience*. 2018; doi: 10.1093/gigascience/giy143.

105. Haas BJ, Salzberg SL, Zhu W, Pertea M, Allen JE, Orvis J, et al.. Automated eukaryotic gene structure annotation using EVidenceModeler and the Program to Assemble Spliced Alignments. *Genome Biol*. 2008; doi: 10.1186/gb-2008-9-1-r7.

106. Lowe TM, Chan PP. tRNAscan-SE On-line: integrating search and context for analysis of transfer RNA genes. *Nucleic Acids Res*. 2016; doi: 10.1093/nar/gkw413.

107. Camacho C, Coulouris G, Avagyan V, Ma N, Papadopoulos J, Bealer K, et al.. BLAST+: architecture and applications. *BMC Bioinformatics*. 2009; doi: 10.1186/1471-2105-10-421.

108. Götz S, García-Gómez JM, Terol J, Williams TD, Nagaraj SH, Nueda MJ, et al.. High-throughput functional annotation and data mining with the Blast2GO suite. *Nucleic Acids Res*. 2008; doi: 10.1093/nar/gkn176.

109. Katoh K, Kuma K, Toh H, Miyata T. MAFFT version 5: improvement in accuracy of multiple sequence alignment. *Nucleic Acids Res*. 2005; doi: 10.1093/nar/gki198.

110. Kalyaanamoorthy S, Minh BQ, Wong TKF, von Haeseler A, Jermiin LS. ModelFinder: fast model selection for accurate phylogenetic estimates. *Nat Methods*. Nature Publishing Group; 2017; doi: 10.1038/nmeth.4285.

111. Minh BQ, Schmidt HA, Chernomor O, Schrempf D, Woodhams MD, von Haeseler A, et al.. IQ-TREE 2: New Models and Efficient Methods for Phylogenetic Inference in the Genomic Era. *Mol Biol Evol*. 2020; doi: 10.1093/molbev/msaa015.

112. Hoang DT, Chernomor O, von Haeseler A, Minh BQ, Vinh LS. UFBoot2: Improving the Ultrafast Bootstrap Approximation. *Mol Biol Evol*. 2018; doi: 10.1093/molbev/msx281.

113. Zhang C, Rabiee M, Sayyari E, Mirarab S. ASTRAL-III: polynomial time species tree reconstruction from partially resolved gene trees. *BMC Bioinformatics*. 2018; doi: 10.1186/s12859-018-2129-y.

114. Otto F. DAPI staining of fixed cells for high-resolution flow cytometry of nuclear DNA. *Methods Cell Biol*. 1990; doi: 10.1016/s0091-679x(08)60516-6.

115. Dolezel J, Binarova P, Lucretti S. Analysis of Nuclear DNA content in plant cells by Flow cytometry. *Biol Plant*. 1989; doi: 10.1007/BF02907241.

116. Wickham H. ggplot2: Elegant Graphics for Data Analysis. 2nd ed. Springer International Publishing;

117. Flynn JM, Hubley R, Goubert C, Rosen J, Clark AG, Feschotte C, et al.. RepeatModeler2 for automated genomic discovery of transposable element families. *Proc Natl Acad Sci*. National Academy of Sciences; 2020; doi: 10.1073/pnas.1921046117.

118. Novák P, Neumann P, Pech J, Steinhaisl J, Macas J. RepeatExplorer: a Galaxy-based web server for genome-wide characterization of eukaryotic repetitive elements from next-generation sequence reads. *Bioinformatics*. 2013; doi: 10.1093/bioinformatics/btt054.

119. Novák P, Ávila Robledillo L, Koblížková A, Vrbová I, Neumann P, Macas J. TAREAN: a computational tool for identification and characterization of satellite DNA from unassembled short reads. *Nucleic Acids Res*. 2017; doi: 10.1093/nar/gkx257.

120. Goubert C, Modolo L, Vieira C, ValienteMoro C, Mavingui P, Boulesteix M. De Novo Assembly and Annotation of the Asian Tiger Mosquito (Aedes albopictus) Repeatome with dnaPipeTE from Raw Genomic Reads and Comparative Analysis with the Yellow Fever Mosquito (Aedes aegypti). *Genome Biol Evol*. 2015; doi: 10.1093/gbe/evv050.

121. Negm S, Greenberg A, Larracuente AM, Sproul JS. RepeatProfiler: a pipeline for visualization and comparative analysis of repetitive DNA profiles. *bioRxiv*. Cold Spring Harbor Laboratory; 2020; doi: 10.1101/2020.05.22.111252.

122. Barker MS, Dlugosch KM, Dinh L, Challa RS, Kane NC, King MG, et al.. EvoPipes.net: Bioinformatic Tools for Ecological and Evolutionary Genomics. *Evol Bioinforma*. SAGE Publications Ltd STM; 2010; doi: 10.4137/EBO.S5861.

123. Birney E, Clamp M, Durbin R. GeneWise and Genomewise. *Genome Res*. 2004; doi: 10.1101/gr.1865504.

124. Yang Z. PAML 4: Phylogenetic Analysis by Maximum Likelihood. *Mol Biol Evol*. 2007; doi: 10.1093/molbev/msm088.

125. Cui L, Wall PK, Leebens-Mack JH, Lindsay BG, Soltis DE, Doyle JJ, et al.. Widespread genome duplications throughout the history of flowering plants. *Genome Res*. 2006; doi: 10.1101/gr.4825606.

126. Benaglia T, Chauveau D, Hunter DR, Young DS. mixtools: An R Package for Analyzing Mixture Models. *J Stat Softw*. 2009; doi: 10.18637/jss.v032.i06.

Manuscript Track Changes

Click here to access/download;Manuscript;Maintext_track_changes.docx

Genome size evolution in the diverse insect order Trichoptera

Authors

Jacqueline Heckenhauer (jacqueline.heckenhauer@senckenberg.de)[1,2*], Paul B. Frandsen (paul_frandsen@byu.edu)[1,3,4], John S. Sproul (johnssproul@gmail.com)[5,6], Zheng Li (zheng.li@austin.utexas.edu)[6,7], Juraj Paule (juraj.paule@senckenberg.de)[7,8], Amanda M. Larracuente (alarracu@ur.rochester.edu)[5], Peter J. Maughan (jeff_maughan@byu.edu)[3], Michael S. Barker (msbarker@arizona.edu)[7,6], Julio V. Schneider (julio.schneider@senckenberg.de)[2], Russell J. Stewart (russell.stewart@utah.edu)[8,9], Steffen U. Pauls (steffen.pauls@senckenberg.de)[1,2,9,10]

Affiliations

[*]corresponding author

[1]LOEWE Centre for Translational Biodiversity Genomics (LOEWE-TBG), Frankfurt, Germany,

[2]Department of Terrestrial Zoology, Senckenberg Research Institute and Natural History Museum Frankfurt, Frankfurt, Germany

[3]Department of Plant & Wildlife Sciences, Brigham Young University, Provo, UT

[4]Data Science Lab, Smithsonian Institution, Washington, DC

[5]Department of Biology, University of Rochester, Rochester, NY, USA

[6]Department of Biology, University of Nebraska Omaha, Omaha, USA

[7,6]Department of Ecology and Evolutionary Biology, University of Arizona, Tucson, USA

[8,7]Department of Botany and Molecular Evolution, Senckenberg Research Institute and Natural History Museum Frankfurt, Frankfurt, Germany

[9,8]Department of Biomedical Engineering, University of Utah, Salt Lake City, UT

[10,9]Institute for Insect Biotechnology, Justus-Liebig-University, Gießen, Germany

Abstract

Background: Genome size is implicated in form, function, and ecological success of a species. Two principally different mechanisms are proposed as major drivers of eukaryotic genome evolution and diversity: Polyploidy (i.e. whole genome duplication: WGD) or smaller duplication events and bursts in the activity of repetitive elements (RE). Here, we generated *de novo* genome assemblies of 17 caddisflies covering all major lineages of Trichoptera. Using these and previously sequenced genomes, we use caddisflies as a model for understanding genome size evolution in diverse insect lineages.

Results: We detect a ~14-fold variation in genome size across the order Trichoptera. W~~While gene age distribution analyses found no evidence that ancient WGD played a major role in genome size variation in this group, w~~e find strong evidence that repetitive element (RE) expansions, particularly those of transposable element (TEs) ~~expansions~~ are ~~the primary~~ important drivers of large caddisfly genome sizes. Using an innovative method to examine TEs associated with universal single copy orthologs (i.e., BUSCO genes), we find that TE expansions have a major impact on protein-coding gene regions, with TE-gene associations showing a linear relationship with increasing genome size. Intriguingly, we find that expanded genomes ~~and TE-gene associations~~ preferentially evolved in caddisfly clades with a higher ecological diversity (i.e. various feeding modes, diversification in variable, less stable environments).

Conclusion: Our findings provide a platform to test hypotheses about the potential evolutionary roles of TE activity and TE-gene associations, particularly in groups with high species, ecological, and functional diversities.

Key words:

biodiversity, *de novo* genome assembly, genomics, genomic diversity, genome duplication, genome size evolution, insects, repetitive elements, transposable elements, Trichoptera

Background

~~Genome size can vary widely among closely and distantly related species, though our knowledge is still scarce for many non-model groups. This is especially true for insects, which account for much of the earth's species diversity. To date 1,345 insect genome size estimates have been published, representing less than 0.15% of all described insect species (Gregory, 2005: Animal Genome Size Database: http://www.genomesize.com, last accessed 2021-04-30). Genome size in insects ranges 240-fold from 69 Mb in chironomid midges [1] to 16.5 Gb in the mountain grasshopper *Podisma pedestris* [2].~~

Genome size is a fundamental biological character. Studying its evolution may potentially leads to a better understanding of the origin and underlying processes of the myriad forms and functions of plants and animals. This diversification process ~~which~~ remains~~s~~ at the core of much biological research. ~~With~~Given their high species, ecological and functional diversities, insects are excellent models for such research. To date 1,345 insect genome size estimates have been published (Gregory, 2005: Animal Genome Size Database: http://www.genomesize.com, last accessed 2021-04-30) ranging 240-fold from 69 Mbp in chironomid midges [1] to 16.5p Gb in the mountain grasshopper *Podisma pedestris* [2].

Genome size variation relates poorly to the number of coding genes or the complexity of the organism (C-value enigma, [3],[4],[5],[6]) and evolutionary drivers of genome size variation remain a topic of ongoing debate (e.g. [7], [8], [9], [10]). Two principally different mechanisms are proposed as primary drivers of eukaryotic genome size evolution: Whole genome duplication (WGD, i.e. polyploidy) or smaller duplication events and expansion of repetitive elements (REs, [5]). While WGD is ubiquitous in plant evolution, it has been regarded as the

Formatted: Font: 12 pt
Formatted: Font: 12 pt
Formatted: Font: 12 pt
Formatted: Font: 12 pt
Formatted: Font: 12 pt
Field Code Changed
Formatted: Font: (Default) Lucida Sans, Font color: Gray-90%, Pattern: Clear (White)
Field Code Changed

exception in animals [11], [12]. However, ancient WGD has been hypothesized to be an important driver of evolution of mollusks (e.g. [13]) amphibians (e.g. [14], [15], fish (e.g. [16], [17], [18]) and arthropods (e.g. [19], [20], [21]), including multiple putative ancient large-scale gene duplications within Trichoptera [22].

RE expansions ~~is~~are ~~the primary~~an important driver of genome size variation in many eukaryotic genomes [23], [24]. The two major categories of REs are tandem repeats (e.g., satellite DNA) and mobile transposable elements (TEs). TEs are classified into class I [retrotransposons: endogenous retroviruses (ERVs), related long terminal repeat (LTR) and non-LTR retrotransposons: SINEs (Short Interspersed Nuclear Elements), LINEs (Long Interspersed Nuclear Elements)] and class II elements (DNA transposons, [25]). In insects, the known genomic proportion of TEs ranges from 1% in the antarctic midge *Belgica antarctica* [26] to 65% in the migratory locust *Locusta migratoria* [27]. Broad-scale analysis of TE abundance in insects suggests that some order-specific signatures are present, however, major shifts in TE abundance are also common at shallow taxonomic levels [28], [29], including in Trichoptera [30]. The movement and proliferation of REs can have deleterious consequences on gene function and genome stability [31], [32], [33], [34], [35]. Moreover, repeat content and abundance can turn over rapidly even over short evolutionary time scales (reviewed in [36]). This rapid evolution has consequences for genome evolution and speciation, e.g. repeat divergence causes genetic incompatibilities between even closely related species [37]. However, TEs can also be sources of genomic innovation with selective advantages for the host [38], [39], [40], [41], [42], [43] and they can contribute to global changes in gene regulatory networks [44], [45], [46]. Investigating RE dynamics in diverse clades provides a powerful lens for understanding their ~~capacity for contributing to~~ roles in genome function and evolution. Broadly studying of RE dynamics in species-rich groups with wide variation in RE activity is an important step towards efficiently identifying study systems at finer taxonomical scales (natural populations, species complexes, or recently diverged species) that are ideally suited to

Field Code Changed
Field Code Changed
Field Code Changed

advance our understanding of molecular and evolutionary mechanisms underlying genome evolution. In addition, by taking this biodiversity genomics approach, we can develop new model systems and eventually better understand links between environmental factors, genome size evolution, adaptation, and speciation ~~species-rich groups Studying REs in species-richthese groups to identify ideal study systems for focusing on finer taxonomical scales. critical for understanding how environmental factors are linked to genome evolution in natural populations~~ (see [47]).~~, species complexes, recently diverged species(see [47]).~~

With more than 16,500 species, caddisflies (Trichoptera) are among the most diverse of all aquatic insects [48]. Their species richness is reflective of their ecological diversity, including, e.g. microhabitat specialization, a full array of feeding modes, and diverse use of underwater silk secretions [49], [50]. An initial comparison of six caddisfly species found wide genome size variation in Trichoptera (ranging from 230 Mbp to 1.4 Gbp). In that study, we hypothesized that the observed variation was correlated with caddisfly phylogeny and that TEs contributed to a suborder-specific increase of genome size [30].

Here, we present a multi-faceted analysis to investigate genome size evolution in the order Trichoptera, as an example for highly diversified non-model organisms. Specifically, we (i) estimated genome size for species across the order to explore phylogenetic patterns in the distribution of genome size variation in Trichoptera and (ii) generated 17 new Trichoptera genomes to analyze, in conjunction with 9 existing genomes, the causes (WGD, TE expansions) of genome size variation in the evolution of caddisflies. Studying the genomic diversity of this highly diversified insect order adds new insights into drivers of genome size evolution with potential to shed light on how genome size is linked to form, function, and ecology.

Data Description

Genomic resources

~~Non-model insect clades are largely underrepresented in genomic research, especially aquatic insects [51], [52]. This makes studying broad trends of genome evolution, such as the evolution of genome size and the links between ecological diversification and genome evolution, very difficult. Only nine genomes were available for the order Trichoptera prior to this study. With this in mind,~~ Here, we combined long- and short-read sequencing technologies to generate 17 new *de novo* genome assemblies across a wide taxonomic range, covering all major lineages of Trichoptera. Details on sequencing coverage and assembly strategies are given in DataS1_Sup.2, DataS1_Sup.3, ~~Data S1~~ and supplementary note~~s 1-4~~3. To assess quality, we calculated assembly statistics with QUAST v5.0.2 [51], examined gene completeness with BUSCO v3.0.2 [52], [53] and screened for potential contamination with taxon-annotated GC-coverage (TAGC) plots using BlobTools v1.0 (94, supplementary Figs. S31-S47). The new genomes are of comparable or better quality than other Trichoptera genomes previously reported in terms of BUSCO completeness and contiguity (Table 1~~, Data S1~~). This study increases the number of assemblies in this order from nine to 26, nearly tripling the number of available caddisfly genomes and thus providing a valuable resource for studying genomic diversity across this ecologically diverse insect order. The annotation of these genomes predicted 6,413 to 12,927 proteins (DataS1_Sup.2~~Table~~ 1). Most of the annotated proteins (94.4% - 98.8%) showed significant sequence similarity to entries in the NCBI nr database. GO Distributions were similar to previously annotated caddisfly genomes, i.e. the major biological processes were cellular and metabolic processes. Catalytic activity was the largest subcategory in molecular function and the cell membrane subcategories were the largest cellular component (supplementary Figs. S1-S30). This project has been deposited at NCBI under BioProject ID: PRJNA558902. For accession numbers of individual assemblies see Table 1.

We downloaded existing Trichoptera genomes from GenBank (https://www.ncbi.nlm.nih.gov/genome/) or Lepbase (http://download.lepbase.org/v4/) and

used these in conjunction with our newly generated genomes to analyze genome size evolution as explained in the following sections of this manuscript.

Flow cytometry

In addition to genomic ~~resources~~sequence data, we used flow cytometry to detect genome size variation across the order. Our study increased the number of species with available flow cytometry-based genome size estimates from 4 [55] to 31. Estimates were submitted to the Animal Genome Size Database (http://www.genomesize.com).

Analysis

Genome size evolution in Trichoptera

Based on the genomes of six trichopteran species, Olsen et al. [30] found a 3-fold suborder-specific increase of genome size and hypothesized that genome size variation is correlated with their phylogeny. To test this hypothesis, we first reconstructed phylogenetic relationships by analyzing ~2,000 single-copy BUSCO genes from the 26 study species (Figs. 1 & ~~Fig.~~ 2, Fig. S48). We obtained a ~~robust~~ molecular phylogeny that was in agreement with recent phylogenetic hypotheses ([56], see supplementary note 6) and which showed that Trichoptera is divided into two suborders: Annulipalpia (Figs. 1 & 2: Clade A, blue) and Integripalpia [consisting of basal Integripalpia (Fig. 1: Clade B1-3, light green) and infraorder Phryganides (Fig. 1: clade B4, dark green)]. Trichopterans use silk to build diverse underwater structures (see illustrations Fig.1; supplementary note 6, supplementary Fig. S48). Thus, we refer to Annulipalpia as 'fixed retreat- and net-spinners', to Phryganides (Integripalpia) as 'tube case-builders', and to basal Integripalpia as 'cocoon-builders'.

We used three approaches for estimating genome size across Trichoptera: *k-mer* distribution-estimates, backmapping of sequence data to available draft genomes (as described in [57]), and flow cytometry (FCM, supplementary note 7, supplementary figures S49-S72, Data-S1_Sup.7).

FCM estimates can be affected by chromatin condensation, the proportion of cells in G0 to G1 phases [58], [59] and endoreplication in insect cells and tissues [60]. ~~while kmer~~Sequence-based estimates can be affected by repetitive elements in the genome resulting in smaller genome size estimates (e.g. [61], [55], [62]), as well as by GC-content because sequence library preparation including PCR amplification steps are associated with underrepresentation of GC and AT rich regions[63]. Bland-Altman plots (supplementary note 8, Fig. S73) revealed general agreement of all three methods in our study. However, the FCM estimates were generally higher compared to the sequence-based estimates (Fig. 1, Data-S1) and, among all three approaches, this measure is expected to be the most accurate [8]. We observe that variation among the methods increased with genome size, indicating issues potentially caused by repeat content (see Results *Repeat dynamics*).

We observed large variation in genome size across the order. Genome size tends to be lower in 'fixed retreat- and net-spinners' and 'cocoon-builders' compared to 'tube case-builders' (Fig. 1). Specifically, we observe that genome size varies ~14-fold ranging from 1C = 154 Mbp in 'cocoon-builders' (Fig. 1, B1: Hydroptilidae) to 1C = 2129 Mbp in 'tube case-builders' (Fig. 1, clade B4: Limnephilidae). Of the 29 species analyzed by FCM, *Halesus digitatus* (Fig. 1, clade B4: Limnephilidae, Intergripalpia) possessed the largest genome (1C = 2129 Mbp), while the genome of *Hydropsyche saxonica* (Fig. 1, clade A: Hydropsychidae, 'fixed retreat- and net-spinners') was the smallest (1C = 242 Mbp). Genome size estimates based on sequence-based methods (*kmer*-based and back-mapping) range from 1C = 154 - 160 Mb in *Agraylea sexmaculata* (Fig. 1, clade B1: Hydroptilidae, 'cocoon-builders') to 1C = 1238 - 1400 Mbp in *Sericostoma* sp. (Fig. 1, clade B4: Sericostomatidae, 'tube case-builders').

Repeat Dynamics

Repetitive element abundance and classification

Formatted: Font: Not Italic

Formatted: Font: Not Italic

To understand the structural basis of genome size variation across the order Trichoptera we explored repetitive element (RE) content. We found that major expansions of transposable elements (TEs) contribute to larger genomes in 'tube case-' and some 'cocoon-builders', but particularly in 'tube case-builders' with an average of ~600 Mbp of REs compared to ~138 Mbp in 'fixed retreat- and net-spinners' (Fig. 2 A, B). LINEs are the most abundant classified TEs in 'cocoon-' and 'tube case-builders' and comprise >154 Mb on average in 'tube case-builders', or an average genome proportion of 16.9% (range = 5.6–34.7%). This represents a 1.8- and 2.8-fold increase in genome proportion relative to 'cocoon-builders' and 'fixed retreat- and net-spinners', respectively. The LINE abundance of >312 Mbp in *Odontocerum albicorne* exceeds the entire assembly lengths (152–282 Mbp) of the three smallest genome assemblies (*Hydropsyche tenuis*, *Parapsyche elsis*, and *Agraylea sexmaculata*) (Fig. 2 A, B). DNA transposons also comprise large genomic fractions in both 'cocoon-' and 'tube case-builders' (averages of 54.4 Mbp and 32.8 Mbp, respectively). However, despite containing a large number of bps, they make up a smaller fraction of total bps in the genomes of 'cocoon-' and 'tube case-builders' than in 'fixed retreat- and net-spinners' (average genome proportion = 5.9%, 4.5%, and 11.1% in 'tube case-builders', 'cocoon-builders', and 'fixed retreat- and net-spinners', respectively) (Fig. 2 B), and thus cannot, by themselves, explain the larger genome sizes. SINEs, LTRs, *Penelope* (grouped with "other" repeats in Fig. 2), and satDNAs show a disproportionate increase in 'cocoon-' and 'tube case-builders', however, all categories combined make up a relatively small proportion of their genomes (all less than 3% on average in Integripalpia) (Fig. 2, B). Unclassified repeats are the most abundant repeat category across all Trichoptera, and they but also show disproportionate expansions in both 'cocoon-' and 'case-builders' relative to 'fixed retreat- and net-spinners' (Fig. 2 A, B). The general trends noted in our assembly-based analysis of REs were corroborated by our reference-free analysis of repeat abundance (Figs. S74S122, S123 supplementary note 109).

TE age distribution analysis

To test whether the observed abundance patterns of specific TEs are driven by shared ancient proliferation events or more recent/ongoing activity of the respective TEs, we analyzed TE age distribution plots~~s~~. These plots allow us to visualize specific RE classes/superfamilies that account for shifts in RE composition and abundance and infer the relative timing of those shifts based on the distribution of sequence divergence within each RE category. TE age distributions showed a high abundance of recently diverged TE sequences in 'cocoon-' and 'tube case-builders', particularly in LINEs, DNA transposons, and LTRs in which the majority of TEs for a given class show 0–10% sequence divergence within copies of a given repeat (Fig. 3). This trend was particularly pronounced among 'tube case-builders' with several species showing high abundance of LINEs and DNA transposons with 0–5% sequence divergence (Fig. 3). This pattern suggests that the observed TE expansion is due primarily to ongoing TE activity within lineages rather than a few shared bursts of activity in ancestral lineages. This is further supported by our analysis of repeat sub-classes with ~~landscape~~ age distribution plots (Fig. S124~~75~~). For example, in our study, LINE abundance is often due to the expansion of different LINE subclasses even between species in the same sub-clade (e.g., compare *Lepidostoma* with *Micrasema*, *Himalopsyche* with *Glossosoma*; Fig. S124~~75~~). We also find evidence of shared ancient bursts of SINE activity in 'cocoon-' and 'tube case-builders', although SINEs are not an abundant repeat class in any species (avg. genomic proportion=1.9% stdev=1.7%) (Fig. ~~S75~~S124).

Associations between TE sequences and protein-coding genes

During early exploration of our sequence data, we made an unexpected discovery that~~it became apparent that~~ in some lineages, universal single copy orthologs or "BUSCO genes", showed higher than expected coverage depth of mapped reads in one or more of their sequence fragments. Further analysis showed that ~~an amplification in the copy number of fragments of~~

~~sequences~~these high coverage BUSCO sequence regions are typically RE sequences (primarily TEs) that are either embedded within or located immediately adjacent to BUSCO genes, such that the BUSCO algorithm includes them in its annotation of a given gene. ~~ classified by BUSCO.~~ We refer to BUSCO genes containing these putative ~~gene~~ RE fragments ~~discovered by BUSCO~~ as 'TE-associated~~repetitive~~ BUSCOs' (supplementary Fig. S125~~76~~, supplementary note ~~10~~11). By estimating how many times they occur, we can quantitatively measure how TE-gene interactions change with changing genome size. In fact, we detected a positive linear relationship between TE-gene interactions and increasing genome size when measured with this accidently discovered metric. ~~.~~ We found major expansions of ~~repetitive~~ TE-associated BUSCOs ~~gene fragments~~ in 'cocoon-' and 'tube case-builders' (Fig. 4A) that are significantly correlated with total repeat abundance, as well as the genomic proportion of LINEs and DNA transposons (supplementary Fig. S126~~77~~). TE-associated BUSCOs comprise a relatively large fraction of total BUSCO genes in these lineages (averages of 11.2% and 21.4% of total BUSCOs in 'cocoon-' and 'tube case-builders', respectively), compared to annulipalpian lineages (avg = 6.2%). This finding highlights the major impact of REs on the composition of protein-coding genes in species with repeat-rich genomes. The BUSCO-associated ~~To test whether repetitive fragments were due to TE insertions near or in the BUSCO genes or, conversely, due to the proliferation of "true" BUSCO protein-coding gene fragments throughout the genome, we examined patterns of BUSCO gene structure in pairwise alignments of three species pairs in which one species has a repetitive fragment (i.e., the "inflated" species) and its counterpart does not (i.e., the "reference" species). 73 of 75 randomly sampled alignments were consistently missing or non-contiguous in inflated regions in the reference sequence (Fig. 4B). The number of BLAST hits in the genome assembly of an "inflated species" when using the BUSCO sequence from the "reference species" as a query was significantly fewer than when using the BUSCO sequence from the "inflated species" as a query (Fig. 4C, Data S2). Our intersect analysis aimed at (I) quantifying shifts in associations between TEs and~~

~~genic regions across Trichoptera lineages with varying repeat abundance and (II) determining if repetitive fragments were annotated as TEs. It showed that large fractions of BLAST hits for repetitive BUSCOs (when BLASTed to their own assembly) are in fact annotated transposable element sequences, especially LINEs and DNA transposons (Fig. 4D). We further validated the insertion of TEs by mapping Nanopore reads to subsets of repetitive BUSCOs, thus ensuring that junctions between repetitive BUSCOs and non-repetitive regions were embedded in full reads and do not occur disproportionately at ends of reads as would be expected in chimeric reads (see supplementary note 11, supplementary Figs. S76-S87). Taken together, these findings provide strong evidence that the repetitive fragments we observe in BUSCO genes are derived from TE sequences. These~~ sequences may represent TEs recently inserted into BUSCO genes, the remnants left behind following historical TE transposition~~insertion~~ events, or TE sequences ~~with open reading frames~~ that are immediately adjacent to ~~protein-coding genes~~ and inadvertently classified as BUSCO ~~fragments.~~sequences.

To confirm that unexpectedly high-coverage sequence regions in TE-associated BUSCOs were in fact TE-derived sequences, we compared patterns of BUSCO gene structure (though pairwise alignment) across species pairs in which high-coverage regions (i.e., putative TE sequences) were present in the BUSCO gene of one species (i.e., the "inflated" species), but absent in the homologous BUSCO of the other (i.e., the "reference" species). This analysis showed that in 73 of 75 randomly sampled alignments, reference species showed gaps or highly non-contiguous alignments in high-coverage regions of the inflated species (Fig. 4B), suggesting that sequence insertions are typically present in high-coverage sequence regions of TE-associated BUSCOs. Our subsequent BLAST analysis showed that BLASTing a TE-associated BUSCO against its own assembly produced thousands-millions of BLAST hits from many contigs (Fig. 4C). This confirmed that the indel sequence present in high-coverage regions of "inflated" species show high sequence similarity to repetitive elements elsewhere in the genome. We then used an intersect analysis on the BLAST results to confirm that the large

majority of the excessive BLAST hits overlap with RE annotations throughout the genome, most of which are TEs with LINEs and DNA transposons being most abundant (Fig. 4D, DataS2 Sup.5). Finally, we found that if we replaced the TE-associated BLAST query sequence with the homologous, but non-TE associated BUSCO from its counterpart reference species, the number of BLAST hits was fewer (Fig. 4C, Data S2_Sup.6), offering further evidence that the TE sequence insertions driving the pattern of high-coverage in read mapping excessive BLAST hits are absent in reference species and thus carriable across relatively short time scales within Trichoptera. Taken together, these findings provide strong evidence that TE sequences (especially LINEs and DNA transposons) inadvertently annotated by BUSCO can account for the high-coverage regions we observe in BUSCO genes (Fig. 4D).

Our accidental discovery that quantifying the frequency of TE-associated BUSCOs can serve as an estimate of TE-gene associations may prove useful in other systems given the wide use of BUSCO analysis in genomic studies. Finer details supporting the TE-gene association analysis are reported in ~~Given the large fraction of BUSCO genes shown to have TE fragments (averages of 11.2% and 21.4% in 'cocoon-' and 'tube case-builders', respectively, compared to 0.062% in annulipalipans), this finding highlights the impact of TEs on the composition of protein-coding genes in species with repeat-rich genomes.~~supplementary note 11.

Formatted: Not Highlight

Gene and genome duplications

Recently, a transcriptome-based study found evidence for putative ancient gene and genome duplications in hexapods, including potential WGD events in caddisflies [22], suggesting that duplication events could be responsible for some genome size variation in Trichoptera. We investigated whether this pattern persists with whole genome data and found that the age distribution of duplications in 18 genomes were significantly different compared to the background rate of gene duplication (Figs. ~~88~~ S137 & S138~~89~~). To identify if any significant peak is consistent with a potential WGD, we used mixture modeling to identify peaks in these

gene age distributions, which recovered. We found no obvious peak consistent with an ancient WGD. To further investigate potential WGD, we used Smudgeplot [64] to visualize the haplotype structure and to estimate ploidy of the genomes.

While suggesting that WGD did not play a major role in genome size variation in Trichoptera. Smudgeplot predicted most of the genomes to be diploid, four genomes with rather small genome sizes (230 Mb – 650 Mb) were predicted to be tetraploid (*Hydropsyche tenuis*, *Rhyacophila evoluta* RSS1 and HR1, *Parapsyche elsis*). However, the Genomescope 2 results indicate that these are highly homozygous samples. Low heterozygosity is a known confounder of smudgeplot analyses (see https://github.com/KamilSJaron/smudgeplot/wiki/tutorial-strawberry) because it inflates the signal of duplication when compared to the low level of heterozygosity. We therefore interpret these four putative polyploids as artifacts of low heterozygosity in the analysis.

Formatted: Font: 12 pt
Formatted: Font: 12 pt
Formatted: Font: 12 pt
Formatted: Font: 12 pt
Formatted: Paragraph
Formatted: Font: Italic
Formatted: Font: Italic
Formatted: Font: Italic
Field Code Changed
Formatted: Font: (Default) Times New Roman, 12 pt

Discussion

The drivers and evolutionary consequences of genome size evolution are a topic of ongoing debate. Several models have been proposed [8]. Some hypothesize genome size to be a (mal)adaptive trait by impacting phenotypic traits such as developmental/life history, body size and other cell-size related effects [65], [66], [67], [68] reviewed in [8]. On the other hand, neutral theories suggest that DNA accumulation occurs only by genetic drift without selective pressures playing a major role in the accumulation or loss of DNA [the mutational hazard hypothesis (MHH, [23]) and the mutational equilibrium hypothesis (MEH, [24])]. The MHH only allows for small deleterious effects for the accumulation of extra DNA which is accompanied by higher mutation rates in larger genomes [23], while the MEH focuses on the balance between insertions and deletions. It suggests that genome expansions arise by 'bursts' of duplication events or TE activity and genome shrinkage may be caused by a more constant

rate of small deletions [24].

In this study, we observe that genome size varies ~14-fold across the order Trichoptera, with lower genome size estimates in 'fixed retreat- and net-spinners' and 'cocoon-builders' compared to 'tube case-builders' and explore potential drivers of genome size evolution. Although, recent genomic studies have shown evidence of bursts of gene duplication and gene family expansion during the evolution of hexapods [22], [69] the presence of ancient genome duplication events are still ~~in~~ a subject of debate [70], [71], [72]. We found neither~~e~~ evidence for whole duplication events when computing haplotype structure and ploidy with Smudgeplot, nor evidence of ancient WGD in the gene age distribution in our Trichoptera genomes~~,~~ although we recognize that some of our current genome assemblies might be too fragmented to infer synteny. This does not mean that we can rule out that duplication events played a role in genome size evolution in Trichoptera in the past. The emergence of PacBio HiFi genomes of caddisflies (e.g., Darwin Tree of Life is currently planning to sequence 28 caddisfly genomes; https://www.darwintreeoflife.org/) will allow a deeper exploration of putative ancient duplication events in Trichoptera.

~~Future studies using chromosome-level genome assemblies are needed to allow a deeper exploration of putative ancient duplications observed by Li *et al.* [22].~~

We fou~~i~~nd evidence that TE expansions (especially LINEs) ~~are~~ were ~~the primary~~important drivers of genome size evolution in Trichoptera (Fig. 2, Figs. S122 & S123~~3~~), which is consistent with the mutational equilibrium hypothesis (MEH). ~~explained above.~~ The~~Our~~ TE age distribution analyse~~i~~s suggested~~s~~ that the high abundance of LINEs ~~is~~ was due to ongoing/recent activity occurring independently across 'cocoon-' and particularly 'tube case-builders' (Fig. 3, Fig. ~~S4~~S124). Thus, the shift to large genomes in these lineages does not appear to be due to a single (or few) shared ancient events, ~~they~~ rather they maintained dynamic turnover in composition ~~in~~ of their large genomes. Mutational bias affecting pathways tied to TE-regulation may affect insertion/deletion ratios and subsequently lead to lineage-specific

Formatted: Font: Italic

shifts in genome size equilibrium [73]. Such changes may be stochastic (e.g., due to drift), or linked to traits that evolve on independent trajectories as lineages diverge and are thereby constrained by phylogeny. Ecological factors, demographic history, and effective population size can further impact mutation rates. For example, environmental stress can trigger bursts of TE activity and elevated mutation rates [74], [75], [76] driving lineages that occupy niche space with frequent exposure to environmental stress toward increased TE loads and larger genomes. Similarly, lineages with small effective population sizes or which are prone to population bottlenecks may have higher mutation rates and/or reduced efficacy of natural selection which would otherwise purge mildly deleterious TE load.

Although our study is not designed to pinpoint specific forces maintaining large genomes in some lineages, the pattern we observe in the distribution of genome size (i.e. lower genome size estimates in 'fixed retreat- and net-spinners' and 'cocoon-builders' compared to 'tube case-builders') leads us to hypothesize that ecological factors may play a role in genome size evolution in the order. The three focal groups discussed here exhibit markedly different ecological strategies. Larvae of 'fixed retreat- and net-spinners' generally occupy relatively narrow niche space in oxygen-rich flowing-water (mostly stream/river) environments where they rely on water currents to bring food materials to their filter nets. The evolutionary innovation of tube-case making is thought to have enabled 'tube case-builders' to occupy a much greater diversity of ecological niche space by allowing them to obtain oxygen in lentic (e.g., pond, lake, marsh) environments which are much more variable in temperature and oxygen availability than lotic environments [77], [78]. This environmental instability is greater over short (daily, seasonal) and long--time scales (centuries, millennia) [79]. It is thus plausible these tube case-building lineages experience greater environmental stress and less stable population demographics that could lead to both more frequent TE bursts and reduced efficacy of natural selection in purging deleterious effects of TE expansions as described above [23], [24].

We show that TE expansions (especially LINEs and DNA transposons) in 'cocoon-' and 'tube case-builders' have a major impact on protein-coding gene regions (Fig. 4). These ~~TE~~TE-gene associations show a linear relationship with increasing genome size. This trend is particularly pronounced among 'tube case-builders' in which TE-associated BUSCOs comprise an average of 21.4% of total BUSCO genes ~~an average of 21.4% of BUSCO genes that have internal or adjacent repetitive sequences~~ (compared to 6.2% in annulipalpians). This finding corroborates other studies highlighting the role of TEs as drivers of rapid genome evolution [80], [81], [82], [83] and highlights their impact on genomic regions that have potential effects on phenotypes. Questions remain as to what evolutionary roles such changes in genic regions may play. In general, TE insertions are considered to have deleterious effects on their host's fitness activity [84], [85]. They are known to "interrupt" genes [33], pose a risk of ectopic recombination that can lead to genome rearrangements [34], [31], [86], and have epigenetic effects on neighboring sequences [87], [88]. Therefore, purifying selection keeps TEs at low frequencies [33]. However, there is growing evidence that TE activity can also be a critical source of new genetic variation driving diversification via chromosomal rearrangements and transposition events which can result in mutations [89], including examples, of co-option [90], e.g. recent research in mammals has shown that DNA transposon fragments can be co-opted to form regulatory networks with genome-wide effects on gene expression [44].

Ecological correlates with genome size are widely discussed in other taxa [91], [92], [93], [94], [95]. Caddisflies and other diverse insect lineages that feature various microhabitat specializations, feeding modes, and/or the use of silk represent evolutionary replicates with contrasting traits and dynamic genome size evolution. They thus have high potential as models for understanding links between ecology and the evolution of REs, genomes, and phenotypes. Our study lays a foundation for future work in caddisflies that investigates the potential impact of TE expansions on phenotypes and tests for evidence of co-option/adaptive impacts of TE-rich genomes against a null of neutral or slightly deleterious effects.

Potential implications

Many open questions remain as to the causes and consequences of genome size evolution. As we move forward in an era where genome assemblies are attainable for historically intractable organisms (e.g. due to constraints given large genome sizes, tissue limitations, no close reference available) we can leverage new model systems spanning a greater diversity of life to understand how genomes evolve. Here, we provide genomic resources and new genome size estimates across lineages of an underrepresented insect order that spans major variation in genome size. These data allowed us to study genome size evolution in a phylogenetic framework to reveal lineage-specific patterns in which genome size correlates strongly with phylogeny and ecological characteristics within lineages. We find that large genomes dominate lineages with a wider range of ecological variation~~that occupy unstable environments~~, and that ongoing recent TE activity appears to maintain large genomes in these lineages. This leads us to hypothesize that ecological factors may ~~play a predictable role in~~be linked to genome size evolution in this group. The future directions spawned by our findings highlight the potential for using Trichoptera and other diverse insect groups to understand the link between ecological and genomic diversity, a link that has been challenging to study with past models [8].

We also show that TE expansions are associated with increasing genome size and have an impact on protein-coding regions. These impacts have been greatest in the most species-rich and ecologically diverse caddisfly clades. While TEs are generally considered to have deleterious effects on their host's fitness activity, their roles can also be neutral or even adaptive. TE activity can be a critical source of new genetic variation and thus an important driver for diversification. Caddisflies and potentially other non-model insect groups are excellent models to test these contrasting hypotheses, as well as the potential impact of TEs on phenotypes. Using these models, especially with respect to the increasing emergence of high-quality insect

genomes [96], will allow researchers to identify recurring patterns in TE dynamics and investigate their evolutionary implications across diverse clades.

Methods

DNA extraction, library preparation, sequencing, and sequence read processing

We extracted high molecular weight genomic DNA (gDNA) from 17 individuals (15 species) of caddisfly larvae (for sampling information, see Data-S1 Sup.1) after removing the intestinal tracts using a salting-out protocol adapted from [97] as described in supplementary note ~~3~~1. We generated gDNA libraries for a low-cost high-contiguity sequencing strategy, i.e. employing a combination of short (Illumina) and long read (Nanopore or PacBio) technologies as described in supplementary notes ~~1 2~~2. For details on sequencing coverage for each specimen see Data-S1 Sup.3.

De novo genome assembly, annotation and quality assessment

We applied different assembly strategies for different datasets. First, we applied a long-read assembly method using wtdbg2 v2.4 [98] with subsequent short-read polishing with Pilon v1.22 [99]~~)~~ as this method revealed good results in previous *de novo* assemblies in caddisflies [55]. In cases where this pipeline did not meet the expected quality regarding contiguity and BUSCO completeness, we applied *de novo* hybrid assembly approaches of MaSuRCA v.3.1.1 (supplementary note ~~5~~3). Illumina-only data was assembled with SPAdes [100] explained in supplementary note 3~~)~~.

Prior to annotating the individual genomes with MAKER2 v2.31.10 [101], [102] we used RepeatModeler v2.0~~1.0.11~~ and RepeatMasker v4.~~0.7~~1.0, to identify species-specific repetitive elements in each of the assemblies, relative to RepBase libraries v20181026; www.girinst.org). Transcriptome evidence for the annotation of the individual genomes included their species-

Formatted: Font: 12 pt
Formatted: Font: 12 pt
Formatted: Font: 12 pt
Formatted: Font: Italic
Formatted: Font: 12 pt

specific or closely related *de novo* transcriptome provided by 1KITE (http://www.1kite.org/; last accessed November 11, 2019, Data-S1 Sup.9) or downloaded from Genbank as well as the cDNA and protein models from *Stenopsyche tienmushanensis* [103] and *Bombyx mori* (AR102, GenBank accession ID# GCF_000151625.1). Additional protein evidence included the uniprot-sprot database (downloaded September 25, 2018). We masked repeats based on species-specific files produced by RepeatModeler. For *ab initio* gene prediction, species specific AUGUSTUS gene prediction models as well as *Bombyx mori* SNAP gene models were provided to MAKER. The EvidenceModeler [104] and tRNAscan [105] options in MAKER were employed to produce a weighted consensus gene structure and to identify tRNAs genes. MAKER default options were utilized for BLASTN, BLASTX, TBLASTX searches. Two assemblies (*Agapetus fuscipens* GL3 and *Micrasema longulum* ML1) were not annotated because of their low contiguity. All protein sequences were assigned putative names by BlastP Protein–Protein BLAST 2.2.30+ searches [106] and were functionally annotated using command line Blast2Go v1.3.3 [107], see supplementary note 4, Figs. S1-S30).

We calculated assembly statistics with QUAST v5.0.2 [51] and examined completeness with BUSCO v3.0.2 [52], [53] using the Endopterygota odb9 dataset with the options *--long*, *–m = genome* and *–sp= fly*. A summary of the assembly statistics and BUSCO completeness is given in Table 1. The final genome assemblies and annotations were screened and filtered for potential contaminations with taxon-annotated GC-coverage (TAGC) plots using BlobTools v1.0 [54]. Details and blobplots are given in supplementary note 5 & supplementary Figs. S31-S47.

Species tree reconstruction

We used the single-copy orthologs resulting from the BUSCO analyses to generate a species tree. We first combined single-copy ortholog amino acid files from each species into a single FASTA for each ortholog. We then aligned them with the MAFFT L-INS-i algorithm [108].

We selected amino acid substitution models for each ortholog using ModelFinder (option -m mfp,[109] in IQtree v.2.0.6 [110] and estimated a maximum likelihood tree with 1000 ultrafast bootstrap replicates [111] with the BNNI correction (option -bb 1000 -bnni). We combined the best maximum likelihood tree from each gene for species tree analysis in ASTRAL-III [112]. A locus tree was inferred using the alignment file (-s) and the partition file (-S) with the settings –prefix loci and -T AUTO in IQtree. Gene and site concordance factors were calculated with IQTree using the species tree (-t), the locus tree (--gcf) and the alignment file (-s) with 100 quartets for computing the site concordance factors (--scf 100) and --prefix concord for computing the gene concordance factors. We visualized the trees using FigTree v.1.4.4 (http://tree.bio.ed.ac.uk/software/figtree/).

Genome size estimations and genome profiling

Genome size estimates of 27 species were conducted using flow cytometry (FCM) according to Otto [113] using *Lycopersicon esculentum* cv. Stupické polnítyčkové rané (2C = 1.96 pg;[114]) as internal standard and propidium iodine as stain. Additionally, we used ~~the~~ trimmed, contamination filtered short-read data (see supplementary note 2) ~~sequenced in this study,~~ to conduct genome profiling (estimation of major genome characteristics such as size, heterozygosity, and repetitiveness) using a *k-mer* distribution-based method (GenomeScope 2.0, [64]. Genome scope profiles are available online (see links to Genomescope 2 in Data-S1 Sup.4). In addition, we applied a second sequencing-based method for genome size estimates, which uses the backmapping rate of sequenced reads to the assembly and coverage distribution (backmap.pl v0.1, [57]. Details of all three methods are described in supplementary note 7**.** Coverage distribution per position and genome size estimate from backmap.pl are shown in Figs. S49-72). We assessed the congruence among the three quantitative methods of measurement (Genomescope2, Backmap.pl and FCM) with Bland-Altman-Plots using the function BlandAltmanLeh::bland.altman.plot in ggplot2 [115] in

RStudio (RStudio Team (2020). RStudio: Integrated Development for R. RStudio, PBC, Boston, MA URL http://www.rstudio.com/; supplementary note 8, Fig. S73).

Repeat dynamics

Repeat abundance and classification

We identified and classified repetitive elements in the genome assemblies of each species using RepeatModeler2.0 [116]. We annotated repeats in the contamination filtered assemblies with RepeatMasker 4.1.0 (http://www.repeatmasker.org) using the custom repeat libraries generated from RepeatModeler2 for each respective assembly with the search engine set to "ncbi" and using the -xsmall option. We converted the softmasked assembly resulting from the first RepeatMasker round into a hardmasked assembly using the lc2n.py script (https://github.com/PdomGenomeProject/repeat-masking). Finally, we re-ran RepeatMasker on the hard-masked genome with RepeatMasker's internal arthropod repeat library using -species "Arthropoda". We then merged RepeatMasker output tables from both runs by parsing them with a script (RM_table_parser_families_.py, available at https://github.com/jhcaddisfly/TE-gene_intersect_analysis) and then combined the resulting data columns for the two runs in Excel. ~~to summarize the abundance of repeat categories.~~

We also estimated repetitive element abundance and composition using RepeatExplorer2-[117], [118] and dnaPipeTE v.1.3.1 [119]~~.~~ ~~This~~ These reference-free approaches quantifies repeats directly from unassembled short-read data. ~~using clustering-based methods.~~ This ~~analysis~~ analyses allowed us to test for general consistency of patterns with our assembly-based approach described above, and to test for the presence of abundant repeat categories such as satellite DNAs which can comprise large fractions of genomes yet can be prone to poor representation in the genome assembly. Prior to analysis, ~~in RepeatExplorer2~~ we normalized contamination filtered (see supplementary note 2) input data sets ~~–~~to 0.5x coverage using

RepeatProfiler [120] and seqtk (https://github.com/lh3/seqtk), and then ran RepeatExplorer2 clustering with the Metazoa 3.0 database specified for annotation (supplementary Fig. ~~S74~~S122) and ~~,~~dnaPipeTE with the -RM_lib flag set to the Repbase v20170127 repeat library (supplementary Fig. 123).

TE age distribution analysis

We further characterized repetitive element dynamics in Trichoptera by analyzing TE landscapes, which show relative age differences among TE sequences and their genomic abundance. We used these analyses to test whether abundance patterns of specific TEs are driven by shared ancient proliferation events or more recent/ongoing activity of the respective TEs. For example, if shared ancient proliferation is driving abundance patterns of a given TE, the majority of its copies would show moderate to high sequence divergence (e.g., >10% pairwise divergence). In contrast, if abundance patterns are driven by recent/ongoing activity of a given TE, we would expect the majority of its sequences to show low sequence divergence (e.g., 0–10%). We generated TE ~~landscape~~ age distribution plots using dnaPipeTE v1.3.1 [119] with genomic coverage for each species sampled to 0.5X prior to analysis and the -RM_lib flag set to the Repbase v20170127 repeat library (supplementary Fig. ~~S75~~S124).

TE sequence associations with protein-coding genes

~~During the early exploration of our sequence data we noted that copy number profiles of some BUSCO genes showed regions of unexpected high copy number with the frequency of such genes varying widely across species. Following this observation, W~~we analyzed ~~all~~ BUSCO genes for all species to quantify the abundance of TE-associated ~~repetitive~~ BUSCOs across samples and ~~test two hypotheses that could account for the observed patterns: (I) inflated copy number of BUSCO gene fragments occur when sequences of repetitive elements (e.g. TEs) are inserted into (or immediately adjacent to genes; (II) inflated regions are BUSCO gene fragments~~

~~that have proliferated throughout the genome (e.g., by hitch-hiking with TEs). We~~ investigated associations between TEs and genic sequences in Trichoptera lineages by quantifying the abundance of ~~repetitive~~ TE-associated BUSCO ~~sequences found within or adjacent to BUSCO~~ genes (for presence and absence of TE-associated BUSCOs ~~repetitive BUSCOs~~ see Fig. S125, Data files S2). This analysis also allowed us to quantify shifts in associations between TEs and genic regions across Trichoptera lineages with varying repeat abundance. We ~~used copy number profiles to~~ identified ~~y~~ BUSCO genes with high-coverage sequence regions~~with repetitive sequences~~ based on coverage profiles and quantified their genomic abundance by using each TE-associated BUSCO ~~repetitive BUSCO~~ as a query in a BLAST search against their respective genome assembly. We then conducted intersect analysis for all unique BUSCO hits from ~~repetitive sequences~~high coverage sequences to determine if ~~repetitive fragments~~these were annotated as TEs. We calculated the total number of bases in filtered BLAST after subtracting the number of bases at the locus belonging to all 'complete' BUSCO genes and categorized ~~BUSCO repeats~~high coverage sequence regions in BUSCO genes based on their annotation status and repeat classification using custom scripts (available at https://~~app.box.com/folder/136985262731?v=trich-genomes~~ https://github.com/jhcaddisfly/TE-gene_intersect_analysis). We plotted the number of ~~BUSCO repeats~~the high coverage BUSCO sequence regions belonging to repetitive element categories (i.e., classes and subclasses) alongside plots of the relative genomic abundance of each respective category. ~~For details of this method readers are directed to supplementary note 10. To validate that the insertion of TEs are not an artefact of poor assembly quality, we mapped Nanopore reads back to repetitive BUSCO sequences and showed that junctions between repetitive BUSCOs and non-repetitive regions are embedded in full reads (see supplementary note 11, supplementary Table S1, supplementary Figs.78-87).~~ In addition, we investigated BUSCO genes with regions of ~~repetitive~~ high coverage~~sequences~~ by pairwise alignments. Specifically, we visualized alignments of BUSCOs with high coverage sequence regions

Field Code Changed
Formatted: Highlight
Formatted: Font: 12 pt
Formatted: Highlight

~~repetitive fragments~~ (i.e., the "inflated species") alongside orthologous BUSCOS that lack ~~repetitive fragments~~such regions taken from closely related species~~. To test whether repetitive fragments are due to TE insertions or to proliferation of true BUSCO gene fragments we examined patterns of BUSCO gene structure in pairwise alignments of species in which one species has a repetitive fragment (i.e., the "inflated" species) and its counterpart does not~~ (i.e., the "reference" species). ~~To reduce the likelihood that indels due to sequence divergence (i.e., not due to REs) would complicate the analysis we conducted this test with three pairs of closely related species: *Micrasema longulum* and *Micrasema minimum*; *Rhyacophila brunnea* and *Rhyacophila evoluta*; and *Hesperophylax magnus* and *Halesus radiatus*. We predicted that if repetitive regions were due to RE sequence insertion (i.e., Hyp1 above) that inflated species would show indels at coordinates of repetitive fragments relative to reference species. If repetitive regions were due to proliferation of true BUSCO sequence (i.e., Hyp2 above) we predicted that alignments would be contiguous over the repetitive region.~~ We further tested this prediction by taking the set of BUSCOs that ~~are only~~only exhibited ~~repetitive~~high coverage regions in the inflated species and contrasted results of two BLAST searches followed by an intersect analysis. A detailed description of this method is provided in supplementary note ~~9~~11.

Gene and Genome duplications

Inference of WGDs from gene age distributions

To recover signal from potential WGDs, for each genome, we used the DupPipe pipeline to construct gene families and estimate the age distribution of gene duplications [121], https://bitbucket.org/barkerlab/evopipes/src/master/). We translated DNA sequences and identified ORFs by comparing the Genewise [122] alignment to the best- hit protein from a collection of proteins from 24 metazoan genomes from Metazome v3.0. For all DupPipe runs, we used protein- guided DNA alignments to align our nucleic acid sequences while maintaining the ORFs. We estimated synonymous divergence (K_s) using PAML with the F3X4

model [123] for each node in the gene family phylogenies. We first identified taxa with potential WGDs by comparing their paralog ages to a simulated null distribution without ancient WGDs using a K-S goodness-of-fit test [124]. We then used mixture modeling to identify if any significant peaks consistent with a potential WGD and to estimate their median paralog K_s values. Significant peaks were identified using a likelihood ratio test in the boot.comp function of the package mixtools in R [125].

Visualization of genome structure to estimate ploidy using smudgeplots

We visualized the genome structure and estimated ploidy levels with smudgeplot. For this purpose, we extracted genomic kmers from kmer counts produced with jellyfish (as described above in "Genome size estimation and genome profiling") using "jellyfish dump" with coverage thresholds previously estimated from kmer histograms using the smudgeplot.py script. We computed the set of kmer pairs with the Smudgeplot tool hetkmers. After generating the list of kmer pair coverages, we generated smudgeplots using the coverage of the kmer pairs and the "plot" tool within Smudgeplot. Ploidy as well as the haploid kmer coverage was estimated directly from the data and compared to the estimates reported by Genomescope2 (see table XXX). Details of the method and smudgeplots are given in XXX.

Formatted: Font: Italic

Formatted: Font: Not Bold

Formatted: Font: 12 pt

Formatted: Font: 12 pt

Formatted: Font: 12 pt

Formatted: Font: 12 pt

Formatted: Font: 12 pt

Formatted: Font: 12 pt

Formatted: Font: 12 pt

Formatted: Font: 12 pt, Highlight

Formatted: Font: 10 pt, Font color: Custom Color(RGB(36,41,47))

Figures Legends:

Fig. 1: Ecological diversity (right) and genome size (left) in caddisflies. Phylogenetic relationships derived from ASTRAL-III analyses using single BUSCO genes. Goeridae, which was not included in the BUSCO gene set, was placed according to [56]. ASTRAL support values (local posterior probabilities) higher than 0.9 are given for each node. The placement of Hydroptilidae (clade B1) was ambiguous. Since its placement was poorly supported in our analyses, we placed it according to Thomas et al. [56]. ~~Nodes with less than 0.9 support were collapsed (B1).~~ Taxa were collapsed to family level. Trichoptera are divided into two suborders:

Formatted: English (United States)

Formatted: English (United States)

673 Annulipalpia (‘fixed retreat- and net-spinners’, clade A: blue) and Intergripalpia (clade B:
674 green) which includes basal Integripalpia (‘cocoon-builders’, clades B1-B3, dark green) and
675 Phryganides or ‘tube case-builders’ (clade B4: light green). ‘Cocoon-builders’ are divided into
676 ‘purse case’- (clade B1), ‘tortoise case-building’ (clade B2) and ‘free-living’ (clade B3)
677 families. –Genome size estimates based on different methods (Genomescope2: orange,
678 Backmap.pl: black, Flow Cytometry (FCM): brown) are given for various caddisfly families.
679 Each dot corresponds to a mean estimate of a species. For detailed information on the species
680 and number of individuals used in each method see Data S1 Sup.7 -Genome size - Summary.
681 Colors and clade numbers in the phylogenetic tree refer to colored boxes with illustrations. The
682 following species are illustrated by Ralph Holzenthal: a: *Hydropsyche sp.* (Hydropsychidae);
683 b: *Chimarra sp.* (Philopotamidae); c: *Stenopsyche sp.* (Stenopsychidae); d: *Polycentropus* sp.
684 (Polycentropodidae); e: *Agraylea sp.* (Hydroptilidae); f: *Glossosoma sp.* (Glossosomatidae); g:
685 *Rhyacophila sp.* (Rhyacophilidae); h: *Fabria inornata* (Phryganeidae); i: *Micrasema* sp.
686 (Brachycentridae); j:*Goera fuscula* (Goeridae); k: *Sphagnophylax meiops* (Limnephilidae); l:
687 *Psilotreta sp.* (Odontoceridae), m: *Grumicha grumicha* (Sericostomatidae).
688
689 **Fig. 2: Repeat abundance and classification in 26 caddisfly genomes.** Number of bp for each
690 repeat type is given for each caddisfly genome. A: Repeat abundance and classification.
691 Phylogenetic tree was reconstructed with ASTRAL-III using single BUSCO genes from the
692 genome assemblies. The placement of Hydroptilidae (clade B1) was ambiguous. Since its
693 placement was poorly supported in our analyses, we placed the single hydroptilid taxon
694 (*Agraylea sexmaculata*) according to Thomas et al. [56]. ~~Nodes with ASTRAL support below~~
695 ~~0.9 were collapsed.~~ Species names corresponding to the abbreviations in the tree can be found
696 in Table 1. Trichoptera are divided into two suborders: Annulipalpia (‘fixed retreat- and net-
697 spinners’, clade A: blue) and Intergripalpia (clade B: green) which includes basal Integripalpia
698 (‘cocoon-builders’, clades B1-B3, dark green) and Phryganides or ‘tube case-builders’ (clade

Formatted: Font: 12 pt

Formatted: Font: 12 pt, Italic

Formatted: Font: 12 pt

B4: light green). ‘Cocoon-builders’ are divided into ‘purse case’- (clade B1), ‘tortoise case-building’ (clade B2) and ‘free-living’ (clade B3) families. An illustration of a representative of each clade is given. The “other_repeats” category includes: rolling-circles, *Penelope*, low-complexity, simple repeats, and small RNAs. B: Boxplots summarizing shifts in the genomic proportion of RE categories in major Trichoptera lineages.

Fig. 3: Transposable element age distribution landscapes. Representative examples are chosen from major Trichoptera lineages. The y-axis shows TE abundance as a proportion of the genome (e.g., 1.0 = 1% of the genome). The x-axis shows sequence divergence relative to TE consensus sequences for major TE classes. TE classes with abundance skewed toward the left (i.e., low sequence divergence) are inferred to have a recent history of diversification relative to TE classes with right-skewed abundance. Plots were generated in dnaPipeTE. Plots for all species are shown in supplementary Fig. S1244. For tip labels of the phylogenetic tree see Fig. 2.

Fig. 4: TETE-BUSCO-gene associations in Trichoptera species. (A) Raw abundance of TE-associated BUSCO sequences BUSCO with repetitive fragments present in the assembly of ut of a total of 2442BUSCOs studied in the OrthoDB 9 Endopterygota dataset. (B) Upper plot: An example of a copy numbercoverage depth profile of a TE-associated BUSCO generepetitive BUSCO gene [(BUSCO EOG090R02Q9 from ML1 (‘inflated species’)]) which shows unexpected high coverage in the second exon putatively due to the presence of a RTE-derived sequence fragment. Lower plot: A typifying alignments between a of TE-associated BUSCOrepetitive BUSCOs to non-repetitive and its orthologous BUSCOs taken from a closely related species (‘reference species’) that lack TE-association. The non-TE-associated orthologous BUSCO shows are consistently non-contiguous alignment in regions of inflated coverage in the TE-associatedrepetitive BUSCO, consistent with the presence of a RTE-derived

sequence fragment in the TE-associated BUSCO~~repetitive BUSCO which~~ that ~~-~~is absent in the reference species. (C) Summary of total bases annotated as REs obtained from each of two BLAST searches. First, when we used BLAST to compare an TE-associated~~repetitive~~ BUSCOs ~~are BLASTed -~~against an assembly for the same species BLAST hits included megabases of annotated repeats (dark plots). Second, when non-TE-associated ~~non-repetitive o~~orthologs of the TE-associated~~repetitive~~ BUSCOs in the first search are taken from a close relative and ~~BLASTed~~ compared against the inflated species using BLAST, there is a dramatic drop in BLAST hits annotated as REs. Note log scale on the y-axis. (D) Summary of annotations for BLAST hits for classified REs when TE-associated~~repetitive~~ BUSCOs are ~~BLASTed~~ compared against an assembly of the same species using BLAST~~.~~

Data and materials availability:

This project has been deposited at NCBI under BioProject ID: PRJNA558902.

The data sets supporting the results of this article are available in the supplementary, data files S1 and S2 and at https://byu.box.com/v/trich-genomes. The data available at the link will be uploaded to GigaDB when the paper is accepted.

~~**Data and materials availability:** This project has been deposited at NCBI under BioProject ID: PRJNA558902. The data sets supporting the results of this article are available in the Supplementary, Data files S1 and S2 and at https://app.box.com/folder/136985262731?v=trich-genomes. *The data available at the link will be uploaded to GigaDB when the paper is sent out for review.*~~

Formatted: Indent: Left: 0", First line: 0"

Declarations

Consent for publication: Not applicable

Competing interests: Authors declare that they have no competing interests.

Funding

This work is a result of the LOEWE-Centre for Translational Biodiversity Genomics funded by the Hessen State Ministry of Higher Education, Research and the Arts (HMWK) that supported JH and SUP, as well as internal funds of Senckenberg Research Institute provided to JP. JSS was supported by an NSF Postdoctoral Research Fellowship in Biology (DBI-1811930) and an NIH General Medical Sciences award (R35GM119515) to AML. Sequencing was, in part, supported by BYU start-up funds to PBF and funds from the Army Research Office, Life Science Division (Award no. W911NF-13-1-0319) to RJS.

Author's contributions:

Conceptualization –JH, JSS, PBF, SUP

Data curation – JH

Formal Analysis - JH, JM, JP, JSS, PBF, ZL

Funding acquisition – AML, PBF, SUP, RJS, RJS

Investigation – JH, JP, JSS, PBF, ZL

Methodology – AML, JSS, JP, JVS, PBF

Project administration – SUP

Resources – JP, MB, PBF, SUP

Visualization - JH, JSS

Writing – original draft – JH, JSS, PBF, ZL

Writing – review & editing – AML, JH, JM, JP, JSS, JVS, MB, PBF, RJS, ,RJS, SUP, ZL

Acknowledgments

The authors thank Ralph Holzenthal for providing illustrations of larval Trichoptera and the structures they build. We thank Bob Wisseman for collecting *Himalopsyche phryganeae.*

We thank both reviewers for their insightful critiques and interest in our manuscript.

References

1. Cornette R, Gusev O, Nakahara Y, Shimura S, Kikawada T, Okuda T. Chironomid midges (Diptera, chironomidae) show extremely small genome sizes. *Zoolog Sci*. 2015; doi: 10.2108/zs140166.

2. Westerman M, Barton NH, Hewitt GM. Differences in DNA content between two chromosomal races of the grasshopper Podisma pedestris. *Heredity*. Nature Publishing Group; 1987; doi: 10.1038/hdy.1987.36.

3. Thomas CA. The genetic organization of chromosomes. *Annu Rev Genet*. Annual Reviews; 1971; doi: 10.1146/annurev.ge.05.120171.001321.

4. Bernard J. The Eukaryote Genome in Development and Evolution. Springer Netherlands;

5. GREGORY TR. The C-value Enigma in Plants and Animals: A Review of Parallels and an Appeal for Partnership. *Ann Bot*. 2005; doi: 10.1093/aob/mci009.

6. Elliott TA, Gregory TR. What's in a genome? The C-value enigma and the evolution of eukaryotic genome content. *Philos Trans R Soc B Biol Sci*. Royal Society; 2015; doi: 10.1098/rstb.2014.0331.

7. Abdel-Haleem H. The Origins of Genome Architecture. *J Hered*. 2007; doi: 10.1093/jhered/esm073.

8. Blommaert J. Genome size evolution: towards new model systems for old questions. *Proc R Soc B Biol Sci*. Royal Society; 2020; doi: 10.1098/rspb.2020.1441.

9. Canapa A, Barucca M, Biscotti MA, Forconi M, Olmo E. Transposons, Genome Size, and Evolutionary Insights in Animals. *Cytogenet Genome Res*. 2015; doi: 10.1159/000444429.

10. Petrov DA. Evolution of genome size: new approaches to an old problem. *Trends Genet TIG*. 2001; doi: 10.1016/s0168-9525(00)02157-0.

11. Orr HA. "Why Polyploidy is Rarer in Animals Than in Plants" Revisited. *Am Nat*. The University of Chicago Press; 1990; doi: 10.1086/285130.

12. Otto SP, Whitton J. Polyploid Incidence and Evolution. *Annu Rev Genet*. 2000; doi: 10.1146/annurev.genet.34.1.401.

13. Hallinan NM, Lindberg DR. Comparative analysis of chromosome counts infers three paleopolyploidies in the mollusca. *Genome Biol Evol*. 2011; doi: 10.1093/gbe/evr087.

14. Alexandrou MA, Swartz BA, Matzke NJ, Oakley TH. Genome duplication and multiple evolutionary origins of complex migratory behavior in Salmonidae. *Mol Phylogenet Evol*. 2013; doi: 10.1016/j.ympev.2013.07.026.

Formatted: Font: (Default) Times New Roman, 12 pt

15. Campbell MA, Hale MC, McKinney GJ, Nichols KM, Pearse DE. Long-Term Conservation of Ohnologs Through Partial Tetrasomy Following Whole-Genome Duplication in Salmonidae. *G3 GenesGenomesGenetics*. 2019; doi: 10.1534/g3.119.400070.

16. Woods IG, Wilson C, Friedlander B, Chang P, Reyes DK, Nix R, et al.. The zebrafish gene map defines ancestral vertebrate chromosomes. *Genome Res*. 2005; doi: 10.1101/gr.4134305.

17. Berthelot C, Brunet F, Chalopin D, Juanchich A, Bernard M, Noël B, et al.. The rainbow trout genome provides novel insights into evolution after whole-genome duplication in vertebrates. *Nat Commun*. Nature Publishing Group; 2014; doi: 10.1038/ncomms4657.

18. Glasauer SMK, Neuhauss SCF. Whole-genome duplication in teleost fishes and its evolutionary consequences. *Mol Genet Genomics MGG*. 2014; doi: 10.1007/s00438-014-0889-2.

19. Clarke TH, Garb JE, Hayashi CY, Arensburger P, Ayoub NA. Spider Transcriptomes Identify Ancient Large-Scale Gene Duplication Event Potentially Important in Silk Gland Evolution. *Genome Biol Evol*. 2015; doi: 10.1093/gbe/evv110.

20. Kenny NJ, Chan KW, Nong W, Qu Z, Maeso I, Yip HY, et al.. Ancestral whole-genome duplication in the marine chelicerate horseshoe crabs. *Heredity*. Nature Publishing Group; 2016; doi: 10.1038/hdy.2015.89.

21. Schwager EE, Sharma PP, Clarke T, Leite DJ, Wierschin T, Pechmann M, et al.. The house spider genome reveals an ancient whole-genome duplication during arachnid evolution. *BMC Biol*. 2017; doi: 10.1186/s12915-017-0399-x.

22. Li Z, Tiley GP, Galuska SR, Reardon CR, Kidder TI, Rundell RJ, et al.. Multiple large-scale gene and genome duplications during the evolution of hexapods. *Proc Natl Acad Sci*. National Academy of Sciences; 2018; doi: 10.1073/pnas.1710791115.

23. Lynch M, Conery JS. The Origins of Genome Complexity. *Science*. American Association for the Advancement of Science; 2003; doi: 10.1126/science.1089370.

24. Petrov DA. Mutational Equilibrium Model of Genome Size Evolution. *Theor Popul Biol*. 2002; doi: 10.1006/tpbi.2002.1605.

25. Wicker T, Sabot F, Hua-Van A, Bennetzen JL, Capy P, Chalhoub B, et al.. A unified classification system for eukaryotic transposable elements. *Nat Rev Genet*. Nature Publishing Group; 2007; doi: 10.1038/nrg2165.

26. Kelley JL, Peyton JT, Fiston-Lavier A-S, Teets NM, Yee M-C, Johnston JS, et al.. Compact genome of the Antarctic midge is likely an adaptation to an extreme environment. *Nat Commun*. Nature Publishing Group; 2014; doi: 10.1038/ncomms5611.

27. Wang X, Fang X, Yang P, Jiang X, Jiang F, Zhao D, et al.. The locust genome provides insight into swarm formation and long-distance flight. *Nat Commun*. Nature Publishing Group; 2014; doi: 10.1038/ncomms3957.

28. Petersen M, Armisén D, Gibbs RA, Hering L, Khila A, Mayer G, et al.. Diversity and evolution of the transposable element repertoire in arthropods with particular reference to insects. *BMC Ecol Evol*. 2019; doi: 10.1186/s12862-018-1324-9.

852 29. Gilbert C, Peccoud J, Cordaux R. Transposable Elements and the Evolution of Insects.
853 *Annu Rev Entomol*. Annual Reviews; 2021; doi: 10.1146/annurev-ento-070720-074650.

854 30. Olsen LK, Heckenhauer J, Sproul JS, Dikow RB, Gonzalesz VL, Kweskin MP, et al.. De
855 novo whole genome assemblies of Agrypnia vestita Walker, and Hesperophlax magnus Banks
856 reveal substantial repetitive element expansion in tube case-making caddisflies (Insecta:
857 Trichoptera). *Genome Biol Evol*. 2021; doi: 10.1093/gbe/evab013.

858 31. Montgomery E, Charlesworth B, Langley CH. A test for the role of natural selection in the
859 stabilization of transposable element copy number in a population of Drosophila
860 melanogaster. *Genet Res*. Cambridge University Press; 1987; doi:
861 10.1017/S0016672300026707.

862 32. Charlesworth B, Langley CH. Transposition of copia elements in Drosophila. *Nature*.
863 Nature Publishing Group; 1988; doi: 10.1038/332021b0.

864 33. Charlesworth B, Langley CH. The Population Genetics of Drosophila Transposable
865 Elements. *Annu Rev Genet*. 1989; doi: 10.1146/annurev.ge.23.120189.001343.

866 34. Montgomery EA, Huang SM, Langley CH, Judd BH. Chromosome rearrangement by
867 ectopic recombination in Drosophila melanogaster: genome structure and evolution. *Genetics*.
868 1991; doi: 10.1093/genetics/129.4.1085.

869 35. Mieczkowski PA, Lemoine FJ, Petes TD. Recombination between retrotransposons as a
870 source of chromosome rearrangements in the yeast Saccharomyces cerevisiae. *DNA Repair*.
871 2006; doi: 10.1016/j.dnarep.2006.05.027.

872 36. Ugarković Đ, Plohl M. Variation in satellite DNA profiles—causes and effects. *EMBO J*.
873 John Wiley & Sons, Ltd; 2002; doi: 10.1093/emboj/cdf612.

874 37. Ferree PM, Barbash DA. Species-Specific Heterochromatin Prevents Mitotic
875 Chromosome Segregation to Cause Hybrid Lethality in Drosophila. *PLOS Biol*. Public
876 Library of Science; 2009; doi: 10.1371/journal.pbio.1000234.

877 38. Gahan LJ, Gould F, Heckel DG. Identification of a Gene Associated with Bt Resistance in
878 Heliothis virescens. *Science*. American Association for the Advancement of Science; 2001;
879 doi: 10.1126/science.1060949.

880 39. Chen S, Li X. Transposable elements are enriched within or in close proximity to
881 xenobiotic-metabolizing cytochrome P450 genes. *BMC Evol Biol*. 2007; doi: 10.1186/1471-
882 2148-7-46.

883 40. González J, Lenkov K, Lipatov M, Macpherson JM, Petrov DA. High Rate of Recent
884 Transposable Element–Induced Adaptation in Drosophila melanogaster. *PLOS Biol*. Public
885 Library of Science; 2008; doi: 10.1371/journal.pbio.0060251.

886 41. González J, Karasov TL, Messer PW, Petrov DA. Genome-Wide Patterns of Adaptation
887 to Temperate Environments Associated with Transposable Elements in Drosophila. *PLOS*
888 *Genet*. Public Library of Science; 2010; doi: 10.1371/journal.pgen.1000905.

889 42. Itokawa K, Komagata O, Kasai S, Okamura Y, Masada M, Tomita T. Genomic structures
890 of Cyp9m10 in pyrethroid resistant and susceptible strains of Culex quinquefasciatus. *Insect*
891 *Biochem Mol Biol*. 2010; doi: 10.1016/j.ibmb.2010.06.001.

892 43. Hof AE van't, Campagne P, Rigden DJ, Yung CJ, Lingley J, Quail MA, et al.. The
893 industrial melanism mutation in British peppered moths is a transposable element. *Nature*.
894 Nature Publishing Group; 2016; doi: 10.1038/nature17951.

895 44. Feschotte C. Transposable elements and the evolution of regulatory networks. *Nat Rev*
896 *Genet*. Nature Publishing Group; 2008; doi: 10.1038/nrg2337.

897 45. Ellison CE, Bachtrog D. Dosage Compensation via Transposable Element Mediated
898 Rewiring of a Regulatory Network. *Science*. American Association for the Advancement of
899 Science; 2013; doi: 10.1126/science.1239552.

900 46. Santos ME, Braasch I, Boileau N, Meyer BS, Sauteur L, Böhne A, et al.. The evolution of
901 cichlid fish egg-spots is linked with a cis -regulatory change. *Nat Commun*. Nature Publishing
902 Group; 2014; doi: 10.1038/ncomms6149.

903 47. Alfsnes K, Leinaas HP, Hessen DO. Genome size in arthropods; different roles of
904 phylogeny, habitat and life history in insects and crustaceans. *Ecol Evol*. 2017; doi:
905 https://doi.org/10.1002/ece3.3163.

906 48. : Trichoptera World Checklist. https://entweb.sites.clemson.edu/database/trichopt/
907 Accessed 2021 May 8.

908 49. Wiggins GB, Mackay RJ. Some Relationships between Systematics and Trophic Ecology
909 in Nearctic Aquatic Insects, with Special Reference to Trichoptera. *Ecology*. 1978; doi:
910 https://doi.org/10.2307/1938234.

911 50. Mackay RJ, Wiggins GB. Ecological Diversity in Trichoptera. *Annu Rev Entomol*. 1979;
912 doi: 10.1146/annurev.en.24.010179.001153.

913 51. Gurevich A, Saveliev V, Vyahhi N, Tesler G. QUAST: Quality assessment tool for
914 genome assemblies. *Bioinforma Oxf Engl*. 2013; doi: 10.1093/bioinformatics/btt086.

915 52. Simão F, Waterhouse R, Ioannidis P, Zdobnov E. BUSCO: Assessing genome assembly
916 and annotation completeness with single-copy orthologs. *Bioinforma Oxf Engl*. 2015; doi:
917 10.1093/bioinformatics/btv351.

918 53. Waterhouse RM, Seppey M, Simão FA, Manni M, Ioannidis P, Klioutchnikov G, et al..
919 BUSCO Applications from Quality Assessments to Gene Prediction and Phylogenomics. *Mol*
920 *Biol Evol*. 2018; doi: 10.1093/molbev/msx319.

921 54. Laetsch DR, Blaxter ML. BlobTools: Interrogation of genome assemblies.
922 *F1000Research*. 2017; doi: 10.12688/f1000research.12232.1.

923 55. Heckenhauer J, Frandsen PB, Gupta DK, Paule J, Prost S, Schell T, et al.. Annotated Draft
924 Genomes of Two Caddisfly Species Plectrocnemia conspersa CURTIS and Hydropsyche
925 tenuis NAVAS (Insecta: Trichoptera). *Genome Biol Evol*. 2019; doi: 10.1093/gbe/evz264.

926 56. Thomas JA, Frandsen PB, Prendini E, Zhou X, Holzenthal RW. A multigene phylogeny
927 and timeline for Trichoptera (Insecta). *Syst Entomol*. 2020; doi:
928 https://doi.org/10.1111/syen.12422.

57. Schell T, Feldmeyer B, Schmidt H, Greshake B, Tills O, Truebano M, et al.. An Annotated Draft Genome for Radix auricularia (Gastropoda, Mollusca). *Genome Biol Evol*. 2017; doi: 10.1093/gbe/evx032.

58. Hanrahan SJ, Johnston JS. New genome size estimates of 134 species of arthropods. *Chromosome Res Int J Mol Supramol Evol Asp Chromosome Biol*. 2011; doi: 10.1007/s10577-011-9231-6.

59. Yu Y-S, Jin S, Cho N, Lim J, Kim C-H, Lee S-G, et al.. Genome Size Estimation of Callipogon relictus Semenov (Coleoptera: Cerambycidae), an Endangered Species and a Korea Natural Monument. *Insects*. Multidisciplinary Digital Publishing Institute; 2021; doi: 10.3390/insects12020111.

60. Hare EE, Johnston JS. Genome size determination using flow cytometry of propidium iodide-stained nuclei. *Methods Mol Biol Clifton NJ*. 2011; doi: 10.1007/978-1-61779-228-1_1.

61. Austin CM, Tan MH, Harrisson KA, Lee YP, Croft LJ, Sunnucks P, et al.. De novo genome assembly and annotation of Australia's largest freshwater fish, the Murray cod (Maccullochella peelii), from Illumina and Nanopore sequencing read. *GigaScience*. 2017; doi: 10.1093/gigascience/gix063.

62. Pflug JM, Holmes VR, Burrus C, Johnston JS, Maddison DR. Measuring Genome Sizes Using Read-Depth, k-mers, and Flow Cytometry: Methodological Comparisons in Beetles (Coleoptera). *G3 GenesGenomesGenetics*. 2020; doi: 10.1534/g3.120.401028.

63. Benjamini Y, Speed TP. Summarizing and correcting the GC content bias in high-throughput sequencing. *Nucleic Acids Res*. 2012; doi: 10.1093/nar/gks001.

64. Ranallo-Benavidez TR, Jaron KS, Schatz MC. GenomeScope 2.0 and Smudgeplot for reference-free profiling of polyploid genomes. *Nat Commun*. 2020; doi: 10.1038/s41467-020-14998-3.

65. Bennett MD, Riley R. The duration of meiosis. *Proc R Soc Lond B Biol Sci*. Royal Society; 1971; doi: 10.1098/rspb.1971.0066.

66. Cavalier-Smith T. Nuclear volume control by nucleoskeletal DNA, selection for cell volume and cell growth rate, and the solution of the DNA C-value paradox. *J Cell Sci*. 1978; doi: 10.1242/jcs.34.1.247.

67. Gregory TR, Hebert PDN. The Modulation of DNA Content: Proximate Causes and Ultimate Consequences. *Genome Res*. 1999; doi: 10.1101/gr.9.4.317.

68. CAVALIER-SMITH T. Economy, Speed and Size Matter: Evolutionary Forces Driving Nuclear Genome Miniaturization and Expansion. *Ann Bot*. 2005; doi: 10.1093/aob/mci010.

69. Thomas GWC, Dohmen E, Hughes DST, Murali SC, Poelchau M, Glastad K, et al.. Gene content evolution in the arthropods. *Genome Biol*. 2020; doi: 10.1186/s13059-019-1925-7.

70. Nakatani Y, McLysaght A. Macrosynteny analysis shows the absence of ancient whole-genome duplication in lepidopteran insects. *Proc Natl Acad Sci U S A*. 2019; doi: 10.1073/pnas.1817937116.

968 71. Li Z, Tiley GP, Rundell RJ, Barker MS. Reply to Nakatani and McLysaght: Analyzing
969 deep duplication events. *Proc Natl Acad Sci*. National Academy of Sciences; 2019; doi:
970 10.1073/pnas.1819227116.

971 72. Roelofs D, Zwaenepoel A, Sistermans T, Nap J, Kampfraath AA, Van de Peer Y, et al..
972 Multi-faceted analysis provides little evidence for recurrent whole-genome duplications
973 during hexapod evolution. *BMC Biol*. 2020; doi: 10.1186/s12915-020-00789-1.

974 73. Lamichhaney S, Catullo R, Keogh JS, Clulow S, Edwards SV, Ezaz T. A bird-like
975 genome from a frog: Mechanisms of genome size reduction in the ornate burrowing frog,
976 Platyplectrum ornatum. *Proc Natl Acad Sci*. National Academy of Sciences; 2021; doi:
977 10.1073/pnas.2011649118.

978 74. Capy P, Gasperi G, Biémont C, Bazin C. Stress and transposable elements: co-evolution
979 or useful parasites? *Heredity*. 2000; doi: 10.1046/j.1365-2540.2000.00751.x.

980 75. Pecinka A, Dinh HQ, Baubec T, Rosa M, Lettner N, Scheid OM. Epigenetic Regulation of
981 Repetitive Elements Is Attenuated by Prolonged Heat Stress in Arabidopsis. *Plant Cell*. 2010;
982 doi: 10.1105/tpc.110.078493.

983 76. Tittel-Elmer M, Bucher E, Broger L, Mathieu O, Paszkowski J, Vaillant I. Stress-Induced
984 Activation of Heterochromatic Transcription. *PLOS Genet*. Public Library of Science; 2010;
985 doi: 10.1371/journal.pgen.1001175.

986 77. Wiggins G. Larvae of the North American Caddisfly Genera (Trichoptera). Larvae North
987 Am. Caddisfly Genera Trichoptera. University of Toronto Press;

988 78. Wiggins GB, Information CI for S and T, Museum RO. Caddisflies: The Underwater
989 Architects. University of Toronto Press;

990 79. Dijkstra K-DB, Monaghan MT, Pauls SU. Freshwater biodiversity and aquatic insect
991 diversification. *Annu Rev Entomol*. 2014; doi: 10.1146/annurev-ento-011613-161958.

992 80. Kordiš D, Lovšin N, Gubenšek F. Phylogenomic Analysis of the L1 Retrotransposons in
993 Deuterostomia. *Syst Biol*. 2006; doi: 10.1080/10635150601052637.

994 81. Warren IA, Naville M, Chalopin D, Levin P, Berger CS, Galiana D, et al.. Evolutionary
995 impact of transposable elements on genomic diversity and lineage-specific innovation in
996 vertebrates. *Chromosome Res*. 2015; doi: 10.1007/s10577-015-9493-5.

997 82. Suh A, Churakov G, Ramakodi MP, Platt RN II, Jurka J, Kojima KK, et al.. Multiple
998 Lineages of Ancient CR1 Retroposons Shaped the Early Genome Evolution of Amniotes.
999 *Genome Biol Evol*. 2015; doi: 10.1093/gbe/evu256.

1000 83. Grandi FC, An W. Non-LTR retrotransposons and microsatellites. *Mob Genet Elem*.
1001 Taylor & Francis; 2013; doi: 10.4161/mge.25674.

1002 84. Mackay TFC. Transposable elements and fitness in Drosophila melanogaster. *Genome*.
1003 NRC Research Press Ottawa, Canada; 2011; doi: 10.1139/g89-046.

1004 85. Pasyukova EG, Nuzhdin SV, Morozova TV, Mackay TFC. Accumulation of Transposable
1005 Elements in the Genome of Drosophila melanogaster is Associated with a Decrease in Fitness.
1006 *J Hered*. 2004; doi: 10.1093/jhered/esh050.

86. Langley CH, Montgomery E, Hudson R, Kaplan N, Charlesworth B. On the role of unequal exchange in the containment of transposable element copy number. *Genet Res*. Cambridge University Press; 1988; doi: 10.1017/S0016672300027695.

87. Hollister JD, Gaut BS. Epigenetic silencing of transposable elements: A trade-off between reduced transposition and deleterious effects on neighboring gene expression. *Genome Res*. 2009; doi: 10.1101/gr.091678.109.

88. Lee YCG, Karpen GH. Pervasive epigenetic effects of Drosophila euchromatic transposable elements impact their evolution. Nordborg M, editor. *eLife*. eLife Sciences Publications, Ltd; 2017; doi: 10.7554/eLife.25762.

89. Kraaijeveld K. Genome Size and Species Diversification. *Evol Biol*. 2010; doi: 10.1007/s11692-010-9093-4.

90. Cosby RL, Chang N-C, Feschotte C. Host–transposon interactions: conflict, cooperation, and cooption. *Genes Dev*. 2019; doi: 10.1101/gad.327312.119.

91. Hardie DC, Hebert PD. Genome-size evolution in fishes. *Can J Fish Aquat Sci*. NRC Research Press Ottawa, Canada; 2011; doi: 10.1139/f04-106.

92. Rees DJRJ, Dufresne FD, Glémet HG, Belzile CB. Amphipod genome sizes: first estimates for Arctic species reveal genomic giants. *Genome*. 2007; doi: 10.1139/G06-155.

93. Dufresne F, Jeffery N. A guided tour of large genome size in animals: what we know and where we are heading. *Chromosome Res*. 2011; doi: 10.1007/s10577-011-9248-x.

94. Lertzman-Lepofsky G, Mooers AØ, Greenberg DA. Ecological constraints associated with genome size across salamander lineages. *Proc R Soc B Biol Sci*. Royal Society; 2019; doi: 10.1098/rspb.2019.1780.

95. Vinogradov AE. Larger genomes for molluskan land pioneers. *Genome*. 2000; doi: 10.1139/g99-063.

96. Hotaling S, Sproul JS, Heckenhauer J, Powell A, Larracuente AM, Pauls SU, et al.. Long-reads are revolutionizing 20 years of insect genome sequencing. *Genome Biol Evol*. 2021; doi: 10.1093/gbe/evab138.

97. Miller SA, Dykes DD, Polesky HF. A simple salting out procedure for extracting DNA from human nucleated cells. *Nucleic Acids Res*. 16:12151988;

98. Ruan J, Li H. Fast and accurate long-read assembly with wtdbg2. *Nat Methods*. Nature Publishing Group; 2020; doi: 10.1038/s41592-019-0669-3.

99. Walker BJ, Abeel T, Shea T, Priest M, Abouelliel A, Sakthikumar S, et al.. Pilon: An Integrated Tool for Comprehensive Microbial Variant Detection and Genome Assembly Improvement. *PLOS ONE*. Public Library of Science; 2014; doi: 10.1371/journal.pone.0112963.

100. Bankevich A, Nurk S, Antipov D, Gurevich AA, Dvorkin M, Kulikov AS, et al.. SPAdes: A New Genome Assembly Algorithm and Its Applications to Single-Cell Sequencing. *J Comput Biol*. 2012; doi: 10.1089/cmb.2012.0021.

101. Cantarel BL, Korf I, Robb SMC, Parra G, Ross E, Moore B, et al.. MAKER: an easy-to-use annotation pipeline designed for emerging model organism genomes. *Genome Res*. 2008; doi: 10.1101/gr.6743907.

102. Holt C, Yandell M. MAKER2: an annotation pipeline and genome-database management tool for second-generation genome projects. *BMC Bioinformatics*. 2011; doi: 10.1186/1471-2105-12-491.

103. Luo S, Tang M, Frandsen PB, Stewart RJ, Zhou X. The genome of an underwater architect, the caddisfly Stenopsyche tienmushanensis Hwang (Insecta: Trichoptera). *GigaScience*. 2018; doi: 10.1093/gigascience/giy143.

104. Haas BJ, Salzberg SL, Zhu W, Pertea M, Allen JE, Orvis J, et al.. Automated eukaryotic gene structure annotation using EVidenceModeler and the Program to Assemble Spliced Alignments. *Genome Biol*. 2008; doi: 10.1186/gb-2008-9-1-r7.

105. Lowe TM, Chan PP. tRNAscan-SE On-line: integrating search and context for analysis of transfer RNA genes. *Nucleic Acids Res*. 2016; doi: 10.1093/nar/gkw413.

106. Camacho C, Coulouris G, Avagyan V, Ma N, Papadopoulos J, Bealer K, et al.. BLAST+: architecture and applications. *BMC Bioinformatics*. 2009; doi: 10.1186/1471-2105-10-421.

107. Götz S, García-Gómez JM, Terol J, Williams TD, Nagaraj SH, Nueda MJ, et al.. High-throughput functional annotation and data mining with the Blast2GO suite. *Nucleic Acids Res*. 2008; doi: 10.1093/nar/gkn176.

108. Katoh K, Kuma K, Toh H, Miyata T. MAFFT version 5: improvement in accuracy of multiple sequence alignment. *Nucleic Acids Res.* 2005; doi: 10.1093/nar/gki198.

109. Kalyaanamoorthy S, Minh BQ, Wong TKF, von Haeseler A, Jermiin LS. ModelFinder: fast model selection for accurate phylogenetic estimates. *Nat Methods*. Nature Publishing Group; 2017; doi: 10.1038/nmeth.4285.

110. Minh BQ, Schmidt HA, Chernomor O, Schrempf D, Woodhams MD, von Haeseler A, et al.. IQ-TREE 2: New Models and Efficient Methods for Phylogenetic Inference in the Genomic Era. *Mol Biol Evol*. 2020; doi: 10.1093/molbev/msaa015.

111. Hoang DT, Chernomor O, von Haeseler A, Minh BQ, Vinh LS. UFBoot2: Improving the Ultrafast Bootstrap Approximation. *Mol Biol Evol*. 2018; doi: 10.1093/molbev/msx281.

112. Zhang C, Rabiee M, Sayyari E, Mirarab S. ASTRAL-III: polynomial time species tree reconstruction from partially resolved gene trees. *BMC Bioinformatics*. 2018; doi: 10.1186/s12859-018-2129-y.

113. Otto F. DAPI staining of fixed cells for high-resolution flow cytometry of nuclear DNA. *Methods Cell Biol*. 1990; doi: 10.1016/s0091-679x(08)60516-6.

114. Dolezel J, Binarova P, Lucretti S. Analysis of Nuclear DNA content in plant cells by Flow cytometry. *Biol Plant*. 1989; doi: 10.1007/BF02907241.

115. Wickham H. ggplot2: Elegant Graphics for Data Analysis. 2nd ed. Springer International Publishing;

116. Flynn JM, Hubley R, Goubert C, Rosen J, Clark AG, Feschotte C, et al.. RepeatModeler2 for automated genomic discovery of transposable element families. *Proc Natl Acad Sci*. National Academy of Sciences; 2020; doi: 10.1073/pnas.1921046117.

117. Novák P, Neumann P, Pech J, Steinhaisl J, Macas J. RepeatExplorer: a Galaxy-based web server for genome-wide characterization of eukaryotic repetitive elements from next-generation sequence reads. *Bioinformatics*. 2013; doi: 10.1093/bioinformatics/btt054.

118. Novák P, Ávila Robledillo L, Koblížková A, Vrbová I, Neumann P, Macas J. TAREAN: a computational tool for identification and characterization of satellite DNA from unassembled short reads. *Nucleic Acids Res*. 2017; doi: 10.1093/nar/gkx257.

119. Goubert C, Modolo L, Vieira C, ValienteMoro C, Mavingui P, Boulesteix M. De Novo Assembly and Annotation of the Asian Tiger Mosquito (Aedes albopictus) Repeatome with dnaPipeTE from Raw Genomic Reads and Comparative Analysis with the Yellow Fever Mosquito (Aedes aegypti). *Genome Biol Evol*. 2015; doi: 10.1093/gbe/evv050.

120. Negm S, Greenberg A, Larracuente AM, Sproul JS. RepeatProfiler: a pipeline for visualization and comparative analysis of repetitive DNA profiles. *bioRxiv*. Cold Spring Harbor Laboratory; 2020; doi: 10.1101/2020.05.22.111252.

121. Barker MS, Dlugosch KM, Dinh L, Challa RS, Kane NC, King MG, et al.. EvoPipes.net: Bioinformatic Tools for Ecological and Evolutionary Genomics. *Evol Bioinforma*. SAGE Publications Ltd STM; 2010; doi: 10.4137/EBO.S5861.

122. Birney E, Clamp M, Durbin R. GeneWise and Genomewise. *Genome Res*. 2004; doi: 10.1101/gr.1865504.

123. Yang Z. PAML 4: Phylogenetic Analysis by Maximum Likelihood. *Mol Biol Evol*. 2007; doi: 10.1093/molbev/msm088.

124. Cui L, Wall PK, Leebens-Mack JH, Lindsay BG, Soltis DE, Doyle JJ, et al.. Widespread genome duplications throughout the history of flowering plants. *Genome Res.* 2006; doi: 10.1101/gr.4825606.

125. Benaglia T, Chauveau D, Hunter DR, Young DS. mixtools: An R Package for Analyzing Mixture Models. *J Stat Softw*. 2009; doi: 10.18637/jss.v032.i06.

126. Weigand H, Weiss M, Cai H, Li Y, Yu L, Zhang C, et al.. Fishing in troubled waters: Revealing genomic signatures of local adaptation in response to freshwater pollutants in two macroinvertebrates. *Sci Total Environ*. 2018; doi: 10.1016/j.scitotenv.2018.03.109.

127. Ferguson L, Marlétaz F, Carter J-M, Taylor WR, Gibbs M, Breuker CJ, et al.. Ancient Expansion of the Hox Cluster in Lepidoptera Generated Four Homeobox Genes Implicated in Extra-Embryonic Tissue Formation. *PLOS Genet*. Public Library of Science; 2014; doi: 10.1371/journal.pgen.1004698.

128. Weigand H, Weiss M, Cai H, Li Y, Yu L, Zhang C, et al.. Deciphering the origin of mito-nuclear discordance in two sibling caddisfly species. *Mol Ecol*. 2017; doi: 10.1111/mec.14292.

1121

1122

Click here to access/download;Table;Table 1.docx

Table 1. Comparison of assembly and annotation statistics of all available Trichoptera Genomes. *Assemblies produced in this study.

**$N_{Arthropoda}$=2442

**Species**	**Abbre-viation**	**Accession number**	**Length (bp)**	**N50 (kbp)**	**No. of scaffolds/contigs**	**BUSCOS****
*Agapetus fuscipes**	AF	JAGTXP000000000	552,637,417	2.8	291,536	C:39.7%[S:39.2%,D:0.5%],F:35.8%,M:24.5%,n:2442
*Agraylea sexmaculata**	AS	JAGTTH000000000	196,044,125	86	7,050	C:94.3%[S:89.8%,D:4.5%],F:2.3%,M:3.4%,n:2442
Agrypnia vestita [30]	AV	JADDOH000000000	1,352,945,503	111.8	25,541	C:87.3%[S:79.0%,D:8.3%],F:5.5%,M:7.2%,n:2442
*Drusus annulatus**	DA	JAGWCC000000000	727,941,535	1,043.7	2,401	C:93.5%[S:93.0%,D:0.5%],F:3.3%,M:3.2%,n:2442
*Glossosoma conforme**	GC1	JAGTXR000000000	568,249,599	2,212.1	653	C:88.9%[S:88.0%,D:0.9%],F:2.9%,M:8.2%,n:2442
Glossosoma conforme [124]	GC2	GCA_003347265.1	604,293,666	17.1	119,821	C:74.2%[S:73.5%,D:0.7%],F:17.9%,M:7.9%,n:2442
Glyphotaelius pellucidula [125]	GP	Glyphotaelius_pellucidus_k51_scaffolds	623,431,006	1.6	461,749	C:15.7%[S:15.7%,D:0.0%],F:31.4%,M:52.9%,n:2442
*Halesus radiatus**	HR	JAHDVE000000000	973,356,502	125.2	12,484	C:85.7%[S:83.3%,D:2.4%],F:4.9%,M:9.4%,n:2442
*Himalopsyche phryganea**	HP	JAGVSL000000000	633,785,554	4,634	710	C:95.5%[S:94.8%,D:0.7%],F:2.3%,M:2.2%,n:2442
Hesperophylax magnus [30]	HM	JADDOG000000000	1,275,967,528	768.2	6,877	C:92.5%[S:85.9%,D:6.6%],F:2.7%,M:4.8%,n:2442
Hydropsyche tenuis [57]	HT	GCA_009617725.1	229,663,394	2,190.1	403	C:94.4%[S:93.5%,D:0.9%],F:3.2%,M:2.4%,n:2442

*Lepidostoma basale**	LB	JAGTTH000000000	769,208,668	1,052	1,621	C:93.9%[S:92.8%,D:1.1%],F:3.1%,M:3.0%,n:2442
Limnephilus lunatus [69]	LL	GCA_000648945.2	1,369,180,260	69.1	58,718	C:79.3%[S:74.6%,D:4.7%],F:11.7%,M:9.0%,n:2442
*Micrasema longulum**	ML2	JAGXCS000000000	668,600,304	2.5	368,330	C:78.6%[S:77.7%,D:0.9%],F:5.9%,M:15.5%,n:2442
*Micrasema longulum**	ML1	JAGVSM000000000	585,245,295	170.5	5,451	C:38.2%[S:38.0%,D:0.2%],F:31.6%,M:30.2%,n:2442
*Micrasema minimum**	MM	JAGVSQ000000000	329,257,313	69.5	7,561	C:55.4%[S:55.2%,D:0.2%],F:11.7%,M:32.9%,n:2442
*Micropterna sequax**	MS	JAGUCF000000000	778,692,278	7.9	144,286	C:43.4%[S:42.0%,D:1.4%],F:25.5%,M:31.1%,n:2442
*Odontocerum albicorne**	OA	JAGTXQ000000000	1,305,984,461	266.4	9,303	C:91.1%[S:90.1%,D:1.0%],F:4.8%,M:4.1%,n:2442
*Parapsyche elsis**	PE	JAGVSN000000000	282,185,525	5,591.7	159	C:95.0%[S:94.5%,D:0.5%],F:2.4%,M:2.6%,n:2442
*Philopotamus ludificatus**	PL	JAGXCT000000000	360,300,449	67.5	37,274	C:91.0%[S:89.4%,D:1.6%],F:5.9%,M:3.1%,n:2442
Plectrocnemia conspersa [57]	PC	GCA_009617715.1	396,695,105	869	1,614	C:93.5%[S:92.6%,D:0.9%],F:4.3%,M:2.2%,n:2442
*Rhyacophila brunnea**	RB	JAGYXB000000000	1,086,872,538	1,030.6	2,125	C:94.5%[S:91.6%,D:2.9%],F:2.8%,M:2.7%,n:2442
*Rhyacophila evoluta**	RE2	JAGVSQ000000000	565,830,460	9.9	114,057	C:71.7%[S:71.3%,D:0.4%],F:20.5%,M:7.8%,n:2442
*Rhyacophila evoluta**	RE1	JAGVSO000000000	562,550,625	9.7	111,706	C:71.7%[S:71.4%,D:0.3%],F:20.6%,M:7.7%,n:2442
Sericostoma sp.[126]	SS	GCA_003003475.1	1,015,727,762	3.2	561,698	C:26.4%[S:26.4%,D:0.0%],F:34.4%,M:39.2%,n:2442
Stenopsyche tienhuanesis [100]	ST	GCA_008973525.1	451,494,475	1,296.7	552	C:94.2%[S:90.8%,D:3.4%],F:3.4%,M:2.4%,n:2442

Click here to access/download;Figure;Figure1.pdf

Figure 1

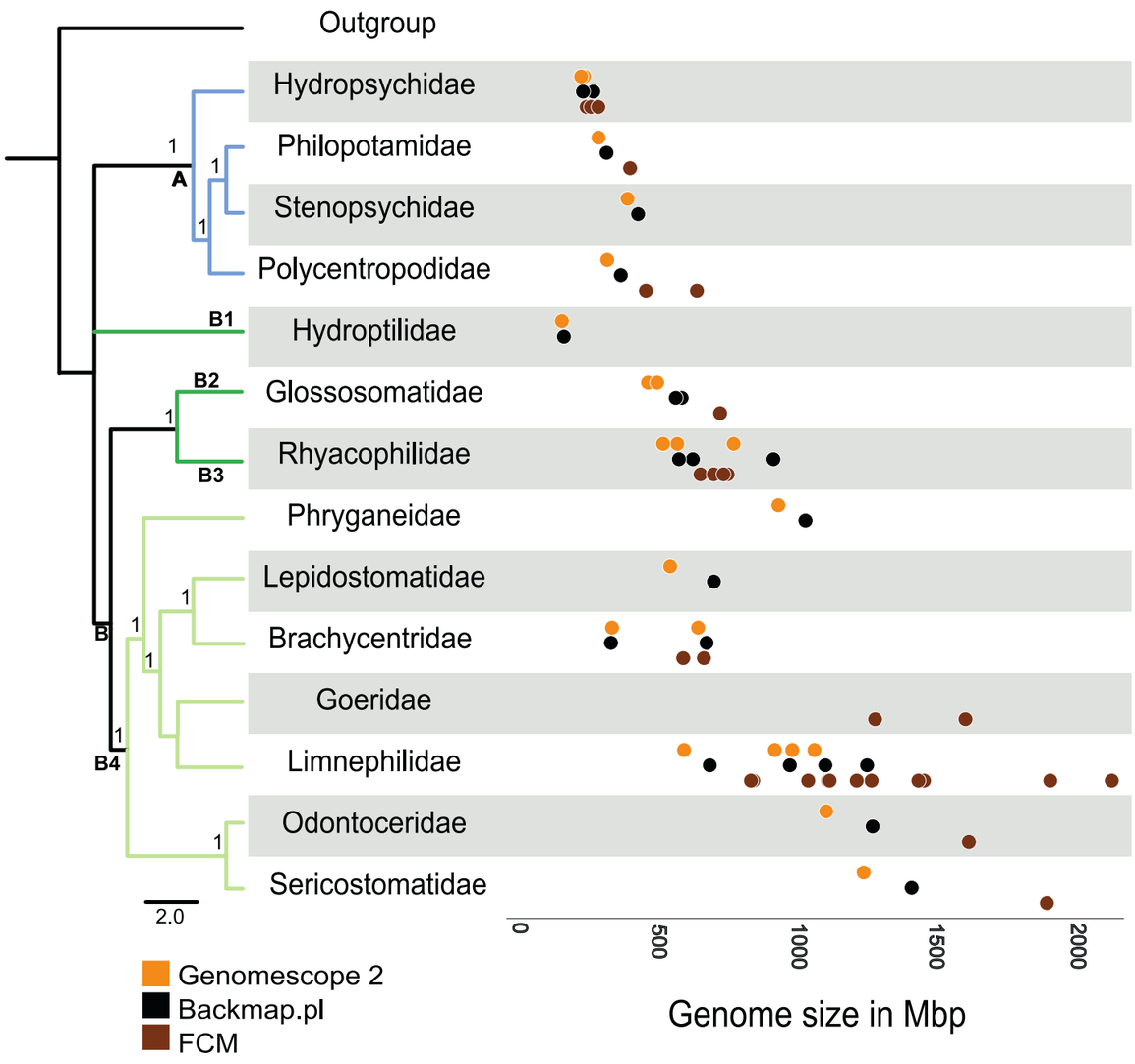

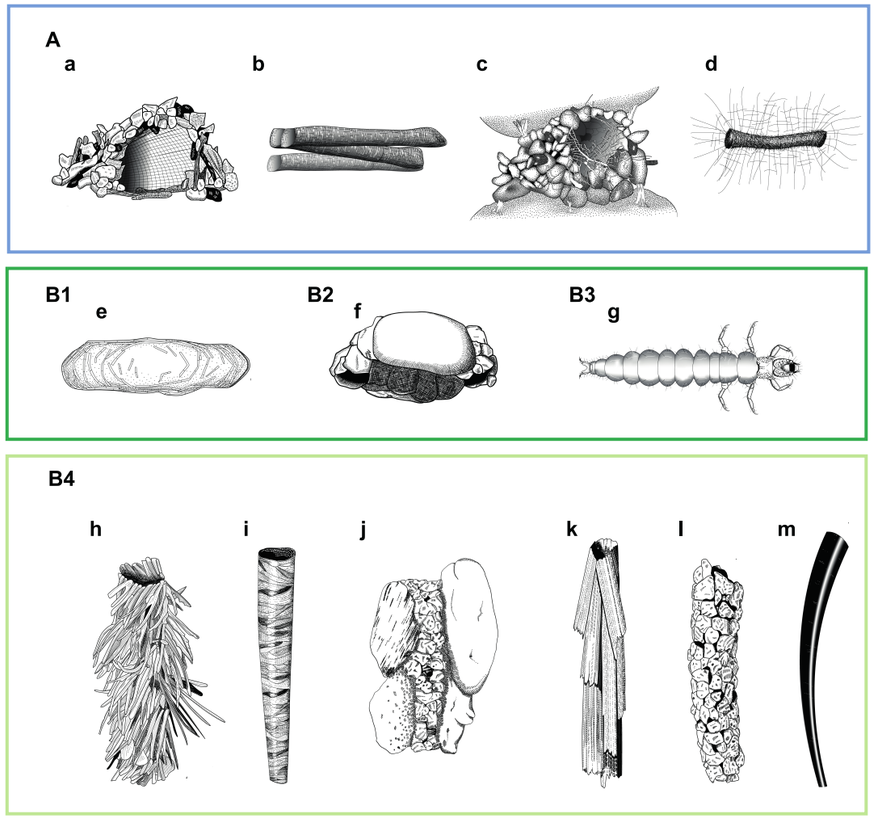

Annulipalpia (‘fixed-retreat- and net-spinners‘)
basal Integripalpia (‘cocoon-builders‘)
Phryganides (‘tube case-builders‘)
