# Supplementary material for: Genome size evolution in the diverse insect order Trichoptera
Source: Gigascience. 2022 Feb 25;11:giac011. doi: 10.1093/gigascience/giac011 (PMC8881205; doi:10.1093/gigascience/giac011)
Supplement: giac011_GIGA-D-21-00216_Revision_2 [file giac011_giga-d-21-00216_revision_2.pdf]

GigaScience

Genome size evolution in the diverse insect order Trichoptera

--Manuscript Draft--

**Manuscript Number:**	GIGA-D-21-00216R2
**Full Title:**	Genome size evolution in the diverse insect order Trichoptera
**Article Type:**	Research
**Funding Information:**	national science foundation (DBI-1811930) — Dr. John S. Sproul<br>national institutes of health (R35GM119515) — Dr. Amanda M. Larracuente<br>brigham young university — Dr. Paul B. Frandsen<br>hessen state ministry of higher education, research and the arts — Dr. Jacqueline Heckenhauer<br>hessisches ministerium für wissenschaft und kunst — Dr. Jacqueline Heckenhauer, Dr. Steffen U. Pauls<br>army research office (W911NF-13-1-0319) — Dr. Russell J. Stewart
**Abstract:**	Background<br><br>Genome size is implicated in form, function, and ecological success of a species. Two principally different mechanisms are proposed as major drivers of eukaryotic genome evolution and diversity: Polyploidy (i.e., whole genome duplication: WGD) or smaller duplication events and bursts in the activity of repetitive elements (RE). Here, we generated de novo genome assemblies of 17 caddisflies covering all major lineages of Trichoptera. Using these and previously sequenced genomes, we use caddisflies as a model for understanding genome size evolution in diverse insect lineages.<br><br>Results<br><br>We detect a ~14-fold variation in genome size across the order Trichoptera. We find strong evidence that repetitive element (RE) expansions, particularly those of transposable elements (TEs), are important drivers of large caddisfly genome sizes. Using an innovative method to examine TEs associated with universal single copy orthologs (i.e., BUSCO genes), we find that TE expansions have a major impact on protein-coding gene regions, with TE-gene associations showing a linear relationship with increasing genome size. Intriguingly, we find that expanded genomes preferentially evolved in caddisfly clades with a higher ecological diversity (i.e., various feeding modes, diversification in variable, less stable environments).<br><br>Conclusion<br><br>Our findings provide a platform to test hypotheses about the potential evolutionary roles of TE activity and TE-gene associations, particularly in groups with high species, ecological, and functional diversities.
**Corresponding Author:**	Jacqueline Heckenhauer, Ph.D.<br>LOEWE-TBG: LOEWE-Zentrum fur Translationale Biodiversitatsgenomik<br>Frankfurt am Main, Hesse GERMANY
**Corresponding Author Secondary Information:**	
**Corresponding Author's Institution:**	LOEWE-TBG: LOEWE-Zentrum fur Translationale Biodiversitatsgenomik
**Corresponding Author's Secondary Institution:**	
**First Author:**	Jacqueline Heckenhauer, Ph.D.
**First Author Secondary Information:**	

Powered by Editorial Manager® and ProduXion Manager® from Aries Systems Corporation

**Order of Authors:**	Jacqueline Heckenhauer, Ph.D.
	Paul B. Frandsen
	John S. Sproul
	Zheng Li
	Juraj Paule
	Amanda M. Larracuente
	Peter J. Maughan
	Michael S. Barker
	Julio V. Schneider
	Russell J. Stewart
	Steffen U. Pauls
**Order of Authors Secondary Information:**	
**Response to Reviewers:**	Cover letter<br>Dear Hongling Zhou, and, dear Editorial Board,<br><br>We would like to re-submit our revised manuscript entitled “Genome size evolution in the diverse insect order Trichoptera (caddisflies)” for review after incorporating feedback from the reviewer.<br><br>We thank both reviewers for reading our revised version of the manuscript. We, especially, thank reviewer 1 for their constructive feedback which we have carefully considered.<br><br>We made changes to the manuscript and believe that the manuscript has improved as a result. The revised manuscript addresses the concerns raised and was submitted via the submission portal (with and without using Track Changes). A point-by-point response to the reviewers’ suggestions can be found below.<br><br>Again, we thank you for the opportunity to revise our manuscript. Please let us know if there is anything else you require from us.<br><br>Sincerely,<br><br>Jacqueline Heckenhauer on behalf of all authors<br><br>Point-by-point 'Response to Reviewers':<br><br>Reviewer reports:<br>Reviewer #1: I thank the authors for their well-written, thorough, and detailed responses to my concerns and those of the other reviewer. I believe most of my concerns have been satisfactorily addressed. I find the revised manuscript suitable for publication, after one minor issue is further addressed (see below). The scale of data and analyses in this paper is no small feat.<br>Minor comment:<br>I appreciate that the authors have run smudgeplot analyses on their data, but I do notice a few inconsistencies in coverage estimates between those based on sequencing effort and the backmapping approach, and the kmer approaches. It is, therefore, in my opinion, still worth comparing the kcov to actual sequencing effort for the samples where smudgeplots are unclear and/or inconsistent with the other

	coverage estimates. I realise these two methodological approaches are based on different data streams (kmers versus assemblies), but considering their shared origin, they are still worth comparing.<br>See example below:<br>O. albicorne has varying coverage estimates (sequencing effort, 2n: 46.7, backmapping, 2n: 58, genomescope, 1n: 24.8, smudgeplot, 1n: 53.8)<br>If you convert sequencing-based coverage to kcov:<br>Kcov, 1n =(((read length -k + 1)* coverage)/read length)/2<br>This gives a kcov 1n estimate between 20.3 and 25.1, more in line with the genomescope estimate, suggesting that smudgeplot should be re-run with manually entered kcov as predicted by genomescope<br>Other incongruent examples, where at least one of the coverage estimates is inconsistent, include: H. phryganea, H. magnus, L. basale, andM. longulum<br>This comparison, and the adjustment of the genomescope kcov, should tidy up a few lose ends and overall help support more of the results of the various analyses. This may suggest that some of these species might not be diploid, but I do agree with the authors that further sequencing would need to be done to confirm this, and this is outside the scope of this manuscript. A brief mention of these different lines of evidence for/against polyploidy, and their (dis)agreement with each other should be sufficient once the above samples are addressed.<br><br>Response:<br>We thank reviewer 1 for their constructive feedback which we addressed as follows:<br><br>1. We calculated kcov for each sample and modified “DataS1_Sup.8-Comparison_Coverage_kcov_backmap.pl_GenomeScope2_Smudgeplots”. This file now includes two additional columns: the kcov for each sample and the backmapping coverage (which is also reported in DataS1_Sup.5-Backmap.pl_Results). This makes it easier for the reader to compare the different lines of evidence, i.e. sequencing based coverage (2n), kcov (1n), backmapping coverage (2n), genomescope2 kcov (1n), and coverage used in smudgeplot (1n).<br><br>2. We refer to incongruencies of coverage estimates pointed out by the reviewer (O. albircorne, H. phryganea, H. magnus, L. basale, and M. longulum) in the supplementary (page 48) and main text at lines 303-311.<br><br>3. We re-ran smudgeplot with the manually entered kcov as predicted by Genomescope 2 for O. albircorne, H. phryganea, H. magnus, L. basale, and M. longulum, see lines 303-311 in the main text. The smudgeplots are reported in the supplementary figures S90-93 (H. phyrganeae), S96-98 (H. magnus), S104-S107 (L. basale), S110-S113 (M. longulum ML3), S116-119 (M. longulum ML1), S127-S129 (O. albicorne).<br><br>4. We report these new results in the main text (lines 303-311), and discuss the different lines of evidence for/against polyploidy and their (dis)agreement with each other (332-337).<br><br>5. As requested by the GigaDB Team we ran BUSCO v5 for the completeness analysis, updated the summary statistic shown in Table 1 with the v5 results and will uploaded the BUSCO results files in GigaDB: 3 files for each run; (i)short_summary.*.txt (ii) full_table.tsv (iii)missing_busco_list.tsv. We changed this in the methods accordingly, lines 118, 469-470.
**Additional Information:**	
**Question**	**Response**
Are you submitting this manuscript to a special series or article collection?	No
**Experimental design and statistics**	Yes

Full details of the experimental design and statistical methods used should be given in the Methods section, as detailed in our Minimum Standards Reporting Checklist. Information essential to interpreting the data presented should be made available in the figure legends.<br><br>Have you included all the information requested in your manuscript?	
**Resources**<br><br>A description of all resources used, including antibodies, cell lines, animals and software tools, with enough information to allow them to be uniquely identified, should be included in the Methods section. Authors are strongly encouraged to cite Research Resource Identifiers (RRIDs) for antibodies, model organisms and tools, where possible.<br><br>Have you included the information requested as detailed in our Minimum Standards Reporting Checklist?	Yes
**Availability of data and materials**<br><br>All datasets and code on which the conclusions of the paper rely must be either included in your submission or deposited in publicly available repositories (where available and ethically appropriate), referencing such data using a unique identifier in the references and in the “Availability of Data and Materials” section of your manuscript.<br><br>Have you have met the above requirement as detailed in our Minimum Standards Reporting Checklist?	No
If not, please give reasons for any	Raw data and assemblies are available at NCBI under BioProject: PRJNA558902.

omissions below.<br><br>as follow-up to "**Availability of data and materials**<br><br>All datasets and code on which the conclusions of the paper rely must be either included in your submission or deposited in publicly available repositories (where available and ethically appropriate), referencing such data using a unique identifier in the references and in the “Availability of Data and Materials” section of your manuscript.<br><br>Have you have met the above requirement as detailed in our Minimum Standards Reporting Checklist?<br><br>"	Data supporting the results will be submitted to GigaDB and can be accessed under this link: https://app.box.com/folder/136985262731?v=trich-genomes

Powered by Editorial Manager® and ProduXion Manager® from Aries Systems Corporation

Manuscript Click here to access/download;Manuscript;Maintext-editor R1_JH_2.docx

Genome size evolution in the diverse insect order Trichoptera

Authors

Jacqueline Heckenhauer (jacqueline.heckenhauer@senckenberg.de)[1,2*], Paul B. Frandsen (paul_frandsen@byu.edu)[1,3,4], John S. Sproul (johnssproul@gmail.com)[5,6], Zheng Li (zheng.li@austin.utexas.edu)[7], Juraj Paule (juraj.paule@senckenberg.de)[8], Amanda M. Larracuente (alarracu@ur.rochester.edu)[5], Peter J. Maughan (jeff_maughan@byu.edu)[3], Michael S. Barker (msbarker@arizona.edu)[7], Julio V. Schneider (julio.schneider@senckenberg.de)[2], Russell J. Stewart (russell.stewart@utah.edu)[9], Steffen U. Pauls (steffen.pauls@senckenberg.de)[1,2,10]

Affiliations

[*]corresponding author

[1]LOEWE Centre for Translational Biodiversity Genomics (LOEWE-TBG), Frankfurt, Germany,

[2]Department of Terrestrial Zoology, Senckenberg Research Institute and Natural History Museum Frankfurt, Frankfurt, Germany

[3]Department of Plant & Wildlife Sciences, Brigham Young University, Provo, UT

[4]Data Science Lab, Smithsonian Institution, Washington, DC

[5]Department of Biology, University of Rochester, Rochester, NY, USA

[6]Department of Biology, University of Nebraska Omaha, Omaha, USA

[7]Department of Ecology and Evolutionary Biology, University of Arizona, Tucson, USA

[8]Department of Botany and Molecular Evolution, Senckenberg Research Institute and Natural History Museum Frankfurt, Frankfurt, Germany

[9]Department of Biomedical Engineering, University of Utah, Salt Lake City, UT

[10]Institute for Insect Biotechnology, Justus-Liebig-University, Gießen, Germany

ORCIDs:

Jacqueline Heckenhauer [0000-0001-8771-9154]

Paul B. Frandsen [0000-0002-4801-7579]

John S. Sproul [0000-0002-6747-3537]

Zheng Li [0000-0001-6894-9616]

Juraj Paule [0000-0001-5375-7689]

Amanda M. Larracuente [0000-0001-5944-5686]

Peter J. Maughan [0000-0003-3714-3411]

Michael S. Barker [0000-0001-7173-1319]

Julio V. Schneider [0000-0002-9823-6569]

Russell J. Stewart [0000-0002-8389-8877]

Steffen U. Pauls [0000-0002-6451-3425]

Abstract

Background: Genome size is implicated in form, function, and ecological success of a species. Two principally different mechanisms are proposed as major drivers of eukaryotic genome evolution and diversity: Polyploidy (i.e., whole genome duplication: WGD) or smaller duplication events and bursts in the activity of repetitive elements (RE). Here, we generated *de novo* genome assemblies of 17 caddisflies covering all major lineages of Trichoptera. Using these and previously sequenced genomes, we use caddisflies as a model for understanding genome size evolution in diverse insect lineages.

Results: We detect a ~14-fold variation in genome size across the order Trichoptera. We find strong evidence that repetitive element (RE) expansions, particularly those of transposable elements (TEs), are important drivers of large caddisfly genome sizes. Using an innovative method to examine TEs associated with universal single copy orthologs (i.e., BUSCO genes), we find that TE expansions have a major impact on protein-coding gene regions, with TE-

gene associations showing a linear relationship with increasing genome size. Intriguingly, we find that expanded genomes preferentially evolved in caddisfly clades with a higher ecological diversity (i.e., various feeding modes, diversification in variable, less stable environments).

Conclusion: Our findings provide a platform to test hypotheses about the potential evolutionary roles of TE activity and TE-gene associations, particularly in groups with high species, ecological, and functional diversities.

Key words:

biodiversity, *de novo* genome assembly, genomics, genomic diversity, genome duplication, genome size evolution, insects, repetitive elements, transposable elements, Trichoptera

Background

Genome size is a fundamental biological character. Studying its evolution may potentially lead to a better understanding of the origin and underlying processes of the myriad forms and functions of plants and animals. These diversification processes remain at the core of much biological research. Given their high species, ecological and functional diversities, insects are excellent models for such research. To date 1,345 insect genome size estimates have been published [1]) ranging 240-fold from 69 Mbp in chironomid midges [2] to 16.5 Gbp in the mountain grasshopper *Podisma pedestris* [3]. Genome size variation relates poorly to the number of coding genes or the complexity of the organism (C-value enigma, [4],[5],[6],[7]) and evolutionary drivers of genome size variation remain a topic of ongoing debate (e.g. [8], [9], [10], [11]). Two principally different mechanisms are proposed as primary drivers of eukaryotic genome size evolution: Whole genome duplication (WGD, i.e., polyploidy) or

smaller duplication events and expansion of repetitive elements (REs, [6]). While WGD is ubiquitous in plant evolution, it has been regarded as the exception in animals [12], [13]. However, ancient WGD has been hypothesized to be an important driver of evolution of mollusks (e.g. [14]) amphibians (e.g. [15], [16], fish (e.g. [17], [18], [19]) and arthropods (e.g. [20], [21], [22]), including multiple putative ancient large-scale gene duplications within Trichoptera [23].

RE expansion is an important driver of genome size variation in many eukaryotic genomes [24], [25]. The two major categories of REs are tandem repeats (e.g., satellite DNA) and mobile transposable elements (TEs). TEs are classified into class I [retrotransposons: endogenous retroviruses (ERVs), related long terminal repeat (LTR) and non-LTR retrotransposons: SINEs (Short Interspersed Nuclear Elements), LINEs (Long Interspersed Nuclear Elements)] and class II elements (DNA transposons, [26]). In insects, the known genomic proportion of TEs ranges from 1% in the antarctic midge *Belgica antarctica* [27] to 65% in the migratory locust *Locusta migratoria* [28]. Broad-scale analysis of TE abundance in insects suggests that some order-specific signatures are present, however, major shifts in TE abundance are also common at shallow taxonomic levels [29], [30], including in Trichoptera [31]. The movement and proliferation of REs can have deleterious consequences on gene function and genome stability [32], [33], [34], [35], [36]. Moreover, repeat content and abundance can turn over rapidly even over short evolutionary time scales (reviewed in [37]). This rapid evolution has consequences for genome evolution and speciation, e.g., repeat divergence causes genetic incompatibilities between even closely related species [38]. However, TEs can also be sources of genomic innovation with selective advantages for the host [39], [40], [41], [42], [43], [44] and they can contribute to global changes in gene regulatory networks [45], [46], [47]. Investigating RE dynamics in diverse clades provides a powerful lens for understanding their roles in genome function and evolution. Broadly studying of RE dynamics in species-rich groups with wide variation in RE activity is an

important step towards efficiently identifying study systems at finer taxonomical scales (natural populations, species complexes, or recently diverged species) that are ideally suited to advance our understanding of molecular and evolutionary mechanisms underlying genome evolution. In addition, by taking this biodiversity genomics approach, we can develop new model systems and eventually better understand links between environmental factors, genome size evolution, adaptation, and speciation (see [48]).

With more than 16,500 species, caddisflies (Trichoptera) are among the most diverse of all aquatic insects [49]. Their species richness is reflective of their ecological diversity, including, e.g. microhabitat specialization, a full array of feeding modes, and diverse use of underwater silk secretions [50], [51]. An initial comparison of six caddisfly species found wide genome size variation in Trichoptera (ranging from 230 Mbp to 1.4 Gbp). In that study, we hypothesized that the observed variation was correlated with caddisfly phylogeny and that TEs contributed to a suborder-specific increase of genome size [31].

Here, we present a multi-faceted analysis to investigate genome size evolution in the order Trichoptera, as an example for highly diversified non-model organisms. Specifically, we (i) estimated genome size for species across the order to explore phylogenetic patterns in the distribution of genome size variation in Trichoptera and (ii) generated 17 new Trichoptera genomes to analyze, in conjunction with 9 existing genomes, the causes (WGD, TE expansions) of genome size variation in the evolution of caddisflies. Studying the genomic diversity of this highly diversified insect order adds new insights into drivers of genome size evolution with potential to shed light on how genome size is linked to form, function, and ecology.

Data Description

Genomic resources

Here, we combined long- and short-read sequencing technologies to generate 17 new *de novo* genome assemblies across a wide taxonomic range, covering all major lineages of Trichoptera. Details on sequencing coverage and assembly strategies are given in DataS1_Sup.2, DataS1_Sup.3, and supplementary note 3. To assess quality, we calculated assembly statistics with QUAST v5.0.2 [52], examined gene completeness with BUSCO v5 [53], [54] and screened for potential contamination with taxon-annotated GC-coverage (TAGC) plots using BlobTools v1.0 ([94], supplementary Figs. S31-S47). The new genomes are of comparable or better quality than other Trichoptera genomes previously reported in terms of BUSCO completeness and contiguity (Table 1). This study increases the number of assemblies in this order from nine to 26, nearly tripling the number of available caddisfly genomes and thus providing a valuable resource for studying genomic diversity across this ecologically diverse insect order. The annotation of these genomes predicted 6,413 to 12,927 proteins (DataS1_Sup.2). Most of the annotated proteins (94.4% - 98.8%) showed significant sequence similarity to entries in the NCBI nr database. GO Distributions were similar to previously annotated caddisfly genomes, i.e. the major biological processes were cellular and metabolic processes. Catalytic activity was the largest subcategory in molecular function and the cell membrane subcategories were the largest cellular component (supplementary Figs. S1-S30). This project has been deposited at NCBI under BioProject ID: PRJNA558902. For accession numbers of individual assemblies see Table 1.

We downloaded existing Trichoptera genomes from GenBank [56]or Lepbase [57] and used these in conjunction with our newly generated genomes to analyze genome size evolution as explained in the following sections of this manuscript.

Flow cytometry

In addition to genomic sequence data, we used flow cytometry to detect genome size variation across the order. Our study increased the number of species with available flow cytometry-

based genome size estimates from 4 [58] to 31. Estimates were submitted to the Animal Genome Size Database [1] .

Analysis

Genome size evolution in Trichoptera

Based on the genomes of six trichopteran species, Olsen et al. [31] found a 3-fold suborder-specific increase of genome size and hypothesized that genome size variation is correlated with their phylogeny. To test this hypothesis, we first reconstructed phylogenetic relationships by analyzing ~2,000 single-copy BUSCO genes from the 26 study species (Figs. 1 & 2, Fig. S48). We obtained a molecular phylogeny that was in agreement with recent phylogenetic hypotheses ([59], see supplementary note 6) and which showed that Trichoptera is divided into two suborders: Annulipalpia (Figs. 1 & 2: Clade A, blue) and Integripalpia [consisting of basal Integripalpia (Fig. 1: Clade B1-3, light green) and infraorder Phryganides (Fig. 1: clade B4, dark green)]. Trichopterans use silk to build diverse underwater structures (see illustrations Fig. 1; supplementary note 6, supplementary Fig. S48). Thus, we refer to Annulipalpia as 'fixed retreat- and net-spinners', to Phryganides (Integripalpia) as 'tube case-builders', and to basal Integripalpia as 'cocoon-builders'.

We used three approaches for estimating genome size across Trichoptera: *k-mer* distribution-estimates, backmapping of sequence data to available draft genomes (as described in [60]), and flow cytometry (FCM, supplementary note 7, Figs. S49-S72, DataS1_Sup.7). FCM estimates can be affected by chromatin condensation, the proportion of cells in G0 to G1 phases [61], [62] and endoreplication in insect cells and tissues [63]. Sequence-based estimates can be affected by repetitive elements in the genome resulting in smaller genome size estimates (e.g. [64], [58], [65]), as well as by GC-content because sequence library preparation including PCR amplification steps are associated with underrepresentation of GC and AT rich regions [66]. Bland-Altman plots (supplementary note 8, Fig. S73) revealed

general agreement of all three methods in our study. However, the FCM estimates were generally higher compared to the sequence-based estimates (Fig. 1, DataS1_Sup.7) and, among all three approaches, this measure is expected to be the most accurate [9]. We observe that variation among the methods increased with genome size, indicating issues potentially caused by repeat content (see Results *Repeat dynamics*).

We observed large variation in genome size across the order. Genome size tends to be lower in 'fixed retreat- and net-spinners' and 'cocoon-builders' compared to 'tube case-builders' (Fig. 1). Specifically, we observe that genome size varies ~14-fold ranging from 1C = 154 Mbp in 'cocoon-builders' (Fig. 1, B1: Hydroptilidae) to 1C = 2129 Mbp in 'tube case-builders' (Fig. 1, clade B4: Limnephilidae). Of the 29 species analyzed by FCM, *Halesus digitatus* (Fig. 1, clade B4: Limnephilidae, Intergripalpia) possessed the largest genome (1C = 2129 Mbp), while the genome of *Hydropsyche saxonica* (Fig. 1, clade A: Hydropsychidae, 'fixed retreat- and net-spinners') was the smallest (1C = 242 Mbp). Genome size estimates based on sequence-based methods (*kmer*-based and back-mapping) range from 1C = 154 - 160 Mbp in *Agraylea sexmaculata* (Fig. 1, clade B1: Hydroptilidae, 'cocoon-builders') to 1C = 1238 - 1400 Mbp in *Sericostoma* sp. (Fig. 1, clade B4: Sericostomatidae, 'tube case-builders').

Repeat Dynamics

Repetitive element abundance and classification

To understand the structural basis of genome size variation across the order Trichoptera we explored repetitive element (RE) content. We found that major expansions of transposable elements (TEs) contribute to larger genomes in 'tube case-' and some 'cocoon-builders', but particularly in 'tube case-builders' with an average of ~600 Mbp of REs compared to ~138 Mbp in 'fixed retreat- and net-spinners' (Fig. 2 A, B). LINEs are the most abundant classified TEs in 'cocoon-' and 'tube case-builders' and comprise >154 Mb on average in 'tube case-

builders', or an average genome proportion of 16.9% (range = 5.6–34.7%). This represents a 1.8- and 2.8-fold increase in genome proportion relative to 'cocoon-builders' and 'fixed retreat- and net-spinners', respectively. The LINE abundance of >312 Mbp in *Odontocerum albicorne* exceeds the entire assembly lengths (152–282 Mbp) of the three smallest genome assemblies (*Hydropsyche tenuis*, *Parapsyche elsis*, and *Agraylea sexmaculata*) (Fig. 2 A, B). DNA transposons also comprise large genomic fractions in both 'cocoon-' and 'tube case-builders' (averages of 54.4 Mbp and 32.8 Mbp, respectively). However, despite containing a large number of bps, they make up a smaller fraction of total bps in the genomes of 'cocoon-' and 'tube case-builders' than in 'fixed retreat- and net-spinners' (average genome proportion = 5.9%, 4.5%, and 11.1% in 'tube case-builders', 'cocoon-builders', and 'fixed retreat- and net-spinners', respectively) (Fig. 2 B), and thus cannot, by themselves, explain the larger genome sizes. SINEs, LTRs, *Penelope* (grouped with "other" repeats in Fig. 2), and satDNAs show a disproportionate increase in 'cocoon-' and 'tube case-builders', however, all categories combined make up a relatively small proportion of their genomes (all less than 3% on average in Integripalpia) (Fig. 2, B). Unclassified repeats are the most abundant repeat category across all Trichoptera, and they also show disproportionate expansions in both 'cocoon-' and 'case-builders' relative to 'fixed retreat- and net-spinners' (Fig. 2 A, B). The general trends noted in our assembly-based analysis of REs were corroborated by our reference-free analysis of repeat abundance (Figs. S146, S147 supplementary note 10).

TE age distribution analysis

To test whether the observed abundance patterns of specific TEs are driven by shared ancient proliferation events or more recent/ongoing activity of the respective TEs, we analyzed TE age distribution plots. These plots allow us to visualize specific RE classes/superfamilies that account for shifts in RE composition and abundance and infer the relative timing of those shifts based on the distribution of sequence divergence within each RE category. TE age

distributions showed a high abundance of recently diverged TE sequences in ‘cocoon-’ and ‘tube case-builders’, particularly in LINEs, DNA transposons, and LTRs in which the majority of TEs for a given class show 0–10% sequence divergence within copies of a given repeat (Fig. 3). This trend was particularly pronounced among ‘tube case-builders’ with several species showing high abundance of LINEs and DNA transposons with 0–5% sequence divergence (Fig. 3). This pattern suggests that the observed TE expansion is due primarily to ongoing TE activity within lineages rather than a few shared bursts of activity in ancestral lineages. This is further supported by our analysis of repeat sub-classes with age distribution plots (Fig. S148). For example, in our study, LINE abundance is often due to the expansion of different LINE subclasses even between species in the same sub-clade (e.g., compare *Lepidostoma* with *Micrasema*, *Himalopsyche* with *Glossosoma*; Fig. S148). We also find evidence of shared ancient bursts of SINE activity in ‘cocoon-’ and ‘tube case-builders’, although SINEs are not an abundant repeat class in any species (avg. genomic proportion=1.9% stdev=1.7%) (Fig. S148).

Associations between TE sequences and protein-coding genes

During early exploration of our sequence data, we made an unexpected discovery that in some lineages, universal single copy orthologs or “BUSCO genes”, showed higher than expected coverage depth of mapped reads in one or more of their sequence fragments. Further analysis showed that these high coverage BUSCO sequence regions are typically RE sequences (primarily TEs) that are either embedded within or located immediately adjacent to BUSCO genes, such that the BUSCO algorithm includes them in its annotation of a given gene. We refer to BUSCO genes containing these putative RE fragments as ‘TE-associated BUSCOs’ (supplementary Fig. S149, supplementary note 11). By estimating how many times they occur, we can quantitatively measure how TE-gene interactions change with changing genome size. In fact, we detected a positive linear relationship between TE-gene interactions

and increasing genome size when measured with this accidently discovered metric. We found major expansions of TE-associated BUSCOs in ‘cocoon-’ and ‘tube case-builders’ (Fig. 4A) that are significantly correlated with total repeat abundance, as well as the genomic proportion of LINEs and DNA transposons (supplementary Fig. S150). TE-associated BUSCOs comprise a relatively large fraction of total BUSCO genes in these lineages (averages of 11.2% and 21.4% of total BUSCOs in ‘cocoon-‘ and ‘tube case-builders’, respectively), compared to annulipalpian lineages (avg = 6.2%). This finding highlights the major impact of REs on the composition of protein-coding genes in species with repeat-rich genomes. The BUSCO-associated sequences may represent TEs recently inserted into BUSCO genes, the remnants left behind following historical TE transposition events, or TE sequences that are immediately adjacent to and inadvertently classified as BUSCO sequences.

To confirm that unexpectedly high-coverage sequence regions in TE-associated BUSCOs were in fact TE-derived sequences, we compared patterns of BUSCO gene structure (though pairwise alignment) across species pairs in which high-coverage regions (i.e., putative TE sequences) were present in the BUSCO gene of one species (i.e., the “inflated” species), but absent in the homologous BUSCO of the other (i.e., the “reference” species). This analysis showed that in 73 of 75 randomly sampled alignments, reference species showed gaps or highly non-contiguous alignments in high-coverage regions of the inflated species (Fig. 4B), suggesting that sequence insertions are typically present in high-coverage sequence regions of TE-associated BUSCOs. Our subsequent BLAST analysis showed that comparing a TE-associated BUSCO against its own assembly produced thousands-millions of BLAST hits from many contigs (Fig. 4C). This confirmed that the indel sequence present in high-coverage regions of “inflated” species show high sequence similarity to repetitive elements elsewhere in the genome. We then used an intersect analysis on the BLAST results to confirm that the large majority of the excessive BLAST hits overlap with RE annotations throughout the genome, most of which are TEs with LINEs and DNA transposons being most abundant (Fig.

4D, DataS2_Sup.5). Finally, we found that if we replaced the TE-associated BLAST query sequence with the homologous, but non-TE associated BUSCO from its counterpart reference species, the number of BLAST hits was fewer (Fig. 4C, DataS2_Sup.6), offering further evidence that the TE sequence insertions driving the pattern of high-coverage in read mapping excessive BLAST hits are absent in reference species and thus carriable across relatively short time scales within Trichoptera. Taken together, these findings provide strong evidence that TE sequences (especially LINEs and DNA transposons) inadvertently annotated by BUSCO can account for the high-coverage regions we observe in BUSCO genes (Fig. 4D).

Our accidental discovery that quantifying the frequency of TE-associated BUSCOs can serve as an estimate of TE-gene associations may prove useful in other systems given the wide use of BUSCO analysis in genomic studies. Finer details supporting the TE-gene association analysis are reported in supplementary note 11.

Gene and genome duplications

Recently, a transcriptome-based study found evidence for putative ancient gene and genome duplications in hexapods, including potential WGD events in caddisflies [23], suggesting that duplication events could be responsible for some genome size variation in Trichoptera. We investigated whether this pattern persists with whole genome data and found that the age distribution of duplications in 18 genomes were significantly different compared to the background rate of gene duplication (Figs. S161 & S162). To identify if any significant peak is consistent with a potential WGD, we used mixture modeling to identify peaks in these gene age distributions, which recovered no obvious peak consistent with an ancient WGD. To further investigate potential WGD, we used Smudgeplot [67] to visualize the haplotype structure and to estimate ploidy of the genomes.

While Smudgeplot predicted most of the genomes to be diploid, four genomes with rather small genome sizes (230 Mb – 650 Mbp) were predicted to be tetraploid (*Hydropsyche tenuis*, *Rhyacophila evoluta* RSS1 and HR1, *Parapsyche elsis*). However, the Genomescope 2 results indicate that these are highly homozygous samples. Low heterozygosity is a known confounder of smudgeplot analyses [67] because it inflates the signal of duplication when compared to the low level of heterozygosity. We therefore interpret these four putative polyploids as artifacts of low heterozygosity in the analysis. Moreover, in some cases smudgeplot results remain unclear because the estimated coverage (1n) differs from the sequencing coverage, peak coverage from the backmap.pl approach, and Genomescope2 coverage *kcov* (DataS1_Sup.8) when automatically estimated from the data. The adjustment of the expected haploid coverage based on Genomescope2 *kcov* when running smudgeplot suggests that some species might not be diploid (*Hesperophylax magnus*: octoploid, Figs. S96-S98; *Micrasema longulum* ML1: tripolid, Figs. S116-S118; and *Odontocerum albicorne*: tetraploid, Figs. S127-S129. However, further sequencing as well as karyotyping including chromosome counting would need to be done to confirm polyploidy in these species.

Discussion

The drivers and evolutionary consequences of genome size evolution are a topic of ongoing debate. Several models have been proposed [9]. Some hypothesize genome size to be a (mal)adaptive trait by impacting phenotypic traits such as developmental/life history, body size and other cell-size related effects [68], [69], [70], [71] reviewed in [9]. On the other hand, neutral theories suggest that DNA accumulation occurs only by genetic drift without selective pressures playing a major role in the accumulation or loss of DNA [the mutational hazard hypothesis (MHH, [24]) and the mutational equilibrium hypothesis (MEH, [25])]. The MHH only allows for small deleterious effects for the accumulation of extra DNA which is accompanied by higher mutation rates in larger genomes [24], while the MEH focuses on the

balance between insertions and deletions. It suggests that genome expansions arise by 'bursts' of duplication events or TE activity and genome shrinkage may be caused by a more constant rate of small deletions [25].

In this study, we observe that genome size varies ~14-fold across the order Trichoptera, with lower genome size estimates in 'fixed retreat- and net-spinners' and 'cocoon-builders' compared to 'tube case-builders' and explore potential drivers of genome size evolution. Although, recent genomic studies have shown evidence of bursts of gene duplication and gene family expansion during the evolution of hexapods [23], [72] the presence of ancient genome duplication events are still a subject of debate [73], [74], [75]. When computing haplotype structure and ploidy estimation, Smudgeplot suggested polyploidy in three species. However, karyotypes including chromosome counts are missing for these species, as only very few have been reported for caddisflies in general [76], [77], [78]. We found no evidence, of ancient WGD in the gene age distribution in our Trichoptera genomes although we recognize that some of our current genome assemblies might be too fragmented to infer synteny. This does not mean that we can rule out that duplication events played a role in genome size evolution in Trichoptera in the past. The emergence of PacBio HiFi genomes of caddisflies (e.g., Darwin Tree of Life Project is currently planning to sequence several caddisfly genomes [79] will allow a deeper exploration of putative ancient duplication events in Trichoptera.

We found evidence that TE expansions (especially LINEs) were important drivers of genome size evolution in Trichoptera (Fig. 2, Figs. S146 & S147), which is consistent with the mutational equilibrium hypothesis (MEH). The TE age distribution analyses suggested that the high abundance of LINEs was due to ongoing/recent activity occurring independently across 'cocoon-' and particularly 'tube case-builders' (Fig. 3, Fig. S148). Thus, the shift to large genomes in these lineages does not appear to be due to a single (or few) shared ancient events, rather they maintained dynamic turnover in composition of their large genomes. Mutational bias affecting pathways tied to TE-regulation may affect insertion/deletion ratios

and subsequently lead to lineage-specific shifts in genome size equilibrium [80]. Such changes may be stochastic (e.g., due to drift), or linked to traits that evolve on independent trajectories as lineages diverge and are thereby constrained by phylogeny. Ecological factors, demographic history, and effective population size can further impact mutation rates. For example, environmental stress can trigger bursts of TE activity and elevated mutation rates [81], [82], [83] driving lineages that occupy niche space with frequent exposure to environmental stress toward increased TE loads and larger genomes. Similarly, lineages with small effective population sizes or which are prone to population bottlenecks may have higher mutation rates and/or reduced efficacy of natural selection which would otherwise purge mildly deleterious TE load.

Although our study is not designed to pinpoint specific forces maintaining large genomes in some lineages, the pattern we observe in the distribution of genome size (i.e. lower genome size estimates in 'fixed retreat- and net-spinners' and 'cocoon-builders' compared to 'tube case-builders') leads us to hypothesize that ecological factors may play a role in genome size evolution in the order. The three focal groups discussed here exhibit markedly different ecological strategies. Larvae of 'fixed retreat- and net-spinners' generally occupy relatively narrow niche space in oxygen-rich flowing-water (mostly stream/river) environments where they rely on water currents to bring food materials to their filter nets. The evolutionary innovation of tube-case making is thought to have enabled 'tube case-builders' to occupy a much greater diversity of ecological niche space by allowing them to obtain oxygen in lentic (e.g., pond, lake, marsh) environments which are much more variable in temperature and oxygen availability than lotic environments [84], [85]. This environmental instability is greater over short (daily, seasonal) and long-time scales (centuries, millennia) [86]. It is thus plausible these tube case-building lineages experience greater environmental stress and less stable population demographics that could lead to both more frequent TE bursts and reduced

efficacy of natural selection in purging deleterious effects of TE expansions as described above [24], [25].

We show that TE expansions (especially LINEs and DNA transposons) in ‘cocoon-’ and ‘tube case-builders’ have a major impact on protein-coding gene regions (Fig. 4). These TE-gene associations show a linear relationship with increasing genome size. This trend is particularly pronounced among ‘tube case-builders’ in which TE-associated BUSCOs comprise an average of 21.4% of total BUSCO genes (compared to 6.2% in annulipalpians). This finding corroborates other studies highlighting the role of TEs as drivers of rapid genome evolution [87], [88], [89], [90] and highlights their impact on genomic regions that have potential effects on phenotypes. Questions remain as to what evolutionary roles such changes in genic regions may play. In general, TE insertions are considered to have deleterious effects on their host’s fitness activity [91], [92]. They are known to “interrupt” genes [34], pose a risk of ectopic recombination that can lead to genome rearrangements [35], [32], [93], and have epigenetic effects on neighboring sequences [94], [95]. Therefore, purifying selection keeps TEs at low frequencies [34]. However, there is growing evidence that TE activity can also be a critical source of new genetic variation driving diversification via chromosomal rearrangements and transposition events which can result in mutations [96], including examples, of co-option [97], e.g. recent research in mammals has shown that DNA transposon fragments can be co-opted to form regulatory networks with genome-wide effects on gene expression [45].

Ecological correlates with genome size are widely discussed in other taxa [98], [99], [100], [101], [102]. Caddisflies and other diverse insect lineages that feature various microhabitat specializations, feeding modes, and/or the use of silk represent evolutionary replicates with contrasting traits and dynamic genome size evolution. They thus have high potential as models for understanding links between ecology and the evolution of REs, genomes, and phenotypes. Our study lays a foundation for future work in caddisflies that investigates the

potential impact of TE expansions on phenotypes and tests for evidence of co-option/adaptive impacts of TE-rich genomes against a null of neutral or slightly deleterious effects.

Potential implications

Many open questions remain as to the causes and consequences of genome size evolution. As we move forward in an era where genome assemblies are attainable for historically intractable organisms (e.g. due to constraints given large genome sizes, tissue limitations, no close reference available) we can leverage new model systems spanning a greater diversity of life to understand how genomes evolve. Here, we provide genomic resources and new genome size estimates across lineages of an underrepresented insect order that spans major variation in genome size. These data allowed us to study genome size evolution in a phylogenetic framework to reveal lineage-specific patterns in which genome size correlates strongly with phylogeny and ecological characteristics within lineages. We find that large genomes dominate lineages with a wider range of ecological variation, and that ongoing recent TE activity appears to maintain large genomes in these lineages. This leads us to hypothesize that ecological factors may be linked to genome size evolution in this group. The future directions spawned by our findings highlight the potential for using Trichoptera and other diverse insect groups to understand the link between ecological and genomic diversity, a link that has been challenging to study with past models [9].

We also show that TE expansions are associated with increasing genome size and have an impact on protein-coding regions. These impacts have been greatest in the most species-rich and ecologically diverse caddisfly clades. While TEs are generally considered to have deleterious effects on their host's fitness activity, their roles can also be neutral or even adaptive. TE activity can be a critical source of new genetic variation and thus an important driver for diversification. Caddisflies and potentially other non-model insect groups are excellent models to test these contrasting hypotheses, as well as the potential impact of TEs

on phenotypes. Using these models, especially with respect to the increasing emergence of high-quality insect genomes [103], will allow researchers to identify recurring patterns in TE dynamics and investigate their evolutionary implications across diverse clades.

Methods

DNA extraction, library preparation, sequencing, and sequence read processing

We extracted high molecular weight genomic DNA (gDNA) from 17 individuals (15 species) of caddisfly larvae (for sampling information, see DataS1_Sup.1) after removing the intestinal tracts using a salting-out protocol adapted from [104] as described in supplementary note 1. We generated gDNA libraries for a low-cost high-contiguity sequencing strategy, i.e. employing a combination of short (Illumina) and long read (Nanopore or PacBio) technologies as described in supplementary notes 2. For details on sequencing coverage for each specimen see DataS1_Sup.3.

De novo genome assembly, annotation and quality assessment

We applied different assembly strategies for different datasets. First, we applied a long-read assembly method using wtdbg2 v2.4 (WTDBG, RRID:SCR_017225) [105] with subsequent short-read polishing with Pilon v1.22 (Pilon, RRID:SCR_014731) [106] as this method revealed good results in previous *de novo* assemblies in caddisflies [58]. In cases where this pipeline did not meet the expected quality regarding contiguity and BUSCO completeness, we applied *de novo* hybrid assembly approaches of MaSuRCA v.3.1.1 (MaSuRCA, RRID:SCR_010691) [107] (supplementary note 3). Illumina-only data was assembled with SPAdes (SPAdes, RRID:SCR_000131) [108] explained in supplementary note 3. Prior to annotating the individual genomes with MAKER2 v2.31.10 [109], [110] we used RepeatModeler v2.0 (RepeatModeler, RRID:SCR_015027) and RepeatMasker v4.1.0

(RepeatMasker, RRID:SCR_012954), to identify species-specific repetitive elements in each of the assemblies, relative to RepBase libraries v20181026 [111]Transcriptome evidence for the annotation of the individual genomes included their species-specific or closely related *de novo* transcriptome provided by 1KITE [112], [113] DataS1_Sup.9) or downloaded from Genbank as well as the cDNA and protein models from *Stenopsyche tienmushanensis* [114] and *Bombyx mori* (AR102, GenBank accession ID# GCF_000151625.1). Additional protein evidence included the uniprot-sprot database (downloaded September 25, 2018). We masked repeats based on species-specific files produced by RepeatModeler. For *ab initio* gene prediction, species specific AUGUSTUS gene prediction models as well as *Bombyx mori* SNAP gene models were provided to MAKER. The EvidenceModeler (EVidenceModeler, RRID:SCR_014659) [115] and tRNAscan [116] options in MAKER were employed to produce a weighted consensus gene structure and to identify tRNAs genes. MAKER default options were utilized for BLASTN (BLASTN, RRID:SCR_001598), BLASTX (BLASTX, RRID:SCR_001653), TBLASTX (TBLASTX, RRID:SCR_011823) searches. Two assemblies (*Agapetus fuscipens* GL3 and *Micrasema longulum* ML1) were not annotated because of their low contiguity. All protein sequences were assigned putative names by BlastP Protein–Protein BLAST 2.2.30+ searches [117] and were functionally annotated using command line Blast2Go v1.3.3 (Blast2GO, RRID:SCR_005828) [118], see supplementary note 4, Figs. S1-S30).

We calculated assembly statistics with QUAST v5.0.2 (QUAST, RRID:SCR_001228) [52] and examined completeness with BUSCO v5 (BUSCO, RRID:SCR_015008) [53], [54] using the Endopterygota odb10 dataset with the options *--long, --m = genome* . A summary of the assembly statistics and BUSCO completeness is given in Table 1. The final genome assemblies and annotations were screened and filtered for potential contaminations with taxon-annotated GC-coverage (TAGC) plots using BlobTools v1.0 (Blobtools,

RRID:SCR_017618) [55]. Details and blobplots are given in supplementary note 5 & supplementary Figs. S31-S47.

Species tree reconstruction

We used the single-copy orthologs resulting from a BUSCOv3.0.2 analyses (with the Endopterygota odb9 dataset and options --long, --m =genome and --sp=fly) to generate a species tree. We first combined single-copy ortholog amino acid files from each species into a single FASTA for each ortholog. We then aligned them with the MAFFT L-INS-i algorithm [119]. We selected amino acid substitution models for each ortholog using ModelFinder (option -m mfp,[120] in IQtree v.2.0.6 [121] and estimated a maximum likelihood tree with 1000 ultrafast bootstrap replicates [122] with the BNNI correction (option -bb 1000 -bnni). We combined the best maximum likelihood tree from each gene for species tree analysis in ASTRAL-III [123]. A locus tree was inferred using the alignment file (-s) and the partition file (-S) with the settings –prefix loci and -T AUTO in IQtree. Gene and site concordance factors were calculated with IQTree using the species tree (-t), the locus tree (--gcf) and the alignment file (-s) with 100 quartets for computing the site concordance factors (--scf 100) and --prefix concord for computing the gene concordance factors. We visualized the trees using FigTree v.1.4.4 (FigTree, RRID:SCR_008515)

Genome size estimations and genome profiling

Genome size estimates of 27 species were conducted using flow cytometry (FCM) according to Otto [124] using *Lycopersicon esculentum* cv. Stupické polnítyčkové rané (2C = 1.96 pg;[125]) as internal standard and propidium iodine as stain. Additionally, we used trimmed, contamination filtered short-read data (see supplementary note 2) to conduct genome profiling (estimation of major genome characteristics such as size, heterozygosity, and repetitiveness) using a *k-mer* distribution-based method (GenomeScope 2.0,

RRID:SCR_017014) [67]. Genome scope profiles are available online (see links to Genomescope 2 in DataS1_Sup.4). In addition, we applied a second sequencing-based method for genome size estimates, which uses the backmapping rate of sequenced reads to the assembly and coverage distribution (backmap.pl v0.1, [60]. Details of all three methods are described in supplementary note 7**.** Coverage distribution per position and genome size estimate from backmap.pl are shown in Figs. S49-72). We assessed the congruence among the three quantitative methods of measurement (Genomescope2, Backmap.pl and FCM) with Bland-Altman-Plots using the function BlandAltmanLeh::bland.altman.plot in ggplot2 [126] in RStudio [127] (supplementary note 8, Fig. S73).

Repeat dynamics

Repeat abundance and classification

We identified and classified repetitive elements in the genome assemblies of each species using RepeatModeler2.0 [128]. We annotated repeats in the contamination filtered assemblies with RepeatMasker 4.1.0 (RepeatMasker, RRID:SCR_012954 using the custom repeat libraries generated from RepeatModeler2 for each respective assembly with the search engine set to "ncbi" and using the -xsmall option. We converted the softmasked assembly resulting from the first RepeatMasker round into a hardmasked assembly using the lc2n.py script [129]Finally, we re-ran RepeatMasker on the hard-masked genome with RepeatMasker's internal arthropod repeat library using -species "Arthropoda". We then merged RepeatMasker output tables from both runs by parsing them with a custom made script (RM_table_parser_families_.py, [142]) and then combined the resulting data columns for the two runs in Excel.

We also estimated repetitive element abundance and composition using RepeatExplorer2[130], [131] and dnaPipeTE v.1.3.1 [132]. These reference-free approaches quantify repeats directly from unassembled short-read data. These analyses allowed us to test

for general consistency of patterns with our assembly-based approach described above, and to test for the presence of abundant repeat categories such as satellite DNAs which can comprise large fractions of genomes yet can be prone to poor representation in the genome assembly. Prior to analysis, we normalized contamination filtered (see supplementary note 2) input data sets to 0.5x coverage using RepeatProfiler [133] and seqtk [134]and then ran RepeatExplorer2 clustering with the Metazoa 3.0 database specified for annotation (supplementary Fig. S146) and dnaPipeTE with the -RM_lib flag set to the Repbase v20170127 repeat library (supplementary Fig. S147).

TE age distribution analysis

We further characterized repetitive element dynamics in Trichoptera by analyzing TE landscapes, which show relative age differences among TE sequences and their genomic abundance. We used these analyses to test whether abundance patterns of specific TEs are driven by shared ancient proliferation events or more recent/ongoing activity of the respective TEs. For example, if shared ancient proliferation is driving abundance patterns of a given TE, the majority of its copies would show moderate to high sequence divergence (e.g., >10% pairwise divergence). In contrast, if abundance patterns are driven by recent/ongoing activity of a given TE, we would expect the majority of its sequences to show low sequence divergence (e.g., 0–10%). We generated TE age distribution plots using dnaPipeTE v1.3.1 [132] with genomic coverage for each species sampled to 0.5X prior to analysis and the -RM_lib flag set to the Repbase v20170127 repeat library (supplementary Fig. S148).

TE sequence associations with protein-coding genes

We analyzed BUSCO genes (obtained from a BUSCOv3.0.2 analyses with the Endopterygota odb9 dataset and options --long, --m =genome and --sp=fly) for all species to quantify the abundance of TE-associated BUSCOs across samples and investigated associations between

TEs and genic sequences in Trichoptera lineages by quantifying the abundance of TE-associated BUSCO genes (for presence and absence of TE-associated BUSCOs see Fig. S149, DataS2_Sup.3). This analysis also allowed us to quantify shifts in associations between TEs and genic regions across Trichoptera lineages with varying repeat abundance. We identified BUSCO genes with high-coverage sequence regions based on coverage profiles and quantified their genomic abundance by using each TE-associated BUSCO as a query in a BLAST search against their respective genome assembly. We then conducted intersect analysis for all unique BUSCO hits from high coverage sequences to determine if these were annotated as TEs. We calculated the total number of bases in filtered BLAST after subtracting the number of bases at the locus belonging to all 'complete' BUSCO genes and categorized high coverage sequence regions in BUSCO genes based on their annotation status and repeat classification using custom scripts [142] We plotted the number of the high coverage BUSCO sequence regions belonging to repetitive element categories (i.e., classes and subclasses) alongside plots of the relative genomic abundance of each respective category. In addition, we investigated BUSCO genes with regions of high coverage by pairwise alignments. Specifically, we visualized alignments of BUSCOs with high coverage sequence regions (i.e., the "inflated species") alongside orthologous BUSCOS that lack such regions taken from closely related species (i.e., the "reference" species). We further tested this prediction by taking the set of BUSCOs that only exhibited high coverage regions in the inflated species and contrasted results of two BLAST searches followed by an intersect analysis. A detailed description of this method is provided in supplementary note 11.

Gene and Genome duplications

Inference of WGDs from gene age distributions

To recover signal from potential WGDs, for each genome, we used the DupPipe pipeline to construct gene families and estimate the age distribution of gene duplications [135], [136].

We translated DNA sequences and identified ORFs by comparing the Genewise [137] alignment to the best- hit protein from a collection of proteins from 24 metazoan genomes from Metazome v3.0. For all DupPipe runs, we used protein- guided DNA alignments to align our nucleic acid sequences while maintaining the ORFs. We estimated synonymous divergence (K_s) using PAML with the F3X4 model [138] for each node in the gene family phylogenies. We first identified taxa with potential WGDs by comparing their paralog ages to a simulated null distribution without ancient WGDs using a K-S goodness-of-fit test [139]. We then used mixture modeling to identify if any significant peaks consistent with a potential WGD and to estimate their median paralog K_s values. Significant peaks were identified using a likelihood ratio test in the boot.comp function of the package mixtools in R [140].

Visualization of genome structure to estimate ploidy using smudgeplots

We visualized the genome structure and estimated ploidy levels with smudgeplot. For this purpose, we extracted genomic kmers from kmer counts produced with jellyfish (as described above in "Genome size estimation and genome profiling") using "jellyfish dump" with coverage thresholds previously estimated from kmer histograms using the smudgeplot.py script. We computed the set of kmer pairs with the Smudgeplot tool hetkmers. After generating the list of kmer pair coverages, we generated smudgeplots using the coverage of the kmer pairs and the "plot" tool within Smudgeplot. Ploidy as well as the haploid kmer coverage was estimated directly from the data and compared to the estimated *kcov* reported by Genomescope2, sequencing coverage (and sequencing-based *kcov*), peak coverage from the backmap.pl approach (see DataS1-Sup.8). When the haploid kmer coverage estimated by smudgeplot was inconsistent with the *kcov* observed by Genomescope2, it was manually adjusted using -n in smudgeplot.py plot. Details of the method and smudgeplots are given in Figs. S74-145.

Figures Legends:

Fig. 1: Ecological diversity (right) and genome size (left) in caddisflies. Phylogenetic relationships derived from ASTRAL-III analyses using single BUSCO genes. Goeridae, which was not included in the BUSCO gene set, was placed according to [59]. ASTRAL support values (local posterior probabilities) higher than 0.9 are given for each node. The placement of Hydroptilidae (clade B1) was ambiguous. Since its placement was poorly supported in our analyses, we placed it according to Thomas et al. [59]. Taxa were collapsed to family level. Trichoptera are divided into two suborders: Annulipalpia ('fixed retreat- and net-spinners', clade A: blue) and Intergripalpia (clade B: green) which includes basal Integripalpia ('cocoon-builders', clades B1-B3, dark green) and Phryganides or 'tube case-builders' (clade B4: light green). 'Cocoon-builders' are divided into 'purse case'- (clade B1), 'tortoise case-building' (clade B2) and 'free-living' (clade B3) families. Genome size estimates based on different methods (Genomescope2: orange, Backmap.pl: black, Flow Cytometry (FCM): brown) are given for various caddisfly families. Each dot corresponds to a mean estimate of a species. For detailed information on the species and number of individuals used in each method see Data S1_Sup.7 -Genome size - Summary. Colors and clade numbers in the phylogenetic tree refer to colored boxes with illustrations. The following species are illustrated by Ralph Holzenthal: a: *Hydropsyche sp.* (Hydropsychidae); b: *Chimarra sp.* (Philopotamidae); c: *Stenopsyche sp.* (Stenopsychidae); d: *Polycentropus* sp. (Polycentropodidae); e: *Agraylea sp.* (Hydroptilidae); f: *Glossosoma sp.* (Glossosomatidae); g: *Rhyacophila sp.* (Rhyacophilidae); h: *Fabria inornata* (Phryganeidae); i: *Micrasema* sp. (Brachycentridae); j:*Goera fuscula* (Goeridae); k: *Sphagnophylax meiops* (Limnephilidae); l: *Psilotreta sp.* (Odontoceridae), m: *Grumicha grumicha* (Sericostomatidae).

Fig. 2: Repeat abundance and classification in 26 caddisfly genomes. Number of bp for each repeat type is given for each caddisfly genome. A: Repeat abundance and classification. Phylogenetic tree was reconstructed with ASTRAL-III using single BUSCO genes from the genome assemblies. The placement of Hydroptilidae (clade B1) was ambiguous. Since its placement was poorly supported in our analyses, we placed the single hydroptilid taxon (*Agraylea sexmaculata*) according to Thomas et al. [59]. Species names corresponding to the abbreviations in the tree can be found in Table 1. Trichoptera are divided into two suborders: Annulipalpia ('fixed retreat- and net-spinners', clade A: blue) and Intergripalpia (clade B: green) which includes basal Integripalpia ('cocoon-builders', clades B1-B3, dark green) and Phryganides or 'tube case-builders' (clade B4: light green). 'Cocoon-builders' are divided into 'purse case'- (clade B1), 'tortoise case-building' (clade B2) and 'free-living' (clade B3) families. An illustration of a representative of each clade is given. The "other_repeats" category includes: rolling-circles, *Penelope*, low-complexity, simple repeats, and small RNAs. B: Boxplots summarizing shifts in the genomic proportion of RE categories in major Trichoptera lineages.

Fig. 3: Transposable element age distribution landscapes. Representative examples are chosen from major Trichoptera lineages. The y-axis shows TE abundance as a proportion of the genome (e.g., 1.0 = 1% of the genome). The x-axis shows sequence divergence relative to TE consensus sequences for major TE classes. TE classes with abundance skewed toward the left (i.e., low sequence divergence) are inferred to have a recent history of diversification relative to TE classes with right-skewed abundance. Plots were generated in dnaPipeTE. Plots for all species are shown in supplementary Fig. S148. For tip labels of the phylogenetic tree see Fig. 2.

Fig. 4: TE-BUSCO-gene associations in Trichoptera species. (A) Raw abundance of TE-associated BUSCO sequences present in the assembly of 2442 BUSCOs in the OrthoDB 9 Endopterygota dataset. (B) Upper plot: An example of a coverage depth profile of a TE-associated BUSCO gene [BUSCO EOG090R02Q9 from ML1 ('inflated species')] which shows unexpected high coverage in the second exon putatively due to the presence of a RE-derived sequence fragment. Lower plot: A typifying alignment between a TE-associated BUSCO and its orthologous BUSCO from a closely related species ('reference species') that lack TE-association. The non-TE-associated orthologous BUSCO shows non-contiguous alignment in regions of inflated coverage in the TE-associated BUSCO, consistent with the presence of a RE-derived sequence fragment in the TE-associated BUSCO that is absent in the reference species. (C) Summary of total bases annotated as REs obtained from each of two BLAST searches. First, when we used BLAST to compare an TE-associated BUSCOs against an assembly for the same species BLAST hits included megabases of annotated repeats (dark plots). Second, when non-TE-associated orthologs of the TE-associated BUSCOs in the first search are taken from a close relative and compared against the inflated species using BLAST, there is a dramatic drop in BLAST hits annotated as REs. Note log scale on the y-axis. (D) Summary of annotations for BLAST hits for classified REs when TE-associated BUSCOs are compared against an assembly of the same species using BLAST,

Data availability

This project has been deposited at NCBI under BioProject ID: PRJNA558902.

The data sets supporting the results of this article are available in the supplementary, data files S1 and S2. All supporting data and materials are available in the *GigaScience* GigaDB database [141]. All custom made scripts used in this study are available at [142]

List of abbreviations

bp: base pair; BLAST: Basic Local Alignment Search Tool; BUSCO: Benchmarking Universal Single-Copy Orthologs; ERV: endogenous Retroviruses; FCM: Flow Cytometry; Gbp: Giga base pair; LINE: Long Interspersed Nuclear Elements; LTR: Related Long Terminal Repeat; Mbp: Mega base pair; MEH: mutational equilibrium hypothesis; MHH: mutational hazard hypothesis; NCBI: National Center for Biotechnology Information; RE: repetitive element; SINE: Short Interspersed Nuclear Elements; SPAdes: St. Petersburg genome Assembler; SRA: Sequence Read Archive; TE: transposable element; WGD: Whole Genome Duplication. **Declarations**

Consent for publication: Not applicable

Competing interests: Authors declare that they have no competing interests.

List of abbreviations

Funding

This work is a result of the LOEWE-Centre for Translational Biodiversity Genomics funded by the Hessen State Ministry of Higher Education, Research and the Arts (HMWK) that supported JH and SUP, as well as internal funds of Senckenberg Research Institute provided to JP. JSS was supported by an NSF Postdoctoral Research Fellowship in Biology (DBI-1811930) and an NIH General Medical Sciences award (R35GM119515) to AML. Sequencing was, in part, supported by BYU start-up funds to PBF and funds from the Army Research Office, Life Science Division (Award no. W911NF-13-1-0319) to RJS.

Author's contributions:

Conceptualization –JH, JSS, PBF, SUP

Data curation – JH

Formal Analysis - JH, JM, JP, JSS, PBF, ZL

Funding acquisition – AML, PBF, SUP, RJS, RJS

Investigation – JH, JP, JSS, PBF, ZL

Methodology – AML, JSS, JP, JVS, PBF

Project administration – SUP

Resources – JP, MB, PBF, SUP

Visualization - JH, JSS

Writing – original draft – JH, JSS, PBF, ZL

Writing – review & editing – AML, JH, JM, JP, JSS, JVS, MB, PBF, RJS, RJS, SUP, ZL

Acknowledgments

The authors thank Ralph Holzenthal for providing illustrations of larval Trichoptera and the structures they build. We thank Bob Wisseman for collecting *Himalopsyche phryganeae.* We thank both reviewers for their insightful critiques and interest in our manuscript.

References

1. Animal Genome Size Database. https://www.genomesize.com/ Accessed 2022 Jan 19.

2. Cornette R, Gusev O, Nakahara Y, Shimura S, Kikawada T, Okuda T. Chironomid midges (Diptera, chironomidae) show extremely small genome sizes. *Zoolog Sci*. 2015; doi: 10.2108/zs140166.

3. Westerman M, Barton NH, Hewitt GM. Differences in DNA content between two chromosomal races of the grasshopper *Podisma pedestris*. *Heredity*. Nature Publishing Group; 1987; doi: 10.1038/hdy.1987.36.

4. Thomas CA. The genetic organization of chromosomes. *Annu Rev Genet*. Annual Reviews; 1971; doi: 10.1146/annurev.ge.05.120171.001321.

5. Bernard John. The Eukaryote Genome in Development and Evolution. Netherlands: Springer Science & Business Media; 1988. https://doi.org/10.1007/978-94-011-5991-3

6. Gregory TR. The C-value Enigma in Plants and Animals: A Review of Parallels and an Appeal for Partnership. *Ann Bot*. 2005; doi: 10.1093/aob/mci009.

7. Elliott TA, Gregory TR. What's in a genome? The C-value enigma and the evolution of eukaryotic genome content. *Philos Trans R Soc B Biol Sci*. Royal Society; 2015; doi: 10.1098/rstb.2014.0331.

8. Abdel-Haleem H. The Origins of Genome Architecture. *J Hered*. 2007; doi: 10.1093/jhered/esm073.

9. Blommaert J. Genome size evolution: towards new model systems for old questions. *Proc R Soc B Biol Sci*. Royal Society; 2020; doi: 10.1098/rspb.2020.1441.

10. Canapa A, Barucca M, Biscotti MA, Forconi M, Olmo E. Transposons, Genome Size, and Evolutionary Insights in Animals. *Cytogenet Genome Res*. 2015; doi: 10.1159/000444429.

11. Petrov DA. Evolution of genome size: new approaches to an old problem. *Trends Genet TIG*. 2001; doi: 10.1016/s0168-9525(00)02157-0.

12. Orr HA. “Why Polyploidy is Rarer in Animals Than in Plants” Revisited. *Am Nat*. The University of Chicago Press; 1990; doi: 10.1086/285130.

13. Otto SP, Whitton J. Polyploid Incidence and Evolution. *Annu Rev Genet*. 2000; doi: 10.1146/annurev.genet.34.1.401.

14. Hallinan NM, Lindberg DR. Comparative analysis of chromosome counts infers three paleopolyploidies in the mollusca. *Genome Biol Evol*. 2011; doi: 10.1093/gbe/evr087.

15. Alexandrou MA, Swartz BA, Matzke NJ, Oakley TH. Genome duplication and multiple evolutionary origins of complex migratory behavior in Salmonidae. *Mol Phylogenet Evol*. 2013; doi: 10.1016/j.ympev.2013.07.026.

16. Campbell MA, Hale MC, McKinney GJ, Nichols KM, Pearse DE. Long-Term Conservation of Ohnologs Through Partial Tetrasomy Following Whole-Genome Duplication in Salmonidae. *G3 GenesGenomesGenetics*. 2019; doi: 10.1534/g3.119.400070.

17. Woods IG, Wilson C, Friedlander B, Chang P, Reyes DK, Nix R, et al.. The zebrafish gene map defines ancestral vertebrate chromosomes. *Genome Res*. 2005; doi: 10.1101/gr.4134305.

18. Berthelot C, Brunet F, Chalopin D, Juanchich A, Bernard M, Noël B, et al.. The rainbow trout genome provides novel insights into evolution after whole-genome duplication in vertebrates. *Nat Commun*. Nature Publishing Group; 2014; doi: 10.1038/ncomms4657.

19. Glasauer SMK, Neuhauss SCF. Whole-genome duplication in teleost fishes and its evolutionary consequences. *Mol Genet Genomics MGG*. 2014; doi: 10.1007/s00438-014-0889-2.

20. Clarke TH, Garb JE, Hayashi CY, Arensburger P, Ayoub NA. Spider Transcriptomes Identify Ancient Large-Scale Gene Duplication Event Potentially Important in Silk Gland Evolution. *Genome Biol Evol*. 2015; doi: 10.1093/gbe/evv110.

21. Kenny NJ, Chan KW, Nong W, Qu Z, Maeso I, Yip HY, et al.. Ancestral whole-genome duplication in the marine chelicerate horseshoe crabs. *Heredity*. Nature Publishing Group; 2016; doi: 10.1038/hdy.2015.89.

22. Schwager EE, Sharma PP, Clarke T, Leite DJ, Wierschin T, Pechmann M, et al.. The house spider genome reveals an ancient whole-genome duplication during arachnid evolution. *BMC Biol*. 2017; doi: 10.1186/s12915-017-0399-x.

23. Li Z, Tiley GP, Galuska SR, Reardon CR, Kidder TI, Rundell RJ, et al.. Multiple large-scale gene and genome duplications during the evolution of hexapods. *Proc Natl Acad Sci*. National Academy of Sciences; 2018; doi: 10.1073/pnas.1710791115.

24. Lynch M, Conery JS. The Origins of Genome Complexity. *Science*. American Association for the Advancement of Science; 2003; doi: 10.1126/science.1089370.

25. Petrov DA. Mutational Equilibrium Model of Genome Size Evolution. *Theor Popul Biol*. 2002; doi: 10.1006/tpbi.2002.1605.

26. Wicker T, Sabot F, Hua-Van A, Bennetzen JL, Capy P, Chalhoub B, et al.. A unified classification system for eukaryotic transposable elements. *Nat Rev Genet*. Nature Publishing Group; 2007; doi: 10.1038/nrg2165.

795 27. Kelley JL, Peyton JT, Fiston-Lavier A-S, Teets NM, Yee M-C, Johnston JS, et al.. Compact genome of
796 the Antarctic midge is likely an adaptation to an extreme environment. *Nat Commun*. Nature
797 Publishing Group; 2014; doi: 10.1038/ncomms5611.

798 28. Wang X, Fang X, Yang P, Jiang X, Jiang F, Zhao D, et al.. The locust genome provides insight into
799 swarm formation and long-distance flight. *Nat Commun*. Nature Publishing Group; 2014; doi:
800 10.1038/ncomms3957.

801 29. Petersen M, Armisén D, Gibbs RA, Hering L, Khila A, Mayer G, et al.. Diversity and evolution of the
802 transposable element repertoire in arthropods with particular reference to insects. *BMC Ecol Evol*.
803 2019; doi: 10.1186/s12862-018-1324-9.

804 30. Gilbert C, Peccoud J, Cordaux R. Transposable Elements and the Evolution of Insects. *Annu Rev*
805 *Entomol*. Annual Reviews; 2021; doi: 10.1146/annurev-ento-070720-074650.

806 31. Olsen LK, Heckenhauer J, Sproul JS, Dikow RB, Gonzalesz VL, Kweskin MP, et al.. De novo whole
807 genome assemblies of Agrypnia vestita Walker, and Hesperophlax magnus Banks reveal substantial
808 repetitive element expansion in tube case-making caddisflies (Insecta: Trichoptera). *Genome Biol*
809 *Evol*. 2021; doi: 10.1093/gbe/evab013.

810 32. Montgomery E, Charlesworth B, Langley CH. A test for the role of natural selection in the
811 stabilization of transposable element copy number in a population of Drosophila melanogaster.
812 *Genet Res*. Cambridge University Press; 1987; doi: 10.1017/S0016672300026707.

813 33. Charlesworth B, Langley CH. Transposition of copia elements in Drosophila. *Nature*. Nature
814 Publishing Group; 1988; doi: 10.1038/332021b0.

815 34. Charlesworth B, Langley CH. The Population Genetics of Drosophila Transposable Elements. *Annu*
816 *Rev Genet*. 1989; doi: 10.1146/annurev.ge.23.120189.001343.

817 35. Montgomery EA, Huang SM, Langley CH, Judd BH. Chromosome rearrangement by ectopic
818 recombination in Drosophila melanogaster: genome structure and evolution. *Genetics*. 1991; doi:
819 10.1093/genetics/129.4.1085.

820 36. Mieczkowski PA, Lemoine FJ, Petes TD. Recombination between retrotransposons as a source of
821 chromosome rearrangements in the yeast Saccharomyces cerevisiae. *DNA Repair*. 2006; doi:
822 10.1016/j.dnarep.2006.05.027.

823 37. Ugarković Đ, Plohl M. Variation in satellite DNA profiles—causes and effects. *EMBO J*. John Wiley
824 & Sons, Ltd; 2002; doi: 10.1093/emboj/cdf612.

825 38. Ferree PM, Barbash DA. Species-Specific Heterochromatin Prevents Mitotic Chromosome
826 Segregation to Cause Hybrid Lethality in Drosophila. *PLOS Biol*. Public Library of Science; 2009; doi:
827 10.1371/journal.pbio.1000234.

828 39. Gahan LJ, Gould F, Heckel DG. Identification of a Gene Associated with Bt Resistance in Heliothis
829 virescens. *Science*. American Association for the Advancement of Science; 2001; doi:
830 10.1126/science.1060949.

831 40. Chen S, Li X. Transposable elements are enriched within or in close proximity to xenobiotic-
832 metabolizing cytochrome P450 genes. *BMC Evol Biol*. 2007; doi: 10.1186/1471-2148-7-46.

41. González J, Lenkov K, Lipatov M, Macpherson JM, Petrov DA. High Rate of Recent Transposable Element–Induced Adaptation in Drosophila melanogaster. *PLOS Biol*. Public Library of Science; 2008; doi: 10.1371/journal.pbio.0060251.

42. González J, Karasov TL, Messer PW, Petrov DA. Genome-Wide Patterns of Adaptation to Temperate Environments Associated with Transposable Elements in Drosophila. *PLOS Genet*. Public Library of Science; 2010; doi: 10.1371/journal.pgen.1000905.

43. Itokawa K, Komagata O, Kasai S, Okamura Y, Masada M, Tomita T. Genomic structures of Cyp9m10 in pyrethroid resistant and susceptible strains of Culex quinquefasciatus. *Insect Biochem Mol Biol*. 2010; doi: 10.1016/j.ibmb.2010.06.001.

44. Hof AE van't, Campagne P, Rigden DJ, Yung CJ, Lingley J, Quail MA, et al.. The industrial melanism mutation in British peppered moths is a transposable element. *Nature*. Nature Publishing Group; 2016; doi: 10.1038/nature17951.

45. Feschotte C. Transposable elements and the evolution of regulatory networks. *Nat Rev Genet*. Nature Publishing Group; 2008; doi: 10.1038/nrg2337.

46. Ellison CE, Bachtrog D. Dosage Compensation via Transposable Element Mediated Rewiring of a Regulatory Network. *Science*. American Association for the Advancement of Science; 2013; doi: 10.1126/science.1239552.

47. Santos ME, Braasch I, Boileau N, Meyer BS, Sauteur L, Böhne A, et al.. The evolution of cichlid fish egg-spots is linked with a cis -regulatory change. *Nat Commun*. Nature Publishing Group; 2014; doi: 10.1038/ncomms6149.

48. Alfsnes K, Leinaas HP, Hessen DO. Genome size in arthropods; different roles of phylogeny, habitat and life history in insects and crustaceans. *Ecol Evol*. 2017; doi: https://doi.org/10.1002/ece3.3163.

49. : Trichoptera World Checklist. https://entweb.sites.clemson.edu/database/trichopt/ Accessed 2021 May 8.

50. Wiggins GB, Mackay RJ. Some Relationships between Systematics and Trophic Ecology in Nearctic Aquatic Insects, with Special Reference to Trichoptera. *Ecology*. 1978; doi: https://doi.org/10.2307/1938234.

51. Mackay RJ, Wiggins GB. Ecological Diversity in Trichoptera. *Annu Rev Entomol*. 1979; doi: 10.1146/annurev.en.24.010179.001153.

52. Gurevich A, Saveliev V, Vyahhi N, Tesler G. QUAST: Quality assessment tool for genome assemblies. *Bioinforma Oxf Engl*. 2013; doi: 10.1093/bioinformatics/btt086.

53. Simão F, Waterhouse R, Ioannidis P, Zdobnov E. BUSCO: Assessing genome assembly and annotation completeness with single-copy orthologs. *Bioinforma Oxf Engl*. 2015; doi: 10.1093/bioinformatics/btv351.

54. Waterhouse RM, Seppey M, Simão FA, Manni M, Ioannidis P, Klioutchnikov G, et al.. BUSCO Applications from Quality Assessments to Gene Prediction and Phylogenomics. *Mol Biol Evol*. 2018; doi: 10.1093/molbev/msx319.

55. Laetsch DR, Blaxter ML. BlobTools: Interrogation of genome assemblies. *F1000Research*. 2017; doi: 10.12688/f1000research.12232.1.

873 56. Sayers EW, Bolton EE, Brister JR, Canese K, Chan J, Comeau DC, et al.. Database resources of the
874 national center for biotechnology information. *Nucleic Acids Res*. 2022; doi: 10.1093/nar/gkab1112.

875 57. Challi RJ, Kumar S, Dasmahapatra KK, Jiggins CD, Blaxter M. Lepbase: the Lepidopteran genome
876 database. 2016. http://download.lepbase.org/v4/. Accessed 30 April 2021.

877 58. Heckenhauer J, Frandsen PB, Gupta DK, Paule J, Prost S, Schell T, et al.. Annotated Draft Genomes
878 of Two Caddisfly Species *Plectrocnemia conspersa* CURTIS and *Hydropsyche tenuis* NAVAS (Insecta:
879 Trichoptera). *Genome Biol Evol*. 2019; doi: 10.1093/gbe/evz264.

880 59. Thomas JA, Frandsen PB, Prendini E, Zhou X, Holzenthal RW. A multigene phylogeny and timeline
881 for Trichoptera (Insecta). *Syst Entomol*. 2020; doi: https://doi.org/10.1111/syen.12422.

882 60. Schell T, Feldmeyer B, Schmidt H, Greshake B, Tills O, Truebano M, et al.. An Annotated Draft
883 Genome for Radix auricularia (Gastropoda, Mollusca). *Genome Biol Evol*. 2017; doi:
884 10.1093/gbe/evx032.

885 61. Hanrahan SJ, Johnston JS. New genome size estimates of 134 species of arthropods. *Chromosome
886 Res Int J Mol Supramol Evol Asp Chromosome Biol*. 2011; doi: 10.1007/s10577-011-9231-6.

887 62. Yu Y-S, Jin S, Cho N, Lim J, Kim C-H, Lee S-G, et al.. Genome Size Estimation of Callipogon relictus
888 Semenov (Coleoptera: Cerambycidae), an Endangered Species and a Korea Natural Monument.
889 *Insects*. Multidisciplinary Digital Publishing Institute; 2021; doi: 10.3390/insects12020111.

890 63. Hare EE, Johnston JS. Genome size determination using flow cytometry of propidium iodide-
891 stained nuclei. *Methods Mol Biol Clifton NJ*. 2011; doi: 10.1007/978-1-61779-228-1_1.

892 64. Austin CM, Tan MH, Harrisson KA, Lee YP, Croft LJ, Sunnucks P, et al.. De novo genome assembly
893 and annotation of Australia's largest freshwater fish, the Murray cod (*Maccullochella peelii*), from
894 Illumina and Nanopore sequencing read. *GigaScience*. 2017; doi: 10.1093/gigascience/gix063.

895 65. Pflug JM, Holmes VR, Burrus C, Johnston JS, Maddison DR. Measuring Genome Sizes Using Read-
896 Depth, k-mers, and Flow Cytometry: Methodological Comparisons in Beetles (Coleoptera). *G3
897 GenesGenomesGenetics*. 2020; doi: 10.1534/g3.120.401028.

898 66. Benjamini Y, Speed TP. Summarizing and correcting the GC content bias in high-throughput
899 sequencing. *Nucleic Acids Res*. 2012; doi: 10.1093/nar/gks001.

900 67. Ranallo-Benavidez TR, Jaron KS, Schatz MC. GenomeScope 2.0 and Smudgeplot for reference-free
901 profiling of polyploid genomes. *Nat Commun*. 2020; doi: 10.1038/s41467-020-14998-3.

902 68. Bennett MD, Riley R. The duration of meiosis. *Proc R Soc Lond B Biol Sci*. Royal Society; 1971; doi:
903 10.1098/rspb.1971.0066.

904 69. Cavalier-Smith T. Nuclear volume control by nucleoskeletal DNA, selection for cell volume and cell
905 growth rate, and the solution of the DNA C-value paradox. *J Cell Sci*. 1978; doi: 10.1242/jcs.34.1.247.

906 70. Gregory TR, Hebert PDN. The Modulation of DNA Content: Proximate Causes and Ultimate
907 Consequences. *Genome Res*. 1999; doi: 10.1101/gr.9.4.317.

908 71. CAVALIER-SMITH T. Economy, Speed and Size Matter: Evolutionary Forces Driving Nuclear
909 Genome Miniaturization and Expansion. *Ann Bot*. 2005; doi: 10.1093/aob/mci010.

910 72. Thomas GWC, Dohmen E, Hughes DST, Murali SC, Poelchau M, Glastad K, et al.. Gene content
911 evolution in the arthropods. *Genome Biol*. 2020; doi: 10.1186/s13059-019-1925-7.

912 73. Nakatani Y, McLysaght A. Macrosynteny analysis shows the absence of ancient whole-genome
913 duplication in lepidopteran insects. *Proc Natl Acad Sci U S A*. 2019; doi: 10.1073/pnas.1817937116.

914 74. Li Z, Tiley GP, Rundell RJ, Barker MS. Reply to Nakatani and McLysaght: Analyzing deep
915 duplication events. *Proc Natl Acad Sci*. National Academy of Sciences; 2019; doi:
916 10.1073/pnas.1819227116.

917 75. Roelofs D, Zwaenepoel A, Sistermans T, Nap J, Kampfraath AA, Van de Peer Y, et al.. Multi-faceted
918 analysis provides little evidence for recurrent whole-genome duplications during hexapod evolution.
919 *BMC Biol*. 2020; doi: 10.1186/s12915-020-00789-1.

920 76. Kiauta B, Lankhorst L. The chromosomes of the caddis-fly,*Glyphotaelius pellucidus* (Retzius, 1783)
921 (Trichoptera: Limnephilidae, limnephilinae). *Genetica*. 1969; doi: 10.1007/BF01787334.

922 77. Wolf KW, Novák K, Marec F. Chromosome structure in spermatogenesis of Anabolia furcata
923 (Trichoptera). *Genome*. NRC Research Press; 1992; doi: 10.1139/g92-008.

924 78. Wolf KW, Novák K, Marec F. Kinetic organization of metaphase I bivalents in spermatogenesis of
925 Lepidoptera and Trichoptera species with small chromosome numbers. *Heredity*. 1997; doi:
926 10.1038/hdy.1997.136.

927 79. Darwin Tree of Life – Reading the genomes of all life: a new platform for understanding our
928 biodiversity. https://www.darwintreeoflife.org/ Accessed 19 Jan 2022.

929 80. Lamichhaney S, Catullo R, Keogh JS, Clulow S, Edwards SV, Ezaz T. A bird-like genome from a frog:
930 Mechanisms of genome size reduction in the ornate burrowing frog, Platyplectrum ornatum. *Proc*
931 *Natl Acad Sci*. National Academy of Sciences; 2021; doi: 10.1073/pnas.2011649118.

932 81. Capy P, Gasperi G, Biémont C, Bazin C. Stress and transposable elements: co-evolution or useful
933 parasites? *Heredity*. 2000; doi: 10.1046/j.1365-2540.2000.00751.x.

934 82. Pecinka A, Dinh HQ, Baubec T, Rosa M, Lettner N, Scheid OM. Epigenetic Regulation of Repetitive
935 Elements Is Attenuated by Prolonged Heat Stress in Arabidopsis. *Plant Cell*. 2010; doi:
936 10.1105/tpc.110.078493.

937 83. Tittel-Elmer M, Bucher E, Broger L, Mathieu O, Paszkowski J, Vaillant I. Stress-Induced Activation
938 of Heterochromatic Transcription. *PLOS Genet*. Public Library of Science; 2010; doi:
939 10.1371/journal.pgen.1001175.

940 84. Wiggins G. Larvae of the North American Caddisfly Genera (Trichoptera). London; Toronto,
941 Buffalo: University of Toronto Press; 1996. ISBN 0-8020-2723-7

942 85. Wiggins GB, Caddisflies: The Underwater Architects. Toronto: University of Toronto Press; 2004.

943 86. Dijkstra K-DB, Monaghan MT, Pauls SU. Freshwater biodiversity and aquatic insect diversification.
944 *Annu Rev Entomol*. 2014; doi: 10.1146/annurev-ento-011613-161958.

945 87. Kordiš D, Lovšin N, Gubenšek F. Phylogenomic Analysis of the L1 Retrotransposons in
946 Deuterostomia. *Syst Biol*. 2006; doi: 10.1080/10635150601052637.

947 88. Warren IA, Naville M, Chalopin D, Levin P, Berger CS, Galiana D, et al.. Evolutionary impact of
948 transposable elements on genomic diversity and lineage-specific innovation in vertebrates.
949 *Chromosome Res*. 2015; doi: 10.1007/s10577-015-9493-5.

950 89. Suh A, Churakov G, Ramakodi MP, Platt RN II, Jurka J, Kojima KK, et al.. Multiple Lineages of
951 Ancient CR1 Retroposons Shaped the Early Genome Evolution of Amniotes. *Genome Biol Evol*. 2015;
952 doi: 10.1093/gbe/evu256.

953 90. Grandi FC, An W. Non-LTR retrotransposons and microsatellites. *Mob Genet Elem*. Taylor &
954 Francis; 2013; doi: 10.4161/mge.25674.

955 91. Mackay TFC. Transposable elements and fitness in Drosophila melanogaster. *Genome*. NRC
956 Research Press Ottawa, Canada; 2011; doi: 10.1139/g89-046.

957 92. Pasyukova EG, Nuzhdin SV, Morozova TV, Mackay TFC. Accumulation of Transposable Elements in
958 the Genome of Drosophila melanogaster is Associated with a Decrease in Fitness. *J Hered*. 2004; doi:
959 10.1093/jhered/esh050.

960 93. Langley CH, Montgomery E, Hudson R, Kaplan N, Charlesworth B. On the role of unequal
961 exchange in the containment of transposable element copy number. *Genet Res*. Cambridge
962 University Press; 1988; doi: 10.1017/S0016672300027695.

963 94. Hollister JD, Gaut BS. Epigenetic silencing of transposable elements: A trade-off between reduced
964 transposition and deleterious effects on neighboring gene expression. *Genome Res*. 2009; doi:
965 10.1101/gr.091678.109.

966 95. Lee YCG, Karpen GH. Pervasive epigenetic effects of Drosophila euchromatic transposable
967 elements impact their evolution. Nordborg M, editor. *eLife*. eLife Sciences Publications, Ltd; 2017;
968 doi: 10.7554/eLife.25762.

969 96. Kraaijeveld K. Genome Size and Species Diversification. *Evol Biol*. 2010; doi: 10.1007/s11692-010-
970 9093-4.

971 97. Cosby RL, Chang N-C, Feschotte C. Host–transposon interactions: conflict, cooperation, and
972 cooption. *Genes Dev*. 2019; doi: 10.1101/gad.327312.119.

973 98. Hardie DC, Hebert PD. Genome-size evolution in fishes. *Can J Fish Aquat Sci*. NRC Research Press
974 Ottawa, Canada; 2011; doi: 10.1139/f04-106.

975 99. Rees DJRJ, Dufresne FD, Glémet HG, Belzile CB. Amphipod genome sizes: first estimates for Arctic
976 species reveal genomic giants. *Genome*. 2007; doi: 10.1139/G06-155.

977 100. Dufresne F, Jeffery N. A guided tour of large genome size in animals: what we know and where
978 we are heading. *Chromosome Res*. 2011; doi: 10.1007/s10577-011-9248-x.

979 101. Lertzman-Lepofsky G, Mooers AØ, Greenberg DA. Ecological constraints associated with genome
980 size across salamander lineages. *Proc R Soc B Biol Sci*. Royal Society; 2019; doi:
981 10.1098/rspb.2019.1780.

982 102. Vinogradov AE. Larger genomes for molluskan land pioneers. *Genome*. 2000; doi: 10.1139/g99-
983 063.

984 103. Hotaling S, Sproul JS, Heckenhauer J, Powell A, Larracuente AM, Pauls SU, et al.. Long-reads are
985 revolutionizing 20 years of insect genome sequencing. *Genome Biol Evol*. 2021; doi:
986 10.1093/gbe/evab138.

987 104. Miller SA, Dykes DD, Polesky HF. A simple salting out procedure for extracting DNA from human
988 nucleated cells. *Nucleic Acids Res*. 16:12151988;

989 105. Ruan J, Li H. Fast and accurate long-read assembly with wtdbg2. *Nat Methods*. Nature Publishing
990 Group; 2020; doi: 10.1038/s41592-019-0669-3.

991 106. Walker BJ, Abeel T, Shea T, Priest M, Abouelliel A, Sakthikumar S, et al.. Pilon: An Integrated Tool
992 for Comprehensive Microbial Variant Detection and Genome Assembly Improvement. *PLOS ONE*.
993 Public Library of Science; 2014; doi: 10.1371/journal.pone.0112963.

994 107. Zimin AV, Puiu D, Luo M-C, Zhu T, Koren S, Marçais G, et al.. Hybrid assembly of the large and
995 highly repetitive genome of Aegilops tauschii, a progenitor of bread wheat, with the MaSuRCA mega-
996 reads algorithm. *Genome Res*. 2017; doi: 10.1101/gr.213405.116.

997 108. Bankevich A, Nurk S, Antipov D, Gurevich AA, Dvorkin M, Kulikov AS, et al.. SPAdes: A New
998 Genome Assembly Algorithm and Its Applications to Single-Cell Sequencing. *J Comput Biol*. 2012; doi:
999 10.1089/cmb.2012.0021.

1000 109. Cantarel BL, Korf I, Robb SMC, Parra G, Ross E, Moore B, et al.. MAKER: an easy-to-use
1001 annotation pipeline designed for emerging model organism genomes. *Genome Res*. 2008; doi:
1002 10.1101/gr.6743907.

1003 110. Holt C, Yandell M. MAKER2: an annotation pipeline and genome-database management tool for
1004 second-generation genome projects. *BMC Bioinformatics*. 2011; doi: 10.1186/1471-2105-12-491.

1005 111. Bao W, Kojima KK, Kohany O. Repbase Update, a database of repetitive elements in eukaryotic
1006 genomes. *Mob DNA*. 2015; doi: 10.1186/s13100-015-0041-9.

1007 112. : Kawahara AY, Plotkin D, Espeland M, Meusemann K, Toussaint EFA, Donath A, Gimnich F,
1008 Frandsen PB, Zwick A, Dos Reis M, Barber JR, Peters RS, Liu S, Zhou X, Mayer C, Podsiadlowski L,
1009 Storer C, Yack JE, Misof B, Breinholt JW. Phylogenomics reveals the evolutionary timing and pattern
1010 of butterflies and moths. Proc Natl Acad Sci U S A. 2019 Nov 5;116(45):22657-22663. doi:
1011 10.1073/pnas.1907847116.

1012 113. Misof B, Liu S, Meusemann K, Peters RS, Donath A, Mayer C, et al.. Phylogenomics resolves the
1013 timing and pattern of insect evolution. *Science*. American Association for the Advancement of
1014 Science; 346:763–72014;

1015 114. Luo S, Tang M, Frandsen PB, Stewart RJ, Zhou X. The genome of an underwater architect, the
1016 caddisfly Stenopsyche tienmushanensis Hwang (Insecta: Trichoptera). *GigaScience*. 2018; doi:
1017 10.1093/gigascience/giy143.

1018 115. Haas BJ, Salzberg SL, Zhu W, Pertea M, Allen JE, Orvis J, et al.. Automated eukaryotic gene
1019 structure annotation using EVidenceModeler and the Program to Assemble Spliced Alignments.
1020 *Genome Biol*. 2008; doi: 10.1186/gb-2008-9-1-r7.

1021 116. Lowe TM, Chan PP. tRNAscan-SE On-line: integrating search and context for analysis of transfer
1022 RNA genes. *Nucleic Acids Res*. 2016; doi: 10.1093/nar/gkw413.

117. Camacho C, Coulouris G, Avagyan V, Ma N, Papadopoulos J, Bealer K, et al.. BLAST+: architecture and applications. *BMC Bioinformatics*. 2009; doi: 10.1186/1471-2105-10-421.

118. Götz S, García-Gómez JM, Terol J, Williams TD, Nagaraj SH, Nueda MJ, et al.. High-throughput functional annotation and data mining with the Blast2GO suite. *Nucleic Acids Res*. 2008; doi: 10.1093/nar/gkn176.

119. Katoh K, Kuma K, Toh H, Miyata T. MAFFT version 5: improvement in accuracy of multiple sequence alignment. *Nucleic Acids Res*. 2005; doi: 10.1093/nar/gki198.

120. Kalyaanamoorthy S, Minh BQ, Wong TKF, von Haeseler A, Jermiin LS. ModelFinder: fast model selection for accurate phylogenetic estimates. *Nat Methods*. Nature Publishing Group; 2017; doi: 10.1038/nmeth.4285.

121. Minh BQ, Schmidt HA, Chernomor O, Schrempf D, Woodhams MD, von Haeseler A, et al.. IQ-TREE 2: New Models and Efficient Methods for Phylogenetic Inference in the Genomic Era. *Mol Biol Evol*. 2020; doi: 10.1093/molbev/msaa015.

122. Hoang DT, Chernomor O, von Haeseler A, Minh BQ, Vinh LS. UFBoot2: Improving the Ultrafast Bootstrap Approximation. *Mol Biol Evol*. 2018; doi: 10.1093/molbev/msx281.

123. Zhang C, Rabiee M, Sayyari E, Mirarab S. ASTRAL-III: polynomial time species tree reconstruction from partially resolved gene trees. *BMC Bioinformatics*. 2018; doi: 10.1186/s12859-018-2129-y.

124. Otto F. DAPI staining of fixed cells for high-resolution flow cytometry of nuclear DNA. *Methods Cell Biol*. 1990; doi: 10.1016/s0091-679x(08)60516-6.

125. Dolezel J, Binarova P, Lucretti S. Analysis of Nuclear DNA content in plant cells by Flow cytometry. *Biol Plant*. 1989; doi: 10.1007/BF02907241.

126. Wickham H. ggplot2: Elegant Graphics for Data Analysis. 2nd ed. Springer International Publishing; 2009. https://doi.org/10.1007/978-0-387-98141-3

127. RStudio Team (2020). RStudio: Integrated Development for R. RStudio, PBC, Boston, MA URL http://www.rstudio.com/.

128. Flynn JM, Hubley R, Goubert C, Rosen J, Clark AG, Feschotte C, et al. RepeatModeler2 for automated genomic discovery of transposable element families. *Proc Natl Acad Sci*. National Academy of Sciences; 2020; doi: 10.1073/pnas.1921046117.

129. Repeat masking. *Polistes dominula* Genome Project: https://github.com/PdomGenomeProject/repeat-masking

130. Novák P, Neumann P, Pech J, Steinhaisl J, Macas J. RepeatExplorer: a Galaxy-based web server for genome-wide characterization of eukaryotic repetitive elements from next-generation sequence reads. *Bioinformatics*. 2013; doi: 10.1093/bioinformatics/btt054.

131. Novák P, Ávila Robledillo L, Koblížková A, Vrbová I, Neumann P, Macas J. TAREAN: a computational tool for identification and characterization of satellite DNA from unassembled short reads. *Nucleic Acids Res*. 2017; doi: 10.1093/nar/gkx257.

132. Goubert C, Modolo L, Vieira C, ValienteMoro C, Mavingui P, Boulesteix M. De Novo Assembly and Annotation of the Asian Tiger Mosquito (Aedes albopictus) Repeatome with dnaPipeTE from Raw

Genomic Reads and Comparative Analysis with the Yellow Fever Mosquito (Aedes aegypti). *Genome Biol Evol*. 2015; doi: 10.1093/gbe/evv050.

133. Negm S, Greenberg A, Larracuente AM, Sproul JS. RepeatProfiler: a pipeline for visualization and comparative analysis of repetitive DNA profiles. *bioRxiv*. Cold Spring Harbor Laboratory; 2020; doi: 10.1101/2020.05.22.111252.

134. Heng Li. Toolkit for processing sequences in FASTA/Q formats. 2012. https://github.com/lh3/seqtk. Accessed 20 Jan 2022.

135. Barker MS, Dlugosch KM, Dinh L, Challa RS, Kane NC, King MG, et al. EvoPipes.net: Bioinformatic Tools for Ecological and Evolutionary Genomics. *Evol Bioinforma*. SAGE Publications Ltd STM; 2010; doi: 10.4137/EBO.S5861;

136. Barker MS, Dlugosch KM, Dinh L, Challa RS, Kane NC, King MG, et al. EvoPipes.net: Bioinformatic Tools for Ecological and Evolutionary Genomics. 2010. https://bitbucket.org/barkerlab/evopipes/src/master. Accessed 20 Jan 2022

137. Birney E, Clamp M, Durbin R. GeneWise and Genomewise. *Genome Res*. 2004; doi: 10.1101/gr.1865504.

138. Yang Z. PAML 4: Phylogenetic Analysis by Maximum Likelihood. *Mol Biol Evol*. 2007; doi: 10.1093/molbev/msm088.

139. Cui L, Wall PK, Leebens-Mack JH, Lindsay BG, Soltis DE, Doyle JJ, et al.. Widespread genome duplications throughout the history of flowering plants. *Genome Res*. 2006; doi: 10.1101/gr.4825606.

140. Benaglia T, Chauveau D, Hunter DR, Young DS. mixtools: An R Package for Analyzing Mixture Models. *J Stat Softw*. 2009; doi: 10.18637/jss.v032.i06.

141. Heckenhauer J; Frandsen PB; Sproul JS; Li Z; Paule J; Larracuente AM; Maughan PJ; Barker MS; Schneider JV; Stewart RJ; Pauls SU (2022): Supporting data for "Genome size evolution in the diverse insect order Trichoptera" GigaScience Database. http://doi.org/10.5524/100976

142. Heckenhauer J. TE-gene intersect analysis. 2021. https://github.com/jhcaddisfly/TE-gene_intersect_analysis. Accessed 20 Jan 2022.

Click here to access/download;Table;Table 1.docx

Table 1. Comparison of assembly and annotation statistics of all available Trichoptera Genomes. *Assemblies produced in this study.

**$N_{Arthropoda}$=2124

Species	Abbre-viation	Accession number	Length (bp)	N50 (kbp)	No. of contigs / scaffolds	BUSCOS**
*Agapetus fuscipes**	AF	JAGTXP000000000	552,637,417	2.8	296,752 / 291,536	C:43.8%[S:43.0%,D:0.8%],F:35.2%,M:21.0%
*Agraylea sexmaculata**	AS	JAGTTH000000000	196,044,125	86	7,077 / 7,050	C:94.2%[S:88.6%,D:5.6%],F:1.9%,M:3.9%
Agrypnia vestita [30]	AV	GCA_016648135.1	1,352,945,503	111.8	25,541 / 25,153	C:87.5%[S:77.1%,D:10.4%],F:6.1%,M:6.4%
*Drusus annulatus**	DA	JAGWCC000000000	727,941,535	1,043.7	2,401	C:90.3%[S:89.6%,D:0.7%],F:6.5%,M:3.2%
*Glossosoma conforme**	GC1	JAGTXR000000000	568,249,599	2,212.1	653	C:90.1%[S:89.1%,D:1.0%],F:2.7%,M:7.2%
Glossosoma conforme [124]	GC2	GCA_003347265.1	604,293,666	17.1	132,934 / 119,821	C:78.4%[S:77.3%,D:1.1%],F:15.0%,M:6.6%
Glyphotaelius pellucidula [125]	GP	Glyphotaelius_pellucidus_k51_scaffolds	623,431,006	1.6	461,749	C:20.3%[S:19.7%,D:0.6%],F:39.9%,M:39.8%
*Halesus radiatus**	HR	JAHDVE000000000	973,356,502	125.2	12,636 / 12,484	C:85.0%[S:82.6%,D:2.4%],F:8.5%,M:6.5%
*Himalopsyche phryganea**	HP	JAGVSL000000000	633,785,554	4,634	710	C:96.0%[S:95.3%,D:0.7%],F:2.5%,M:1.5%
Hesperophylax magnus [30]	HM	GCA_016648045.1	1,275,967,528	768.2	6,877	C:88.4%[S:80.7%,D:7.7%],F:6.4%,M:5.2%
Hydropsyche tenuis [57]	HT	GCA_009617725.1	229,663,394	2,190.1	403	C:95.7%[S:94.9%,D:0.8%],F:2.4%,M:1.9%
*Lepidostoma basale**	LB	JAGTTH000000000	769,208,668	1,052	1,712 / 1,621	C:94.4%[S:93.1%,D:1.3%],F:3.5%,M:2.1%
Limnephilus lunatus [69]	LL	GCA_000648945.2	1,369,180,260	69.1	69,049 / 58,718	70.4%[S:66.2%,D:4.2%],F:20.8%,M:8.8%
*Micrasema longulum**	ML2	JAGXCS000000000	668,600,304	2.5	374,883 / 368,330	C:45.0%[S:44.1%,D:0.9%],F:34.5%,M:20.5%
*Micrasema longulum**	ML1	JAGVSM000000000	585,245,295	170.5	5,470/5,451	C:78.6%[S:77.6%,D:1.0%],F:5.4%,M:16.0%
*Micrasema minimum**	MM	JAGVSQ000000000	329,257,313	69.5	7,561	C:59.1%[S:58.6%,D:0.5%],F:10.4%,M:30.5%
*Micropterna sequax**	MS	JAGUCF000000000	778,692,278	7.9	144,300 / 144,286	C:44.4%[S:41.7%,D:2.7%],F:29.6%,M:26.0%
*Odontocerum albicorne**	OA	JAGTXQ000000000	1,305,984,461	266.4	9,583 / 9,303	C:92.2%[S:90.6%,D:1.6%],F:5.4%,M:2.4%
*Parapsyche elsis**	PE	JAGVSN000000000	282,185,525	5,591.7	159	C:95.1%[S:94.3%,D:0.8%],F:1.8%,M:3.1%
*Philopotamus ludificatus**	PL	JAGXCT000000000	360,300,449	67.5	44,049 / 37,274	C:92.0%[S:90.0%,D:2.0%],F:4.7%,M:3.3%
Plectrocnemia conspersa [57]	PC	GCA_009617715.1	396,695,105	869	1,614	C:94.7%[S:93.8%,D:0.9%],F:2.5%,M:2.8%
*Rhyacophila brunnea**	RB	JAGYXB000000000	1,086,872,538	1,030.6	2,227 / 2,125	C:95.4%[S:92.0%,D:3.4%],F:2.5%,M:2.1%
*Rhyacophila evoluta**	RE2	JAGVSQ000000000	565,830,460	9.9	118,140 / 114,057	C:75.1%[S:74.3%,D:0.8%],F:17.9%,M:7.0%

*Rhyacophila evoluta**	RE1	JAGVSO000000000	562,550,625	9.7	115,243 / 111,706	C:74.1%[S:73.4%,D:0.7%],F:18.7%,M:7.2%
Sericostoma sp.[126]	SS	GCA_003003475.1	1,015,727,762	3.2	561,698	C:30.9%[S:30.3%,D:0.6%],F:40.2%,M:28.9%
Stenopsyche tienhuanesis [100]	ST	GCA_008973525.1	451,494,475	1,296.7	552	C:95.3%[S:92.4%,D:2.9%],F:2.3%,M:2.4%

Click here to access/download;Figure;Figure1.pdf

Figure 1

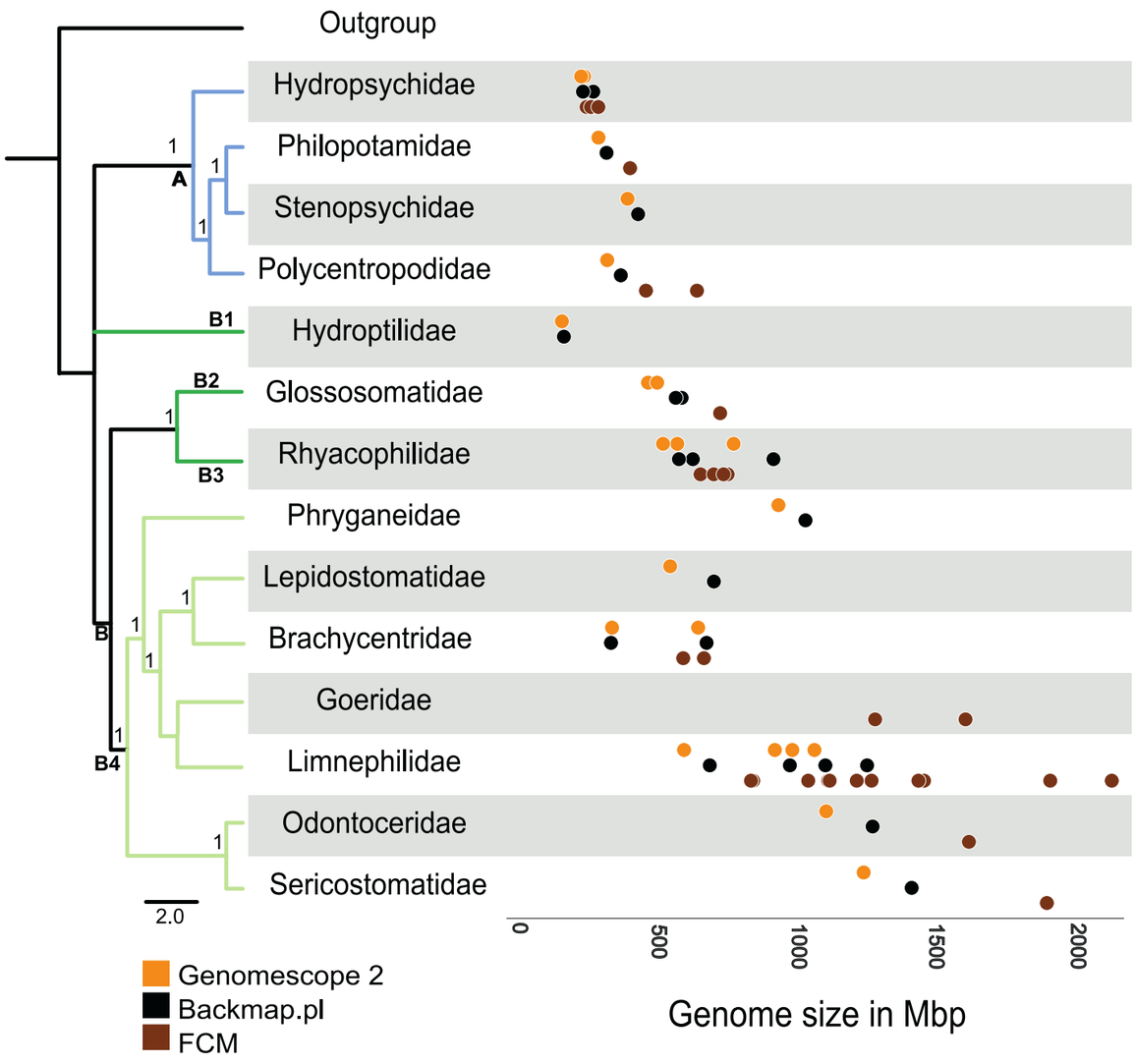

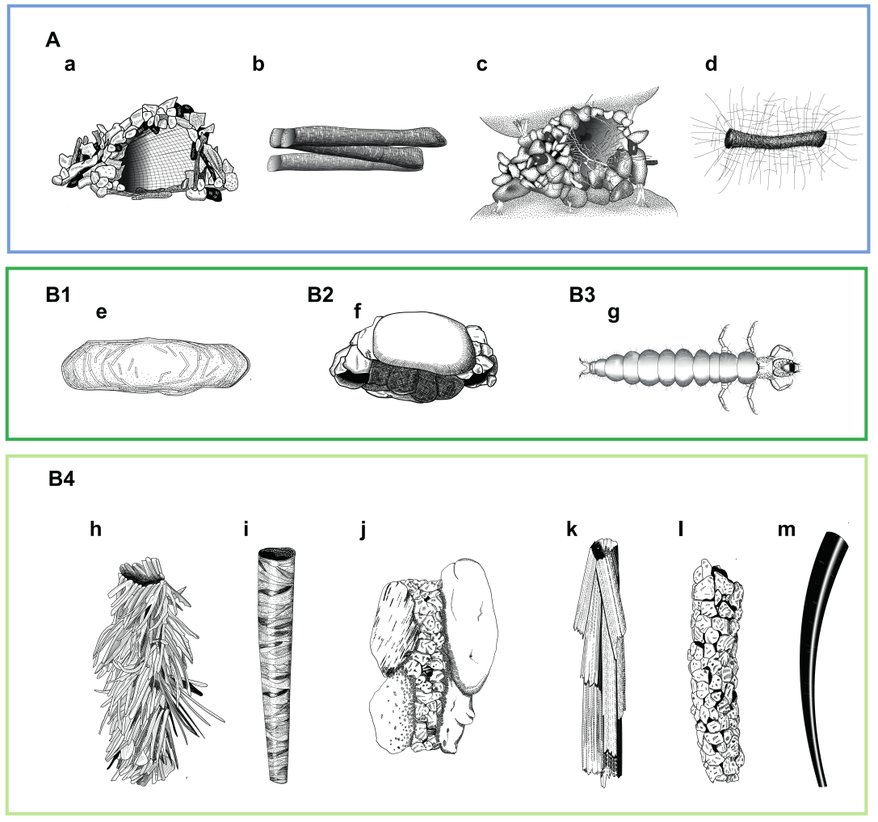

Annulipalpia (‘fixed-retreat- and net-spinners‘)
basal Integripalpia (‘cocoon-builders‘)
Phryganides (‘tube case-builders‘)

Figure 2

Click here to access/download;Figure;Figure2.pdf

A

Repetitive Element Abundance

Annulipalpia

Integripalpia

A

B1

B2

B3

B4

B

H. tenuis
P. elsis
P. conspersa
P. ludificatus
S. tienmushanesis
A. sexmaculata
A. fuscipes
G. conforme 1
G. conforme 2
H. phryganea
R. evoluta 1
R. evoluta 2
R. brunnea
M. minimum
M. longulum 1
M. longulum 2
L. basale
D. annulatus
M. sequax
H. radiatus
H. magnus
G. pelliucidus
L. lunatus
A. vestita
S. sp.
O. albicorne

2.0

0

500 Mb

1000 Mb

Repeat

LINEs

LTRs

DNA transposons

SINEs

satellites

other repeats

unclassified repeats

non-repeat/low copy

B

Repetitive Element Genomic Proportion in Major lineages

genome proportion

0.0

0.1

0.2

0.3

Annulipalpia (fixed-retreat & net)

basal Integripalpia (cocoon)

Phryganides (tube case)

unclassified

LINEs

DNAs

SINEs

LTRs

other

Figure 3

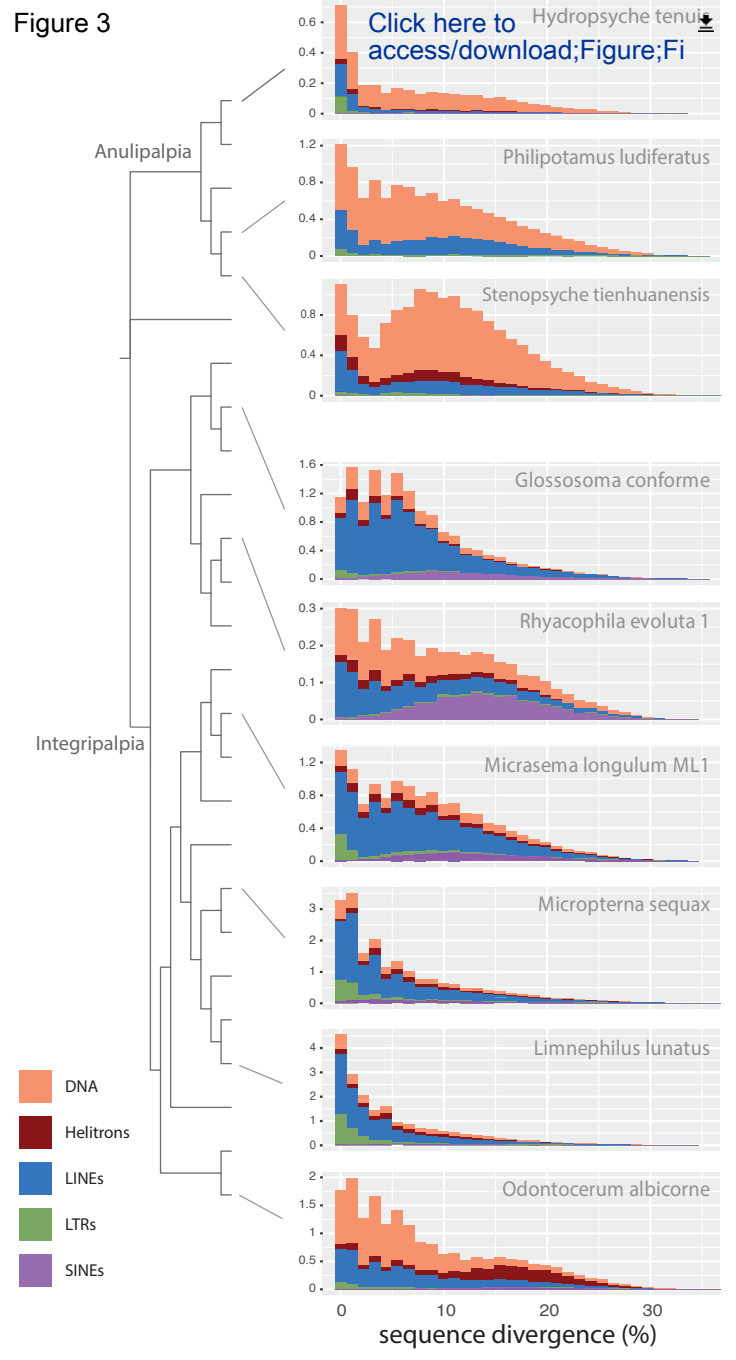
Click here to access/download;Figure;Fi
Hydropsyche tenuis
Philipotamus ludiferatus
Stenopsyche tienhuanensis
Glossosoma conforme
Rhyacophila evoluta 1
Micrasema longulum ML1
Micropterna sequax
Limnephilus lunatus
Odontocerum albicorne
Anulipalpia
Integripalpia
DNA
Helitrons
LINEs
LTRs
SINEs
sequence divergence (%)

Figure 4

Click here to access/download;Figure;Figure4.pdf

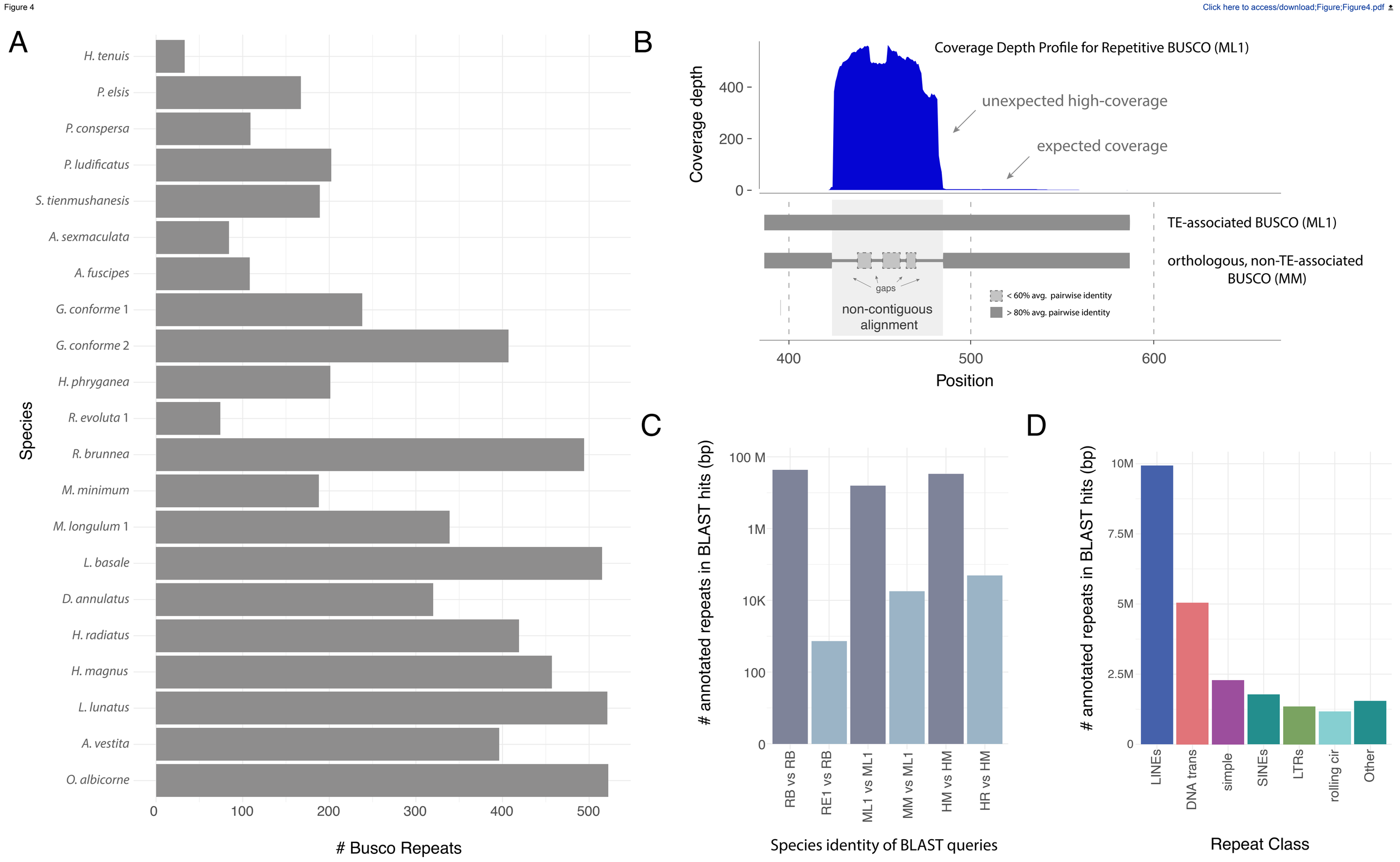

Click here to access/download
Supplementary Material
Supplementary Material.docx

Additional Data File

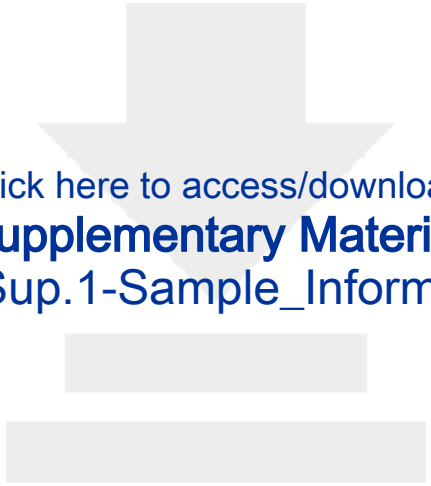

Click here to access/download

Supplementary Material

DataS1_Sup.1-Sample_Information.xlsx

Additional Data File

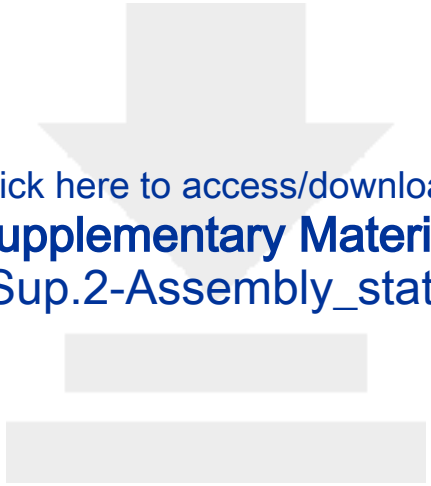

Click here to access/download

Supplementary Material

DataS1_Sup.2-Assembly_statistics.ods

Click here to access/download
Supplementary Material
DataS1_Sup.3_Sequencing_coverages.xlsx

Additional Data File

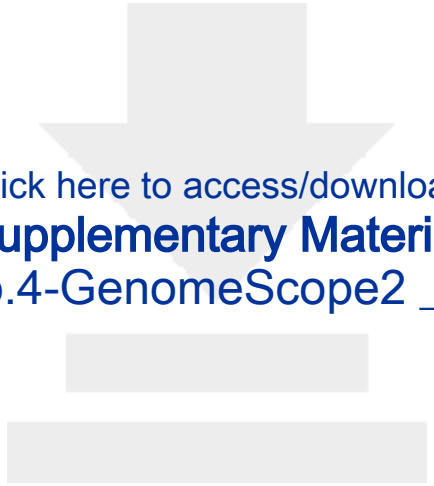

Click here to access/download

Supplementary Material

DataS1_Sup.4-GenomeScope2 _Results.xlsx

Additional Data File

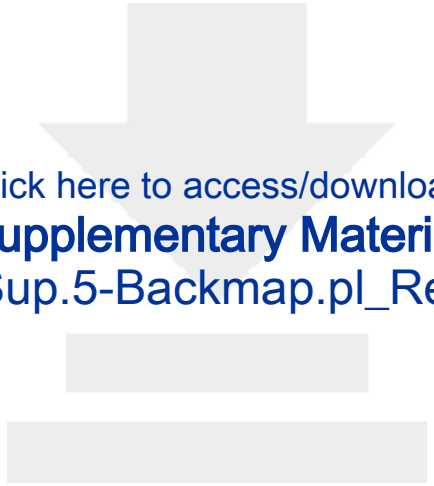

Click here to access/download

Supplementary Material

DataS1_Sup.5-Backmap.pl_Results.xlsx

Additional Data File

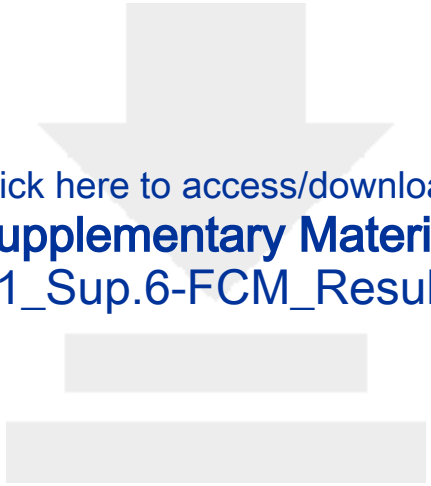

Click here to access/download

Supplementary Material

DataS1_Sup.6-FCM_Results.xlsx

Additional Data File

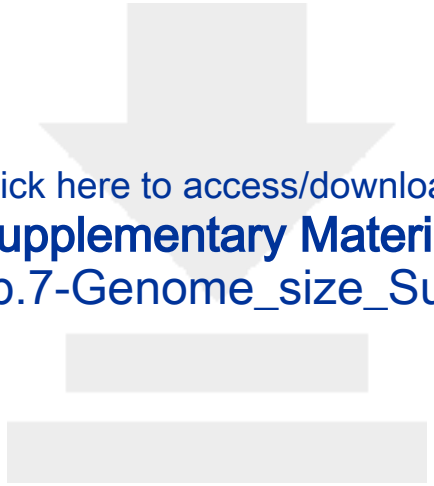

Click here to access/download

Supplementary Material

DataS1_Sup.7-Genome_size_Summary.xlsx

Additional Data File

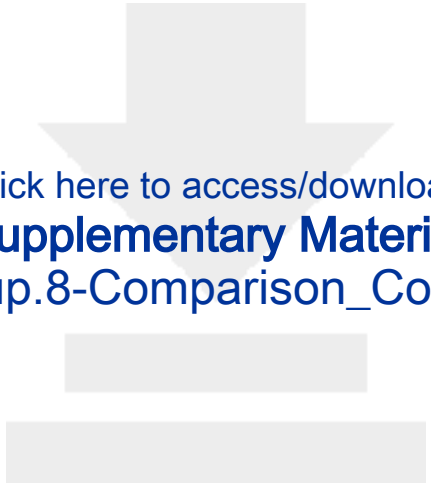

Click here to access/download

Supplementary Material

DataS1_Sup.8-Comparison_Coverage.ods

Additional Data File

Click here to access/download
Supplementary Material
DataS1_Sup.9-List_of_Transcriptomes.xlsx

Additional Data File

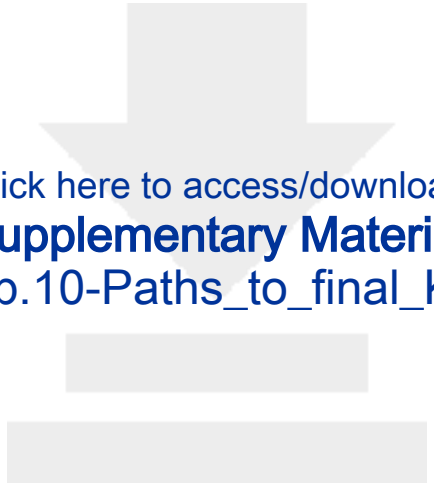

Click here to access/download

Supplementary Material

DataS1_Sup.10-Paths_to_final_Ks_files.xlsx

Additional Data File

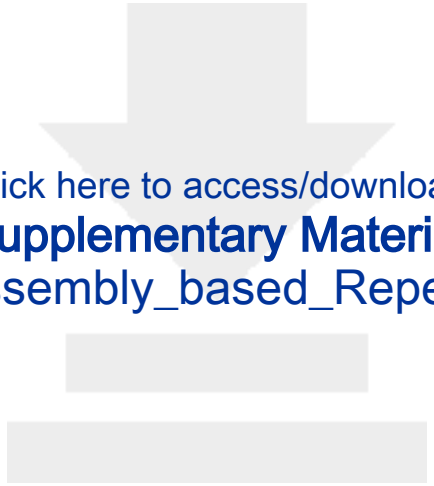

Click here to access/download

Supplementary Material

DataS2_Sup.1-Assembly_based_Repeat_Summary.xlsx

Additional Data File

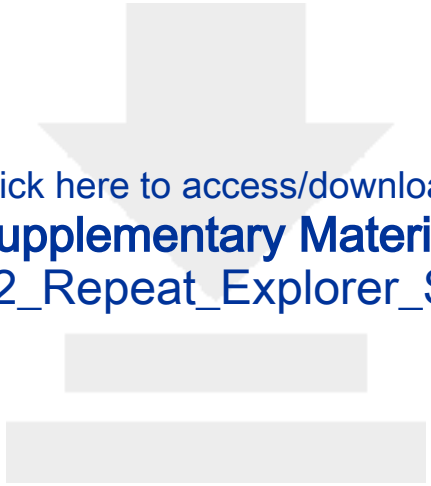

Click here to access/download

Supplementary Material

DataS2_Sup.2_Repeat_Explorer_Summary.xlsx

Additional Data File

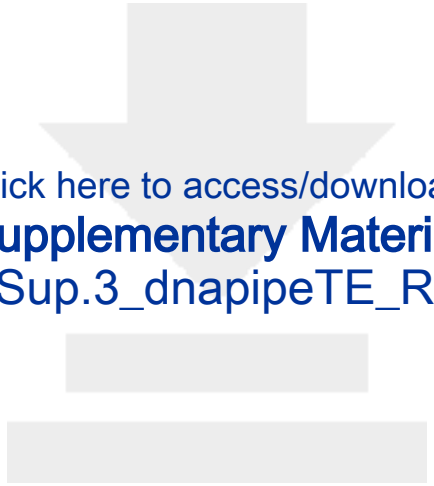

Click here to access/download

Supplementary Material

DataS2_Sup.3_dnapipeTE_Results.txt

Additional Data File

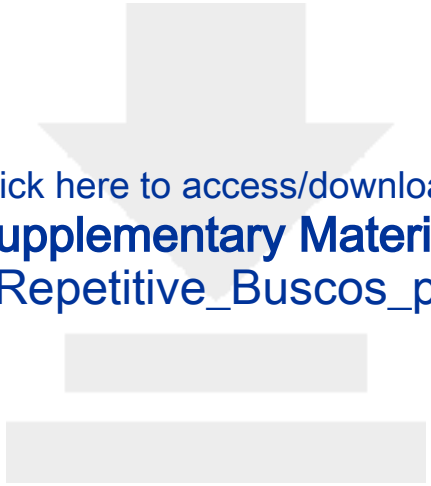

Click here to access/download

Supplementary Material

DataS2_Sup.4_Repetitive_Buscos_per_Species.xlsx

Additional Data File

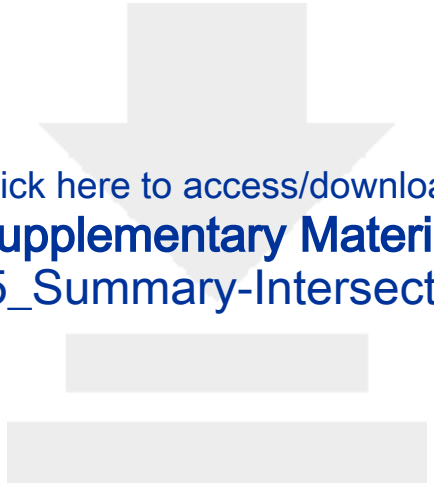

Click here to access/download

Supplementary Material

DataS2_Sup.5_Summary-Intersect_Analysis.xlsx

Additional Data File

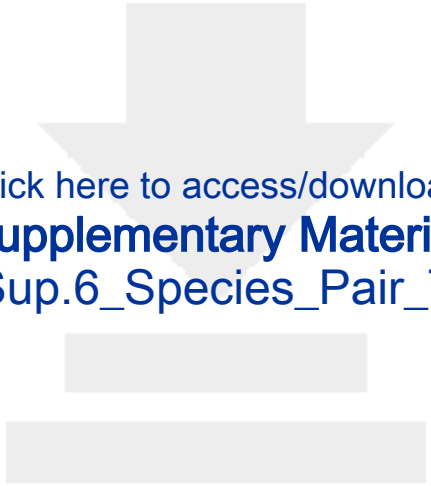

Click here to access/download

Supplementary Material

DataS2_Sup.6_Species_Pair_Tests.xlsx

Personal Cover Click here to access/download;Personal Cover;Cover Letter.doc

Jacqueline Heckenhauer, PhD
LOEWE Centre for Translational Biodiversity Genomics
Senckenberg Research Institute and Natural History Museum
Email: jacqueline.heckenhauer@senckenberg.de
2021-12-14 Frankfurt (Main)

Cover letter

Dear Hongling Zhou, and, dear Editorial Board,

We would like to re-submit our revised manuscript entitled “Genome size evolution in the diverse insect order Trichoptera (caddisflies)” for review after incorporating feedback from the reviewer.

We thank both reviewers for reading our revised version of the manuscript. We, especially, thank reviewer 1 for their constructive feedback which we have carefully considered.

We made changes to the manuscript and believe that the manuscript has improved as a result. The revised manuscript addresses the concerns raised and was submitted via the submission portal (with and without using Track Changes). A point-by-point response to the reviewers’ suggestions can be found below.

Again, we thank you for the opportunity to revise our manuscript. Please let us know if there is anything else you require from us.

Sincerely,

Jacqueline Heckenhauer on behalf of all authors

Point-by-point 'Response to Reviewers':

Reviewer reports:

Reviewer #1: I thank the authors for their well-written, thorough, and detailed responses to my concerns and those of the other reviewer. I believe most of my concerns have been satisfactorily addressed. I find the revised manuscript suitable for publication, after one minor issue is further addressed (see below). The scale of data and analyses in this paper is no small feat.

Minor comment:

I appreciate that the authors have run smudgeplot analyses on their data, but I do notice a few inconsistencies in coverage estimates between those based on sequencing effort and the backmapping approach, and the kmer approaches. It is, therefore, in my opinion, still worth comparing the kcov to actual sequencing effort for the samples where smudgeplots are unclear and/or inconsistent with the other coverage estimates. I realise these two methodological approaches are based on different data streams (kmers versus assemblies), but considering their shared origin, they are still worth comparing. See example below:

O. albicorne has varying coverage estimates (sequencing effort, 2n: 46.7, backmapping, 2n: 58, genomescope, 1n: 24.8, smudgeplot, 1n: 53.8)

If you convert sequencing-based coverage to kcov:

Kcov, 1n =(((read length -k + 1) coverage)/read length)/2*

This gives a kcov 1n estimate between 20.3 and 25.1, more in line with the genomescope estimate, suggesting that smudgeplot should be re-run with manually entered kcov as predicted by genomescope

Other incongruent examples, where at least one of the coverage estimates is inconsistent, include: H. phryganea, H. magnus, L. basale, andM. longulum

This comparison, and the adjustment of the genomescope kcov, should tidy up a few lose ends and overall help support more of the results of the various analyses. This may suggest that some of these species might not be diploid, but I do agree with the authors that further sequencing would need to be done to confirm this, and this is outside the scope of this manuscript. A brief mention of these different lines of evidence for/against polyploidy, and their (dis)agreement with each other should be sufficient once the above samples are addressed.

Response:

We thank reviewer 1 for their constructive feedback which we addressed as follows:

1. We calculated kcov for each sample and modified "DataS1_Sup.8-Comparison_Coverage_kcov_backmap.pl_GenomeScope2_Smudgeplots". This file now includes two additional columns: the kcov for each sample and the backmapping coverage (which is also reported in DataS1_Sup.5-Backmap.pl_Results). This makes it easier for the reader to compare the different lines of evidence, i.e. sequencing based coverage (2n), kcov (1n), backmapping coverage (2n), genomescope2 kcov (1n), and coverage used in smudgeplot (1n).

2. We refer to incongruencies of coverage estimates pointed out by the reviewer (*O. albircorne, H. phryganea, H. magnus, L. basale,* and *M. longulum*) in the supplementary (page 48) and main text at lines 303-311.

3. We re-ran smudgeplot with the manually entered kcov as predicted by Genomescope 2 for *O. albircorne, H. phryganea, H. magnus, L. basale,* and *M. longulum*, see lines 303-311 in the main text. The smudgeplots are reported in the supplementary figures S90-93 (*H. phyrganeae*), S96-98 (*H. magnus*), S104-S107 (*L. basale*), S110-S113 (*M. longulum* ML3), S116-119 (*M. longulum* ML1), S127-S129 (*O. albicorne*).

4. We report these new results in the main text (lines 303-311), and discuss the different lines of evidence for/against polyploidy and their (dis)agreement with each other (332-337).

5. As requested by the GigaDB Team we ran BUSCO v5 for the completeness analysis, updated the summary statistic shown in Table 1 with the v5 results and will uploaded the BUSCO results files in GigaDB: 3 files for each run; (i)short_summary.*.txt (ii) full_table.tsv (iii)missing_busco_list.tsv. We changed this in the methods accordingly, lines 118, 469-470.
