# Supplementary material for: Genome size evolution in the diverse insect order Trichoptera
Source: Gigascience. 2022 Feb 25;11:giac011. doi: 10.1093/gigascience/giac011 (PMC8881205; doi:10.1093/gigascience/giac011)
Supplement: giac011_Response_to_Reviewer_Comments_Revision_1 [file giac011_response_to_reviewer_comments_revision_1.pdf]

Author's Response To Reviewer Comments

Close

Dear Hongling Zhou, and, dear Editorial Board,
We would like to thank you for the opportunity to revise our manuscript and respond to the important points raised by qualified reviewers.
We would like to re-submit our revised manuscript entitled “Genome size evolution in the diverse insect order Trichoptera (caddisflies)” for review after incorporating feedback from both reviewers.
We thank both reviewers for their insightful critiques and interest in our manuscript, which nearly triples the number of available caddisfly genomes, explores the role of transposable element expansions in genome size evolution of caddisflies and provides a new method for detection of TE sequence associations with protein-coding genes.
Since both reviewers stated that they found the description of this new method difficult to understand, we undertook a substantial revision to the relevant sections in the manuscript.
Another suggestion of one reviewer was to conduct further analyses to investigate the role of WGD events in the evolution of genome size in caddisflies. As recommended by the reviewer, we ran Smudgeplot and did not detect evidence for polyploidy or WGD events. While this, of course, does not mean that we can completely rule out that WGD plays a role in genome size evolution in Trichoptera, the bulk of the evidence from our study suggests that repetitive elements played the most important role and we focus primarily on their role in this study. We reformulated the discussion to emphasize this.

The revised manuscript addresses the concerns raised and was submitted via the submission portal (with and without using Track Changes). A point-by-point response to the reviewers’ suggestions can be found below.
Again, we thank you for the opportunity to revise our manuscript. Please let us know if there is anything else you require from us.

Sincerely,

Jacqueline Heckenhauer on behalf of all authors

Point-by-point 'Response to Reviewers':

Reviewer reports:

Reviewer #1: Summary of the paper and overall impression
In their paper, "Genome size evolution in the diverse insect order Trichoptera", Heckenhauer et al report a 14-fold variation in genome size in caddis-flies. The authors find evidence for increases in transposable elements associated with larger genomes, and report that in caddis-flies living in less stable environments, some genes are replicated in association with transposable elements. Overall, this paper represents a comprehensive collection of data, however, I have some concerns about some of the reporting of methods, some analyses and conclusions. To support some of the conclusions, namely that WGD or large-scale duplications do not play a role in caddis fly genomes, I believe the authors could perform additional analyses. Further, I was left confused by the descriptions of the methods, especially around the replicated BUSCO gene analyses. Please see my comments below.

Response: We thank the reviewer for their valuable comments and suggestions. We address these below.

Main comments:
1. The authors report that their gene-age distribution analyses do not support the hypothesis of a WGD, but given previous suggestions that WGD are important in these species, the authors should conduct additional analyses (e.g smudgeplot, minor allele frequency distributions in single-copy genes) to rule out this possibility. While it can indeed be difficult to find a balance between the evidence of absence and an absence of evidence, more effort should go into resolving the matter of WGD in caddis-flies.
Some of the genomescope peaks, and some of the coverage peaks from the backmap approach seem to

at least hint at large-scale duplications or variations in copy number. Further analyses should also consider if assembled gene copies may be collapsed duplicates.

Response: We appreciate this critique and conducted additional analyses to detect WGD events. As suggested by the reviewer, we ran Smudgeplot on all genomes. This is described in detail in the main text (lines 294-304, 323-326, 577-587, supplementary note 9) and smudgeplots are given in the supplementary material (Figs. S74-121). The new, additional analyses, did not uncover evidence for WGD. This does, of course, not mean that we can entirely rule out that WGD played a role in genome size evolution in Trichoptera. However, we do find strong evidence that repetitive elements played a key role in genome size expansion in this study. We reformulated this in the discussion accordingly (lines 323-331). We hope that further studies using chromosome-length assemblies might help to resolve the matter of WGD in caddisflies. However, generating these is out of the scope of this present study. The emergence of PacBio HiFi genomes of caddisflies (e.g., Darwin Tree of Life is currently planning to sequence 28 caddisfly genomes) might be useful to further test the role of WGD events in the evolution of Trichoptera. We have emphasized this in the revised version of the manuscript (lines lines 323-331).

2. I admit I am confused by the terminology around the TE-associated BUSCO genes. Are these cases where BUSCO has reported a high number of duplicates? Or where BUSCO annotated regions have a high coverage? Two things need to be clarified here; what made them stick out in the first place (coverage? Duplications?), and what are they really (TEs that BUSCO mistook for BUSCOs? fragments of real BUSCOs attached to TEs?).

Response: We thank the reviewer for expressing this confusion and suggesting points that need clarification. To address both reviewers' comments (see also reviewer 2, main comment 1), we thoroughly reworked our presentation of the TE-associated BUSCO analysis and results to help readers to understand the concept of this new discovery (lines 234–281, 536-559, 639-656, supplementary note 11).

To answer Reviewer 1's questions here: (1) These BUSCOs first stuck out because we had mapped reads to all BUSCOs as part of our early data exploration and noted that a subset of BUSCO genes had sequence regions with unexpectedly high coverage when visualizing read depth profiles, and this was especially pronounced in species with large genome sizes. (2) The high coverage regions turned out to be fragments of repetitive elements (especially transposable elements) inserted within or immediately adjacent to BUSCOs, and incorrectly annotated by the BUSCO algorithm. Most of our methods/results, which caused some confusion to both reviewers were describing methods used to validate that the high coverage regions do, in fact, consist of TE-derived sequences.

We improved our presentation of this topic in three ways. First, in our presentation of this topic (lines 234–244), we now lead off by answering both questions raised by Reviewer 1 above up front. Only then do we go into more detail (see pasted text below). Second, we use more effective and standardized terminology throughout. Instead of using the somewhat vague/misleading term, "repetitive BUSCOs", we use the more correct and descriptive term "TE-associated BUSCOs" throughout the text and in Fig.4. Third, in supplementary note 11, we integrated results alongside methods so that the flow of methodological logic and the results from each intermediate analysis are integrated and all in one place. In addition to these major edits, we include minor adjustments to the text in lines noted above. We feel the manuscript is much improved through addressing both reviewers' feedback on this point.

(**pasted from lines 234–244) "During early exploration of our sequence data, we made an unexpected discovery that in some lineages, universal single copy orthologs, or "BUSCO genes," showed higher than expected coverage depth of mapped reads in one or more of their sequence fragments. Further analysis showed that these high-coverage BUSCO sequence regions are typically RE sequences (primarily TEs) that are either embedded within or located immediately adjacent to BUSCO genes, such that the BUSCO algorithm includes them in its annotation of a given gene. We refer to BUSCO genes containing these putative RE fragments as 'TE-associated BUSCOs' (supplementary Fig. S125, supplementary note 11). By estimating how many times they occur, we can quantitatively measure how TE-gene interactions change with changing genome size. In fact, we detected a positive linear relationship between TE-gene interactions and increasing genome size when measured with this metric."

Minor comments:
1. Lines 53-57: "Genome size can vary widely among closely and distantly related species, though our knowledge is still scarce for many non-model groups. This is especially true for insects, which account for much of the earth's species diversity. To date 1,345 insect genome size estimates have been

published, representing less than 0.15% of all described insect species." While I appreciate the authors' point that there is a relatively little data available about genome size and only a small proportion of non-model insects in the Animal Genome Size database, this is the case for all groups, and insects actually represent the largest group of invertebrates in the AGSDb. However, this does not mean insects, or chironomid are a poor system to study this in, so authors could reframe this first sentence to justify the study system with something more than highlighting how understudied this is in insects.

Response: This is an important point. We reformulated the beginning of the background section (lines 54-61) as follows: "Genome size is a fundamental biological character. Studying its evolution may potentially lead to a better understanding of the origin and underlying processes of the myriad forms and functions of plants and animals, subjects which remain at the core of much biological research. Given their high species, ecological and functional diversities, insects are excellent models for such research. To date 1,345 insect genome size estimates have been published (Gregory, 2005: Animal Genome Size Database: http://www.genomesize.com, last accessed 2021-04-30) ranging 240-fold from 69 Mbp in chironomid midges [1] to 16.5p Gbp in the mountain grasshopper Podisma pedestris [2].

2. Line 76: correct to "In insects, the KNOWN ranges of genomic repeat proportion are…"

Response: We agree and corrected this sentence (lines 76-78): "In insects, the known genomic proportion of TEs ranges from 1% in the antarctic midge Belgica antarctica [26] to 65% in the migratory locust Locusta migratoria [27]."

3. Lines 89-91: Why are species rich groups a better system to study RE evolution and environmental interactions than e.g. populations, species complexes, recently diverged species, or groups in the process of speciation?

Response: Thank you for raising this point. As the reviewer points out we didn't present the most effective logical argument here. We updated this section (lines 87-95) to better represent our logic as follows: "Investigating RE dynamics in diverse clades provides a powerful lens for understanding their roles in genome function and evolution. Broadly studying of RE dynamics in species-rich groups with wide variation in RE activity is an important step towards efficiently identifying study systems at finer taxonomical scales (natural populations, species complexes, or recently diverged species) that are ideally suited to advance our understanding of molecular and evolutionary mechanisms underlying genome evolution. In addition, by taking this biodiversity genomics approach, we can develop new model systems and eventually better understand links between environmental factors, genome size evolution, adaptation, and speciation (see [47])."

4. Lines 113-115: The data description does not, in my opinion, need to justify the species selection since this is done in the intro

Response: Thanks for this suggestion. We deleted the first two sentences of the data description (line 114).

5. Genome size estimates- sequencing based estimates can also be impacted by GC-content, especially in libraries which were produced using PCR, this may be a useful point regarding the differences between FCM and sequencing-based estimates

Response: We added this point in the main text (lines 164-166).

6. RepMod versions inconsistent Line 463 says v2, earlier says v1

Response: We used RepeatModeler2.0 and corrected the text in line 439.

7. Line 468-469- What did you use to merge repmask out files?

Response: To merge the repeatmasker out files, we parsed the output tables from each of the two repeatmasker runs with a script (RM_table_parser_families_.py) and then combined the resulting data columns for the two runs in Excel. We added this information in lines 507-510. The script has been uploaded to https://github.com/jhcaddisfly/TE-gene_intersect_analysis.

8. All read-based analyses: were they run on decontaminated read libraries? If so, please briefly clarify this in the main manuscript. Genome size with GenomeScope: 444-448; RepeatExplorer: Lines 471-479

Response: Yes, all read-based analyses were run on decontaminated read libraries. We clarified this in lines 483 and 517.

9. Why only use dnaPipeTE for repeat divergences and not also abundances? Does dnaPipeTE agree with RepeatExplorer?

Response: As suggested, we now include dnaPipeTE-based abundances, as well. The results were overall congruent to those obtained by RepeatMasker and RepeatExplorer2 and the primary message of our manuscript is consistent among all three methods. Compared to RepeatExplorer, dnaPipeTE resulted in more thorough annotation of DNA transposons (which were very uncommon in the RepeatExplorer output, but commonly annotated in our primary approach with RepeatModeler/RepeatMasker), simple repeats, Helitrons, and some other categories. This results in a smaller proportion of unclassified repeats compared to RepeatExplorer. We note that there is a stylistic difference in output between the methods. The dnaPipeTE plot (supplementary note 10, Figure S123) has more repeat categories shown, and the categories are in a different order. However, for shared categories, we use the same color scheme between both plots. We adjusted the main text to include dnapipeTE (lines 208-210, 511-512, 520) and added the results to supplementary note 10, and supplementary figure S123.

10. Line 495: What is meant by "BUSCO genes showed regions of unexpected high copy number…"? Are these genes reported by BUSCO as duplicated or is this referring to increased coverage?

Response: Here, "copy number" referred to coverage depths. We updated the language to clarify. The updated language is pasted in our previous response above (see response above, reviewer 1, main comment 2).

11. Lines 506-507: "We used copy number profiles to identify BUSCO genes with repetitive sequences based on coverage profiles" The meaning of this is unclear. The reported copy number from BUSCO? Coverage of mapped reads?

Response: The copy number profiles were generated through mapping reads back to BUSCO sequences and are not reported in BUSCO output. We clarified this text in response to an earlier comment from the reviewer (see response above to reviewer 1, main comment 2).

12. Table 1- please report the full BUSCO summary (e.g. C:39.7%[S:39.2%,D:0.5%],F:35.8%,M:24.5%,n:2442) for each species, lumping complete and fragmented together is unneccesary, and readers are usually interested enough in the full complement of BUSCOs that it should not be in the supplements, but in the main paper

Response: We adjusted table 1 according to the reviewer's suggestion.

13. Coverages from backmap method can and should be compared to genomescope kcov estimates (while correcting for kmer size; see here for a brief explanation https://www.biostars.org/p/221672/), this will validate both approaches and offer further evidence when considering polploidy.

Response: We hope that we understand the reviewer's suggestion correctly. We did not compare mapping coverage of the backmap method with the Genomescope 2 kcov estimates. We think that it is important to make a fundamental distinction between these values since these are derived from two different methods/algorithms. However, we agree that it is interesting to compare kcov estimates between Genomescope2 and smudgeplots. We did this and the results are given in table "DataS1_Sup.8-Comparison_GenomeScope2_Smudgeplots".

14. In the supplementary note about TAGC plots, Figures S31, S36, S38, S44, S45, S46, S47 don't list contaminant exclusion criteria- if contaminants weren't removed this needs to be stated, and in some cases, especially those where there are different "blobs", (e.g. S47) justified

Response: We only classified contigs with taxonomic assignment to phyla other than Arthropoda as contaminated, when these also showed different GC content and coverage. The taxonomic assignment may be not as expected because a) some contigs are just too small to be recognized correctly and b) there might be conflict in the assignment and therefore Blobtools just declares these contigs as no-hit (see "taxrule" in Blobtools manual). A valid reason for not excluding any contigs of assemblies shown in

figures S31, S36, S38, S44, S45, S46, S47 is that in every "fraction" of the blobs there are contigs assigned to arthropods. Moreover, after uploading the genomes to NCBI, the NCBI genome assembly processing pipeline served as a second quality control step. In a few cases (e.g., S38, S44, S45) NCBI's contamination screen identified additional contamination. These were filtered out manually or by NCBI. We added this information to supplementary note 5.

15. Supplementary note 9: Figure reference is wrong?

Response: We have corrected the Figure reference in supplementary note 10 (before supplementary note 9).

16. Supplementary note 10: Can coverage comparisons using average BUSCO coverage be re-run using corrected kcov estimates? This would validate the BUSCO coverage approach.

Response: Our understanding from this comment is that there is a lack of clarity in describing what we mean by repetitive BUSCOs / and average BUSCO coverage. We reformulated these definitions in the main text (see respond to reviewer 1, main comment 2).

17. Supp Data 1: Coverage estimates would be more accurate if based on FCM measurements and total sequenced bp (before and after decontamination) and can also be compared to corrected kcov estimates

Response: We recalculated sequencing coverage based on total sequenced base pairs before and after decontamination as well as FCM measurements if these were available. For sequences without FCM, we used the Genomescope2 genome size estimates as a basis for coverage calculation ("DataS1_Sup.2-Assembly_statistics", "DataS1_Sup.3_Sequencing_coverages"). These results, as well as the results of the corrected kcov estimates obtained by Genomescope2 and smudgeplots are given in "DataS1_Sup.8-Comparison_GenomeScope2_Smudgeplots".

18. Limnephilus lunatus has too low coverage to get reliable genomescope

Response: We agree and exclude the results from Limnephilus lunatus from Genomescope2 as well as from the backmap approach. We also realized that the coverage of Glyphothaelius was too low for backmap.pl. In addition, we realized that for previously published genomes (Sericostoma, Stenopsyche, Glosossoma), we used all short-read used in the assembly to estimate genome sizes backmap.pl. However, since these belong to multiple individuals, we reran the contamination filtering, backmap.pl on only reads from one individual (see "DataS1_Sup.3_Sequencing_coverage" for accession number of reads used) belonged to multiple individuals. However, the resulting genome size estimates did not change much. We updated the results in the supplementary files, rerun the Bland-Altman-analysis and modified figure 1.

Reviewer #2: This paper presents 17 new insect genomes from the order of caddisflies (Trichoptera). The authors combine these genomes with 9 previously sequenced genomes to analyze genome size evolution across the order. They find that genome size tends to correlate with evolution of repeat elements, specifically expansion of transposable elements (TEs). Interestingly, the authors also notice that TE expansions also correlate with gene copy-number (or gene fragment copy-number), even of highly conserved genes used to assess genome completeness. Overall, I find this paper very well written and easy to follow. The genomic resources and analyses presented provide novel new resources and findings for insects in the order Trichoptera, with potential implications beyond. I have only minor suggestions before publication, outlined below.

Response: We thank the reviewer for the kind comments, we are pleased to hear that our manuscript was easy to follow and our new resources and findings are appreciated.

1. Regarding the TE and BUSCO gene fragment associations, while I think this is a really interesting analysis, I found the underlying models a bit difficult to understand.
Line 236 reads, "To test whether repetitive fragments were due to TE insertions near or in the BUSCO genes or, conversely, due to the proliferation of 'true' BUSCO protein-coding gene fragments…"
Is the idea that a BUSCO gene has been duplicated itself and then one copy is either fragmented by a TE insertion or hitch-hikes with a TE (as mentioned on line 501)? Or are these fragments only of BUSCO genes that didn't match a full BUSCO gene at all, but the fragments that did match had unexpectedly high coverage? I guess I'm just confused as to whether a gene duplication needs to precede the TE insertions/hitch-hiking, which is subsequently pseudogenized either prior to or because of the TE

activity, or if these are gene losses. I understand how the TE could inflate the coverage of these fragments, but I guess I'm still not clear on how these fragments arise in the first place. Any clarification would be helpful! Also, if the case is that these are fragments of BUSCO genes that have no full matches in the genome, how might assembly contiguity or quality be affecting these matches?

Response: Thank you for raising this point, which was also raised by reviewer 1. As described in our response to Reviewer 1 (main comment 2), we have thoroughly revised our presentation of the TE-associated BUSCO gene analysis/results to clarify points raised by both reviewers. The revised text we pasted in our response is pasted again below with additional text from the manuscript and addresses the reviewer’s question about how these fragments arise in the first place.
Regarding the additional point on assembly quality raised by the reviewer here, we considered potential impact of the assembly quality on our conclusion on the TE-gene association study. One line of evidence that suggests assembly artefacts are not driving the pattern of increasing TE-associated BUSCOs with increasing assembly size is the fact that in less contiguous assemblies we found notably fewer TE-associated BUSCOs (including in replicate conspecifics with varying assembly quality). If assembly artefacts in low-quality assemblies were driving the abundance of TE-associated BUSCOs we would have expected to find an increase of these with decreasing assembly quality. Moreover, when mapping Nanopore reads back to an exemplary subset of TE-associated BUSCOs, we observed that junctions between TE-associated and non- TE-associated regions are embedded in full reads and do not occur disproportionately at ends of reads. For this reason, we conclude that our analysis detected valid TE-gene associations and not simply assembly artefacts (this test is explained in detail in supplementary note 12).

(**pasted from lines 234–244) “During early exploration of our sequence data, we made an unexpected discovery that in some lineages, universal single copy orthologs, or “BUSCO genes,” showed higher than expected coverage depth of mapped reads in one or more of their sequence fragments. Further analysis showed that these high-coverage BUSCO sequence regions are typically RE sequences (primarily TEs) that are either embedded within or located immediately adjacent to BUSCO genes, such that the BUSCO algorithm includes them in its annotation of a given gene. We refer to BUSCO genes containing these putative RE fragments as ‘TE-associated BUSCOs’ (supplementary Fig. S125, supplementary note 11). By estimating how many times they occur, we can quantitatively measure how TE-gene interactions change with changing genome size. In fact, we detected a positive linear relationship between TE-gene interactions and increasing genome size when measured with this metric.” And from lines 251-254: “The BUSCO-associated sequences may represent TEs recently inserted into BUSCO genes, the remnants left behind following historical TE transposition events, or TE sequences that are immediately adjacent to and inadvertently classified as BUSCO sequences.”

2. One thing that I noticed throughout the figures is that branch B1, leading to A. sexmaculata, the branch leading to clade A, and the branch leading to clade B (as labeled in Figures 1 and 2) appear to form a polytomy. I don't find this mentioned in the text and am wondering why this relationship remains unresolved with these data. I don't think this has any bearing on the results, since all analyses are done on the tips of the tree, but I think readers looking at these trees will want to know what is going on at that node.

Response: As shown in supplementary S48, the position of the branch B1 leading to A. sexmaculata is not supported in this study. Both the local posterior probability (48) and the site/ gene concordance values (31, 35) show that there is conflicting signal in the single locus trees. Moreover, the internode length is very short. For this reason, we collapsed the tree in figures 1 and 2 in the main text. As written in the figure legends, we collapsed nodes with less than 0.9 local posterior probability. We adjusted this unresolved node according to Thomas et al. (2020). While this incongruence is interesting from a phylogenetic standpoint, more data is necessary to assess the phylogenetic position of Hydroptilidae (Figs. 1 & 2: Clade B1, supplementary Fig. S48: Agraylea sexmaculata) which remains underrepresented in genomic research.

3. The authors use custom scripts for their BUSCO-TE correlation analysis and provide a link to a Box folder on line 514. I would request that these scripts be put somewhere more stable and accessible (e.g., github). Not only was I asked to login when clicking the link, but after I had done so that link didn't seem to exist.

Response: We are sorry that the reviewer was unable to access the data. We created a repository for the TE-BUSCO gene intersect analysis including all custom scripts and a description of the whole pipeline: https://github.com/jhcaddisfly/TE-gene_intersect_analysis

Data supporting the results will be submitted to GigaDB and can be accessed under this link without logging in: https://byu.box.com/v/trich-genomes
Minor/editorial points
1. Would the authors be able to report concordance factors for the species tree? I think this should be easy enough with IQ-tree and is something I ask everyone to do. This may also help answer my question about the polytomy.

Response: We computed the concordance factors as described in lines 473-476 and included these in the supplementary figure S48. The position of the branch B1 is not supported (Astral support value: 48). The low site concordance (31) and gene concordance (35) values also suggest that is disconcordant signal in the single locus trees. For this reason, the position of A. sexmaculata remains unresolved.

2. The authors do a good job of mentioning and citing programs used throughout the manuscript but seem to skip this in the Assembly section (starting on Line 398). "First, we applied a long-read assembly method…" Which one? Same for "de novo hybrid assembly approaches." I see that assembly is covered in detail in the Supplement, but I think naming the main programs used (wbtdbg2 and Masurca) should be in the main text.

Response: We added the main programs used to the main text (lines 433, 436).

3. Line 281-282: I think some of the brackets and parentheses here are mismatched or un-closed.

Response: We corrected this (line 312).

Close
