# Supplementary material for: Genome size evolution in the diverse insect order Trichoptera
Source: Gigascience. 2022 Feb 25;11:giac011. doi: 10.1093/gigascience/giac011 (PMC8881205; doi:10.1093/gigascience/giac011)
Supplement: giac011_Response_to_Reviewer_Comments_Revision_2 [file giac011_response_to_reviewer_comments_revision_2.pdf]

Author's Response To Reviewer Comments

Close

Cover letter
Dear Hongling Zhou, and, dear Editorial Board,

We would like to re-submit our revised manuscript entitled “Genome size evolution in the diverse insect order Trichoptera (caddisflies)” for review after incorporating feedback from the reviewer.

We thank both reviewers for reading our revised version of the manuscript. We, especially, thank reviewer 1 for their constructive feedback which we have carefully considered.

We made changes to the manuscript and believe that the manuscript has improved as a result. The revised manuscript addresses the concerns raised and was submitted via the submission portal (with and without using Track Changes). A point-by-point response to the reviewers’ suggestions can be found below.

Again, we thank you for the opportunity to revise our manuscript. Please let us know if there is anything else you require from us.

Sincerely,

Jacqueline Heckenhauer on behalf of all authors

Point-by-point 'Response to Reviewers':

Reviewer reports:
Reviewer #1: I thank the authors for their well-written, thorough, and detailed responses to my concerns and those of the other reviewer. I believe most of my concerns have been satisfactorily addressed. I find the revised manuscript suitable for publication, after one minor issue is further addressed (see below). The scale of data and analyses in this paper is no small feat.
Minor comment:
I appreciate that the authors have run smudgeplot analyses on their data, but I do notice a few inconsistencies in coverage estimates between those based on sequencing effort and the backmapping approach, and the kmer approaches. It is, therefore, in my opinion, still worth comparing the kcov to actual sequencing effort for the samples where smudgeplots are unclear and/or inconsistent with the other coverage estimates. I realise these two methodological approaches are based on different data streams (kmers versus assemblies), but considering their shared origin, they are still worth comparing.
See example below:
O. albicorne has varying coverage estimates (sequencing effort, 2n: 46.7, backmapping, 2n: 58, genomescope, 1n: 24.8, smudgeplot, 1n: 53.8)
If you convert sequencing-based coverage to kcov:
Kcov, 1n =(((read length -k + 1)* coverage)/read length)/2
This gives a kcov 1n estimate between 20.3 and 25.1, more in line with the genomescope estimate, suggesting that smudgeplot should be re-run with manually entered kcov as predicted by genomescope
Other incongruent examples, where at least one of the coverage estimates is inconsistent, include: H. phryganea, H. magnus, L. basale, andM. longulum
This comparison, and the adjustment of the genomescope kcov, should tidy up a few lose ends and

overall help support more of the results of the various analyses. This may suggest that some of these species might not be diploid, but I do agree with the authors that further sequencing would need to be done to confirm this, and this is outside the scope of this manuscript. A brief mention of these different lines of evidence for/against polyploidy, and their (dis)agreement with each other should be sufficient once the above samples are addressed.

Response:
We thank reviewer 1 for their constructive feedback which we addressed as follows:

1. We calculated kcov for each sample and modified "DataS1_Sup.8-Comparison_Coverage_kcov_backmap.pl_GenomeScope2_Smudgeplots". This file now includes two additional columns: the kcov for each sample and the backmapping coverage (which is also reported in DataS1_Sup.5-Backmap.pl_Results). This makes it easier for the reader to compare the different lines of evidence, i.e. sequencing based coverage (2n), kcov (1n), backmapping coverage (2n), genomescope2 kcov (1n), and coverage used in smudgeplot (1n).

2. We refer to incongruencies of coverage estimates pointed out by the reviewer (O. albircorne, H. phryganea, H. magnus, L. basale, and M. longulum) in the supplementary (page 48) and main text at lines 303-311.

3. We re-ran smudgeplot with the manually entered kcov as predicted by Genomescope 2 for O. albircorne, H. phryganea, H. magnus, L. basale, and M. longulum, see lines 303-311 in the main text. The smudgeplots are reported in the supplementary figures S90-93 (H. phyrganeae), S96-98 (H. magnus), S104-S107 (L. basale), S110-S113 (M. longulum ML3), S116-119 (M. longulum ML1), S127-S129 (O. albicorne).

4. We report these new results in the main text (lines 303-311), and discuss the different lines of evidence for/against polyploidy and their (dis)agreement with each other (332-337).

5. As requested by the GigaDB Team we ran BUSCO v5 for the completeness analysis, updated the summary statistic shown in Table 1 with the v5 results and will uploaded the BUSCO results files in GigaDB: 3 files for each run; (i)short_summary.*.txt (ii) full_table.tsv (iii)missing_busco_list.tsv. We changed this in the methods accordingly, lines 118, 469-470.

Close
