# Supplementary material for: Genome size evolution in the diverse insect order Trichoptera
Source: Gigascience. 2022 Feb 25;11:giac011. doi: 10.1093/gigascience/giac011 (PMC8881205; doi:10.1093/gigascience/giac011)
Supplement: giac011_Reviewer_1_Report_Original_Submission — Julie Blommaert -- 8/23/2021 Reviewed [file giac011_reviewer_1_report_original_submission.pdf]

Reviewer Report

Title: Genome size evolution in the diverse insect order Trichoptera

Version: Original Submission **Date:** 8/23/2021

Reviewer name: Julie Blommaert

Reviewer Comments to Author:

Summary of the paper and overall impression
In their paper, "Genome size evolution in the diverse insect order Trichoptera", Heckenhauer et al report a 14-fold variation in genome size in caddis-flies. The authors find evidence for increases in transposable elements associated with larger genomes, and report that in caddis-flies living in less stable environments, some genes are replicated in association with transposable elements. Overall, this paper represents a comprehensive collection of data, however, I have some concerns about some of the reporting of methods, some analyses and conclusions. To support some of the conclusions, namely that WGD or large-scale duplications do not play a role in caddis fly genomes, I believe the authors could perform additional analyses. Further, I was left confused by the descriptions of the methods, especially around the replicated BUSCO gene analyses. Please see my comments below.
Main comments:
1. The authors report that their gene-age distribution analyses do not support the hypothesis of a WGD, but given previous suggestions that WGD are important in these species, the authors should conduct additional analyses (e.g smudgeplot, minor allele frequency distributions in single-copy genes) to rule out this possibility. While it can indeed be difficult to find a balance between the evidence of absence and an absence of evidence, more effort should go into resolving the matter of WGD in caddis-flies. Some of the genomescope peaks, and some of the coverage peaks from the backmap approach seem to at least hint at large-scale duplications or variations in copy number. Further analyses should also consider if assembled gene copies may be collapsed duplicates.
2. I admit I am confused by the terminology around the TE-associated BUSCO genes. Are these cases where BUSCO has reported a high number of duplicates? Or where BUSCO annotated regions have a high coverage? Two things need to be clarified here; what made them stick out in the first place (coverage? Duplications?), and what are they really (TEs that BUSCO mistook for BUSCOs? fragments of real BUSCOs attached to TEs?).
Minor comments:
1. Lines 53-57: "Genome size can vary widely among closely and distantly related species, though our knowledge is still scarce for many non-model groups. This is especially true for insects, which account for much of the earth's species diversity. To date 1,345 insect genome size estimates have been published, representing less than 0.15% of all described insect species." While I appreciate the authors' point that there is a relatively little data available about genome size and only a small proportion of non-model insects in the Animal Genome Size database, this is the case for all groups, and insects actually represent the largest group of invertebrates in the AGSDb. However, this does not mean insects, or chironomid are a poor system to study this in, so authors could reframe this first sentence to justify the

study system with something more than highlighting how understudied this is in insects.
2. Line 76: correct to "In insects, the KNOWN ranges of genomic repeat proportion are…"
3. Lines 89-91: Why are species rich groups a better system to study RE evolution and environmental interactions than e.g. populations, species complexes, recently diverged species, or groups in the process of speciation?
4. Lines 113-115: The data description does not, in my opinion, need to justify the species selection since this is done in the intro
5. Genome size estimates- sequencing based estimates can also be impacted by GC-content, especially in libraries which were produced using PCR, this may be a useful point regarding the differences between FCM and sequencing-based estimates
6. RepMod versions inconsistent Line 463 says v2, earlier says v1
7. Line 468-469- What did you use to merge repmask out files?
8. All read-based analyses: were they run on decontaminated read libraries? If so, please briefly clarify this in the main manuscript. Genome size with GenomeScope: 444-448; RepeatExplorer: Lines 471-479
9. Why only use dnaPipeTE for repeat divergences and not also abundances? Does dnaPipeTE agree with RepeatExplorer?
10. Line 495: What is meant by "BUSCO genes showed regions of unexpected high copy number…"? Are these genes reported by BUSCO as duplicated or is this referring to increased coverage?
11. Lines 506-507: "We used copy number profiles to identify BUSCO genes with repetitive sequences based on coverage profiles" The meaning of this is unclear. The reported copy number from BUSCO? Coverage of mapped reads?
12. Table 1- please report the full BUSCO summary (e.g. C:39.7%[S:39.2%,D:0.5%],F:35.8%,M:24.5%,n:2442) for each species, lumping complete and fragmented together is unneccesary, and readers are usually interested enough in the full complement of BUSCOs that it should not be in the supplements, but in the main paper
13. Coverages from backmap method can and should be compared to genomescope kcov estimates (while correcting for kmer size; see here for a brief explanation https://www.biostars.org/p/221672/), this will validate both approaches and offer further evidence when considering polploidy.
14. In the supplementary note about TAGC plots, Figures S31, S36, S38, S44, S45, S46, S47 don't list contaminant exclusion criteria- if contaminants weren't removed this needs to be stated, and in some cases, especially those where there are different "blobs", (e.g. S47) justified
15. Supplementary note 9: Figure reference is wrong?
16. Supplementary note 10: Can coverage comparisons using average BUSCO coverage be re-run using corrected kcov estimates? This would validate the BUSCO coverage approach.
17. Supp Data 1: Coverage estimates would be more accurate if based on FCM measurements and total sequenced bp (before and after decontamination) and can also be compared to corrected kcov estimates
18. Limnephilus lunatus has too low coverage to get reliable genomescope

Methods

Are the methods appropriate to the aims of the study, are they well described, and are necessary controls included? Choose an item.

Conclusions

Are the conclusions adequately supported by the data shown? Choose an item.

Reporting Standards

Does the manuscript adhere to the journal's guidelines on minimum standards of reporting? Choose an item.

Choose an item.

Statistics

Are you able to assess all statistics in the manuscript, including the appropriateness of statistical tests used? Choose an item.

Quality of Written English

Please indicate the quality of language in the manuscript: Choose an item.

Declaration of Competing Interests

Please complete a declaration of competing interests, considering the following questions:

- Have you in the past five years received reimbursements, fees, funding, or salary from an organisation that may in any way gain or lose financially from the publication of this manuscript, either now or in the future?
- Do you hold any stocks or shares in an organisation that may in any way gain or lose financially from the publication of this manuscript, either now or in the future?
- Do you hold or are you currently applying for any patents relating to the content of the manuscript?
- Have you received reimbursements, fees, funding, or salary from an organization that holds or has applied for patents relating to the content of the manuscript?
- Do you have any other financial competing interests?
- Do you have any non-financial competing interests in relation to this paper?

If you can answer no to all of the above, write 'I declare that I have no competing interests' below. If your reply is yes to any, please give details below.

I declare that I have no competing interests

I agree to the open peer review policy of the journal. I understand that my name will be included on my report to the authors and, if the manuscript is accepted for publication, my named report including any attachments I upload will be posted on the website along with the authors' responses. I agree for my report to be made available under an Open Access Creative Commons CC-BY license (http://creativecommons.org/licenses/by/4.0/). I understand that any comments which I do not wish to be included in my named report can be included as confidential comments to the editors, which will not be published.

Choose an item.

To further support our reviewers, we have joined with Publons, where you can gain additional credit to further highlight your hard work (see: https://publons.com/journal/530/gigascience). On publication of this paper, your review will be automatically added to Publons, you can then choose whether or not to claim your Publons credit. I understand this statement.

Yes Choose an item.
