# Supplementary material for: Genome size evolution in the diverse insect order Trichoptera
Source: Gigascience. 2022 Feb 25;11:giac011. doi: 10.1093/gigascience/giac011 (PMC8881205; doi:10.1093/gigascience/giac011)
Supplement: giac011_Reviewer_1_Report_Revision_1 — Julie Blommaert -- 11/30/2021 Reviewed [file giac011_reviewer_1_report_revision_1.pdf]

Reviewer Report

Title: Genome size evolution in the diverse insect order Trichoptera

Version: Revision 1 **Date:** 11/30/2021

Reviewer name: Julie Blommaert

Reviewer Comments to Author:

I thank the authors for their well-written, thorough, and detailed responses to my concerns and those of the other reviewer. I believe most of my concerns have been satisfactorily addressed. I find the revised manuscript suitable for publication, after one minor issue is further addressed (see below). The scale of data and analyses in this paper is no small feat.

Minor comment:

I appreciate that the authors have run smudgeplot analyses on their data, but I do notice a few inconsistencies in coverage estimates between those based on sequencing effort and the backmapping approach, and the kmer approaches. It is, therefore, in my opinion, still worth comparing the kcov to actual sequencing effort for the samples where smudgeplots are unclear and/or inconsistent with the other coverage estimates. I realise these two methodological approaches are based on different data streams (kmers versus assemblies), but considering their shared origin, they are still worth comparing.

See example below:

O. albicorne has varying coverage estimates (sequencing effort, 2n: 46.7, backmapping, 2n: 58, genomescope, 1n: 24.8, smudgeplot, 1n: 53.8)

If you convert sequencing-based coverage to kcov:

Kcov, 1n =(((read length -k + 1)* coverage)/read length)/2

This gives a kcov 1n estimate between 20.3 and 25.1, more in line with the genomescope estimate, suggesting that smudgeplot should be re-run with manually entered kcov as predicted by genomescope

Other incongruent examples, where at least one of the coverage estimates is inconsistent, include: H. phryganea, H. magnus, L. basale, andM. longulum

This comparison, and the adjustment of the genomescope kcov, should tidy up a few lose ends and overall help support more of the results of the various analyses. This may suggest that some of these species might not be diploid, but I do agree with the authors that further sequencing would need to be done to confirm this, and this is outside the scope of this manuscript. A brief mention of these different lines of evidence for/against polyploidy, and their (dis)agreement with each other should be sufficient once the above samples are addressed.

Methods

Are the methods appropriate to the aims of the study, are they well described, and are necessary controls included? Choose an item.

Conclusions

Are the conclusions adequately supported by the data shown? Choose an item.

Reporting Standards

Does the manuscript adhere to the journal's guidelines on minimum standards of reporting? Choose an item.

Choose an item.

Statistics

Are you able to assess all statistics in the manuscript, including the appropriateness of statistical tests used? Choose an item.

Quality of Written English

Please indicate the quality of language in the manuscript: Choose an item.

Declaration of Competing Interests

Please complete a declaration of competing interests, considering the following questions:

- Have you in the past five years received reimbursements, fees, funding, or salary from an organisation that may in any way gain or lose financially from the publication of this manuscript, either now or in the future?
- Do you hold any stocks or shares in an organisation that may in any way gain or lose financially from the publication of this manuscript, either now or in the future?
- Do you hold or are you currently applying for any patents relating to the content of the manuscript?
- Have you received reimbursements, fees, funding, or salary from an organization that holds or has applied for patents relating to the content of the manuscript?
- Do you have any other financial competing interests?
- Do you have any non-financial competing interests in relation to this paper?

If you can answer no to all of the above, write 'I declare that I have no competing interests' below. If your reply is yes to any, please give details below.

I declare that I have no competing interests

I agree to the open peer review policy of the journal. I understand that my name will be included on my report to the authors and, if the manuscript is accepted for publication, my named report including any attachments I upload will be posted on the website along with the authors' responses. I agree for my report to be made available under an Open Access Creative Commons CC-BY license (http://creativecommons.org/licenses/by/4.0/). I understand that any comments which I do not wish to

be included in my named report can be included as confidential comments to the editors, which will not be published.

Choose an item.

To further support our reviewers, we have joined with Publons, where you can gain additional credit to further highlight your hard work (see: https://publons.com/journal/530/gigascience). On publication of this paper, your review will be automatically added to Publons, you can then choose whether or not to claim your Publons credit. I understand this statement.

Yes Choose an item.
