# Supplementary material for: Genome size evolution in the diverse insect order Trichoptera
Source: Gigascience. 2022 Feb 25;11:giac011. doi: 10.1093/gigascience/giac011 (PMC8881205; doi:10.1093/gigascience/giac011)
Supplement: giac011_Reviewer_2_Report_Original_Submission — Gregg Thomas -- 8/27/2021 Reviewed [file giac011_reviewer_2_report_original_submission.pdf]

Reviewer Report

Title: Genome size evolution in the diverse insect order Trichoptera

Version: Original Submission **Date:** 8/27/2021

Reviewer name: Gregg Thomas

Reviewer Comments to Author:

This paper presents 17 new insect genomes from the order of caddisflies (Trichoptera). The authors combine these genomes with 9 previously sequenced genomes to analyze genome size evolution across the order. They find that genome size tends to correlate with evolution of repeat elements, specifically expansion of transposable elements (TEs). Interestingly, the authors also notice that TE expansions also correlate with gene copy-number (or gene fragment copy-number), even of highly conserved genes used to assess genome completeness. Overall, I find this paper very well written and easy to follow. The genomic resources and analyses presented provide novel new resources and findings for insects in the order Trichoptera, with potential implications beyond. I have only minor suggestions before publication, outlined below.

1. Regarding the TE and BUSCO gene fragment associations, while I think this is a really interesting analysis, I found the underlying models a bit difficult to understand.
Line 236 reads, "To test whether repetitive fragments were due to TE insertions near or in the BUSCO genes or, conversely, due to the proliferation of 'true' BUSCO protein-coding gene fragments…"
Is the idea that a BUSCO gene has been duplicated itself and then one copy is either fragmented by a TE insertion or hitch-hikes with a TE (as mentioned on line 501)? Or are these fragments only of BUSCO genes that didn't match a full BUSCO gene at all, but the fragments that did match had unexpectedly high coverage? I guess I'm just confused as to whether a gene duplication needs to precede the TE insertions/hitch-hiking, which is subsequently pseudogenized either prior to or because of the TE activity, or if these are gene losses. I understand how the TE could inflate the coverage of these fragments, but I guess I'm still not clear on how these fragments arise in the first place. Any clarification would be helpful!
Also, if the case is that these are fragments of BUSCO genes that have no full matches in the genome, how might assembly contiguity or quality be affecting these matches?

2. One thing that I noticed throughout the figures is that branch B1, leading to A. sexmaculata, the branch leading to clade A, and the branch leading to clade B (as labeled in Figures 1 and 2) appear to form a polytomy. I don't find this mentioned in the text and am wondering why this relationship remains unresolved with these data. I don't think this has any bearing on the results, since all analyses are done on the tips of the tree, but I think readers looking at these trees will want to know what is going on at that node.

3. The authors use custom scripts for their BUSCO-TE correlation analysis and provide a link to a Box folder on line 514. I would request that these scripts be put somewhere more stable and accessible (e.g., github). Not only was I asked to login when clicking the link, but after I had done so that link didn't seem to exist.

Minor/editorial points
1. Would the authors be able to report concordance factors for the species tree? I think this should be easy enough with IQ-tree and is something I ask everyone to do. This may also help answer my question about the polytomy.
2. The authors do a good job of mentioning and citing programs used throughout the manuscript but seem to skip this in the Assembly section (starting on Line 398). "First, we applied a long-read assembly method…" Which one? Same for "de novo hybrid assembly approaches." I see that assembly is covered in detail in the Supplement, but I think naming the main programs used (wbtdbg2 and Masurca) should be in the main text.
3. Line 281-282: I think some of the brackets and parentheses here are mismatched or un-closed.

Methods

Are the methods appropriate to the aims of the study, are they well described, and are necessary controls included? Choose an item.

Conclusions

Are the conclusions adequately supported by the data shown? Choose an item.

Reporting Standards

Does the manuscript adhere to the journal's guidelines on [minimum standards of reporting?]() Choose an item.

Choose an item.

Statistics

Are you able to assess all statistics in the manuscript, including the appropriateness of statistical tests used? Choose an item.

Quality of Written English

Please indicate the quality of language in the manuscript: Choose an item.

Declaration of Competing Interests

Please complete a declaration of competing interests, considering the following questions:

- Have you in the past five years received reimbursements, fees, funding, or salary from an organisation that may in any way gain or lose financially from the publication of this manuscript, either now or in the future?

- Do you hold any stocks or shares in an organisation that may in any way gain or lose financially from the publication of this manuscript, either now or in the future?
- Do you hold or are you currently applying for any patents relating to the content of the manuscript?
- Have you received reimbursements, fees, funding, or salary from an organization that holds or has applied for patents relating to the content of the manuscript?
- Do you have any other financial competing interests?
- Do you have any non-financial competing interests in relation to this paper?

If you can answer no to all of the above, write 'I declare that I have no competing interests' below. If your reply is yes to any, please give details below.

I declare that I have no competing interests

I agree to the open peer review policy of the journal. I understand that my name will be included on my report to the authors and, if the manuscript is accepted for publication, my named report including any attachments I upload will be posted on the website along with the authors' responses. I agree for my report to be made available under an Open Access Creative Commons CC-BY license (http://creativecommons.org/licenses/by/4.0/). I understand that any comments which I do not wish to be included in my named report can be included as confidential comments to the editors, which will not be published.

Choose an item.

To further support our reviewers, we have joined with Publons, where you can gain additional credit to further highlight your hard work (see: https://publons.com/journal/530/gigascience). On publication of this paper, your review will be automatically added to Publons, you can then choose whether or not to claim your Publons credit. I understand this statement.

Yes Choose an item.
